# Association of *CYP2C19* Loss-of-Function Metabolizer Status With Stroke Risk Among Chinese Patients Treated With Ticagrelor-Aspirin vs Clopidogrel-Aspirin: A Prespecified Secondary Analysis of a Randomized Clinical Trial

**DOI:** 10.1001/jamanetworkopen.2023.17037

**Published:** 2023-06-06

**Authors:** Xuewei Xie, S. Claiborne Johnston, Anxin Wang, Qin Xu, Philip M. Bath, Yuesong Pan, Hao Li, Jinxi Lin, Yilong Wang, Xingquan Zhao, Zixiao Li, Yong Jiang, Liping Liu, Anding Xu, Jing Jing, Xia Meng, Yongjun Wang

**Affiliations:** 1 China National Clinical Research Center for Neurological Diseases, Beijing, China; 2 Department of Neurology, Beijing Tiantan Hospital, Capital Medical University, Beijing, China; 3 Department of Neurology, University of California, San Francisco; 4 Stroke Trials Unit, Division of Clinical Neuroscience, University of Nottingham, Nottingham, United Kingdom; 5 Department of Neurology, The First Affiliated Hospital of Jinan University, Guangzhou, Guangdong Province, China; 6 Advanced Innovation Center for Human Brain Protection, Beijing Tiantan Hospital, Capital Medical University, Beijing, China

## Abstract

**Importance:**

The Clopidogrel With Aspirin in High-Risk Patients With Acute Nondisabling Cerebrovascular Events II (CHANCE-2) trial showed that ticagrelor-aspirin combination therapy reduced the risk of stroke compared with a clopidogrel-aspirin combination among carriers of *CYP2C19* loss-of-function (LOF) alleles after a transient ischemic attack (TIA) or minor ischemic stroke. However, the association between the degree of *CYP2C19* LOF and ideal treatment allocation remains unknown.

**Objective:**

To investigate whether the efficacy and safety of ticagrelor-aspirin vs clopidogrel-aspirin are consistent with the expected degree of *CYP2C19* LOF after TIA or minor stroke.

**Design, Setting, and Participants:**

CHANCE-2 was a multicenter, double-blind, double-dummy, placebo-controlled randomized clinical trial. Patients were enrolled at 202 centers in China from September 23, 2019, through March 22, 2021. Patients with at least two *2 or *3 alleles (*2/*2, *2/*3, or *3/*3) according to point-of-care genotyping were classified as “poor metabolizers,” and those with one *2 or *3 allele (*1/*2 or *1/*3) were classified as “intermediate metabolizers.”

**Interventions:**

Patients were randomly assigned in a 1:1 ratio to receive ticagrelor (180-mg loading dose on day 1 followed by 90 mg twice daily for days 2-90) or clopidogrel (300-mg loading dose on day 1 followed by 75 mg/d for days 2-90). All patients received aspirin (75- to 300-mg loading dose followed by 75 mg/d for 21 days).

**Main Outcomes and Measures:**

The primary efficacy outcome was a new ischemic or hemorrhagic stroke. The secondary efficacy outcome was a composite of new clinical vascular events and individual ischemic stroke events within 3 months. The primary safety outcome was severe or moderate bleeding. Analyses were performed according to the intention-to-treat principle.

**Results:**

Of the 6412 patients enrolled, the median age was 64.8 years (IQR, 57.0-71.4 years), and 4242 patients (66.2%) were men. Of the 6412 patients, 5001 (78.0%) were intermediate metabolizers, and 1411 (22.0%) were poor metabolizers. The primary outcome occurred less often with ticagrelor-aspirin vs clopidogrel-aspirin, irrespective of metabolizer status (6.0% [150 of 2486] vs 7.6% [191 of 2515]; hazard ratio [HR], 0.78 [95% CI, 0.63-0.97] among intermediate metabolizers and 5.7% [41 of 719] vs 7.5% [52 of 692]; HR, 0.77 [95% CI, 0.50-1.18] among poor metabolizers; *P* = .88 for interaction). Patients taking ticagrelor-aspirin had a higher risk of any bleeding event compared with those taking clopidogrel-aspirin, irrespective of metabolizer status: 5.4% (134 of 2486) vs 2.6% (66 of 2512) (HR, 2.14 [95% CI, 1.59-2.89]) among intermediate metabolizers and 5.0% (36 of 719) vs 2.0% (14 of 692) (HR, 2.99 [95% CI, 1.51-5.93]) among poor metabolizers (*P* = .66 for interaction).

**Conclusions and Relevance:**

This prespecified analysis of a randomized clinical trial found no difference in treatment effect between poor and intermediate *CYP2C19* metabolizers. The relative clinical efficacy and safety of ticagrelor-aspirin vs clopidogrel-aspirin were consistent across *CYP2C19* genotypes.

**Trial Registration:**

ClinicalTrials.gov Identifier: NCT04078737

## Key Points

**Question:**

Does *CYP2C19* loss-of-function metabolizer status, which blunts metabolism of clopidogrel to its active form, modify the degree of benefit of ticagrelor-aspirin or clopidogrel-aspirin for patients with minor stroke or transient ischemic attack?

**Findings:**

In this prespecified subgroup analysis of the CHANCE-2 trial, new stroke occurred less often with ticagrelor-aspirin vs clopidogrel-aspirin, irrespective of metabolizer status: 6.0% vs 7.6%, respectively, among patients with intermediate metabolizer status and 5.7% vs 7.5%, respectively, among poor metabolizers. No difference was found in treatment effect between poor and intermediate *CYP2C19* metabolizers.

**Meaning:**

These findings suggest that it may be advisable to consider using ticagrelor-aspirin not only for poor metabolizers but also intermediate metabolizers for patients with transient ischemic attack or minor stroke, although caution should be taken when using ticagrelor added to aspirin due to the risk of any bleeding, particularly mild bleeding.

## Teaser

This prespecified subgroup analysis of the randomized clinical CHANCE-2 trial investigates whether the efficacy and safety of ticagrelor-aspirin vs clopidogrel-aspirin are consistent with the expected degree of *CYP2C19* loss-of-function after transient ischemic attack or minor stroke.

## Introduction

Dual antiplatelet therapy with aspirin and clopidogrel is the cornerstone therapy for the prevention of recurrent stroke events among patients with acute minor stroke and transient ischemic attacks (TIAs).^1,2,3^ The pharmacodynamic effects of clopidogrel are not uniform, however, and loss-of-function (LOF) alleles of the *CYP2C19* gene (OMIM 124020) are associated with reduced generation of clopidogrel’s active metabolite, diminished platelet inhibition, and increased risk of stroke recurrence.^4,5,6^ Patients carrying LOF alleles and receiving clopidogrel after ischemic stroke have been established to have a higher risk of recurrent stroke than noncarriers.^7,8,9,10^ As shown in the Clopidogrel in High-Risk Patients With Acute Nondisabling Cerebrovascular Events (CHANCE) trial, the use of clopidogrel plus aspirin reduced the risk of a new stroke compared with aspirin alone only for the subgroup of patients who were not carriers of the *CYP2C19* LOF alleles.^8,11^

The subsequent Clopidogrel in High-Risk Patients With Acute Nondisabling Cerebrovascular Events II (CHANCE-2) trial showed that, among patients with TIA or minor ischemic stroke carrying *CYP2C19* LOF alleles who can be treated within 24 hours after the onset of symptoms, the combination of ticagrelor plus aspirin compared with clopidogrel plus aspirin reduced the risk of stroke.^12^ However, based on the specifics of the *CYP2C19* LOF allele, carriers of *CYP2C19* LOF alleles can be further divided into 2 groups: intermediate metabolizers (1 LOF carriers) and poor metabolizers (2 LOF carriers).^13^ Clopidogrel resistance can be overcome by increasing the dose for heterozygous carriers but not for homozygous carriers. The response to clopidogrel may depend on the number of altered alleles of *CYP2C19* (gene-dose effect).^14,15^ Thus, the US Food and Drug Administration–approved drug label (from 2017) gives guidance for *CYP2C19* poor metabolizers to use a different platelet P2Y12 inhibitor; however, it does not give dosing guidance for *CYP2C19* intermediate metabolizers.^16^ It is important to understand whether therapy recommendations should differ between intermediate and poor metabolizers with minor stroke and TIA.

The association of the *CYP2C19* phenotype, genotype, and LOF alleles with clinical outcomes among patients treated with ticagrelor-aspirin vs clopidogrel-aspirin has not been well defined. This study aimed to describe allele, genotype, and phenotype frequencies for *CYP2C19* and evaluate the effect of the *CYP2C19* variants on treatment with ticagrelor-aspirin vs clopidogrel-aspirin in terms of prognosis for patients with minor stroke and high-risk TIA.

## Methods

### Trial Design and Oversight

The trial was approved by the ethics committee at Beijing Tiantan Hospital and each participating site (trial protocol and statistical analysis plan in Supplement 1). Written informed consent was provided by all the patients or their representatives before screening. The details of the rationale and design of the CHANCE-2 trial have been published previously.^17^ Briefly, CHANCE-2 was a multicenter, double-blind, double-dummy, placebo-controlled trial conducted at 202 centers in China. Patients were enrolled from September 23, 2019, through March 22, 2021. Patients with acute nondisabling ischemic stroke (National Institutes of Health Stroke Scale [NIHSS] score ≤3) or high risk of TIA (ABCD^2^ score ≥4 [assesses the risk of stroke on the basis of age, blood pressure, clinical features, duration of TIA, and presence or absence of diabetes, with scores ranging from 0 to 7 and higher indicating greater short-term risk]) who were *CYP2C19* LOF allele carriers were randomly assigned to receive either ticagrelor (180-mg loading dose on day 1 followed by 90 mg twice daily for days 2-90) or clopidogrel (300-mg loading dose on day 1 followed by 75 mg per day for days 2-90) within 24 hours of symptom onset. All patients also received aspirin (75- to 300-mg loading dose followed by 75 mg daily for 21 days).

### Efficacy and Safety Outcomes

The primary efficacy and safety outcomes in this analysis were identical to those of the CHANCE-2 trial.^16^ The primary outcome of the study was any stroke (ischemic or hemorrhagic) within 3 months. Ischemic stroke was defined as an acute focal infarction of the brain or retina with 1 of the following: sudden onset of a new focal neurologic deficit lasting less than 24 hours with clinical or imaging evidence of infarction, or rapid worsening of an existing focal neurologic deficit lasting 24 hours or more with imaging evidence of new ischemic changes clearly distinct from the index ischemic event. Hemorrhagic stroke was defined as acute extravasation of blood into the brain parenchyma or subarachnoid space with associated neurologic symptoms. Secondary efficacy outcomes included new stroke within 30 days, composite vascular events (stroke, TIA, myocardial infarction, and vascular death), ischemic stroke, disabling stroke (with a subsequent modified Rankin Scale score of 2 or higher; range, 0-6, with higher scores reflecting worse outcomes) at 90 days.

The primary safety outcome was severe or moderate bleeding events within 3 months, defined by the criteria from the Global Utilization of Streptokinase and Tissue Plasminogen Activator for Occluded Coronary Arteries (GUSTO) trial.^18^ The secondary safety outcomes were any bleeding, death, adverse events, and severe adverse events through 90 days of follow-up. Severe hemorrhage was defined as fatal or intracranial hemorrhage or other hemorrhage causing hemodynamic compromise requiring blood or fluid replacement, inotropic support, or surgical intervention. Moderate hemorrhage was defined as bleeding that required transfusion of blood but did not lead to hemodynamic compromise requiring intervention. Any bleeding within 3 months was also reported as a secondary safety outcome in this subgroup study.

### Genotyping

The GMEX point-of-care genotyping system (Chongqing Jingyin Bioscience Co Ltd) was used to define *CYP2C19* (*2, *3, and *17) allele status at screening.^19^ This test consists of 4 separate steps intended to be done within a period of 5 to 10 minutes: acquisition of a buccal swab, insertion of the swab into the reagent tube, insertion of the reaction solution into the device, and analysis of *CYP2C19* genotype triggered by the “Run” button on the device. In this trial, patients with the *2 or *3 LOF carrier status (homozygotes [*2/*2, *3/*3, or *2/*3] or heterozygotes [*1/*2, *1/*3, *2/*17, or *3/*17]) were considered eligible for randomization, whereas noncarriers of LOF alleles (*1/*1, *1/*17, or *17/*17) were not.

### Statistical Analysis

Baseline characteristics are presented by antiplatelet treatment groups (ticagrelor-aspirin or clopidogrel-aspirin) and *CYP2C19* phenotype groups (intermediate metabolizers and poor metabolizers). Categorical variables are presented as percentages, and continuous variables as median values with IQRs or mean (SD) values, as appropriate. We performed the analyses according to the intention-to-treat principle for the efficacy and safety outcomes. Interactions between treatment assignment on all outcomes and *CYP2C19* phenotype groups were evaluated by including terms for treatment assignment (ticagrelor-aspirin or clopidogrel-aspirin), *CYP2C19* phenotype (intermediate metabolizers and poor metabolizers), and treatment × metabolizer status interaction as covariates in Cox proportional hazards regression models or logistic regression models. Interaction terms with a 2-tailed *P* < .05 were considered statistically significant.

Participants were censored at their last follow-up assessment when experiencing a clinical event, at the end of the study, or at the time of withdrawal from the study. When there were multiple events of the same type, the time to the first event was used in the model. The cumulative risks of any ischemic or hemorrhagic event and of bleeding events during the 90-day follow-up were reported as Kaplan-Meier estimates. Cox proportional hazards regression methods were used for calculation of hazard ratios (HRs) and 95% CIs. Differences in time to the first event between study groups within the *CYP2C19* phenotypes and genotypes were evaluated using Cox proportional hazards regression models, and HRs were reported with 95% CIs. All statistical analyses were performed with the use of SAS software, version 9.4 (SAS Institute Inc). The study followed the Consolidated Standards of Reporting Trials (CONSORT) reporting guideline.

## Results

### Patient Demographic and Baseline Characteristics

A total of 6412 patients (median age, 64.8 years [IQR, 57.0-71.4 years]; 4242 men [66.2%]) with ischemic stroke or TIA were enrolled from 202 clinical sites in the CHANCE-2 trials, with 3205 randomly assigned to the ticagrelor-aspirin group and 3207 to the clopidogrel-aspirin group.^12^ All randomized patients were included in this secondary analysis. A patient flow diagram is shown in eFigure 1 in Supplement 2. Of the 6112 patients, 5001 (78.0%) were carriers of 1 LOF allele (*1/*2, *1/*3, *2/*17, or *3/*17), and 1411 (22.0%) were carriers of more than 1 LOF allele (*2/*2, *3/*3, or *2/*3). Baseline demographics, clinical presentation, and medical history were mostly well balanced between the intermediate metabolizers and poor metabolizers, except for a slightly lower NIHSS score and greater incidence of a history of myocardial infarction among the intermediate metabolizers group (Table 1). Baseline characteristics of patients classified by *CYP2C19* genotype are presented in eTable 1 in Supplement 2.

**Table 1.** Baseline Characteristics of the Patients with Different *CYP2C19* Phenotypes

Characteristic	Intermediate metabolizers			Poor metabolizers			*P* value^a^
	Total (n = 5001)	Ticagrelor-aspirin (n = 2486)	Clopidogrel-aspirin (n = 2515)	Total (n = 1411)	Ticagrelor-aspirin (n = 719)	Clopidogrel-aspirin (n = 692)	
Median age, (IQR), y	64.8 (57.0-71.4)	65.2 (57.0-71.6)	64.5 (56.9-71.1)	64.6 (56.9-71.5)	64.3 (57.0-71.9)	64.9 (56.9-71.0)	.96
Sex, No. (%)							
Female	1691 (33.8)	855 (34.4)	836 (33.2)	479 (34.0)	235 (32.7)	244 (35.3)	.93
Male	3310 (66.2)	1631 (65.6)	1679 (66.8)	932 (66.0)	484 (67.3)	448 (64.7)	
Han ethnicity, No. (%)	4898 (97.9)	2440 (98.1)	2458 (97.7)	1384 (98.1)	704 (97.9)	680 (98.3)	.73
Median BMI (IQR)	24.5 (22.6-26.6)	24.5 (22.7-26.7)	24.3 (22.5-26.5)	24.3 (22.6-26.6)	24.4 (22.5-26.6)	24.3 (22.6-26.5)	.91
Median blood pressure (IQR), mm Hg							
Systolic	148 (136-161)	148 (136-162)	148 (135-161)	149 (136-163)	149 (136-163)	149 (136-162)	.15
Diastolic	86 (80-95)	86 (80-95)	86 (80-95)	87 (80-95)	87 (80-95)	87 (80-95)	.83
Medical history, No. (%)							
Hypertension	3688 (73.8)	1823 (73.3)	1865 (74.2)	1042 (73.8)	533 (74.1)	509 (73.6)	.94
Diabetes mellitus	1588 (31.8)	799 (32.1)	789 (31.4)	454 (32.2)	234 (32.5)	220 (31.8)	.76
Dyslipidemia	479 (9.6)	254 (10.2)	225 (8.9)	135 (9.6)	69 (9.6)	66 (9.5)	.99
Previous ischemic stroke	1038 (20.8)	503 (20.2)	535 (21.3)	312 (22.1)	166 (23.1)	146 (21.1)	.27
Previous TIA	70 (1.4)	36 (1.5)	34 (1.4)	18 (1.3)	10 (1.4)	8 (1.2)	.72
Myocardial infarction	83 (1.7)	47 (1.9)	36 (1.4)	13 (0.9)	7 (1.0)	6 (0.9)	.04
Angina	139 (2.8)	63 (2.5)	76 (3.0)	38 (2.7)	22 (3.1)	16 (2.3)	.86
Peripheral vascular disease	9 (0.2)	5 (0.2)	4 (0.2)	4 (0.3)	1 (0.1)	3 (0.4)	.45
Median time to randomization (IQR), h	13.9 (8.9-20.4)	13.7 (9.0-20.2)	14.1 (8.8-20.5)	14.1 (8.9-20.9)	13.4 (8.7-20.5)	15.0 (9.2-21.1)	.32
Time to randomization, No. (%)							
<12 h	2065 (41.3)	1017 (40.9)	1048 (41.7)	561 (39.8)	311 (43.3)	250 (36.1)	.30
≥12 h	2936 (58.7)	1469 (59.1)	1467 (58.3)	850 (60.2)	408 (56.8)	442 (63.9)	
Qualifying event, No. (%)							
Ischemic stroke	4042 (80.8)	2002 (80.5)	2040 (81.1)	1116 (79.1)	575 (80.0)	541 (78.2)	.15
TIA	959 (19.2)	484 (19.5)	475 (18.9)	295 (20.9)	144 (20.0)	151 (21.8)	
Median NIHSS score among patients with qualifying ischemic stroke (IQR)	2 (1-3)	2 (1-3)	2 (1-3)	2 (1-3)	2 (1-3)	2 (1-3)	.01
Median ABCD^2^ score among patients with qualifying TIA (IQR)^b^	5 (4-5)	5 (4-5)	5 (4-5)	4 (4-5)	4 (4-5)	4 (4-5)	.18
Previous antiplatelet therapy, No. (%)^c^	588 (11.8)	293 (11.8)	295 (11.7)	160 (11.3)	92 (12.8)	68 (9.8)	.67
Previous lipid-lowering therapy, No. (%)^c^	391 (7.8)	202 (8.1)	189 (7.5)	108 (7.7)	56 (7.8)	52 (7.5)	.84
Low-density lipoprotein cholesterol (IQR), mg/dL	108.1 (84.9-131.3)	108.1 (84.9-131.3)	108.1 (84.9-131.3)	108.1 (88.8-135.1)	112.0 (88.8-135.1)	108.1 (84.9-135.1)	.12

Abbreviations: BMI, body mass index (calculated as weight in kilograms divided by height in meters squared); NIHSS, National Institutes of Health Stroke Scale; TIA, transient ischemic attack.

SI conversion factor: To convert low-density lipoprotein cholesterol to millimoles per liter, multiply by 0.0259.

^a^ For comparisons between intermediate metabolizers and poor metabolizers.

^b^ Data are only for the patients who had a TIA. The ABCD^2^ score assesses the risk of stroke on the basis of age, blood pressure, clinical features, duration of TIA, and presence or absence of diabetes, with scores ranging from 0 to 7 and higher scores indicating greater short-term risk.

^c^ Medication after onset to before randomization.

### Allele, Genotype, and Phenotype Frequencies for *CYP2C19* and Crude Stroke Risk

Frequency distributions for each allele, genotype, and phenotype for *CYP2C19* are shown in Table 2. Carriers of the CYP2C19*2 allele were common, accounting for 87.7% of the study population (5624 of 6412) (71.6% [4593 of 6412] for GA genotypes and 16.1% [1031 of 6412] for AA genotypes), and 17.4% of the genotyped patients (1116 of 6412) were *3 carriers (16.6% [1064 of 6412] for GA genotypes and 0.8% [52 of 6412] for AA genotypes). Gain-of-function allele carriers were rare in this population; 109 of 6412 patients (1.7%) were *17 carriers (CT genotypes). The frequency of intermediate metabolizers of *CYP2C19* was 78.0% (5001 of 6412) and of poor metabolizers of *CYP2C19* was 22.0% (1411 of 6421), in which the most common variant diplotypes were *1/*2 (65.2% [4178 of 6412]) and *2/*2 (16.1% [1031 of 6412]).

**Table 2.** Distribution and Event Rates of New Stroke by Genotypes or Metabolizer Phenotype

Allele and phenotype or genotype	No. (%)					
	Overall		Ticagrelor-aspirin		Clopidogrel-aspirin	
	Frequency (N = 6412)	Event rate	Frequency (n = 3205)	Event rate	Frequency (n = 3170)	Event rate
Alleles						
CYP2C19*2						
GG	788 (12.3)	60 (7.6)	388 (12.1)	33 (8.5)	400 (12.6)	27 (6.8)
GA	4593 (71.6)	306 (6.7)	2298 (71.7)	128 (5.6)	2295 (72.4)	178 (7.8)
AA	1031 (16.1)	68 (6.6)	519 (16.2)	30 (5.8)	512 (16.2)	38 (7.4)
CYP2C19*3						
GG	5296 (82.6)	351 (6.6)	2646 (82.6)	148 (5.6)	2650 (83.6)	203 (7.7)
GA	1064 (16.5)	81 (7.6)	530 (16.5)	42 (7.9)	534 (16.8)	39 (7.3)
AA	52 (0.8)	2 (3.8)	29 (0.9)	1 (3.4)	23 (0.7)	1 (4.3)
CYP2C19*17						
CC	6303 (98.3)	426 (6.8)	3153 (98.4)	237 (7.5)	3150 (99.4)	189 (6.0)
CT	109 (1.7)	8 (7.3)	54 (1.7)	6 (11.1)	55 (1.7)	2 (3.6)
Phenotypes or genotypes^a^						
Intermediate metabolizers	5001 (78.0)	341 (6.8)	2486 (77.6)	150 (6.0)	2515 (0.8)	191 (7.6)
*1/*2	4178 (65.2)	275 (6.6)	2085 (65.1)	116 (5.6)	2093 (66.0)	159 (7.6)
*1/*3	717 (11.2)	58 (8.1)	348 (10.9)	32 (9.2)	369 (11.6)	26 (7.0)
*2/*17 and *3/*17	106 (1.7)	8 (7.5)	53 (1.7)	2 (3.8)	53 (1.7)	6 (11.3)
Poor metabolizers	1411 (22.0)	93 (6.6)	719 (22.4)	41 (5.7)	692 (21.8)	52 (7.5)
*2/*2	1031 (16.1)	68 (6.6)	519 (16.2)	30 (5.8)	512 (16.2)	38 (7.4)
*3/*3	52 (0.8)	2 (3.8)	29 (0.9)	1 (3.4)	23 (0.7)	1 (4.3)
*2/*3	328 (5.1)	23 (7.0)	171 (5.3)	10 (5.8)	157 (5.0)	13 (8.3)

^a^ Patients with at least two *2 or *3 alleles (*2/*2, *2/*3, or *3/*3) according to point-of-care genotyping were classified as poor metabolizers and those with one *2 or *3 allele (*1/*2 or *1/*3) were classified as intermediate metabolizers.

### Efficacy Outcomes

Table 2 also provides the incidence rates for stroke by *CYP2C19* alleles, genotypes, and predicted phenotype. Overall, 341 of 5001 intermediate metabolizers (6.8%) and 93 of 1411 poor metabolizers (6.6%) had a primary efficacy outcome of recurrent stroke at 90 days. As shown in Table 3,^18^ the relative risk reduction for the primary end point with ticagrelor-aspirin vs clopidogrel-aspirin was significant among intermediate metabolizers (HR, 0.78 [95% CI, 0.63-0.97]) and nonsignificant among poor metabolizers (HR, 0.77 [95% CI, 0.50-1.18]; *P* = .88 for treatment × metabolizer status interaction effect). Similar results were observed for the outcomes of composite vascular events, ischemic stroke, and disabling stroke (Table 3).^18^ Relative risk reductions with ticagrelor-aspirin compared with clopidogrel-aspirin for stroke within 30 days were significant among poor metabolizers (HR, 0.62 [95% CI, 0.39-0.99]) but not among intermediate metabolizers (HR, 0.80 [95% CI, 0.63-1.01]; *P* = .32 for treatment × metabolizer status interaction effect). The cumulative risk of new stroke among patients with different metabolizer status by treatment assignment are shown in the Figure. The efficacy outcomes of ticagrelor-aspirin compared with clopidogrel-aspirin among patients with different *CYP2C19* genotypes are shown in eTable 2 and eFigure 2 in Supplement 2. The efficacy of outcomes of metabolizer status among patients with different treatments are shown in eTable 3 in Supplement 2.

**Table 3.** Association of Ticagrelor-Aspirin vs Clopidogrel-Aspirin With Clinical Outcomes Stratified by *CYP2C19* Metabolizer Status

Outcome	Intermediate metabolizers, No. (%)		HR (95% CI)	*P* value	Poor metabolizers, No. (%)		HR (95% CI)	*P* value	*P* value for interaction
	T+A (n = 2486)	C+A (n = 2515)			T+A (n = 719)	C+A (n = 692)			
Primary outcome									
Stroke	150 (6.0)	191 (7.6)	0.78 (0.63-0.97)	.03	41 (5.7)	52 (7.5)	0.77 (0.50-1.18)	.23	.88
Secondary outcome									
Stroke within 30 d	125 (5.0)	157 (6.2)	0.80 (0.63-1.01)	.06	31 (4.3)	48 (6.9)	0.62 (0.39-0.99)	.05	.32
Composite vascular events^a^	174 (7.0)	232 (9.2)	0.75 (0.62-0.92)	.004	55 (7.6)	61 (8.8)	0.88 (0.61-1.29)	.52	.54
Ischemic stroke	148 (6.0)	187 (7.4)	0.79 (0.64-0.98)	.03	41 (5.7)	51 (7.4)	0.79 (0.51-1.21)	.27	.92
Disabling stroke^b^	74 (3.0)	70 (2.8)	1.03 (0.74-1.43)	.85	23 (3.2)	22 (3.2)	1.01 (0.55-1.82)	.99	.91
Primary safety outcome									
Severe or moderate bleeding^c^	7 (0.3)	10 (0.4)	0.70 (0.27-1.85)	.47	2 (0.3)	1 (0.1)	1.58 (0.14-18.19)	.71	.45
Fatal bleeding	2 (0.1)	3 (0.1)	0.62 (0.10-3.74)	.61	1 (0.1)	0	NA		
Intracranial hemorrhage	3 (0.1)	5 (0.2)	0.60 (0.14-2.53)	.49	0	1 (0.1)	NA		
Secondary safety outcome									
Any bleeding	134 (5.4)	66 (2.6)	2.14 (1.59-2.89)	<.001	36 (5.0)	14 (2.0)	2.99 (1.51-5.93)	.002	.66
Mild bleeding^c^	127 (5.1)	56 (2.2)	2.42 (1.75-3.33)	<.001	34 (4.7)	13 (1.9)	3.16 (1.55-6.43)	.002	.85
Mortality	5 (0.2)	15 (0.6)	0.32 (0.12-0.88)	.03	4 (0.6)	3 (0.4)	1.63 (0.29-9.16)	.58	.11
Adverse event	419 (16.9)	339 (13.5)	1.29 (1.12-1.49)	<.001	121 (16.8)	88 (12.7)	1.42 (1.06-1.91)	.02	.90
Serious adverse event	60 (2.4)	63 (2.5)	0.96 (0.67-1.38)	.84	18 (2.5)	21 (3.0)	0.90 (0.46-1.77)	.77	.71

Abbreviations: C+A, clopidogrel-aspirin; HR, hazard ratio; NA, not applicable; T+A, ticagrelor-aspirin.

^a^ Composite vascular events comprise ischemic stroke, hemorrhagic stroke, transient ischemic attack, myocardial infarction, and vascular death.

^b^ A stroke was defined as disabling if the patient had a subsequent modified Rankin scale score greater than 1 (indicating death or any degree of disability).

^c^ Severe or moderate bleeding and mild bleeding were defined in accordance with the Global Utilization of Streptokinase and Tissue Plasminogen Activator for Occluded Coronary Arteries^18^ criteria.

**Figure.**
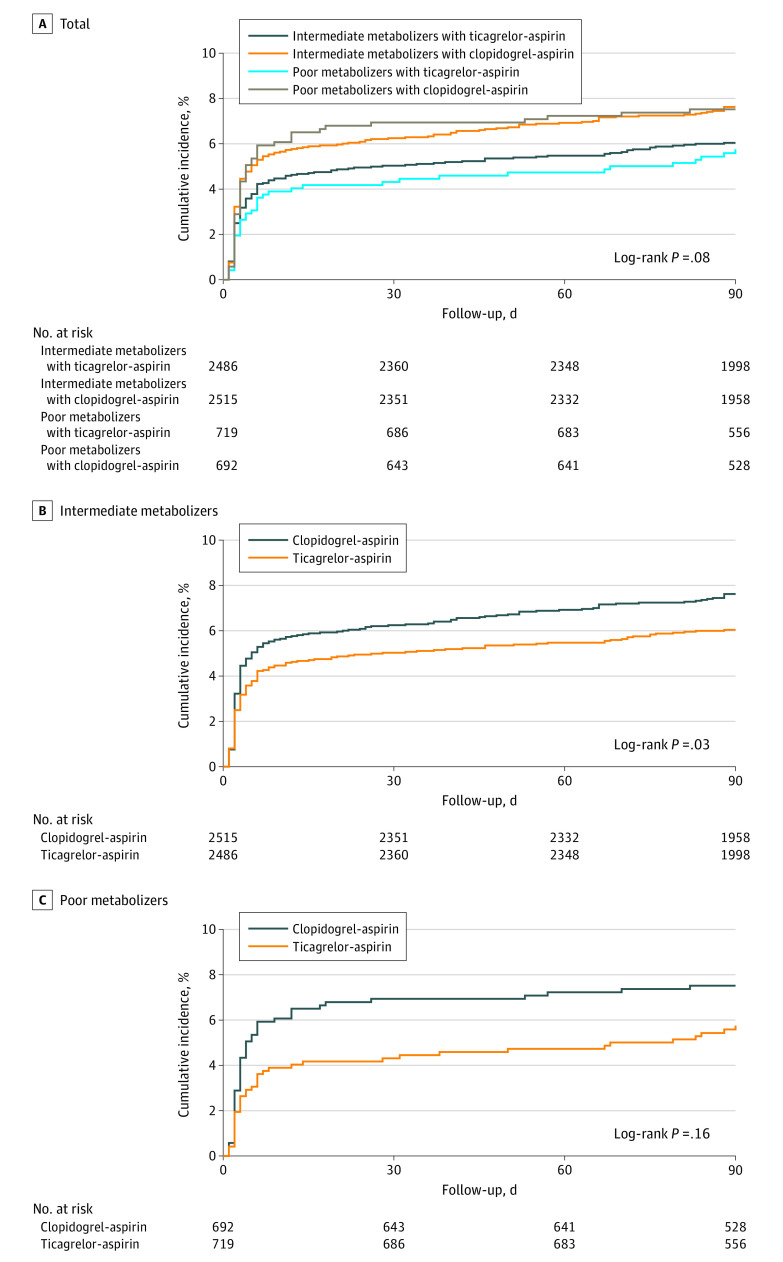
Cumulative Probability of Stroke Recurrence According to Treatment and *CYP2C19* Metabolizer Status

### Safety Outcomes

Treatment assignment was not associated with severe or moderate bleeding for either intermediate metabolizers (HR, 0.70 [95% CI, 0.27-1.85]) or poor metabolizers (HR, 1.58 [95% CI, 0.14-18.29]) and did not differ between the 2 groups (*P* = .45 for interaction). The relative increases in risk for any bleeding with ticagrelor-aspirin compared with clopidogrel-aspirin were similar across predicted phenotypes (intermediate metabolizers: HR, 2.14 [95% CI, 1.59-2.89]; poor metabolizers: HR, 2.99 [95% CI, 1.51-5.93]; *P* = .66 for interaction). The other safety outcomes of ticagrelor-aspirin compared with clopidogrel-aspirin among patients with different *CYP2C19* genotypes are shown in eTable 2 in Supplement 2.

## Discussion

In this prespecified subgroup analysis of the CHANCE-2 trial, we investigated outcomes among patients with TIA or minor ischemic stroke with ticagrelor-aspirin or clopidogrel-aspirin based on background *CYP2C19* genotype and found that new stroke occurred less often with ticagrelor-aspirin vs clopidogrel-aspirin, irrespective of metabolizer status: 6.0% vs 7.6%, respectively, among intermediate metabolizers (a significant 22% relative risk reduction), and 5.7% vs 7.5%, respectively, among poor metabolizers (a nonsignificant 23% relative risk reduction). We found no difference in treatment effect between poor and intermediate *CYP2C19* metabolizers. Compared with clopidogrel-aspirin, ticagrelor-aspirin demonstrated superior potency for prevention of stroke events among both intermediate metabolizers and poor metabolizers. Furthermore, the benefits of treatment with ticagrelor over clopidogrel were achieved without resulting in a significant increase in the incidence of severe or moderate bleeding among intermediate or poor metabolizers. Our study demonstrates that relative risk reductions for the primary end point and key secondary cardiovascular outcomes were similar regardless of *CYP2C19* phenotype.

Various studies have reported that *CYP2C19* polymorphisms vary greatly among racial and ethnic groups. In the CHANCE-2 trial, a total of 4572 (41.6%) noncarriers and 6412 (58.4%) carriers of *CYP2C19* LOF alleles were identified.^12^ Compared with White populations, our Chinese population has a high frequency of *CYP2C19* LOF alleles, mostly attributable to CYP2C19*2, which was present in 87.7% of patients with *CYP2C19* LOF alleles.^20,21^ The prevalence of the LOF allele carriers reported in this study is similar to that in previous studies with East Asian populations (50%-60%) and is much higher than that in studies with African American and White populations (approximately 30%).^22,23,24,25,26^ Given this high prevalence of *CYP2C19* LOF allele carriers among East Asian populations, there is a need to determine the influence of metabolizer phenotype on antiplatelet therapy allocations and clinical outcomes. The results provide evidence supporting genetic testing that may allow clinicians to personalize antiplatelet therapy, especially for East Asian patient populations, for whom the prevalence of *CYP2C19* LOF alleles is high.

It is well established that patients carrying LOF *CYP2C19* alleles have a reduced capacity to metabolize clopidogrel to its active form and that polymorphisms of the *CYP2C19* genotype partially explain the variability in response to clopidogrel. Several studies have shown that the effectiveness of clopidogrel for secondary stroke prevention is reduced among *CYP2C19* LOF carriers.^8,9,27,28,29^ A previously published meta-analysis also found that the carriers of 1 or 2 *CYP2C19* LOF alleles display a significantly increased risk of stroke and composite vascular events compared with noncarriers among patients with ischemic stroke or TIA treated with clopidogrel.^10^ Ticagrelor is one of the alternative agents for this population.^30^ However, the magnitude of any additional benefit of ticagrelor over clopidogrel between carriers of 2 and 1 *CYP2C19* LOF alleles is unclear. The present study found ticagrelor-aspirin to be superior to clopidogrel-aspirin among patients with ischemic stroke regardless of whether they had 1 (intermediate metabolizers) or more than 1 (poor metabolizers) nonfunctional copy of *CYP2C19*. Intermediate metabolizers showed no differences in safety or efficacy from poor metabolizers; the 2 groups could be categorized as a single entity for use of ticagrelor-aspirin for secondary prevention. Intermediate metabolizers are able to process some clopidogrel, so they receive partial benefit from the treatment, while poor metabolizers process little or no clopidogrel, so they receive very limited benefit from it. However, in the CHANCE trial, the rate of stroke was 9.4% (17 of 181) for poor metabolizers and 9.5% (63 of 664) for intermediate metabolizers treated with clopidogrel.^8^ In POINT (Platelet-Oriented Inhibition in New TIA and Minor Ischemic Stroke Trial), the rate of stroke was 0% (0 of 11) for poor metabolizers and 3.3% (3 of 92) for intermediate metabolizers treated with clopidogrel.^26^ There was no large gap in stroke risk between poor metabolizers and intermediate metabolizers with minor stroke and TIA treated with clopidogrel, although events were rare in POINT due to limited genotyping and a lower prevalence of LOF alleles. Thus, similar benefits may occur with ticagrelor for both populations. The 2022 update of the Clinical Pharmacogenetics Implementation Consortium has already increased the strength of recommendation to avoid standard-dose (75 mg) clopidogrel if possible and use prasugrel or ticagrelor at the standard dose if there is no contraindication (from moderate to strong) for *CYP2C19* intermediate metabolizers when considering clopidogrel for cardiovascular indications.^31^ For neurovascular indications, the recommendation (with moderate strength) is to consider an alternative P2Y12 inhibitor at the standard dose if clinically indicated and there is no contraindication. As shown in our study, intermediate metabolizers with minor stroke or TIA had a similar efficacy with ticagrelor compared with clopidogrel, which might partly support the use of an alternative P2Y12 inhibitor not only for poor metabolizers but also for intermediate metabolizers when considering clopidogrel for neurovascular indications. The data from our study indicated that, for patients with minor stroke or TIA, ticagrelor is an alternative to clopidogrel for poor metabolizers as well as for intermediate metabolizers.

The absolute risk of severe or moderate bleeding was low in both treatment groups, and the risk among those treated with ticagrelor-aspirin was not significantly greater than that among those treated with clopidogrel-aspirin regardless of phenotypes for *CYP2C19* polymorphism. This finding differs with the observations in The Acute Stroke or Transient Ischemic Attack Treated With Ticagrelor and Aspirin for Prevention of Stroke and Death (THALES) trial,^30^ in which ticagrelor was associated with a greater risk of severe hemorrhage. In the current analysis, no increase in the incidence of severe or moderate bleeding was reported in the ticagrelor-aspirin group among patients carrying either 1 or 2 *CYP2C19* LOF alleles. This difference may be partly due to the use of single antiplatelet therapy in the control group in the THALES study. The incidence of severe or moderate bleeding therefore cannot be underestimated for patients treated with ticagrelor-aspirin.

### Limitations

Our study has several limitations. First, noncarriers of *CYP2C19* LOF alleles were not included in the CHANCE-2 trial, and the relative effectiveness of ticagrelor among this population remains unknown. The differences between ticagrelor-aspirin and clopidogrel-aspirin in *CYP2C19* normal metabolizers should be explored in future studies. Second, this study was a subgroup analysis, which may increase the possibility of type I error, and had much less statistical power to identify subgroup effects, so our results require confirmation by other studies. Third, this study evaluated only the effect of different *CYP2C19* genotypes on the clinical efficacy of ticagrelor-aspirin compared with clopidogrel-aspirin among patients receiving the drugs after stroke or TIA. The *CYP2C19* genotype contributed importantly to the heterogeneity of clopidogrel response; however, there was still a gap between the *CYP2C19* genotype and clopidogrel’s response. Associations with clopidogrel response and clinical outcomes were not clarified by this study. Fourth, all patients in the CHANCE-2 trial were Chinese, which may limit the generalizability of the findings to other populations.

## Conclusions

In this prespecified secondary analysis of the CHANCE-2 randomized clinical trial, we found no treatment-by-metabolizer status interaction. The relative clinical efficacy and safety of ticagrelor-aspirin vs clopidogrel-aspirin were similar across *CYP2C19* phenotypes that defined intermediate and poor metabolizers. The absolute reduction in new stroke events, the composite end point of clinical vascular events, and individual ischemic stroke events within 3 months were similar in each genotyping group.

## Notes

Supplement 1.
Trial Protocol and Statistical Analysis Plan

Supplement 2. **eTable 1.** Baseline Characteristics of the Patients Classified by *CYP2C19* Genotypes **eTable 2.** Primary Efficacy and Safety Outcomes by Genotypes **eTable 3.** Association of Intermediate in Patients Receiving Ticagrelor-Aspirin or Clopidogrel-Aspirin **eFigure 1.** The Flowchart of the Study **eFigure 2.** Cumulative Probability of Stroke According to Loss-of-Function Allele Carrier Status

Supplement 3.
Data Sharing Statement
