# Supplementary material for: Association of CYP2C19 Loss-of-Function Metabolizer Status With Stroke Risk Among Chinese Patients Treated With Ticagrelor-Aspirin vs Clopidogrel-Aspirin: A Prespecified Secondary Analysis of a Randomized Clinical Trial
Source: JAMA Netw Open. 2023 Jun 6;6(6):e2317037. doi: 10.1001/jamanetworkopen.2023.17037 (PMC10245195; doi:10.1001/jamanetworkopen.2023.17037)
Supplement: Supplement 1. — Trial Protocol and Statistical Analysis Plan [file jamanetwopen-e2317037-s001.pdf]

Clopidogrel with Aspirin in High-risk patients with Acute Non-disabling Cerebrovascular Events II (CHANCE-2)

Protocol

Research team: Beijing Tiantan Hospital, Capital Medical University, Beijing, China

Principal Investigator: Yongjun Wang, MD, Professor of Neurology

Protocol Version 1.0

January 9th 2019

Catalogue

Abstract of research protocol 4
Abrreviations 10

I． Background 14

II. Study purpose....23
III. Study design....24
IV. Study endpoints....26
V. Participant selection 28
VI. Treatments 30
VII. Study conduct....32
VIII. Biological sample collection....41
IX. Data collection....42
X. Study procedures....42
XI. Study risk pre-assessment and risk management....48
XII. Ethical standards 52
XIII. Statistical analysis....54
XIV. Confidentiality and publication of research findings....59
XV. Study Organization....60
XVI. Study monitoring and quality assurance....62
XVII. Data retention 64
XVIII. Data Security Monitoring 64
XIX. References 66
Appendix Table 2. Modified Rankin Scale....75
Appendix Table 3. National institutes of health stroke scale....77
Appendix Table 5. Abnormal clinical laboratory indicators....85
Appendix Table 6. Global Utilization of Streptokinase and Tissue Plasminogen Activator for Occluded Coronary Arteries (GUSTO) bleeding definition....86

Abstract of research protocol

**Brief Title**	**Clopidogrel With Aspirin in High-risk Patients With Acute Non-disabling Cerebrovascular Events II**
**Study objective**	**Primary objective:**<br>To assess the effects of ticagrelor plus aspirin versus clopidogrel plus aspirin on reducing the 3-month risk of any new stroke (ischemic or hemorrhagic, primary outcome) when initiated within 24 hours of symptom onset in CYP2C19 LOF alleles carriers with TIA or minor ischemic stroke (MIS).
**Study settings**	Multicenter, prospective, double-blind, placebo-controlled, randomized clinical trial
**Sample size estimation**	The minimum necessary sample size in the trial is established by the requirement to detect the smallest expected, clinically meaningful treatment difference comparing the treatment with placebo. Based on the genetic sub-analysis of the CHANCE study, we presume that the 90-day risk of stroke recurrence in CYP2C19 loss-of-function allele carriers is about 9.4%, and 6.7% for noncarriers. With the point-of-care identification of the CYP2C19 loss-of-function allele carriers to assess a proper pharmacogenetic approach for patients with high-risk TIA or MIS, we assumed a 25% relative risk reduction with alteration from clopidogrel to ticagrelor (90-day risk of stroke recurrence: 7.1%). Considering the potential impact of the interim analyses on the probability of type I error, we adjusted the statistical significance to a 2-sided α of 0.048. With a sample size of 6,396 patients, we will have 90% power to detect a relative risk reduction (Ticagrelor: loading dose: 180mg, 90mg bid on day 2-90 vs. Clopidogrel: loading dose: 300mg, 75mg qd on day 2-90) of 25% and 5% dropouts (medication nonadherence). Assuming 58.8% prevalence of CYP2C19 loss-of-function allele carriers in a Chinese population, we projected screening 10,878 patients would be necessary.
**Participants**	**Inclusion Criteria**<br>1. 40 years or older than 40 years and less than 80years;<br>2. Acute cerebral ischemic event due to:<br>• Acute non-disabling ischemic stroke (NIHSS≤3 at the time of randomization)or,

	• TIA with moderate-to-high risk of stroke (ABCD2 score ≥ 4 at the time of randomization) or responsible vessel stenosis more than 50%;<br>3. Can be treated with study drug within 24 hours of symptoms onset*(*Symptom onset is defined by the "last seen normal" principle)；<br>4. *CYP2C19* loss-of-function allele carriers；<br>**5.** Informed consent signed.
	**Exclusion Criteria**<br>1. Malformation, tumor, abscess or other major non-ischemic brain disease (e.g., multiple sclerosis) on baseline head CT or MRI.<br>2. Isolated or pure sensory symptoms (e.g., numbness), isolated visual changes, or isolated dizziness/vertigo without evidence of acute infarction on baseline head CT or MRI.<br>3. Preceding moderate or severe dependency (modified Rankin scale [mRS] score 3-5).<br>4. Contraindication to clopidogrel, ticagrelor or aspirin:<br>1) Known allergy<br>2) Severe renal (creatinine exceeding 1.5 times of the upper limit of normal range) or hepatic (ALT or AST > twice the upper limit of normal range) insufficiency<br>3) Severe cardiac failure (NYHA level: III to IV)<br>4) History of hemostatic disorder or systemic bleeding<br>5) History of thrombocytopenia or neutropenia<br>6)History of drug-induced hematologic disorder or hepatic dysfunction<br>7) Low white blood cell ($<2\times10^9$/L) or platelet count ($<100\times10^9$/L)<br>5..Clear indication for anticoagulation (presumed cardiac source of embolus, e.g., atrial fibrillation, prosthetic cardiac valves known or suspected endocarditis)<br>6. Clopidogrel or ticagrelor has been used continuously for ≥5 days before enrollment.<br>7. Used heparin or oral anticoagulant drugs within 10 days before enrollment.<br>8. Undergone intravenous or arterial thrombolysis and mechanical thrombectomy within 24 hours before enrollment.<br>9 History of intracranial hemorrhage or amyloid angiopathy.

	10. History of aneurysm (including intracranial aneurysm and peripheral aneurysm).<br>11. Diagnosis or suspicious diagnosis of acute coronary syndrome<br>12. History of asthma or COPD (chronic obstructive pulmonary disease).<br>13. High-risk for bradyarrhythmia (first-degree or second-degree AV block caused by sinus node disease, and brady-arrhythmic syncope without pacemaker).<br>14. History of hyperuricemia nephropathy.<br>15. Anticipated requirement for long-term (>7 days) non-steroidal anti-inflammatory drugs (NSAIDs).<br>16. History of gastrointestinal bleeding within 3 months before enrollment or major surgery within 30 days.<br>17. Iatrogenic causes (angioplasty or surgery) of minor stroke or TIA.<br>18. Planned or likely revascularization (any angioplasty or vascular surgery) within the next 3 months.<br>19. Scheduled for surgery or interventional treatment requiring study drug cessation.<br>20. Severe non-cardiovascular comorbidity with life expectancy < 3 months.<br>21. Women of childbearing age who have not taken effective contraceptive measures and have a positive pregnancy test record, as well as women who are pregnant or breastfeeding.<br>22. Currently receiving an experimental drug or device.<br>23. Participation in another clinical study with an experimental product during the last 30 days.<br>24. Inability to understand and/or follow research procedures due to mental, cognitive, or emotional disorders.<br>25. Hematocrit (HCT) <30%.
**Treatment Allocation**	Patients meeting the inclusion criteria and offering informed consent will be randomized, and the first dose of study medication will be given within 24 hours of symptom onset Patients will be randomized into 2 groups:<br>● **Ticagrelor and aspirin group**<br>Day1:Ticagrelor 180mg; placebo of clopidogrel 300mg; aspirin 75-300mg (open label)

	Day2nd-21st: Ticagrelor 90mg bid/day; placebo of clopidogrel 75mg; aspirin 75mg (open label)<br>Day 22nd-3 months:Ticagrelor 90mg bid/day; placebo of clopidogrel 75mg<br>• **Clopidogrel and aspirin group**<br>Day 1: Clopidogrel 300mg; placebo of ticagrelor 180mg; aspirin 75-300mg (open label)<br>Day2nd-21st: Clopidogrel 75mg/day; placebo of ticagrelor 90mg bid/day; aspirin 75mg (open label)<br>Day 22nd-3 months:Clopidogrel 75mg; placebo of ticagrelor 90mg bid/day
**Efficacy and safety measures**	**Primary Outcome Measure:**<br>Any new stroke events (ischemic stroke or hemorrhagic stroke) within 3 months<br>**Secondary Outcome Measures:**<br>• Any new stroke events (ischemic stroke or hemorrhagic stroke) within 1 year;<br>• New clinical vascular events (ischemic stroke/ hemorrhagic stroke/ TIA/ myocardial infarction/vascular death) within 3 months and 1 year；<br>• New ischemic stroke events within 3 months and 1 year；<br>• The percentage change of the mRS score between 0-2 points group and 3-6 points group follow-up to 3 months and 1-year.<br>• Neurological impairment (National Institutes of Health Stroke Scale, NIHSS change at 3-month compared to baseline) at 3 months；<br>• Quality of Life (EuroQol EQ-5D scale) at 3 months and at 1 year.<br>At the same time, a stratified analysis of the effective endpoint events will perform: gender stratified analysis (male and female), age stratified analysis (<65 years old and ≥65 years old), etiological subgroup analysis (main subgroups type of ischemic stroke), diabetic (diabetic and non-diabetic), CYP2C19 genotype (intermediate metabolism and poor metabolism), stratified analysis of target events (TIA and minor stroke),

	stratified analysis of responsible blood vessels (intracranial and external arterial stenosis).
	**Safety Outcome Measure**<br>**1) Primary Safety Outcome Measure:：**<br>Moderate and severe bleeding events according to the GUSTO criteria at 3-month<br>**2) Secondary Primary Safety Outcome Measures：**<br>• Severe bleeding events according to the GUSTO criteria at 1-year；<br>• All bleeding events (severe/moderate bleeding and intracranial hemorrhage) at 3-month and 1-year；<br>• Total mortality at 3-month and 1-year；<br>• Symptomatic and asymptomatic intracranial hemorrhage at 3-month and 1-year；<br>• Adverse events/Severe adverse events reported by investigators.
**Statistical considerations**	All statistics will be 2-sided with $P < 0.05$ considered significant.<br>1. Primary null hypothesis: in patients with TIA or minor ischemic stroke carried *CYP2C19* LOF allele treated with aspirin 75 mg/d, there is no difference in 90-day risk of stroke (ischemic or hemorrhagic) in those treated with a 3-month regimen of ticagrelor initiated with a loading dose of 180 mg followed by 90 mg bid compared with a 3-month regimen of clopidogrel initiated with a loading dose of 300 mg followed by 75 mg/d when therapy is initiated within 24 hours of symptom onset.<br>2. Data set of statistical analysis: intention to treat (ITT)<br>3. Statistical analysis：<br>**3.1 Balance of baseline characteristics（comparison between groups）**<br>1）Continuous variables were compared using *t* test or Wilcoxon rank sum test；<br>2）Categorical variables were compared using a $\chi 2$ test, Fisher exact test, or Wilcoxon rank sum test as appropriate.<br>**3.2 Effectiveness analysis**

	1）Primary efficacy outcome：The hazard ratio with 95%CI for the treatment comparison will be derived using a $\chi 2$ test and Logistic regression analysis.<br>2）Secondary efficacy outcome：<br>① Kaplan-Meier estimates of the cumulative risk of stroke or combined vascular events will be reported during the 3-month treatment period. The hazard ratio with 95%CI for the treatment comparison will be derived using a Cox's proportional hazards model. The log-rank test will be used to evaluate the statistical significance of the treatment effect；<br>② Continuous variables were compared using t test or Wilcoxon rank sum test；<br>③ Categorical variables were compared using a $\chi 2$ test, Fisher exact test, or Wilcoxon rank sum test as appropriate；<br>④ Comparison of neurologic impairment and EQ-5D-5L scales were performed using non-parametric analysis<br>⑤ Extreme values were thoroughly checked and corrected before analysis, sensitivity tests were prespecified.<br>**3.3 Safety analysis: Rate of safety end points and adverse events were compared.**<br>Subgroup analyses were based on the carrier status of the *CYP2C19* LOF allele.
**Follow-up schedule**	Study visits will be performed on the day of randomization, at 14 days/discharge, at day21±2, and at day 90±7 via face-to-face interview. Study visit at month 12 (±15 day) will be performed via telephone interview for clinical events.

ABRREVIATIONS

Abbreviations	Interpretation of the meaning
ACEI	Angiotensin converting enzyme inhibitors
ACS	Acute coronary syndrome
AE	Adverse event
AHA	American Heart Association
ALP	Alkaline phosphatase
ANTARCTIC	Platelet function monitoring to adjust antiplatelet therapy in elderly patients stented for an acute coronary syndrome
ALT	Alanine aminotransferase
ADC	Apparent diffusion coefficient
ARCTIC	Double Randomization of a Monitoring Adjusted Antiplatelet Treatment Versus a Common Antiplatelet Treatment for DES Implantation, and Interruption Versus Continuation of Double Antiplatelet Therapy
ARR	Absolute risk reduction
ARU	Aspirin reaction unit
ASA	Aspirin, Acetylsalicylic acid
AST	Aspartate aminotransferase
CABG	Coronary artery bypass graft
CHANCE	Clopidogrel with Aspirin in High-risk patients with Acute Non-disabling Cerebrovascular Events
CHANCE-2	Clopidogrel with Aspirin in High-risk patients with Acute Non-disabling Cerebrovascular Events II
COPD	Chronic obstructive pulmonary disease
Cox1	Cyclooxygenase 1
Cox2	Cyclooxygenase 1
CRA	Clinical research associate
CRF	Case report form

CRO	Contract Research Organization
CT	Computed Tomography
CYP2C19	Cytochrome P450 2C19
CYP3A	Cytochrome P450 3A
DAE	Discontinuation of Investigational Product due to Adverse Event
DICOM	Digital imaging and communications in medicine
DSMB	Data and Safety Monitoring Board
DWI	Diffusion weighted imaging
EDC	Electronic Data Capture System
ELEVATE-TIMI 56	Escalating Clopidogrel by Involving a Genetic Strategy - Thrombolysis In Myocardial Infarction 56
EM	extensive metabolizer
EQ-5D-5L	EuroQol five dimensions questionnaire
FAS	Full analysis set
FLAIR	Fluid attenuated inversion recovery
GCP	Good clinical practice
GPIIb/IIIa	Glycoprotein IIb/IIIa
GRAVITAS	Gauging Responsiveness with A VerifyNow assay-Impact on Thrombosis And Safety
GRE-T2*	Gradient recalled echo-T2*
GUSTO	Global Use of Strategies to Open Occluded Coronary Arteries
Holter	24-hour dynamic electrocardiogram
HR	Hazard ratio
IEC	Independent Ethics Committee
IM	intermediate metabolizer

IRB	Institutional Review Board
ITT	Intention-to-treat
LOCF	Last observation carried forward
LOF	Loss of functional
MRA	Magnetic resonance angiography
MRI	magnetic resonance imaging
mRS	Modified Rankin Scale
NCRC-ND	National Clinical Research Center of Neurological Diseases
NIHSS	National Institute of Health Stroke Score
NYHA	New York Heart Association
PCI	Percutaneous coronary intervention
PCR	Polymerase chain reaction
PLATO	The Study of Platelet Inhibition and Patient Outcomes Trial
PM	Poor metabolizer
POCT	Point-of-care Testing
POPular	Cost-effectiveness of Genotype Guided Treatment With Antiplatelet Drugs in STEMI Patients: Optimization of Treatment
PPI	Proton-pump inhibitor
RAPID GENE	Point-of-care genetic testing for personalisation of antiplatelet treatment
PHARMCLO	Pharmacogenetics of Clopidogrel in Acute Coronary Syndromes
rt-PA	Recombinant Human Tissue Plasminogen Activator
PRINCE	Platelet Reactivity in Acute Non-disabling Cerebrovascular Events
PPS	Per Protocol Set
SAE	Severe adverse event

SAP	Statistical analysis plan
SOCRATES	Acute Stroke or Transient Ischaemic Attack Treated with Aspirin or Ticagrelor and Patient Outcomes
SS	Safety set
SSRIs	Selective Serotonin Reuptake Inhibitor
TAILOR-PCI	Tailored Antiplatelet Therapy Following PCI
UM	ultrarapid metabolizer

I. Background

1. Combined therapy with clopidogrel and aspirin could effectively reduce the recurrent risk of non-disabling ischemic cerebrovascular events and has been recommended by recent guidelines

Non-disabling ischemic cerebrovascular events (NICE), including minor ischemic stroke (MIS) and transient ischemic attack (TIA), accounted for 65% of ischemic cerebrovascular events.[1] Early risk of recurrence after NICE was up to 10% to 20%, but these patients could be neglected for their minor or rapidly improving neurologic deficits. These warning events provide a short window of opportunity for prevention on an urgent basis.[2-5] According to the CHANCE (Clopidogrel in high-risk patients with acute non-disabling cerebrovascular events) study, combined therapy with clopidogrel and aspirin was associated with a 32% relative risk reduction of stroke recurrence within 90 days. (Figure 1) [6] Findings of the CHANCE study provided important evidence of treatment recommendations of Chinese and international guidelines.[7, 8]

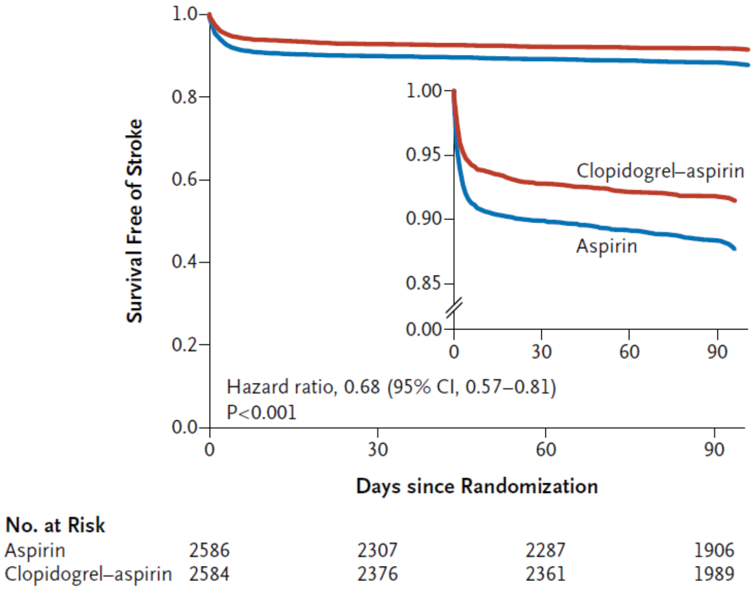

Figure 1： Cumulative Probability of Survival Free of Stroke (Ischemic or Hemorrhagic Stroke) by treatment. Red line for Clopidogrel plus Aspirin, and blue line for Aspirin alone. ***N Engl J Med.*** 2013; 369(1):11-19

2. Carrier status of the *CYP2C19* loss-of-function allele was associated with different response to antiplatelet treatment, especially in Asian patients

In the pharmacogenetic sub-study of the CHANCE trial, the *CYP2C19* LOF carrier genotypes were associated with less protection from subsequent stroke and composite vascular events for patients with NICE in China. Noncarrier of the *CYP2C19* LOF alleles could have an additional 17% benefits from combined therapy of clopidogrel and aspirin. But the use of clopidogrel plus aspirin did not reduced recurrent stroke or composite vascular events in carriers of the *CYP2C19* LOF alleles (Figure 2).[9] In a meta-analysis of 15 studies, carriers of the *CYP2C19* LOF alleles were at increased risk of stroke recurrence (risk ratio, RR:1.92) (Figure 3).10 These findings may justify genetic testing when clopidogrel is otherwise considered the preferred treatment modality, especially in Asian patient populations for whom the prevalence of *CYP2C19* LOF allele is high. As the variation rate of *CYP2C19* gene in Chinese is as high as 58.8%, its impact on the efficacy of clopidogrel is far greater than that in western populations. Therefore, screening suitable populations based on genotyping and accurately formulating effective antiplatelet treatment is a major clinical problem that needs to be resolved urgently.

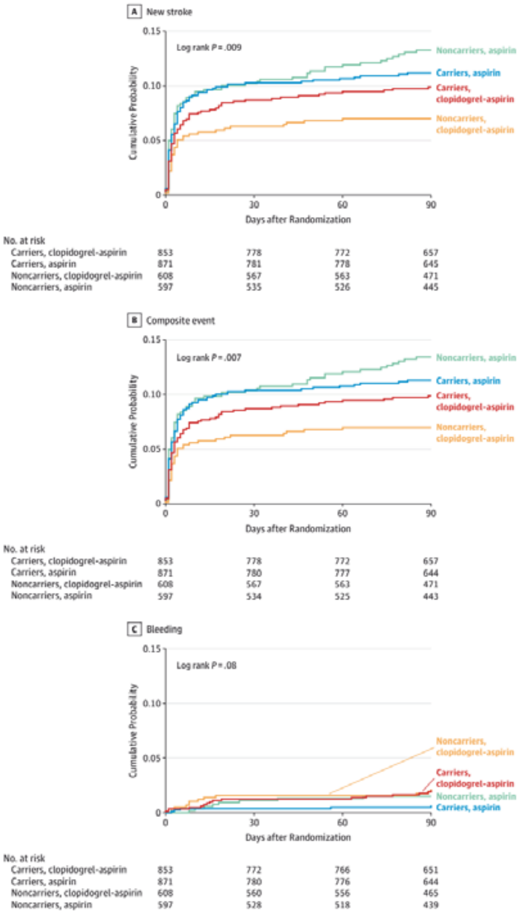

Figure 2： Cumulative Probability of Stroke, Composite Event, and Bleeding According to CYP2C19 Loss-of-Function Allele Carrier Status. JAMA, 2016,316 (1):70-78

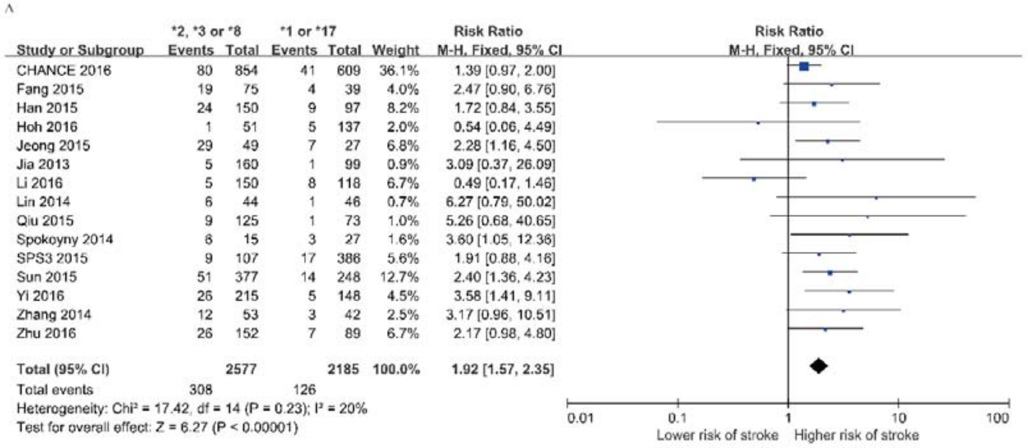

Figure 3： Risk of Stroke for Acute Ischemic Stroke or Transient Ischemic Attack Patients with Any Copy of CYP2C19 *2, *3 or *8 to wild-type (*1) or *17. Circulation.2017; 135 (1):21-33.

3. Genotyping-guided antiplatelet therapy has become a new focus in international research in the era of precision medicine

How to identify the most suitable patients and personalize the choose of antiplatelet agent is extremely challenging. Many researchers have tried to guide antiplatelet therapy based on the results of in vitro platelet reactivity tests, but the results are not satisfactory. A clinical trial in France found that vasodilator-stimulated phosphoprotein (VASP)-guided loading dose clopidogrel could lower the risk of stent thrombosis at 30 days.[11] But platelet function test-guided clopidogrel use was not associated with reduced cardiovascular or cerebrovascular events after acute coronary syndrome (ACS) according to the GRAVITAS (Gauging Responsiveness with A VerifyNow assay—Impact on Thrombosis And Safety) study[12], the ARCTIC(The Assessment by a Double Randomization of a Conventional Antiplatelet Strategy versus a Monitoring-guided Strategy for Drug-Eluting Stent Implantation and of Treatment Interruption versus Continuation One Year after Stenting) study[13], and the ANTARCTIC (Tailored Antiplatelet Therapy Versus Recommended Dose of Prasugrel) study[14]. According to the CREATIVE (Clopidogrel response evaluation and antiplatelet intervention in high thrombotic risk PCI patients) trial, in patients with low responsiveness to clopidogrel, as measured by thromboelastography, the intensified antiplatelet strategies (triple antiplatelet therapy) with adjunctive use of cilostazol significantly improved the clinical outcomes after percutaneous coronary intervention (PCI).[15] However, triple antiplatelet therapy increases the risk of bleeding in stroke patients, and this strategy could not be extrapolate to stroke patients. The application prospects of guiding drug therapy based on platelet reactivity in ischemic stroke are not optimistic. Researchers have turned their attention to the effectiveness and feasibility of tailoring antiplatelet therapy based on genotyping.

With the rapid development of point-of-care genetic testing technology, genotyping guides

antiplatelet therapy trials to be continuously optimized. According to the ELEVATE-TIMI 56 (Escalating clopidogrel by involving a genetic strategy - thrombolysis in myocardial infarction 56) study[16] and the RAPID GENE study[17], rapid genetic test coupled with subsequent personalized treatment reduces the on-treatment platelet reactivity compare to traditional antiplatelet therapy among *CYP2C19* LOF carriers after PCI. In a Chinese study randomized 623 patients after PCI, combined therapy of clopidogrel and aspirin in intermediate metabolizers and triple antiplatelets in poor metabolizers could effectively reduce the 6-month risk of MI, stroke and vascular death.[18] The ongoing TAILOR-PCI and POPular studies will also provide more clinical evidence for genotyping to guide antiplatelet therapy in cardiovascular patients. The PHARMCLO study showed that high-risk acute coronary syndrome patients benefit more from the application of precision therapy, and rapid point-of-care genetic testing (POCT) has a promising prospect for precision therapy. Although the trial was terminated early, it can still prove that precision therapy improves prognosis. Up to now, there is still a lack of similar research in the field of ischemic cerebrovascular disease. For carriers of *CYP2C19* loss-of-function alleles, whether increasing the dose of clopidogrel or switching to a new P2Y12 receptor antagonist is better remains unclear. Relevant research evidence is urgently needed.

4. Intensive antiplatelet therapy is expected to become a new option for patients with non-disabling ischemic cerebrovascular disease

Results of the ELEVATE-TIMI 56 trial indicated that elevated dose of clopidogrel intensified the platelet inhibition but did not reduce the risk of cardiac ischemic events. Moreover, for *CYP2C19*2* homozygotes, doses as high as 300 mg daily did not result in comparable degrees of platelet inhibition.[16] Ticagrelor is a reversibly binding, potent, oral adenosine diphosphate

(ADP) P2Y12 receptor blocker.[19] The metabolism of ticagrelor does not involve CYP450 enzyme, thus not influenced by carrier status of *CYP2C19* LOF allele.[20]

According to the PLATO (platelet inhibition and patient outcomes) study, ticagrelor was superior to clopidogrel in reducing the rate of the composite efficacy endpoint of Cardiovascular (CV) death, myocardial infarction (MI), and stroke after ACS events within 12 months (9.8% vs.11.7%, HR 0.84, 95%CI 0.77-0.92, P< 0.001).[21] And among patients with a history of stroke or TIA, the reduction of the total mortality at one year with ticagrelor vs. clopidogrel was consistent with the overall trial results and with a trend of even greater reduction.[22] In the genetic sub analysis of the PLATO trial, ticagrelor is a more efficacious treatment for ACS than is clopidogrel, irrespective of *CYP2C19* polymorphisms.[23] Ticagrelor compared with clopidogrel was associated with similar total major bleeding[24, 25] but increased non-coronary artery bypass bridging (CABG) and non-procedure-related major bleeding. This increased risk of spontaneous hemorrhage was seen primarily after a more than 30 days of dual antiplatelet treatment. And the increased risk of spontaneous hemorrhage could be partly attributed to higher dose of aspirin. Therefore, the treatment regimen of the PLATO trial is not suitable for stroke patients.

The SOCRATES trial is a superiority research comparing the efficacy of ticagrelor and aspirin in patients with TIA or MIS. Although the expected results were not achieved[24], it provide sufficient evidence of the superiority of ticagrelor comparing to aspirin in reduce subsequent risk of major vascular events in acute-phase high-risk patients.[25] Noticably, in the subgroup analysis of Asian patients in the SOCRATES trial, there was a trend toward a lower hazard ratio in reducing risk of the primary end point of stroke, MI, or death in the ticagrelor group (ticagrelor: 9.6％ vs. aspirin: 11.6％， HR 0.81, 95% CI 0.67～0.99， *P* = 0.04）[26]On the other hand, the study also confirmed the safety of ticagrelor in patients with ischemic stroke.

Regardless of the overall population or the Asian population, the bleeding risk in ticagrelor group is similar to that of aspirin group (P = 0.45 in the overall population and in the Asian population). The SOCRATES and PLATO indicated that in patients with non-disabling ischemic cerebrovascular disease with a high risk of recurrence, especially for Asian patients, the combination of ticagrelor and aspirin antiplatelet therapy may be a more effective treatment option.

The Prince trial (Platelet Reactivity in Acute Non-disabling Cerebrovascular Events, NCT02506140), a proof-of-concept randomized clinical trial, intended to test the difference of efficacy and safety between ticagrelor plus aspirin and clopidogrel and aspirin in reducing 90-day stroke recurrence after TIA or MIS when treatment initiated within 24 hours after symptom onset (Figure 4), particularly in carriers of the *CYP2C19* LOF allele. Patients with TIA or MIS who are treated with ticagrelor plus aspirin have a lower proportion of high platelet reactivity than those who are treated with clopidogrel plus aspirin (Figure 5). There was a trend toward lower risk of stroke recurrence (Figure 6). No difference was seen in the rates of major hemorrhagic events between the two groups.

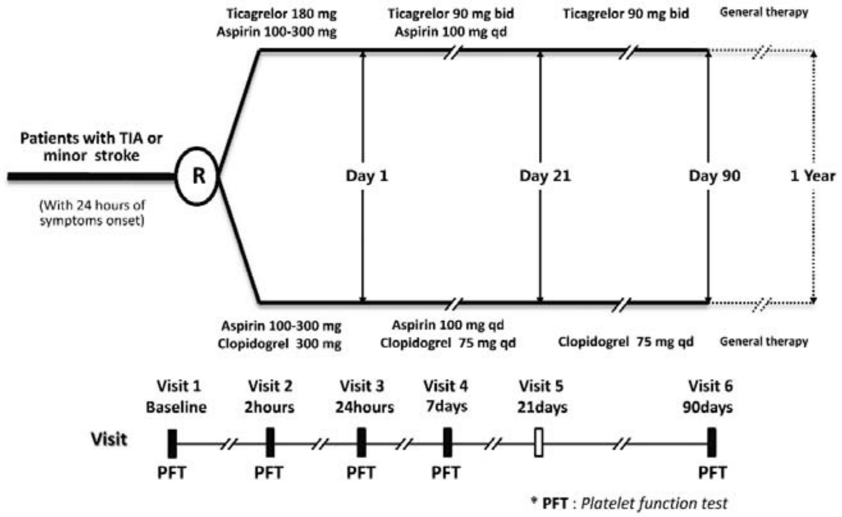

Figure 4: Flowchart of the PRINCE study. Platelet Reactivity in Acute Non-disabling Cerebrovascular Events (PRINCE) is designed as a prospective, multi-center, randomized, open-label, active-controlled, and blind-endpoint, phase II b trial. (NCT02506140).

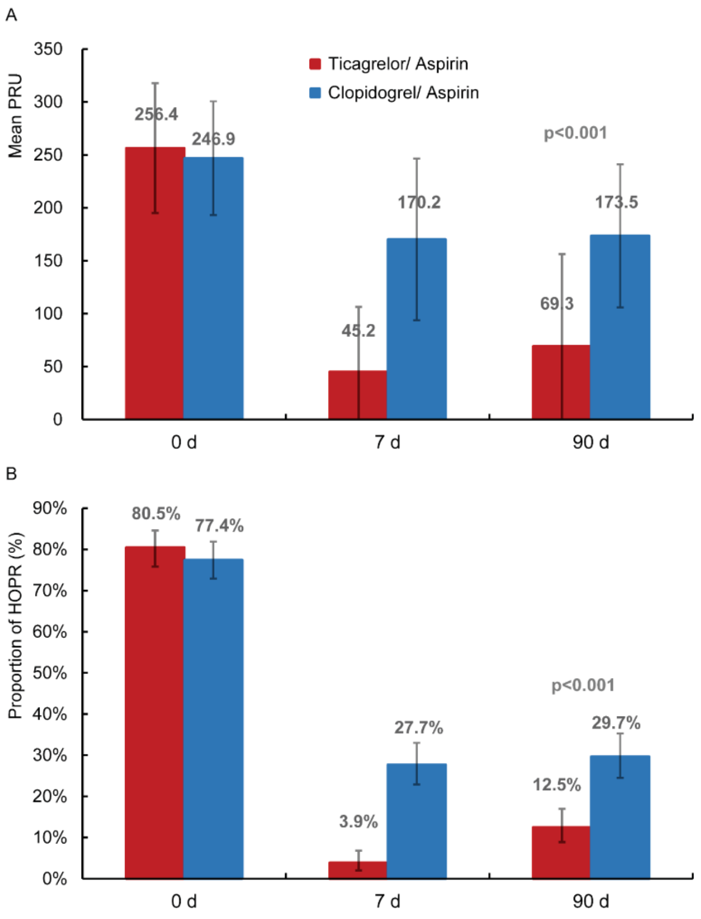

Figure 5: Platelet reactivity at different visits during 90 days. (a) P2Y12 reaction units; (b) proportion of patients with high platelet reactivity.

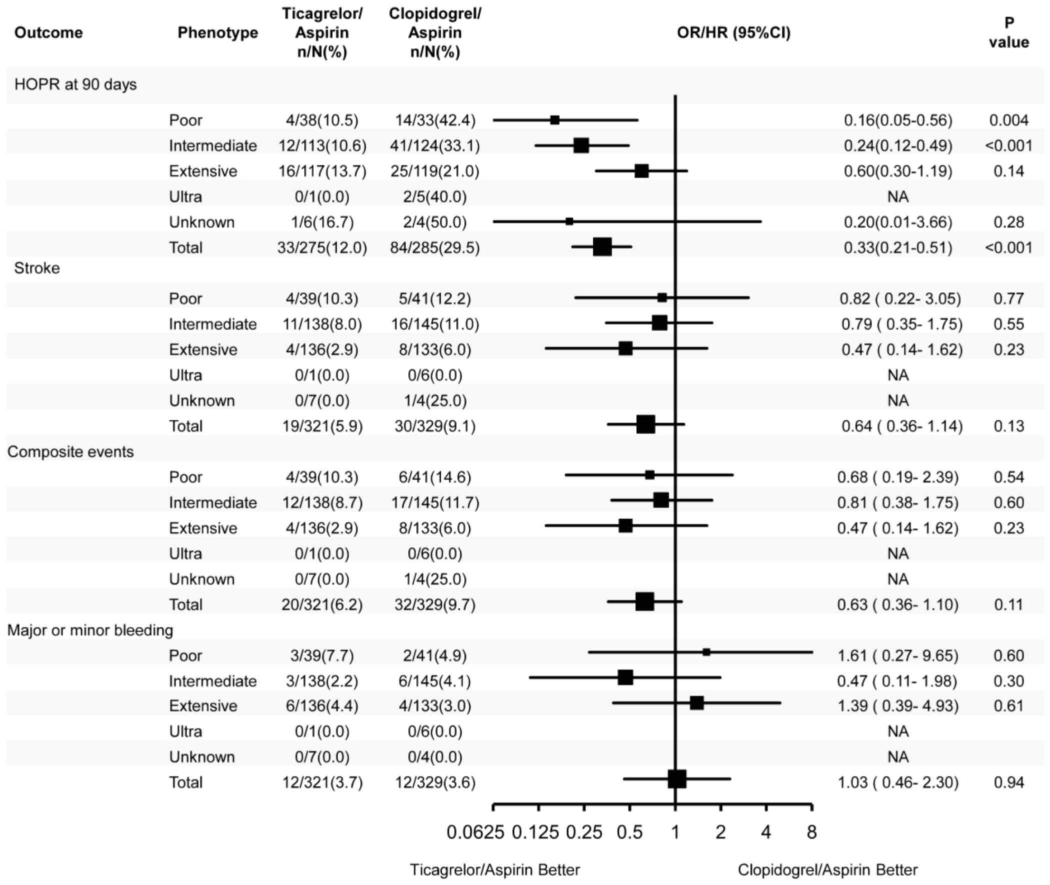

Figure 6: Effect of ticagrelor/aspirin as compared with clopidogrel/aspirin on 90-day high platelet reactivity and clinical outcome stratified by metabolizer status.

5. New hypothesis and new strategy

Based on the prior evidence, we hypothesize that pharmacogenetic test-guided antiplatelet treatment is superior to current guideline-recommended strategy. Therefore, we plan to carry out this prospective, multi-center, double-blind, randomized controlled clinical study to compare the effects of ticagrelor plus aspirin versus clopidogrel plus aspirin on reducing the 3-month risk of any stroke (ischemic or hemorrhagic, primary outcome) when initiated within 24 hours of symptom onset in *CYP2C19* LOF alleles carriers with TIA or minor ischemic stroke (MIS) with the guide of point-of-care *CYP2C19* genotyping. And we aim to provide diagnosis and treatment evidence for individualized antiplatelet therapy.

II. Study purpose

2.1 Primary objective

To assess the effects of ticagrelor plus aspirin versus clopidogrel plus aspirin on reducing the 3-month risk of any new stroke (ischemic or hemorrhagic, primary outcome) when initiated within 24 hours of symptom onset in *CYP2C19* LOF alleles carriers with TIA or minor ischemic stroke (MIS).

2.2 Secondary objectives

2.2.1 To assess the different rate of composite vascular outcome: composite of any stroke, TIA, myocardial infarction, and vascular death at 3-month and at one-year;

2.2.2 To assess separately the effects of this ticagrelor plus aspirin regemin versus clopidogrel plus aspirin regimen on the incidence of: ischemic stroke, hemorrhagic stroke, TIA, MI, vascular death, death of all-cause, disability (mRS: 2-6) at 3-month and at one-year;

2.2.3 To compare the safety of the two treatment regimens in terms of:

- Severe or moderate bleeding（GUSTO definition）
- Bleeding events
- Total mortality
- Intracranial hemorrhage (symptomatic and asymptomatic)
- Adverse events/ Severe adverse events

2.2.4 Subgroup analyses:

- Compare the main efficacy endpoints and safety of the two treatment options in different etiology subtype;
- Compare the effectiveness and safety of the two treatment options in the stratification of different gender (male vs. female) and age (<65 years old vs. ≥65 years old);
- Compare the effectiveness and safety of the two treatment options in diabetic and non-diabetic patients;

- Compare the effectiveness and safety of the two treatment options in different target events (TIA vs. mild stroke);
- For patients with pre-onset anti-platelet therapy (those who took aspirin and/or clopidogrel within 1 month before the onset vs. those who did not take aspirin and/or clopidogrel), compare the effectiveness and safety of the two treatment ;
- Compare the effectiveness and safety of the two treatment options according to the time from the onset to the first adminstration (within 18 hours vs. 18-24 hours)

2.2.5 To evaluate neurological impairment (change in NIHSS scores), and Quality of Life (EuroQol EQ-5D scale) among survivors

2.2.6 To evaluate the health economics indicators of the two treatment regimens

2.28 Consistency analysis and performance evaluation of different rapid genotyping instruments

III. Study design

3.1. Study design

This is a multicenter, prospective, double-blind, placebo-controlled, randomized clinical trial. To assess the effects of ticagrelor plus aspirin versus clopidogrel plus aspirin on reducing the 3-month risk of any stroke (both ischemic and hemorrhagic, primary outcome) when initiated within 24 hours of symptom onset in *CYP2C19* LOF alleles carriers with TIA or minor ischemic stroke (MIS).

3.1.1 Ticagrelor plus aspirin group**:**

- Day 1: two tablets of ticagrelor 90 mg, four tablets of placebo clopidogrel 75mg, and open label ASA (75mg – 300mg)
- From D2 to D21±2 days: one tablet of ticagrelor 90mg twice per day, one tablet of placebo clopidogrel 75mg per day, and open label ASA (75mg) per day

- From D22 to D90±7 days: one tablet of ticagrelor 90mg twice per day and one tablet of placebo clopidogrel (75mg) per day

The first administration was within 24 hours of the onset of symptoms.

3.1.2 Clopidogrel plus aspirin group:

- Day 1: four tablets of clopidogrel 75mg, two tablets of placebo ticagrelor 90 mg, and open label ASA (75mg – 300mg)
- From D2 to D21±2 days: one tablet of clopidogrel 75mg per day, one tablet of placebo ticagrelor 90mg twice per day, and open label ASA (75mg) per day
- From D22 to D90±7 days: one tablet of clopidogrel 75mg per day, and one tablet of placebo ticagrelor (90mg) twice per day

The first administration was within 24 hours of the onset of symptoms.

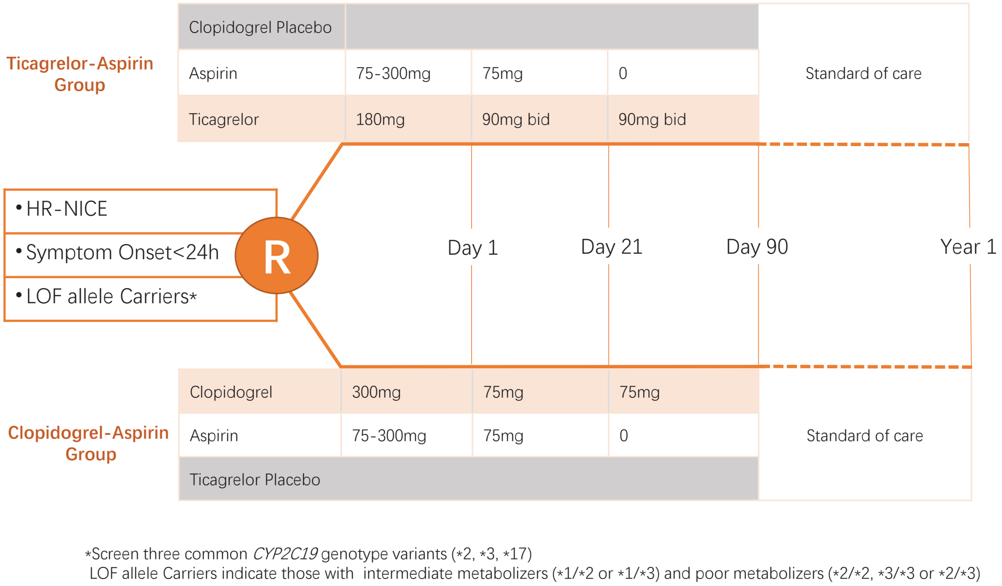

Figure 7. Study design

3.2 Follow-up schedule

Study visits will be performed on the day of randomization, at day 14/discharge, at day21±2, and at day 90±7 via face-to-face interview. Final diagnosis, etiologic subtype and

relevant evaluation or treatment will be recorded at hospital discharge visit. After the day 90±7 days visit, a long-term follow-up will be scheduled. Study visits will be performed on the day of randomization, at day 21±2, and at day 90±7 via face-to-face interview. If a patient experiences a potential clinical neurological event, including a clinical deterioration that could be possibly related to ischemia, or new transient or persistent neurological symptoms, an adjudication packet will be produced by the site within 72 hours.

3.3 Study milestones

A 4-year study plan has been created; key study milestones below.

Progress	Time interval
Complete research design, ethical review, research registration, recruitment of branch centers	8 months (2018.10-2019.06)
Recruiment and enrollment	24 months (2019.06-2021.06)
Interim analysis	Dec, 2020
Completion of 3-month follow-up*	3 months (2021.06-2021.09)
Data cleansing, analysis and publication	9 months (2021.09-2022.06)
**Total duration**	**44 months**

* According to the 3-month follow-up, long-term follow-up was not taking into account

IV. Study endpoints

4.1 Primary efficacy endpoint

Any new stroke events (ischemic stroke or hemorrhagic stroke) within 3 months

4.2 Secondary efficacy endpoints

- New clinical vascular events (any strok/ TIA/ myocardial infarction/vascular death) within 3 months; At the same time, each new vascular event were evaluated independently;
- New ischemic stroke events within 3 months;

- The percentage change of the mRS score between 0-2 points group and 3-6 points group follow-up to 3 months;
- Continuous changes in NIHSS scores at 3 months;
- Changes in EQ-5D-5L scale at 3 months;
- Any new stroke events (ischemic stroke or hemorrhagic stroke) within 1year;
- New clinical vascular events (any strok/ TIA/ myocardial infarction/vascular death) within 1 year； At the same time, each new vascular event were evaluated independently;
- New ischemic stroke events within 1 year;
- The percentage change of the mRS score between 0-2 points group and 3-6 points group follow-up to 1-year;
- Continuous changes in NIHSS scores at 1 year;
- Changes in EQ-5D-5L scale at 1 year.

At the same time, a stratified analysis of the effective endpoint events will perform: gender stratified analysis (male and female), age stratified analysis (<65 years old and ≥65 years old), etiological subgroup analysis (main subgroups type of ischemic stroke), diabetic (diabetic and non-diabetic), CYP2C19 genotype (intermediate metabolism and weak metabolism), stratified analysis of target events (TIA and mild stroke), stratified analysis of responsible blood vessels (intracranial and external arterial stenosis).

4.3 Safety endpoint

1) Primary safety endpoint

Incidence of severe bleedings or moderate bleedings (GUSTO definition) at 3 months

2) Secondary safety endpoints

- Incidence of severe bleedings or moderate bleedings (GUSTO definition) at 1 year;
- Bleeding events (severe or moderate bleedings or intracranial hemorrhage) at 3 months and 1 year;
- Mortality at 3 months and 1 year;
- Incidence of symptomatic and asymptomatic intracranial hemorrhagic events at 3 months

and 1 year;

- AEs/SAEs reported by the investigators.

V. Participant selection

During the course of the trial, about 6,396 *CYP2C19* LOF allele(s) carriers with TIA or minor ischemic stroke. Before enrolling patients into the study, all collaborating sites will obtain approval from local Institutional Review Boards (IRBs), which will have access to all study documentation and educational materials.

5.1 Inclusion Criteria

1. 40 years or older than 40 years and less than 80 years；
2. Acute cerebral ischemic event due to：

- Acute non-disabling ischemic stroke (NIHSS≤3 at the time of randomization)or，
- TIA with moderate-to-high risk of stroke recurrence (ABCD2 score ≥ 4 at the time of randomization) or responsible vessel stenosis more than 50%;

3. Can be treated with study drug within 24 hours of symptoms onset*(*Symptom onset is defined by the "last see normal" principle)；
4. Informed consent signed.

*The time of symptoms onset is defined as "the time when the last appeared normal"

5.2 Exclusion Criteria

1. Malformation, tumor, abscess or other major non-ischemic brain disease (e.g., multiple sclerosis) on baseline head CT or MRI.

2. Isolated or pure sensory symptoms (e.g., numbness), isolated visual changes, or isolated dizziness/vertigo without evidence of acute infarction on baseline head CT or MRI.

4. Preceding moderate or severe dependency (modified Rankin scale [mRS] score > 2).
5. Contraindication to clopidogrel, ticagrelor or aspirin:

1) Known allergy

2) Severe renal (creatinine exceeding 1.5 times of the upper limit of normal range) or hepatic (ALT or AST > twice the upper limit of normal range) insufficiency

3) Severe cardiac failure (NYHA level: III to IV)

5) History of hemostatic disorder or systemic bleeding

5) History of thrombocytopenia or neutropenia

6)History of drug-induced hematologic disorder or hepatic dysfunction

7) Low white blood cell (<2×10^9/L) or platelet count (<100×10^9/L)

5. Clear indication for anticoagulation (presumed cardiac source of embolus, e.g., atrial fibrillation, prosthetic cardiac valves known or suspected endocarditis)

6. Clopidogrel or ticagrelor has been used continuously for ≥5 days before enrollment.

7. Used heparin or oral anticoagulant drugs within 10 days before enrollment.

8. Undergone intravenous or arterial thrombolysis and mechanical thrombectomy within 24 hours before enrollment.

9 History of intracranial hemorrhage or amyloid angiopathy.

10. History of aneurysm (including intracranial aneurysm and peripheral aneurysm).

11. Diagnosis or suspicious diagnosis of acute coronary syndrome.

12. History of asthma or COPD (chronic obstructive pulmonary disease).

13. High-risk for bradyarrhythmia (first-degree or second-degree AV block caused by sinus node disease, and brady-arrhythmic syncope without pacemaker).

14. History of hyperuricemia nephropathy.

15. Anticipated requirement for long-term (>7 days) non-steroidal anti-inflammatory drugs (NSAIDs).

16. History of gastrointestinal bleeding within 3 months before enrollment or major surgery within 30 days.

17. Mild stroke/TIA due to angioplasty/vascular surgery.

18. Planned or likely revascularization (any angioplasty or vascular surgery) within the next 3 months.

19. Scheduled for surgery or interventional treatment requiring study drug cessation.

20. Severe non-cardiovascular comorbidity with life expectancy < 3 months.

21. Women of childbearing age who have not taken effective contraceptive measures and have a positive

pregnancy test record, as well as women who are pregnant or breastfeeding.

22. Currently receiving an experimental drug or device.

23. Participation in another clinical study with an experimental product during the last 30 days.

24. Inability to understand and/or follow research procedures due to mental, cognitive, or emotional disorders.

25. Hematocrit (HCT) <30%.

VI. Treatments

6.1 Study drugs and drug specifications

Drug	Specifications	Dosage form	Delivery way	Manufacturer	Storage method	Validity
Ticagrelor	90 mg	tablet	oral	Shenzhen Xinlitai Pharmaceutical Co., Ltd.	Below 30℃	24 months
Placebo ticagrelor	0mg	tablet	oral	Shenzhen Xinlitai Pharmaceutical Co., Ltd.	No special requirements	24 months
Clopidogrel	75 mg	tablet	oral	Shenzhen Xinlitai Pharmaceutical Co., Ltd.	No special requirements	36 months
Placebo clopidogrel	0mg	tablet	oral	Shenzhen Xinlitai Pharmaceutical Co., Ltd.	No special requirements	36 months
Asipirin	25 mg	tablet	oral	Shanghai Xinyi Bailuda Pharmaceutical Co., Ltd.	Below 25℃	60 months

6.2 Study drugs

CYP2C19 **intermediate or poor metabolizers (double blind)**

Ticagrelor plus asipirin	Day 1	two tablets of ticagrelor 90 mg, four tablets of placebo clopidogrel 75mg, and open label ASA (75mg – 300mg)
	From D2 to D21±2 days	one tablet of ticagrelor 90mg twice per day, one tablet of

		placebo clopidogrel 75mg per day, and open label ASA (75mg) per day
	From D22 to D90±7 days	one tablet of ticagrelor 90mg twice per day and one tablet of placebo clopidogrel (75mg) per day
Clopidogrel plus aspirin	Day 1	four tablets of clopidogrel 75mg, two tablets of placebo ticagrelor 90 mg, and open label ASA (75mg – 300mg)
	From D2 to D21±2 days	one tablet of clopidogrel 75mg per day, one tablet of placebo ticagrelor 90mg twice per day, and open label ASA (75mg) per day
	From D22 to D90±7 days	one tablet of clopidogrel 75mg per day, and one tablet of placebo ticagrelor (90mg) twice per day

6.3 Dose regimen

Patients are randomized 1 : 1 to ticagrelor plus aspirin or clopidogrel plus aspirin within 24 h of the onset of symptoms of *CYP2C19* LOF allele(s) carriers with high-risk TIAs, defined as an ABCD2 score ≥4 or responsible vessel stenosis more than 50%, or minor ischemic stroke, with a National Institutes of Health Stroke Scale (NIHSS) score ≤3, who can be randomized within 24 h symptoms onset. The study is conducted in a double-blind manner. The placebo for clopidogrel or ticagrelor will be identical in appearance and taste. Minor side effects are unusual with the medication, so it is not anticipated that either subjects or clinicians will be able to differentiate the placebo from the active drug.

Patients with CYP2C19 intermediate metabolism and weak metabolism genotypes were randomly divided into 2 groups within 24 hours of the onset of symptoms (both researchers and subjects were blinded):

The first group: intensive combined antiplatelet therapy group

Subjects will take the study drug as soon as possible after the genotype is determined. Ticagrelor twice a day with an interval of approximately 12 hours. If the patient took a loading dose of ticagrelor 180 mg before 2 pm on the first day of enrollment, the first maintenance dose of ticagrelor 90 mg should be taken before going to bed on the same day with at least 8 hours

between the two doses. If the patient takes the loading dose of ticagrelor 180 mg after 2 pm on the first day of enrollment, the first maintenance dose of ticagrelor 90 mg should be taken as early as possible on the morning of the second day. The total dose of aspirin (75mg-300mg) on the first day of enrollment includes: aspirin given to patients at any time and any place (including in the emergency room) on the first day of onset.

The second group: standard combined antiplatelet therapy group

Both clopidogrel and aspirin were taken once a day in the morning. The total dose of aspirin (75mg-300mg) on the first day of enrollment includes: aspirin given to patients at any time and any place (including in the emergency room) on the first day of onset.

For patients who have taken clopidogrel within 6 hours before the disease onset of symptoms, the investigator should carefully evaluate the risk of bleeding and decide whether to enter the study. For patients who have taken ticagrelor within 8 hours before the disease onset, investigators should carefully evaluate the risk of bleeding and decide whether to enroll in the study.

VII. Study conduct

7.1 Point-of-care genetic testing of *CYP2C19* genotype

*CYP2C19*2* allele (dbSNP rs4244285) is the 681G>A variant on exon 5 of *CYP2C19* gene, which is the most common loss-of-function allele. *CYP2C19*3* allele (dbSNP rs4986893) is the 636G>A variant on exon 4. Carriers of *CYP2C19*2* or **3* have decreased activity of CYP2C19 enzyme, and the activated metabolite concentration in vivo is about one-third lower than that of non-carriers. Carrying LOF allele will weaken the antiplatelet effect of clopidogrel. *CYP2C19*17* allele (dbSNP rs12248560) is a -806C>T variant on the 5'flanking region of the gene, which can specifically bind to nucleoprotein, significantly increase gene transcription level, enhance the activity of *CYP2C19* enzyme, increasing the concentration of effective

metabolite in vivo. Perform genotyping on the *2, *3 and *17 allele of the *CYP2C19* gene for all enrolled patients, and obtain the test results within 1 hour and inform the investigator to determine the patient's *CYP2C19* gene metabolizer status：① Extensive metabolizer, EM): *1/*1, without *2, *3, and *17; ② Intermediate metabolizer, IM: with one *2 or *3 allele (*1/*2 or *1/*3); ③ Poor metabolizer, PM: with at least two *2 or *3 alleles (*2/*2, *2/*3, or *3/*3); ④ Ultrarapid metabolizer, UM: carrying at least one *17 allele (*1/*17 or *17/*17), which is extremely rare in Chinese population.

In this study a novel point-of-care genetic test platform was used to identify carriers of the *CYP2C19* LOF alleles. The *CYP2C19* Genotyping implemented by the GMEX® Point-of-Care Genotyping system, which includes a portable DNA analyzer, genotyping reagents, and a buccal sample collection kit. A uniform type of PCR instrument will be used for rapid genotyping assay, and a portable fluorescent PCR instrument will be preferred for bedside rapid genotyping (POCT).Please refer to the "Instruction Manual for Point-of-care Genotyping Testing" for specific testing methods.

7.2 Randomization of the *CYP2C19* intermediate/poor metabolizers

The randomization code list will be generated centrally by a Contract Research Organization (CRO).

The patient kits will be packaged in accordance with this randomization code list. During the treatment period, the patient will receive study medication corresponding to either the ticagrelor plus ASA/clopidogrel plus ASA group using a randomization ratio of 1:1.

The treatment number will be allocated using a centralized treatment allocation system on D1 (baseline visit). Subjects will be randomly assigned to the group, from small to large, to obtain the randomized treatment number. A treatment number will then be given to the investigator, who will give the first box of the corresponding package to the patient. According to the research progress of each participating center, the goal of enrollment can be adjusted, but it needs to be confirmed by the data management commitee.

7.3 Blinding system and emergency unblinding procedure of the *CYP2C19*

intermediate/poor motabolizers

7.3.1 Description of blinding methods

This randomized double-blind study is primarily designed to compare a ticagrelor /ASA combination followed by ticagrelor alone regimen versus a clopidogrel/ASA combination followed by clopidogrel alone regimen.

The two types of ticagrelor tablets developed (90 mg active ticagrelor and placebo ticagrelor) are indistinguishable (identical in size, shape, color, appearance).

The two types of clopidogrel tablets developed (75 mg active clopidogrel and placebo clopidogrel) are indistinguishable (identical in size, shape, color, appearance).

No locally used biological test that could potentially unblind the treatment is planned in this study. Investigators will not have access to the randomization (treatment) code, except in exceptional circumstances, such as occurrence of a serious adverse event for which knowledge of the study medication would be considered essential for treating the subject.

7.3.2 Emergency unblinding procedure during the study

In case of an Adverse Event, the code may be broken only in exceptional circumstances when knowledge of the Investigational Product is essential for treating the patient. If possible, contact should be made with the Monitoring Team before breaking the code. If the physician at the investigational site believes unblinding is needed, he/she must call the monitoring committee and sponsor immediately. All the calls will be documented by the CRO. If the blind is broken, the Investigator will document the date, time of day and reason for the code break. Study drug will not be resumed afterwards.

7.4 Study drug handling

7.4.1 Supply and storage

The sponsor will supply the blinded investigational products (study drug and placebo) used in the study. All investigational drug supplies in the study will be stored in a secure, safe place, under the responsibility of the Investigator or other authorized individual, and under the conditions described on the labeling.

7.4.2 Packaging and labeling

Each patient (the CYP2C19 intermediate/poor motabolizers) will be assigned a patient kit" according

to the randomization list. According to the study periods and visits, each patient kit will consist of both the public box and the patient box that includes two boxes. Each box is composed of two parts as following:

Box 1 (D1 to D 21±2 days): 1 wallet of open label aspirin, 50 ticagrelor 90mg tablets, 26 clopidogrel 75mg tablets;

Box 2 (D21±2 days to D 90±7 days): 158 ticagrelor 90mg tablets, 79 clopidogrel 75mg tablets.

Group1 Ticagrelor plus asipirin group

Box 1: 1 wallet of open label aspirin, 50 ticagrelor 90mg tablets, 26 placebo clopidogrel 75mg tablets;

Box 2: 158 ticagrelor 90mg tablets, 79 placebo clopidogrel 75mg tablets.

Group2 Clopidogrel plus aspirin group

Box 1: 1 wallet of open label aspirin, 26 clopidogrel 75mg tablets, 50 placebo ticagrelor 90mg tablets;

Box 2: 79 clopidogrel 75mg tablets, 158 placebo ticagrelor 90mg tablets.

#Administrate twice a day and do not mix. Be sure to take the drug in Box 1 in the morning and take the drug in Box 2 at night.

Label of box:

- Abbreviation of the study: CHANCE-2
- Symbol of the study
- Randomization number
- Box 1 (D1 to D 21±2 days), Box 2 (D21±2 days to D 90±7 days)
- Names of drugs
- Form, specification, route of administration and dosage of the study drug
- Indications
- Storage conditions
- Period of validity
- Please take the medicine as prescribed by the doctor, and please take back the remaining medicine, which will be counted at the next visit

- Keep away from children to prevent children from taking it by mistake
- For clinical research use only
- Please swallow carefully to avoid suffocation

7.4.3 Responsibilities of drug handling

The Investigator, the Hospital Pharmacist, or other personnel allowed to store and dispense Investigational Product will be responsible for ensuring that the Investigational Product used in the clinical trial is securely maintained as specified by the Sponsor and in accordance with the applicable regulatory requirements.

All Investigational Product shall be dispensed in accordance with the Investigator's prescription, and it is the Investigator's responsibility to ensure that an accurate record of Investigational Product issued and returned is maintained.

Any quality issue noticed with the receipt or use of an Investigational Product (deficient IP in condition, appearance, pel1aining documentation, labeling, expiry date, etc.) should be promptly reported to the Sponsor, who will initiate a complaint procedure.

Under no circumstances will the Investigator supply Investigational Product to a third party, allows the Investigational Product to be used other than as directed by this Clinical Trial Protocol, or dispose of Investigational Product in any other manner.

7.4.4 Compliance to treatment and accountability

The administration of all medication (including study medication and concomitant medication) should be recorded in the appropriate sections of the CRF. At Day 1 (randomization), patients will receive enough study medication to cover up to 21 days visit. At 21 days visit, patients will receive enough study medication to cover up to 90 days visit. Patients will be asked to return all unused study medications and empty packages to the participating site at 21 days visit and 90 days visit. A pill count should be done at a patient level and recorded in both the CRF and a dispensing log by the study site personnel.

The study personnel will account for all study medication dispensed and returned from the patient to evaluate the study compliance.

Investigators are responsible for recording the administration of all medication (including study medication and concomitant medication) in the appropriate sections of the CRF.

Information about the time of discontinuation and restart of study drugs should be recorded.

7.4.5 Return and destruction of study drugs

The investigator (or pharmacist) should establish a detailed record of the return of the study drug, and the investigator and the monitoring team should jointly confirm the record.

Investigators cannot destruct unused or partially used study drugs unless the principal investigator provides written authorization.

If the study drug has defects in quality, the investigator will adopt a recall procedure for recall and return. At this time, in order to recall the study drug and eliminate potential hazards, the investigator is responsible for meeting any requirements put forward by the principal investigator.

7.5 **Concurrent treatment**

During the study period, any medication, surgery, vascular intervention after enrollment, and any medication changes during the study should be recorded in the case report form.

7.5.1 Prohibited concomitant treatments

1) Antiplatelet agents: Dipyridamole, Cilostazol, Ticlopidine, Prasugrel, GPIIb/IIIa receptor antagonist, Ozagrel, etc.

2) All anticoagulants

3) Thrombolytic drugs: rt-PA, urokinase and streptokinase, etc. Patients who received intravenous or arterial thrombolysis 24 hours before enrollment should be excluded. If the patient receives intravenous or intraarterial thrombolysis after enrollment, the study treatment must be discontinued for more than 24 hours before restart.

4) Batroxobin, defibrase, snake venom preparation, lumbrokinase, etc.

5) Non-steroidal anti-inflammatory drugs (Cox1 and Cox2 inhibitors). The use of non-steroidal anti-inflammatory drugs (Cox1 and Cox2 inhibitors) within 7 days after

randomization is violation to the protocol. If absolutely necessary, non-steroidal anti-inflammatory drugs should be started 24 hours after the study drug is temporarily discontinued, the use time should be less than 5 days, and the start time should not be earlier than 8 days after randomization.

6) Potent CYP3A inhibitors: ketoconazole, nefazodone, ritonavir, saquinavir, atazanavir, nelfinavir, itraconazole, voriconazole, clarithromycin, telithromycin (but Does not include erythromycin or azithromycin)。

7) Potent CYP3A inducers: rifampicin, dexamethasone, phenytoin, carbamazepine, oxcarbazepine, phenobarbital.

8) Potent *CYP2C19* inhibitors: omeprazole, esomeprazole; fluvoxamine, fluoxetine, moclobemide, fluconazole, chlorbenzylpyridine, ciprofloxacin, cimetidine, chlorine Mycin.

9) Potent P-glycoprotein (P-gp) inhibitors: verapamil, quinidine, cyclosporine.

10) P-glycoprotein substrate: digoxin. If digoxin is absolutely necessary, the dose of digoxin should be reduced as appropriate, and the blood concentration of digoxin should be closely monitored.

7.5.2 Permitted concomitant treatments

Any drugs other than those listed above are permitted. Patients with underlying diseases (hypertension, diabetes, coronary heart disease, epilepsy, etc.) can use diuretics, beta blockers, ACEIs, ARBs, calcium antagonists, lipid-lowering medications, coronary vasodilators, and anti-diabetic medication (including Insulin), anti-epileptics, etc. Information about diagnosis, medication, dose and administration should be collected. Any treatment with necessity (concomitant diseases during the study period) must be recorded in the CRF, including information about diagnosis, medication, dose and administration, etc.

1) After randomisation, patients who develop an indication (ACS or Percutaneous coronary intervention (PCI)) for dual antiplatelet therapy must discontinue study therapy and be treated

with standard of care. Detailed information about the adjustment of treatment should be recorded in the CRF.

2) H2 receptor blockers are permitted except cimetidin.

3) Proton pump inhibitors (PPIs): If necessary, PPIS can not be used except omeprazole and esomeprazole. Rabeprazole is recommended. Other PPIs can be used, such as dexlansoprazole, lansoprazole and pantoprazole.

4) Statin: doses of simvastatin or lovastatin ≤40 mg daily or any dose of any other statin is permitted

5) Drugs that can induce bradycardia: such as beta blockers, calcium antagonists, etc., when combined with ticagrelor, heart rate should be closely monitored.

6) Serotonin reuptake inhibitors (SSRIs): such as paroxetine, sertraline, and citalopram, which may increase the risk of bleeding. If the concomitant use of SSRIs is inevitable, bleeding events should be carefully monitored.

7.5.3 Surgery and invasive procedure

If intervention (surgery or invasive procedure including vascular surgery, coronary artery angioplasty/angiography, internal carotid/external carotid artery angioplasty/angiography) with high bleeding risk is absolutely necessary within the three months period after randomization, study drug will be stopped 5 days prior to the intervention. After surgery, study medications should be restarted when the risk of bleeding is deemed low in the judgment of the investigator. Other surgery or invasive procedure, whether discontinue the study drug(s) is up to the judgement of investigator.

7.6 Temporary, permanent discontinuation of the study drug(s) or loss of follow-up

The study drug(s) should be continued whenever possible. If the study drug(s) are stopped, it should be determined if the discontinuation can be made temporarily; permanent study drug(s) discontinuation should be a last resort. Any study drug(s) discontinuation should be fully documented in the CRF. Discontinuation of study medication does not mean discontinuation

of follow-up or termination of study participation.In any case, the patient should remain in the study as long as possible.

7.6.1 Temporary treatment discontinuation with the study drug(s)

If the study drug must be temporarily discontinued, it should be re-started as soon as possible:

Including the following conditions:

1) Severe thrombocytopenia (platelet count <50,000/uL). Patients may restart study

medication once the severe thrombocytopenia resolves;

2) Severe bleeding (GUSTO definition);
3) Need of treatment with prohibited concomitant medications, see Section 7.5;
4) Surgery or procedures associated with major haemorrhage, see Section 7.5.3; For other surgery or other invasive procedures, study medication may be continued or interrupted temporarily at the discretion of the investigator.

Re-initiation of treatment with the Investigational Product will be done under close and appropriate clinical/and or laboratory monitoring once the Investigator has considered according to his/her best medical judgment that the role of the Investigational Product(s) in the occurrence of the event concerned was unlikely and there is no other contraindication to continuing in the study.

7.6.2 Permanently discontinuing study medication

1) Patient decision. The patient is at any time free to discontinue treatment, without prejudice to further treatment

2) Investigator’s decision, including but not limited to these examples:

① Pregnancy or desire to become pregnant;

② Incorrectly enrolled patient in whom the inclusion/exclusion criteria

violation would put the patient at undue risk;

③ Severe non-compliance to study protocol;

④ Adverse event for which the investigator thinks continued treatment may put

the patient at undue risk;

⑤ Clear indication for anticoagulation;

⑥ After temporarily discontinuation of the study drugs, the investigator judged that the patient should not restart the study drugs.

7.6.3 Handling of patients after definitive treatment discontinuation

Patients permanently discontinuing study medication should be given conventional therapy. A patient that decides to discontinue study medication will always be asked about the reason(s) for their desire to discontinue study medication and the presence of (if any) adverse events. These data will be ascertained and documented by the Investigator and recorded in the CRF. Adverse events will be followed up; and the patient should return all study drugs.

All patients who have discontinued the study drug prior to the last visit should have a complete end-of-study visit at three months or up to recovery or stabilization of an AE, which ever comes last. All withdrawls should be recorded by the investigator on the appropriate CRF pages when considered to be confirmed.

7.6.4 Loss to follow-up

The investigator should make every effort to contact the patient and to identify the reason why he/she failed to attend the visit and to determine his/her health status. For patients considered lost to follow-up, the CRF must be completed up to the last visit performed.

7.7 Consequence of withdrawal

Patients who have been withdrawn from the study cannot be included again in the study. Their patient number and treatment must not be re-used.

The investigator will call the monitoring team to notify the treatment discontinuation and/or the patient's withdrawal.

VIII. Biological sample collection

The collected blood sample is specifically required to be sent to the relevant laboratory. Samples of serum, plasma, and white blood cells (see the Biological Sample Collection and

Processing Manual for details), and then transport them via cold-chain transportation to Beijing Tiantan Hospital for centralized storage and data analysis. The time of collecting blood samples should be recorded in detail on the blood sample information page.

IX. Data collection

Refer to the manual of procedures and data collection guidelines, investigators should guarantee the entry of CRF is precise, complete and timely, and answer the queries in time. Brain MRI includes: T1+T2+DWI+FLAIR+ADC+GRE- T2*/SWI+MRA, collected as DICOM format. Vascular evaluation, transthoracic echocardiography, 24-hour Holter and laboratory results will be collected by photocopy of reports or in DICOM format.

X. Study procedures

Investigator(s) should keep a record, the Eligibility CRF, of subjects who entered prestudy screening. The sub-center number must be indicated. The screening table will be used to analyze and determine whether the enrolled patients in different study sites are representative.

10.1 Screening and inclusion

- Medical history, history of medication uses and physical examination;
- ABCD2 score for TIA patients and neurological evaluation (Modified Rankin Scale and NIHSS) will be performed for screening subjects;
- Perform emergency laboratory examinations: emergency blood routine, emergency renal function, emergency liver function (transaminase), emergency coagulation, random blood glucose, etc.;
- An electrocardiogram (ECG) will be performed (12-lead) to rule out atrial fibrillation, sick sinus syndrome, second or third degree atrioventricular block;
- A head CT or MRI scan will be required to rule out hemorrhage, vascular malformation, tumor, or abscess;

(Since ECG and brain imaging are routinely recommended for all patients with ischemic stroke and TIA, this study will not provide these costs)

- Women of childbearing age need to take a urine pregnancy test to exclude pregnancy;
- Orally inform patients of complete research-related information, and give corresponding written materials;
- Patients who meet the inclusion criteria but not the exclusion criteria need to undergo rapid *CYP2C19* genotyping: Subjects with CYP2C19 normal metabolism genotype will be given the patients' open-label aspirin and clopidogrel; Subjects with CYP2C19 intermediate metabolism and poor metabolism genotypes sign the informed consent to join the study. All subjects need to sign a written informed consent;
- Subjects will get the number of enrollment

10.2 Day of randomization (Day 1)

10.2.1 Baseline evaluation

- Baseline demographic characteristics;
- Symptoms of the index event, clinical course, past medical history, medications, cigarette and alcohol use, family history;
- Measurement of height, weight, waistline, physical examination, NIHSS at screening, $ABCD^2$, prestroke Rankin Score examination findings.

10.2.2 Collection of biological sample

- The first fasting peripheral venous blood samples collected by all subjects on the day they were admitted to the group must be processed in accordance with the requirements of the operation manual , and the serum, plasma, and white blood cells should be packed separately and transported to Tian Tan Hospital.

10.2.3 Study drug handling

- Public box including the open-label aspirin and patient box 1 (Day 1 to Day 21±2) according to treatment number
- Study drug will be started as soon as possible after randomzation (within **one hour** after completing the point-of-care *CYP2C19* genotyping)
- The investigator instructs the patient to take the first dose of the study drug:

  1. Total dose on D1 includes any ASA treatment taken by the patient, or given in the ambulence, the emergency unit, or after admission on the same day.

2. If ticagrelor is taken for the first time before 2 pm on the same day, a maintenance dose is required that night.

10.2.4 Schedule of auxilliary tests

- Schedule brain MRI for subjects (Include: T1+T2+DWI+FLAIR+ADC+GRE- T2*/SWI+MRA, completed within 72 hours after randomization; and 24h holter.
- Schedule vascular evaluation (completed before discharge)

10.3 Record of hospital discharge visit

- Information will be collected on the drug accountability;
- Information will be collected on concomitant medications;
- Information will be collected on new stroke or vascular events since the last visit;
- Information will be collected on adverse events or bleeding events since the last visit;
- Confirm that the brain imaging examination and vascular assessment have been completed;
- Raw data of auxiliary tests will be collected (photocopy of reports or imaging data of DICOM format);
- Record the final diagnosis and etiologic subtype according to the ASCO classification of the qualifying event;
- Record the discharge diagnosis of the subject;
- An appointment will be made for next study visit (21±2 days after randomization, face-to-face interview)

Inform the patients not to discard the pill box, empty medicine board and all wrapping paper, and they need to bring it to the hospital during the next follow-up visit

10.4 Day 21±2 visit

- A physical examination will be performed, including measurement of weight (kg) and vital signs (supine systolic and diastolic blood pressure, heart rate) and neurological examination;
- A neurological evaluation will be performed (Modified Rankin Scale and NIHSS);
- Schedule brain MRI for subjects, and all series should be performed (T1+T2+DWI+FLAIR+ADC+GRE- T2*/SWI+MRA)
- Treatment Boxes (public box of open-label aspirin and treatment box 1) with unused study medication from completed study period (with Day 1 and Day 2 to Day 21±2 boxes) will be collected for drug

accountability and assessment of treatment compliance;

Attention: Carefully count the number of unused pills and record

- Treatment Box 2 will be dispensed to the patient and he will be instructed how to take the study drug until next visit;

Inform the patients not to discard the pill box, empty medicine board and all wrapping paper, and they need to bring it to the hospital during the next follow-up visit

- Information will be collected on the drug accountability and compliance;
- Information will be collected on **concomitant medications**;
- Information will be collected on new stroke or vascular events since the last visit;
- Information will be collected on adverse events or bleeding events since the last visit;
- An appointment will be made for next study visit (90±7 days after randomization, face-to-face interview)

10.5 Day 90±7 visit

- A physical examination will be performed, including measurement of weight (kg) and vital signs (supine systolic and diastolic blood pressure, heart rate) and neurological examination;
- A neurological evaluation will be performed (Modified Rankin Scale and NIHSS);
- Treatment Boxes (treatment box 2) with unused study medication from completed study period will be collected for drug accountability and assessment of treatment compliance;

Attention: Carefully count the number of unused pills and record

- Information will be collected on the drug accountability and compliance;
- Information will be collected on **concomitant medications**;
- Information will be collected on new stroke or vascular events since the last visit;
- Information will be collected on adverse events or bleeding events since the last visit;
- Quality of life scale (EQ-5D-5L) will be performed;
- Fill in the research summary;
- The subject and the investigator jointly decide which antiplatelet drugs should be used after the study;
- An appointment will be made for next long-term study visit (12 months ± 15 days after randomization, telephone interview)

10.6 12 months ± 15 days visit

- A neurological evaluation will be performed (Modified Rankin Scale and NIHSS);
- Information will be collected on concomitant medications;
- Information will be collected on new stroke or vascular events since the last visit;
- Information will be collected on adverse events or bleeding events since the last visit;
- Quality of life scale (EQ-5D-5L) will be performed;
- Fill in the research summary.

10.7 Possible event visit

If a patient experiences a potential clinical neurological event, including a clinical deterioration that could be possibly related to ischemia, or new transient or persistent neurological symptoms, an adjudication packet will be produced by the site within 72 hours. This will include the following:

- A physical examination will be performed, including measurement of weight (kg) and vital signs (supine systolic and diastolic blood pressure, heart rate) and neurological examination;
- A neurological evaluation will be performed (Modified Rankin Scale and NIHSS);
- Brain CT or MRI (including T1 + T2 + DWI + FlAIR + ADC + GRE-T2*); imaging data of DICOM format will be transferred to the core imaging evaluation lab. Brain CT is recommended for hemorrhagic stroke and MRI is recommended for ischemic stroke;
- If a patient experiences a new potential cardiac event, a cardiac evaluation (including ECG, myocardial enzymology and TnI) will be performed as clinically indicated. Information supporting a possible myocardial infarction will be collected in an adjudication packet and transmitted to Beijing Tiantan Hospital for further adjudication within 72 hours;
- Information will be collected on concomitant medications and adverse events since the last visit

10.8 Schedule of activities and assessments

Measurements	Screening	Treatment period					
		Randomization Day 1	Discharge	21 days visit D 21±2 days	90 days visit D 90±7 days	Final visit M 12±15 days	Event visit
Demographic characteristics		√					
ABCD^2 score (TIA only)	√						
NIHSS	√		√	√	√	√	√
mRS	√		√	√	√	√	√
Physical examination		√	√	√	√	√	√
Focused medical history		√					
Current medications prior to randomisation		√					
Brain CT/MRI	√						
Laboratory tests	√[1]		√[2]				
ECG		√					√
Inclusion/Exclusion	√						
Informed consent signed	√						
Point-of-care genotyping	√						
Dispense and return of study medication		√		√	√		
Blood specimens (fasting)		√					
Standardized brain MRI		√[3]		√			√
Etiologic diagnosis			√[4]				√
EQ-5D-5L					√	√	√
AE/SAEs			√	√	√	√	√
Compliance/drug accountability			√	√	√		
Concomitant medication			√	√	√	√	√

The cost of screening and baseline auxiliary examination will not be included in the budget of this study.

1. Laboratory tests during screening must be completed: emergency blood routine, emergency liver function (serum transaminase), emergency renal function (serum creatinine), emergency coagulation, and urine HCG test for women of childbearing age. The laboratory test results after the onset time can be used as the screening test results.

2. Laboratory examinations during the treatment period should be completed within 7 days after enrollment (including a full set of biochemistry (including liver function, blood lipids, fasting blood glucose, renal function, uric acid, lactate dehydrogenase, creatine kinase, blood electrolytes), glycosylated hemoglobin, homologous Cystine, urine routine, etc.). The laboratory test results after the onset time can be used as the screening test results.

3. Include: T1+T2+DWI+FLAIR+ADC+GRE- T2*/SWI+MRA, completed before discharge, collected as DICOM format.

4.All enrolled subjects should undergo vascular assessment within 7 days after enrollment. And investigators need to perform stroke etiology classification according to the ASCO classification.

XI. Study risk pre-assessment and risk management

Clinical safety will be evaluated , as follows：

- Physical examinations on day 1, 21±2, and 90±7 days, including supine blood pressure, heart rate and neurological examination;
- Collect information on adverse events and bleeding events at each visit after the baseline visit;
- When the clinical endpoint events or suspected clinical endpoint events happened, a follow-up visit should be conducted in time according to Chapter 10.7. Study site should submit data of the event for interpretation and adjudication within 72 hours of the event;
- This study does not require other special laboratory tests. However, if the abnormal laboratory test results are related to the study drug, the recording, reporting and handling of adverse events shall be carried out in accordance with the following requirements.

11.1 Adverse events monitoring

All events, whether reported by subjects or investigators, or via physical examination or laboratory results, will be managed and collected in compliance with all applicable regulations and will be included in the final Clinical Study Report (CSR).

11.2 Definitions of adverse events

11.2.1 Adverse event (AE)

Adverse Events, regardless of the relationship to Investigational Product, spanning the time from the first visit planned in the Clinical Trial Protocol/signature of the informed consent (i.e., occurring during the washout period) to the last visit planned in the protocol, are adverse medical events or deterioration of qualifying event. AEs included symptoms (ie, nausea, chest pain), signs (ie, tachycardia, liver enlargement) or abnormal laboratory results (ie, laboratory

or ECsG abnormalities). AE includes serious and non-serious AE.

11.2.2 Serious adverse event (SAE)

A serious adverse event is any untoward medical occurrence that at any dose:

- Results in death, or
- Is life-threatening, or

  Note: The term "life-threatening" in the definition of "serious" refers to an event in which the patient was at risk of death at the time of the event; it does not refer to an event which hypothetically might have caused death if it were more severe.

- Requires inpatient hospitalization or prolongation of existing hospitalization, or
- Results in persistent or significant disability/incapacity, or
- Is a congenital anomaly/birth defect, or
- Is a medically important event

11.3 Recording of adverse event

Only non-serious AEs (ie, bleeding events, dyspnea, asthma, renal impairment/increased blood creatinine, bradycardia, increased liver function test indicators, gout/increased uric acid, pneumonia, gynecomastia, abnormal uterine bleeding, malignant tumors) and discontinuations due to adverse events (DAEs) will be collected from time of randomization throughout the treatment period and including the follow-up period, until the Study Closure Visit. Other non-serious AEs are up to the investigator to decide whether to collect.

SAE: All SAEs are being recorded and collected.

11.4 Causal relationship between adverse event and study drugs：

Attribution of ：①Definite；②Probably；③Possibly；④Unlikely；⑤Not related；⑥Not accessible. Definitions are as follows：

① **Definite：** Causal relationship is certain (i.e., the temporal relationship between treatment exposure and the adverse event onset/course/resolution is clinically reasonable; other causes have been eliminated; and

the event must be definitive pharmacologically or phenomenological). The adverse reaction is alleviated or disappeared after the reduction of drug dose or discontinuation of the drug. The reaction reappears when the drug is re-initiated.

② **Probably：** High degree of certainty for causal relationship (i.e., the temporal relationship between treatment exposure and the adverse event onset/course/resolution is clinically reasonable and other causes have been eliminated or are unlikely). The adverse reaction is alleviated or disappeared after the reduction of drug dose or discontinuation of the drug. The subject's clinical status or other reasons could cause this reaction.。

③ **Possibly：** Causal relationship is uncertain (i.e., the temporal relationship between treatment exposure and the adverse event onset/course/resolution is clinically reasonable or unknown; and while other potential causes may or may not exist, a causal relationship to the study treatment does not appear probable). The adverse reaction is alleviated or disappeared after the reduction of drug dose or discontinuation of the drug. The subject's clinical status or other reasons could possibly cause this reaction.

④ **Unlikely：** Not reasonably related, although a causal relationship cannot be ruled out (i.e., while the temporal relationship between treatment exposure and the adverse event onset/course does not preclude causality, there is a clear alternate cause that is more likely to have caused the adverse event than the study treatment). The subject's clinical status or other reasons could possibly cause this reaction.

⑤ **Not related：** No possible relationship (i.e., the temporal relationship between treatment exposure and the adverse event onset/course is unreasonable or incompatible; or a causal relationship to study treatment is implausible). The subject's clinical status or other reasons could possibly cause this reaction. After clinical symptoms or other causes resolved, the reaction is alleviated or disappeared.

⑥ **Not accessible**

Total rate of AE was calculated by summarizing the incidence of adverse drug events in each case report form ①+②+③.

11.5 Safety

11.5.1Adverse events

All Adverse Events regardless of seriousness or relationship to Investigational Product, spanning the time from the first visit planned in the Clinical Trial Protocol/signature of the informed consent (i.e., occurring during the washout period) to the last visit planned in the protocol, are to be recorded on the corresponding page(s) included in the Case Report Form. Whenever possible, symptoms should be grouped as a single syndrome or diagnosis. The Investigator should specify the date of onset, intensity, action taken with respect to Investigational Product, corrective treatment/therapy given, outcome and his/her opinion as to whether there is a reasonable possibility that the Adverse Event was caused by the Investigational Product.

Laboratory, vital signs or ECG abnormalities are to be recorded as Adverse Events only if they are medically relevant: symptomatic, requiring corrective treatment, leading to discontinuation and/or fulfilling a seriousness criterion.

11.5.2 Serious adverse event

For serious adverse events, the investigator must immediately take corresponding measures:

Immediately notify the representative of the Monitoring Team, and send the signed and dated corresponding page of the case report form to the representative of the Monitoring Team, and attach a photocopy of all examinations carried out and the dates on which these examinations were performed. Care should be taken to ensure that the patient's identity is protected and the patient's identifiers in the Clinical Trial are properly noted on any copy of source documents provided to the Sponsor. For laboratory results, include the laboratory normal ranges.

These measures should be completed no later than **24 hours** after SAE. Send the signed and dated corresponding page(s) in the Case Report Form to the representative of the Monitoring Team whose name, address and fax number appear on the Clinical Trial Protocol.

11.5.3 Follow-up and risk management

The Investigator should take all appropriate measures to ensure the safety of the patients.

Screening of subjects should strictly follow the inclusion and exclusion criteria of the study. When adverse event happens to subject during the study, medication could be temporarily discontinued or withdrawn. Relevant evaluations include blood routine examination, coagulation, creatinine, hepatic function, renal function, arterial blood gas analysis, ultrasound and computer tomography. Targeted treatment and necessary consultant should be taken timely. Dealing with severe adverse event, it is important to make sure patients' airway is clear, respiration, blood pressure and heart rate is steady. Treatment in intensive care unit is recommended when necessary.

Notably, the investigator should follow up the outcome of any Adverse Events (clinical signs, laboratory values or other, etc.) until the return to normal or stabilization of the patient's condition. The follow-up will continue after the patient withdraws from the clinical trial, and the patient will be interviewed by telephone or face-to-face according to the scheduled visit time. The monitoring team may request additional visits. Follow-up will continue after the patient has left the Clinical Trial and that additional investigations may be requested by the Monitoring Team. In the case of any Serious Adverse Event brought to the attention of the Investigator at any time after cessation of Investigational Product and considered.

XII. Ethical standards

12.1 Ethical standards

This Clinical Trial will be conducted in accordance with the principles laid down by the

18th World Medical Assembly (Helsinki, 1964) and all applicable amendments laid down by the World Medical Assemblies and the ICH guidelines for Good Clinical Practice. Prior to initiating the study, each site will obtain Institutional review board (IRB) or institutional ethics committee (IEC) approval for the protocol, informed consent forms and materials used to recruit subjects. Before each subject is enrolled in this study, the investigator is responsible for fully and comprehensively introducing the purpose, procedures and possible risks of the study to the subject or his/her agent and signing a written informed consent form. And inform the subjects that they have the right to withdraw from this study at any time. The informed consent should be kept as a clinical study document for future reference. The personal privacy and data confidentiality of subjects will be protected during the study process.

12.2 Law and regulations

This Clinical Trial will be conducted in compliance with all international laws and regulations, and Chinese laws and regulations in which the Clinical Trial is performed, as well as any applicable guidelines.

12.3 Informed consent

The Investigator (according to applicable regulatory requirements), or a person designated by the Investigator and under the Investigator's responsibility, should fully inform the Patient of all pertinent aspects of the Clinical Trial including the written information giving approval/favorable opinion by the Ethics Committee (IRB/IEC). All participants should be informed to the fullest extent possible about the study, in language and terms they are able to understand. Prior to a patient's participation in the Clinical Trial, the written Informed Consent Form should be signed, name filled in and personally dated by the patient or by the patient's legally acceptable representative, and by the person who conducted the informed consent discussion. A copy of the signed and dated written Informed Consent Form will be provided to the patient. The Informed Consent Form used by the Investigator for

obtaining the patient's informed consent must be reviewed and approved by the Sponsor prior to submission to the appropriate Ethics Committee (IRB/IEC) for approval/favorable opinion.

12.4 Institutional review board/institutional ethics committee (IRB/IEC)

The Investigator or the Sponsor must submit this Clinical Trial Protocol to the appropriate Ethics Committee (IRB/IEC), and is required to forward to the Sponsor a copy of the written and dated approval/favorable opinion signed by the Chairman with Ethics Committee (IRB/IEC) composition. The Clinical Trial (study number, Clinical Trial Protocol title and version number), the documents reviewed (Clinical Trial Protocol, Informed Consent Form, Investigator's Brochure, Investigator's CV, etc.), the list of voting members along with their qualification and the date of the review should be clearly stated on the written (IRB/IEC) approval/favorable opinion.

Investigational Product will not be released at the study site and the Clinical Trial will not start until a copy of this written and dated approval/favorable opinion has been received by the Sponsor.

During the Clinical Trial, any amendment or modification to the Clinical Trial Protocol should be submitted to the Ethics Committee (IRB/IEC). It should also be informed of any event likely to affect the safety of patients or the continued conduct of the Clinical Trial, in particular any change in safety. All updates to the Investigator's Brochure will be sent to the Ethics Committee (IRB/IEC). If requested, a progress report will be sent to the Ethics Committee (IRB/IEC) annually and a summary of the Clinical Trial's outcome at the end of the Clinical Trial.

XIII. Statistical analysis

The primary null hypothesis of this study is that in patients with TIA or MIS carried *CYP2C19* LOF allele treated with aspirin 75 mg/d, there is no difference in 90-day risk of new

stroke (ischemic or hemorrhagic) in those treated with a 3-month regimen of ticagrelor compared with a 3-month regimen of clopidogrel when therapy is initiated within 24 hours of symptom onset.

13.1 Sample size estimation

Primary null hypothesis: in patients with TIA or minor ischemic stroke carried *CYP2C19* LOF allele treated with aspirin 75 mg/d, there is no difference in 90-day risk of stroke (ischemic or hemorrhagic) in those treated with a 3-month regimen of ticagrelor initiated with a loading dose of 180 mg followed by 90 mg bid compared with a 3-month regimen of clopidogrel initiated with a loading dose of 300 mg followed by 75 mg/d when therapy is initiated within 24 hours of symptom onset.

The minimum necessary sample size in the trial is established by the requirement to detect the smallest expected, clinically meaningful treatment difference comparing the treatment with placebo. Based on the genetic sub-analysis of the CHANCE study, we presume that the 90-day risk of stroke recurrence in *CYP2C19* loss-of-function allele carriers is about 9.4%, and 6.7% for noncarriers. With the point-of-care identification of the *CYP2C19* loss-of-function allele carriers to assess a proper pharmacogenetic approach for patients with high-risk TIA or MIS, we assumed a 25% relative risk reduction with alteration from clopidogrel to ticagrelor (90-day risk of stroke recurrence: 7.1%). Considering the potential impact of the interim analyses on the probability of type I error, we adjusted the statistical significance to a 2-sided α of 0.048. With a sample size of 6,396 patients, we will have 90% power to detect a relative risk reduction (Ticagrelor: loading dose: 180mg, 90mg bid on day 2-90 vs. Clopidogrel: loading dose: 300mg, 75mg qd on day 2-90) of 25% and 5% dropouts (medication nonadherence). Assuming 58.8% prevalence of *CYP2C19* LOF allele carriers in a Chinese population, we projected screening 10,878 patients would be necessary.

13.2 Data collection and entry

Paper-based case report form (CRF) and electronic data capture (EDC) system will be used for data collection and entry. All the content required by the protocol in the system must be provided, the unfilled content should be explained, and the reason needs to be filled in the remarks under each form of the EDC system.

13.2.1 Paper-based CRF filled out by investigator

Site investigators should use black or blue-black recording pens to fill out the paper-based CRF neatly and clearly to ensure that the data is clear and readable. If the paper-based CRF information needs to be modified, it should not be altered or overwritten. The correct information should be written next to the original information, signed and dated by the person who modified it. The clinical research monitor (CRA) will review the completeness and accuracy of the case report form and guide the investigator to make necessary corrections and supplements.

13.2.2 Data entry to the EDC system by CRC

After the paper-based CRF is completed, the research coordinator (CRC) will input the content of the paper CRF into the EDC system.

13.2.3 Submission to the EDC system after the approval of investigator

The paper-based CRF is submitted after the investigator has approved it. After the data is submitted, all data revisions and feedback are carried out through the EDC system. If the EDC system has submitted a form that needs to be modified, you need to contact the CRA of this center. After the CRA opens the form, the investigator can guide the CRC to modify the EDC system data.

13.2.4 Data monitoring and query by CRA via EDC

13.2.5 Data exportation from the EDC system

After the data from the EDC system is exported to the database, it will be proofread by

the data administrator. Obvious errors will be corrected by the data administrator. Other errors or missing values will be filled in the data query form and returned to the participating center for solution through email, express, telephone and WeChat .

The participating centers are responsible for correcting the data in the EDC system after verifying the original data and related information. Site investigators must answer these queries by verifying or modifying relevant information or data.

13.3 Statistical considerations

This section is an overview of the statistical considerations. It provides the general specifications for the analysis of the data to be collected and presented in the Clinical Study Report. A final SAP will be issued prior to database lock and before code breaking. The SAP will define all "pre-specified, planned analyses."

13.3.1 Analysis sets

Full analysis set (FAS): FAS is the set for efficacy evaluation. All efficacy variables will be analyzed using the FAS. According to the ITT principle, the FAS included patients who were enrolled, randomized and had the record of at least one-day treatment of study drugs. Subjects missing outcome data will be censored at the last follow-up assessment time (end of study or last visit preceding loss to follow up).

Per Protocol set (PPS): PPS includes subjects completed study treatment without serious violation against protocol. Definition of serious violation against protocol will be confirmed at data review. Generally, it may include the following situations (but not limited to these situations): failure to meet the main inclusion criteria, concomitant treatment that seriously interferes with the evaluation of the efficacy of study drugs after randomization, poor compliance, and follow-up beyond the time window. PPS is the secondary analysis population for efficacy evaluation, but if its results are inconsistent with the full analysis set, detailed

analysis of the inconsistent results is required.

Safety analysis set (SS): All patients who received at least 1 time of study drugs and safety assessment. Throughout the safety results sections, erroneously treated patients (eg, those randomized to ticagrelor and aspirin group but actually given clopidogrel and aspirin) will be accounted for in the actual treatment group.

13.3.2 Statistical considerations

1. Balance of baseline characteristics（comparison among groups）

1) Continuous variables were compared using t test or Wilcoxon rank sum test;

2) Categorical variables were compared using a $\chi 2$ test, Fisher exact test, or Wilcoxon rank sum test as appropriate.

2、Efficacy analysis

1) Primary efficacy endpoint：

Kaplan-Meier estimates of the cumulative risk of stroke or vascular event will be reported for the maximum 3-month follow-up. The log-rank test will be used to evaluate the statistical significance of the treatment effect.

2) Secondary efficacy endpoint:

① Kaplan-Meier estimates of the cumulative risk of stroke or combined vascular events will be reported during the 3-month treatment period. The hazard ratio with 95%CI for the treatment comparison will be derived using a Cox's proportional hazards model. The log-rank test will be used to evaluate the statistical significance of the treatment effect;

② ORs and 95%CIs will be compared using $\chi 2$ test or logistic analysis;

③ Continuous variable will be compared using t-test or Wilcoxon sum rank test.

④ Categorical variables were compared using a $\chi 2$ test, Fisher exact test, or Wilcoxon rank sum test as appropriate;

⑤ Comparison of neurologic deficits scales and EQ-5D-5L scales were performed using non-parametric analysis;

⑥ Extreme values were thoroughly checked and corrected before analysis, sensitivity tests were prespecified;

All statistics will be 2-sided with $P < 0.05$ considered significant.

3. Safety analysis

All patients who received at least 1 time study drugs and safety assessment were included in the safety analysis set. The safety evaluation data include adverse events observed during the trial and changes in laboratory data before and after treatment. Adverse events will be summarized using descriptive statistics. And the differences in the primary safety endpoints, secondary safety endpoints and the incidence of various adverse reactions were compared between the two treatment arms. In addition to comparing the mean of laboratory indicators before and after treatment, we will list the specific conditions of those been normal before treatment but became abnormal after treatment.

XIV. Confidentiality and publication of research findings

The principle investigator has complete intellectual property rights. The entire research process and data analysis process strictly protect the subjects' information. Publication of the results of this trial will be governed by the policies and procedures developed by the Executive Committee. The trial results will be published as soon as possible after database lockdown. This trial will produce detailed data on treatment effects, medical care, and outcomes in a cohort of subjects with TIA or MIS. CHANCE-2 biostatisticians will be consulted to assure that it is impossible to uniquely identify any participant. Diskettes with the data in comma-delimited text format will be sent to parties that express interest, including a data dictionary in a text file.

XV. Study Organization

15.1 Constitution

- Principal investigator

Yongjun Wang, Beijing Tiantan Hospital, Capital Medical University, Beijing, China

The steering committee members of CHANCE-2 study

- ✓ The steering committee will provide scientific and strategic direction for the trial and will have overall responsibility for its design, execution, and publication.
- ✓ The steering committee will also be responsible for ensuring that study execution and management are of the highest quality.
- ✓ It will approve the protocol and the operational guidelines of the trial prior to its commencement.
- ✓ The steering committee will convene regularly by teleconference or face-to-face meetings to discuss and report on the progress of the study.
- ✓ The composition of the steering committee and its responsibilities are described in a charter which will be finalized before the start of the trial.

- Executive committee

The executive committee is responsible to review the status of the trial and available blinded data and will take appropriate actions regarding the conduct of the study. A face-to-face Executive Committee meeting will be organized to make major decisions. The composition of the Executive Committee and its responsibilities are described in a charter which will be finalized before the start of the trial.

- Data safety and monitoring board (DSMB)

The DSMB will meet regularly and monitor the progress of the CHANCE-2 study to ensure that the study meets the highest standards of ethics and patient safety. It is composed of Academic Members, including an independent statistician, who are not otherwise participating

in the trial. A DSMB charter including membership role and responsibilities will be approved by both the DSMB and the Executive Committee before the start of the trial.

Written recommendations and their rationale will be provided to the Chairs of the Steering Committee immediately after each DSMB meeting.

- Adjudication committee

Clinical outcome events (stroke, MI, death, overt bleedings) will be reviewed by independent experts (neurologists, cardiologists). An adjudication committee charter including membership, role and responsibilities will be approved before the start of the trial by the Adjudication Committee and the Executive Committee.

Neuroimaging associated with clinical events will be read locally and reports will be included in adjudication packets. The adjudication committee may request actual images from sites or from the core lab in special instances.

15.2 Site training and certification

Executive committee have already provided training to their sites in Good Clinical Practice Guidelines and in some outcome assessments (e.g., *CYP2C19* genotyping, NIHSS, mRS). Prior to initiation of patient enrollment, Site Investigators and Coordinators will complete training programs and their certifications.

All investigators must complete the following training modules, and receive certification:

- Study procedures
- Primer on the diagnosis of ischemic stroke or TIA
- Use of the $ABCD^2$ score
- CHANCE-2 eligibility
- Modified Rankin Scale
- NIHSS
- ASCO etiology subtyping

- *CYP2C19* genotyping
- Clinical outcome events adjudication
- GUSTO bleeding criteria
- EQ-5D-5L
- Collecting blood sample
- Collecting DICOM imaging data

Successful completion of the training program will be required before a site is certified to enroll patients. Telephone-based meetings, with the PI and key staff available to address questions, will occur intermittently. Certification of competence will be obtainable on the training centers.

A detailed Manual of Procedures will serve as the primary document describing all study related procedures. It will serve as a guide for training of clinical center personnel and will be updated periodically throughout the study on the CHANCE website, as needed. A system composed of members of executive committee and clinical research associate will be implemented for the clinical centers to call, fax, or e-mail any procedural questions regarding the study. The CHANCE-2 executive committee and monitoring committee will formulate answers in consultation with the Steering Committee and will periodically distribute to the participating centers a set of frequently asked questions (FAQ) and answers.

The members of executive committee will manage and conduct site visits for its sites and ensure the integrity and validity of the data recorded on the Case Report Forms. Each site will be visited at least once during the trial, and as needed if questions about data quality or problems with recruitment arise.

XVI. Study monitoring and quality assurance

16.1 Responsibilities of the investigator(s)

The Investigator(s) undertake(s) to perform the Clinical Trial in accordance with this Clinical Trial Protocol, ICH guidelines for Good Clinical Practice and the applicable regulatory requirements.

The Investigator is required to ensure compliance with all procedures required by the Clinical Trial Protocol and with all study procedures provided by the Sponsor (including security rules). The Investigator agrees to provide reliable data and all information requested by the Clinical Trial Protocol (with the help of the Case Report Form [CRF], Discrepancy Resolution Form [DRF] or other appropriate instrument) in an accurate and legible manner according to the instructions provided and to ensure direct access to source documents (such as drug overdose or pregnancy) by Sponsor representatives.

The Investigator may appoint such other individuals as he/she may deem appropriate as Sub-Investigators to assist in the conduct of the Clinical Trial in accordance with the Clinical Trial Protocol. All Sub-Investigators shall be appointed and listed in a timely manner. The Sub-Investigators will be supervised by and under the responsibility of the Investigator. The Investigator will provide them with a copy of the Clinical Trial Protocol and all necessary information. The Sponsor of this Clinical Trial is responsible to Health Authorities for taking all reasonable steps to ensure the proper conduct of the Clinical Trial Protocol as regards ethics, Clinical Trial Protocol compliance, and integrity and validity of the data recorded on the Case Report Forms.

16.2 Study monitoring

The main responsibility of the monitoring team is to help researchers and sponsors to ensure that all aspects of clinical trials are highly ethical, scientific, professional, and standardized. According to the ICH guidelines for Good Clinical Practice (GCP), the Monitoring Team must check the Case Report Form entries against the source documents,

except for the pre-identified.

The monitoring team will regularly contact each center through site visits, mails or phone calls, and will send inspectors to evaluate the research progress, the adherence of the investigator and the patient to the research protocol and to solve urgent problems. During these inspection visits, the inspector will work together with the site-investigator. The main aspects of inspection and monitoring are as follows (not exclusive): patient’s informed consent, patient recruitment and follow-up, serious adverse event documentation and reporting, study drug supply, adherence to treatment of participants in the study drug arm, study drug counting, concomitant treatment and data quality.

XVII. Data retention

The double reviewed case report form and imaging data will be sent to the trial-designated data management center by clinical research associates (CRAs). The person in charge of the data management center will check and sign for receipt. The data management center should carefully enter and process the received case report forms and keep them properly. The case report form will be kept by the research center after data entry is completed.

XVIII. Data Security Monitoring

Clinical studies will develop corresponding data safety monitoring plans based on the risk. All adverse events are detailed records, deal with and track until the proper solution or in a stable condition, in a timely manner to the ethics committee in accordance with the relevant provisions, the competent department, the sponsor and the pharmaceutical supervisory and administrative departments to report serious adverse events and unexpected events, etc.; The major researchers regularly reviewed all adverse events and convened the researchers' meeting to assess the risks and benefits of the study if necessary; Is greater than the minimum risk research will arrange separate data examiner monitoring data of the study, high-risk

research will establish independent data safety monitoring committee for accumulating safety data, and effectiveness of data monitoring, whether to continue to make a research proposal.

During the clinical trial, the data of the subjects should be collected anonymously in the case report form. The subjects were identified only by the subject number and the abbreviation of the initials. Due to the reasons of safety or administrative instruction, when the subject's identity is known, the researcher and the researcher shall share the responsibility of confidentiality. Informed consent form allows the patient agree to authorized bidders, ethics committee, the authority directly refer to case report on the related original data (such as the patient's medical file case, booking records, the original laboratory records, etc.).The above personnel shall comply with occupational confidentiality rules and must keep all personal identity information or medical information confidential.

XIX. References

1. von Weitzel-Mudersbach P, Andersen G, Hundborg HH, Johnsen SP. Transient ischemic attack and minor stroke are the most common manifestations of acute cerebrovascular disease: a prospective, population-based study--the Aarhus TIA study. Neuroepidemiology. 2013;40:50-55.

2. Johnston SC, Gress DR, Browner WS, Sidney S. Short-term prognosis after emergency department diagnosis of TIA. JAMA. 2000;284:2901-2906.

3. Rothwell PM, Buchan A, Johnston SC. Recent advances in management of transient ischaemic attacks and minor ischaemic strokes. Lancet Neurol. 2006;5:323-331.

4. Hill MD, Yiannakoulias N, Jeerakathil T, Tu JV, Svenson LW, Schopflocher DP. The high risk of stroke immediately after transient ischemic attack: a population-based study. Neurology. 2004;62:2015-2020.

5. Ois A, Gomis M, Rodriguez-Campello A, Cuadrado-Godia E, Jimenez-Conde J, Pont-Sunyer C, et al. Factors associated with a high risk of recurrence in patients with transient ischemic attack or minor stroke. Stroke. 2008;39:1717-1721.

6. Wang Y, Wang Y, Zhao X, Liu L, Wang D, Wang C, et al. Clopidogrel with aspirin in acute minor stroke or transient ischemic attack. N Engl J Med. 2013;369:11-19.

7. Kernan WN, Ovbiagele B, Black HR, Bravata DM, Chimowitz MI, Ezekowitz MD, et al. Guidelines for the Prevention of Stroke in Patients With Stroke and Transient Ischemic Attack: A Guideline for Healthcare Professionals From the American Heart Association/American Stroke Association. Stroke. 2014;45:2160-2236.

8. Wang Y, Zhang S, Zhang L, Dong Q, Cui L, Pu C, et al. Chinese guidelines for the secondary prevention of ischemic stroke and transient ischemic attack 2014. Chin J Neurol. 2014;48:258-273.

9. Wang Y, Zhao X, Lin J, Li H, Johnston SC, Lin Y, et al. Association Between CYP2C19

Loss-of-Function Allele Status and Efficacy of Clopidogrel for Risk Reduction Among Patients With Minor Stroke or Transient Ischemic Attack. JAMA. 2016;316:70-78.

10. Pan Y, Chen W, Xu Y, Yi X, Han Y, Yang Q, et al. Genetic Polymorphisms and Clopidogrel Efficacy for Acute Ischemic Stroke or Transient Ischemic Attack: A Systematic Review and Meta-Analysis. Circulation. 2017;135:21-33.

11. Bonello L, Camoin-Jau L, Armero S, Com O, Arques S, Burignat-Bonello C, et al. Tailored clopidogrel loading dose according to platelet reactivity monitoring to prevent acute and subacute stent thrombosis. Am J Cardiol. 2009;103:5-10.

12. Price MJ, Berger PB, Teirstein PS, Tanguay JF, Angiolillo DJ, Spriggs D, et al. Standard- vs high-dose clopidogrel based on platelet function testing after percutaneous coronary intervention: the GRAVITAS randomized trial. JAMA. 2011;305:1097-1105.

13. Collet JP, Cuisset T, Range G, Cayla G, Elhadad S, Pouillot C, et al. Bedside monitoring to adjust antiplatelet therapy for coronary stenting. N Engl J Med. 2012;367:2100-2109.

14. Cayla G, Cuisset T, Silvain J, Leclercq F, Manzo-Silberman S, Saint-Etienne C, et al. Platelet function monitoring to adjust antiplatelet therapy in elderly patients stented for an acute coronary syndrome (ANTARCTIC): an open-label, blinded-endpoint, randomised controlled superiority trial. Lancet. 2016.

15. Mega JL, Hochholzer W, Frelinger AL, 3rd, Kluk MJ, Angiolillo DJ, Kereiakes DJ, et al. Dosing clopidogrel based on CYP2C19 genotype and the effect on platelet reactivity in patients with stable cardiovascular disease. JAMA. 2011;306:2221-2228.

16. Roberts JD, Wells GA, Le May MR, Labinaz M, Glover C, Froeschl M, et al. Point-of-care genetic testing for personalisation of antiplatelet treatment (RAPID GENE): a prospective, randomised, proof-of-concept trial. Lancet. 2012;379:1705-1711.

17. Xie X, Ma YT, Yang YN, Li XM, Zheng YY, Ma X, et al. Personalized antiplatelet therapy

according to CYP2C19 genotype after percutaneous coronary intervention: a randomized control trial. Int J Cardiol. 2013;168:3736-3740.

18. Gurbel PA, Bliden KP, Butler K, Tantry US, Gesheff T, Wei C, et al. Randomized double-blind assessment of the ONSET and OFFSET of the antiplatelet effects of ticagrelor versus clopidogrel in patients with stable coronary artery disease: the ONSET/OFFSET study. Circulation. 2009;120:2577-2585.

19. Tantry US, Bliden KP, Wei C, Storey RF, Armstrong M, Butler K, et al. First analysis of the relation between CYP2C19 genotype and pharmacodynamics in patients treated with ticagrelor versus clopidogrel: the ONSET/OFFSET and RESPOND genotype studies. Circ Cardiovasc Genet. 2010;3:556-566.

20. Wallentin L, Becker RC, Budaj A, Cannon CP, Emanuelsson H, Held C, et al. Ticagrelor versus clopidogrel in patients with acute coronary syndromes. N Engl J Med. 2009;361:1045-1057.

21. James SK, Storey RF, Khurmi NS, Husted S, Keltai M, Mahaffey KW, et al. Ticagrelor versus clopidogrel in patients with acute coronary syndromes and a history of stroke or transient ischemic attack. Circulation. 2012;125:2914-2921.

22. Wallentin L, James S, Storey RF, Armstrong M, Barratt BJ, Horrow J, et al. Effect of CYP2C19 and ABCB1 single nucleotide polymorphisms on outcomes of treatment with ticagrelor versus clopidogrel for acute coronary syndromes: a genetic substudy of the PLATO trial. Lancet. 2010;376:1320-1328.

23. Becker RC, Bassand JP, Budaj A, Wojdyla DM, James SK, Cornel JH, et al. Bleeding complications with the P2Y12 receptor antagonists clopidogrel and ticagrelor in the PLATelet inhibition and patient Outcomes (PLATO) trial. Eur Heart J. 2011;32:2933-2944.

24. de Lemos JA, Brilakis ES. No free lunches: balancing bleeding and efficacy with ticagrelor.

Eur Heart J. 2011;32:2919-2921.

25. Johnston SC, Amarenco P, Albers GW, Denison H, Easton JD, Evans SR, et al. Ticagrelor versus Aspirin in Acute Stroke or Transient Ischemic Attack. N Engl J Med. 2016;375:35-43.

26. Amarenco P, Albers GW, Denison H, Easton JD, Evans SR, Held P, et al. Efficacy and safety of ticagrelor versus aspirin in acute stroke or transient ischaemic attack of atherosclerotic origin: a subgroup analysis of SOCRATES, a randomised, double-blind, controlled trial. Lancet Neurol. 2017.

27. Wang Y, Minematsu K, Wong KS, Amarenco P, Albers GW, Denison H, et al. Ticagrelor in Acute Stroke or Transient Ischemic Attack in Asian Patients: From the SOCRATES Trial (Acute Stroke or Transient Ischemic Attack Treated With Aspirin or Ticagrelor and Patient Outcomes). Stroke. 2017;48:167-173.

Appendix Table 1. Definitions of stroke events and vascular events

**Stroke**	Acute symptoms and signs of neurologic defect caused by sudden abnormality of the blood supply. Damage of focal or whole brain, spinal or retinal vascular damage, which is related to cerebral circulation disorder.
**Ischemic stroke**	Definitions: (1) Symptoms or imaging evidence of acute newly onset focal neurologic deficit last for more than 24 hours after excluding other non-ischemic reasons, such as brain infection, head trauma, brain tumor, epilepsy, severe metabolic diseases, degeneration diseases or adverse effect of medications; or (2) Acute brain or retinal ischemic event with focal symptoms or signs lasts for less than 24 hours after excluding other causes with imaging evidence of new infarction; or (3) Progression of original vascular ischemic stroke (NIHSS increased≥4 from baseline score after excluding hemorrhagic transformation or symptomatic intracerebral hemorrhage after cerebral infarction) lasts over 24 hours with new ischemic lesion on brain MRI or CT. Which would be classified by ASCO etiology standard.
**Transient ischemic attack**	A brief episode of neurological dysfunction caused by focal brain or retinal ischemia, with clinical symptoms typically lasting less than 24 hours, and without evidence of acute infarction, after excluding other non-ischemic reasons, such as brain infection, head trauma, brain tumor, epilepsy, severe metabolic diseases, degeneration diseases or adverse effect of medications.
**Hemorrhagic stroke**	Hemorrhagic stroke was defined as focal or whole brain or spine damage caused by non-traumatic bleeding into the brain parenchyma, intraventricular or subarachnoid.
**Hemorrhagic**	Any kind of non-traumatic extravascular bleeding in the area of brain tissue of

**transformation after infarction**	existing acute/subacute infarction. Which could lead to relevant neurologic symptoms(symptomatic) or not (asymptomatic).<br>1. To determine a symptomatic hemorrhagic transformation, the following two conditions must be met，(1) Image evidence of extravascular bleeding in the area of infarction on brain CT or MRI; (2) Clinical symptoms are relevant to hemorrhagic transformation. Hemorrhagic transformation can partly explain patients′ neurological symptoms, for example: (a) The area and location of infarction can′t explain the symptoms; (b) Clinically deterioration means NIHSS increased ≥ 4 from original ischemic event or causing death directly from hemorrhagic transformation; (c) a large space-occupying hematoma develops secondary to hemorrhagic transformation.<br>2. To determine an asymptomatic hemorrhagic transformation, the following two conditions must be met, (1) Image evidence of extravascular bleeding in the area of infarction on brain CT or MRI; (2) No symptoms are caused by hemorrhagic transformation or an neurologic deterioration that lead to a less than 4 increase of NIHSS score.
**Myocardial infarction**	Third universal definition of myocardial infarction（Thygesen 2012）<br>The term acute myocardial infarction (MI) should be used when there is evidence of myocardial necrosis in a clinical setting consistent with acute myocardial ischemia. Under these conditions any one of the following criteria meets the diagnosis for MI:<br>1、 Detection of a rise and/or fall of cardiac biomarker values [preferably cardiac troponin (cTn)] with at least one value above the 99th percentile upper reference<br>limit (URL) and with at least one of the following:<br>(1) Symptoms of ischemia.<br>(2) New or presumed new significant ST-segment–T wave (ST–T) changes or

	new left bundle branch block (LBBB).<br>(3) Development of pathological Q waves in the ECG.<br>(4) Imaging evidence of new loss of viable myocardium or new regional wall motion abnormality<br>(5) Identification of an intracoronary thrombus by angiography or autopsy.<br>2、Cardiac death with symptoms suggestive of myocardial ischemia and presumed new ischemic ECG changes or new LBBB, but death occurred before cardiac<br>biomarkers were obtained, or before cardiac biomarker values would be increased.<br>3、Percutaneous coronary intervention (PCI) related MI is arbitrarily defined by elevation of cTn values (>5×99th percentile URL) in patients with normal baseline<br>values (≤99th percentile URL) or a rise of cTn values >20% if the baseline values are elevated and are stable or falling. In addition, either (1) symptoms<br>suggestive of myocardial ischemia or (2) new ischemic ECG changes or (3) angiographic findings consistent with a procedural complication or (4) imaging<br>demonstration of new loss of viable myocardium or new regional wall motion abnormality are required。<br>4、Stent thrombosis associated with MI when detected by coronary angiography or autopsy in the setting of myocardial ischemia and with a rise and/or fall of cardiac<br>biomarker values with at least one value above the 99th percentile URL.<br>5、Coronary artery bypass grafting (CABG) related MI is arbitrarily defined by elevation of cardiac biomarker values (>10×99th percentile URL) in patients with<br>normal baseline cTn values (≤99th percentile URL). In addition, either<br>(1) new pathological Q waves or new LBBB, or<br>(2) angiographic documented new graft or new native coronary artery

	occlusion, or<br>(3) imaging evidence of new loss of viable myocardium or new regional wall motion abnormality.
**Vascular death**	Vascular death include death due to stroke, cardiac sudden death, death caused by acute myocardial infarction, death caused by heart failure, death caused by pulmonary embolism, death caused by cardial/cerebral interventions or operations (not caused by myocardial infarction) and death caused by other cardiovascular diseases. (Arrhythmia irrelevant to cardiac sudden death, rupture of aortic aneurysm or peripheral artery disease).<br>Unexplained death happened within 30 days after stroke, myocardial infarction or cardiovascular/cerebral vascular operation will be considered as stroke, myocardial infarction and accidental death caused by operation separately.

Appendix Table 2. Modified Rankin Scale

The modified Rankin scale is used to measure the results of patients' functional recovery after stroke. Bold typeface shows the formal definition of each level. The italics give further guidance in order to reduce the possible errors between different observers, but there is no requirement for the structure of the interview. Please note that only symptoms that have occurred since the stroke are considered. If the patient can walk with the help of some assistive devices without outside help, it is considered to be able to walk independently.

If the two levels seem to be equally applicable to the patient, and further questions are unlikely to make an absolutely correct choice, the more severe level should be selected.

0- No symptoms at all

There may be mild symptoms. But no noticeable new functional limitations or new symptoms after stroke event.

1- No significant disability despite symptoms; able to carry out all usual duties and activities

No significant disability: symptoms present but no other limitations. Question: Does the person have difficulty reading or writing, difficulty speaking or finding the right word, problems with balance or coordination, visual problems, numbness (face, arms, legs, hands, feet), loss of movement (face, arms, legs, hands, feet), difficulty with swallowing, or other symptom resulting from stroke?

2- Slight disability; unable to carry out all previous activities but able to look after own affairs without assistance

Slight disability; limitations in participation in usual social roles, but independent for ADL. Questions: Has there been a change in the person's ability to work or look after others if these were roles before stroke? Has there been a change in the person′s ability to participate in previous social and leisure activities? Has the person had problems with relationships or become isolated?

3- Moderate disability; requiring some help, but able to walk without assistance

Moderate disability; need for assistance with some instrumental ADL but not basic ADL. Question: Is assistance essential for preparing a simple meal, doing household chores, looking after money, shopping, or traveling locally?

4- Moderately severe disability; unable to walk without assistance, and unable to attend to own bodily needs without assistance

Moderately severe disability: need for assistance with some basic ADL, but not requiring constant care. Question: Is

assistance essential for eating, using the toilet, daily hygiene, or walking?

5- Severe disability: bedridden, incontinent, and requiring constant nursing care and attention

Severe disability: someone needs to be available at all times; care may be provided by either a trained or an untrained caregiver. Question: Does the person require constant care?

Appendix Table 3. National institutes of health stroke scale

Administer stroke scale items in the order listed. Record performance in each category after each subscale exam. Do not go back and change scores. Follow directions provided for each exam technique. Scores should reflect what the patient does, not what the clinician thinks the patient can do. The clinician should record answers while administering the exam and work quickly. Except where indicated, the patient should not be coached (i.e., repeated requests to patient to make a special effort).

	Instructions	Scale Definition	Score
**1a**	Level of consciousness: The investigator must choose a response if a full evaluation is prevented by such obstacles as an endotracheal tube, language barrier, orotracheal trauma/bandages. A 3 is scored only if the patient makes no movement (other than reflexive posturing) in response to noxious stimulation	0 = Alert; keenly responsive.<br>1 = Not alert; but arousable by minor stimulation to obey, answer, or respond.<br>2 = Not alert; requires repeated stimulation to attend or is obtunded and requires strong or painful stimulation to make movements (not stereotyped). 3 = Responds only with reflex motor or autonomic effects or totally unresponsive, flaccid, and areflexic.	_____
**1b**	LOC Questions: The patient is asked the month and his/her age. The answer must be correct - there is no partial credit for being close. Aphasic and stuporous patients who do not comprehend the questions will score 2. Patients unable to speak because of endotracheal intubation, orotracheal trauma, severe dysarthria from any cause, language barrier, or any other problem not secondary to aphasia are given a 1. It is important that only the initial answer be graded and that the examiner not "help" the patient with verbal or non-verbal cues.	0 = Answers both questions correctly.<br>1 = Answers one question correctly.<br>2 = Answers neither question correctly.	_____
**1c**	LOC Commands: The patient is asked to open and close the eyes and then to grip and release the non-paretic hand. Substitute another one step command if the hands cannot be used. Credit is given if an unequivocal attempt is made but not completed due to weakness. If the patient does not respond to command, the task should be demonstrated to him or her (pantomime), and the result scored (i.e., follows none, one or two commands). Patients with trauma, amputation, or other physical impediments should be given suitable one-step commands. Only the first attempt is scored.	0 = Performs both tasks correctly.<br>1 = Performs one task correctly.<br>2 = Performs neither task correctly.	_____
**2**	Best Gaze: Only horizontal eye movements will be tested. Voluntary or reflexive (oculocephalic) eye movements will be scored, but caloric testing is not done. If the patient has a conjugate deviation of the eyes that can be overcome by voluntary or reflexive activity, the score will be 1. If a patient has an isolated peripheral nerve paresis (CN III, IV or VI), score a 1. Gaze is testable in all aphasic	0 = Normal.<br>1 = Partial gaze palsy; gaze is abnormal in one or both eyes, but forced deviation or total gaze paresis is not present.<br>2 = Forced deviation, or total gaze paresis not overcome by the oculocephalic maneuver.	_____

	Instructions	Scale Definition	Score
	patients. Patients with ocular trauma, bandages, pre-existing blindness, or other disorder of visual acuity or fields should be tested with reflexive movements, and a choice made by the investigator. Establishing eye contact and then moving about the patient from side to side will occasionally clarify the presence of a partial gaze palsy.		
**3**	Visual: Visual fields (upper and lower quadrants) are tested by confrontation, using finger counting or visual threat, as appropriate. Patients may be encouraged, but if they look at the side of the moving fingers appropriately, this can be scored as normal. If there is unilateral blindness or enucleation, visual fields in the remaining eye are scored. Score 1 only if a clear-cut asymmetry, including quadrantanopia, is found. If patient is blind from any cause, score 3. Double simultaneous stimulation is performed at this point. If there is extinction, patient receives a 1, and the results are used to respond to item 11.	0 = No visual loss.<br>1 = Partial hemianopia.<br>2 = Complete hemianopia.<br>3 = Bilateral hemianopia (blind including cortical blindness).	_____
**4**	Facial Palsy: Ask – or use pantomime to encourage – the patient to show teeth or raise eyebrows and close eyes. Score symmetry of grimace in response to noxious stimuli in the poorly responsive or non-comprehending patient. If facial trauma/bandages, orotracheal tube, tape or other physical barriers obscure the face, these should be removed to the extent possible.	0 = Normal symmetrical movements.<br>1 = Minor paralysis (flattened nasolabial fold, asymmetry on smiling).<br>2 = Partial paralysis (total or near-total paralysis of lower face).<br>3 = Complete paralysis of one or both sides (absence of facial movement in the upper and lower face).	_____
**5**	Motor Arm: The limb is placed in the appropriate position: extend the arms (palms down) 90 degrees (if sitting) or 45 degrees (if supine). Drift is scored if the arm falls before 10 seconds. The aphasic patient is encouraged using urgency in the voice and pantomime, but not noxious stimulation. Each limb is tested in turn, beginning with the non-paretic arm. Only in the case of amputation or joint fusion at the shoulder, the examiner should record the score as untestable (UN), and clearly write the explanation for this choice.	0 = No drift; limb holds 90 (or 45) degrees for full 10 seconds.<br>1 = Drift; limb holds 90 (or 45) degrees, but drifts down before full 10 seconds; does not hit bed or other support.<br>2 = Some effort against gravity; limb cannot get to or maintain (if cued) 90 (or 45) degrees, drifts down to bed, but has some effort against gravity.<br>3 = No effort against gravity; limb falls.<br>4 = No movement.<br>UN = Amputation or joint fusion, explain: _____	5a Left Arm<br><br>5b Right Arm
**6**	Motor Leg: The limb is placed in the appropriate position: hold the leg at 30 degrees (always tested supine). Drift is scored if the leg falls before 5 seconds. The aphasic patient is encouraged using urgency in the voice and pantomime, but not noxious stimulation. Each limb is tested in turn, beginning	0 = No drift; leg holds 30-degree<br><br>position for full 5 second<br><br>1 = Drift; leg falls by the end of the 5-	6a Left Leg<br><br>6b Right Leg

	Instructions	Scale Definition	Score
	with the non-paretic leg. Only in the case of amputation or joint fusion at the hip, the examiner should record the score as untestable (UN), and clearly write the explanation for this choice.	second period but does not hit bed.<br>2 = Some effort against gravity; leg falls to bed by 5 seconds but has some effort against gravity.<br>3 = No effort against gravity; leg falls to bed immediately.<br>4 = No movement.<br>UN = Amputation or joint fusion, explain: ________________	
7	Limb Ataxia: This item is aimed at finding evidence of a unilateral cerebellar lesion. Test with eyes open. In case of visual defect, ensure testing is done in intact visual field. The finger-nose-finger and heel-shin tests are performed on both sides, and ataxia is scored only if present out of proportion to weakness. Ataxia is absent in the patient who cannot understand or is paralyzed. Only in the case of amputation or joint fusion, the examiner should record the score as untestable (UN), and clearly write the explanation for this choice. In case of blindness, test by having the patient touch nose from extended arm position.	0 = Absent.<br>1 = Present in one limb.<br>2 = Present in two limbs.<br>UN = Amputation or joint fusion, explain: ________________	_____
8	Sensory: Sensation or grimace to pinprick when tested, or withdrawal from noxious stimulus in the obtunded or aphasic patient. Only sensory loss attributed to stroke is scored as abnormal and the examiner should test as many body areas (arms [not hands], legs, trunk, face) as needed to accurately check for hemisensory loss. A score of 2, "severe or total sensory loss," should only be given when a severe or total loss of sensation can be clearly demonstrated.<br>Stuporous and aphasic patients will, therefore, probably score 1 or 0. The patient with brainstem stroke who has bilateral loss of sensation is scored 2. If the patient does not respond and is quadriplegic, score 2. Patients in a coma (item 1a=3) are automatically given a 2 on this item	0 = Normal; no sensory loss.<br>1 = Mild-to-moderate sensory loss; patient feels pinprick is less sharp or is dull on the affected side; or there is a loss of superficial pain with pinprick, but patient is aware of being touched.<br>2 = Severe to total sensory loss; patient is not aware of being touched in the face, arm, and leg.	_____
9	Best Language: A great deal of information about comprehension will be obtained during the preceding sections of the examination. For this scale item, the	0 = No aphasia; normal.<br>1 = Mild-to-moderate aphasia; some obvious loss of fluency or facility of	_____

	Instructions	Scale Definition	Score
	patient is asked to describe what is happening in the attached picture, to name the items on the attached naming sheet and to read from the attached list of sentences.<br>Comprehension is judged from responses here, as well as to all of the commands in the preceding general neurological exam. If visual loss interferes with the tests, ask the patient to identify objects placed in the hand, repeat, and produce speech. The intubated patient should be asked to write. The patient in a coma (item 1a=3) will automatically score 3 on this item. The examiner must choose a score for the patient with stupor or limited cooperation, but a score of 3 should be used only if the patient is mute and follows no one-step commands.	comprehension, without significant limitation on ideas expressed or form of expression. Reduction of speech and/or comprehension, however, makes conversation about provided materials difficult or impossible. For example, in conversation about provided materials, examiner can identify picture or naming card content from patient's response.<br>2 = Severe aphasia; all communication is through fragmentary expression; great need for inference, questioning, and guessing by the listener. Range of information that can be exchanged is limited; listener carries burden of communication. Examiner cannot identify materials provided from patient response.<br>3 = Mute, global aphasia; no usable speech or auditory comprehension.	
**10**	Dysarthria: If patient is thought to be normal, an adequate sample of speech must be obtained by asking patient to read or repeat words from the attached list. If the patient has severe aphasia, the clarity of articulation of spontaneous speech can be rated. Only if the patient is intubated or has other physical barriers to producing speech, the examiner should record the score as untestable (UN), and clearly write an explanation for this choice. Do not tell the patient why he or she is being tested	0 = Normal.<br>1 = Mild-to-moderate dysarthria; patient slurs at least some words and, at worst, can be understood with some difficulty.<br>2 = Severe dysarthria; patient's speech is so slurred as to be unintelligible in the absence of or out of proportion to any dysphasia, or is mute/anarthric.<br>UN = Intubated or other physical barrier, explain:___	____
**11**	Extinction and Inattention (formerly Neglect): Sufficient information to identify neglect may be obtained during the prior testing. If the patient has a severe visual loss preventing visual double simultaneous stimulation, and the cutaneous stimuli are normal, the score is normal. If the patient has aphasia but does appear to attend to both sides, the score is normal. The presence of visual spatial neglect or anosagnosia may also be taken as evidence of abnormality. Since the abnormality is scored only if present, the item is never untestable.	0 = No abnormality.<br>1 = Visual, tactile, auditory, spatial, or personal inattention or extinction to bilateral simultaneous stimulation in one of the sensory modalities.<br>2 = Profound hemi-inattention or extinction to more than one modality; does not recognize own hand or orients to only one side of space.	____
**Total Score**			____

Appendix：1. Pictures for item 9-10

Picture 1

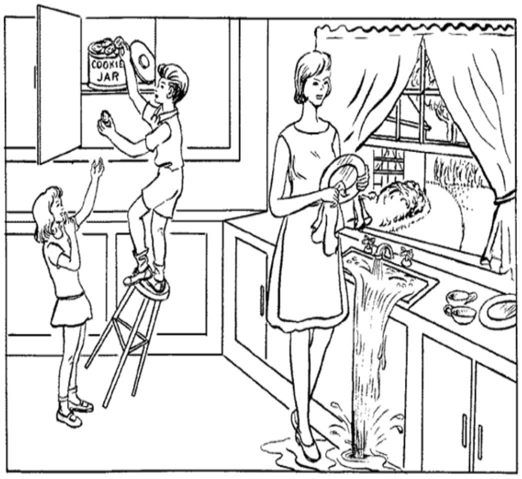

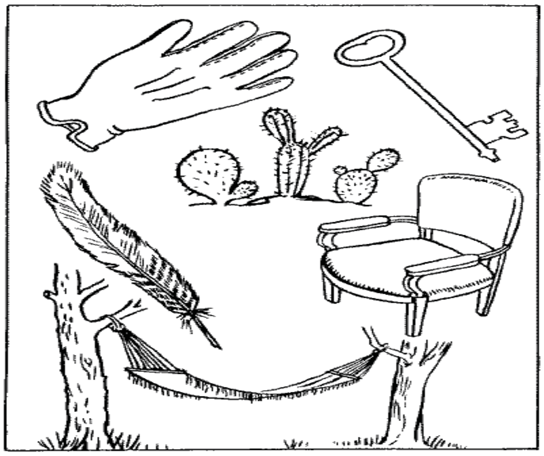

Picture 2

Picture 3

Picture 4

Please read the following scentences：

You know how.

Down to earth

I got home from work

Near the table in the dining room.

They heard him speak on the radio last night.

Please read the following scentences：

You know how.

Down to earth

I got home from work

Near the table in the dining room.

They heard him speak on the radio last night.

Appendix：2. How to evaluate the NIH stroke scale for coma patients?

In patients with a score of 1a item less than 3, each category of the score should be evaluated. A 3 is scored only if the patient makes no movement (other than reflexive posturing) in response to noxious stimulation (sternum friction or orbit pressing). The other categories should be scored as the followings:

- **1b- LOC Questions：2**
- **1c- LOC Commands：2**
- **2-Best gaze：1 = Partial gaze palsy; gaze is abnormal in one or both eyes but forced deviation or total gaze paresis is not present. 2 = Forced deviation, or total gaze paresis not overcome by the oculocephalic maneuver.**
- **3-Visual：Using visual threat to evaluate.**
- **4-Facial palsy：3**
- **5、6-Motor arm/leg：4 for each limb**
- **7- Limb Ataxia：This category can be scored only when the patient has limb ataxia. If the finger-nose-finger and heel-shin tests could not be tested due to limb weakness, then score 0.**
- **8-Sensory：2**
- **9-Best language：3**
- **10-Dysathria：2**
- **11- Extinction and Inattention：Coma means losing all kinds of cognitive abilities, then score 2.**

3、How to calculate the total score of NIH stroke score?

The following items will not be calculated into the total score:

- **The fifth, sixth item: UN = Amputation or joint fusion**

  The seventh item: - Limb ataxia presents in which limb.

Appendix Table 4. EQ-5D-5Lindex

By placing a tick in one box in each group below, please indicate which statements best describe your own health state today.

Mobility

I have no problems in walking about ☐

I have slight problems in walking about ☐

I have moderate problems in walking about ☐

I have severe problems in walking about ☐

I am unable to walk about ☐

Self-care

I have no problems with washing or dressing myself ☐

I have slight problems with washing or dressing myself ☐

I have moderate problems with washing or dressing myself ☐

I have severe problems with washing or dressing myself ☐

I am unable to wash or dress myself ☐

Usual activities (e.g. work, study, housework, family or leisure activities)

I have no problems doing my usual activities ☐

I have slight problems doing my usual activities ☐

I have moderate problems doing my usual activities ☐

I have severe problems doing my usual activities ☐

I am unable to do my usual activities ☐

Pain/discomfort

I have no pain or discomfort ☐

I have slight pain or discomfort ☐

I have moderate pain or discomfort ☐

I have severe pain or discomfort ☐

I have extreme pain or discomfort ☐

Anxiety/depression

I am not anxious or depressed ☐

I am slightly anxious or depressed ☐

I am moderately anxious or depressed ☐

I am severely anxious or depressed ☐

I am extremely anxious or depressed ☐

Illustrations

（1）Please choose only one level under each dimension.

(2) Please fill in the form by participants. The interviewer should avoid obvious guiding questions and should not chose from his/her own subjective judgement after listening to the participant's narration. The interviewer should not express his/her guiding views to the participant.

To help people say how good or bad a health state is, we have drawn a scale (rather like a thermometer) on which the best state you can imagine is marked 100 and the worst state you can imagine is marked 0.

We would like you to indicate on this scale how good or bad your own health is today, in your opinion. Please do this by drawing a line from the box below to whichever point on the scale indicates how good or bad your health state is today.

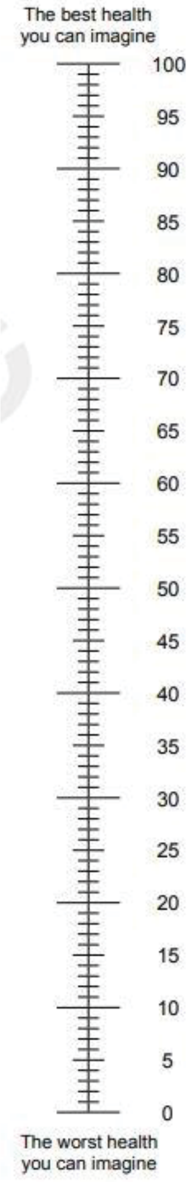

How good or bad your own health is today

Questionnaire completed by:

○1Patients independently

○2Patients with help of others

○3Agent (the patient's family members)

Version 1.0

Jan 09 2019

Appendix Table 5. Abnormal clinical laboratory indicators

Hemorrhagic tendency	The prothrombin time was 1.5 times longer than that of the normal control group or platelet < 10×10^9/L.
Moderate/severe anemia	Hemoglobin (Hb)<90g/L
Hepatic insufficiency	Aminotransaminase exceeded twice the upper limit of standard level
Renal insufficiency	Serum creatinine > 1.5mg/dl or creatinine clearance rate < 50ml/min

Appendix Table 6. Global Utilization of Streptokinase and Tissue Plasminogen Activator for Occluded Coronary Arteries (GUSTO) bleeding definition

Severe bleeding	Including life-threatening bleeding, primary intracranial hemorrhage, post-traumatic symptomatic intracranial hemorrhage that resulted in substantial hemodynamic compromise requiring treatment (blood transfusion, liquid transition, vasoconstrictor medication or surgical intervention)
Moderate bleeding	Moderate bleeding was defined by the need for transfusion, but does not reach the standard as severe bleeding; including reduction of absolute value of hemoglobin or red blood cells, causing severe disability or intraocular hemorrhage with severe visual field defect, etc.
Minor bleeding	Minor bleeding referred to other bleeding, not requiring transfusion or causing hemodynamic compromise. Including bleeding at the puncture site or hematoma, etc.

Clopidogrel with Aspirin in High-risk patients with Acute Non-disabling Cerebrovascular Events II (CHANCE-2)

Protocol

Research team：Beijing Tiantan Hospital, Capital Medical University, Beijing, China

Principal Investigator：Yongjun Wang, MD, Professor of Neurology

Protocol Version 1.4

December 9th 2020

Catalogue

Abstract of research protocol 91

Abrreviations 97

I. Background 101

II. Study purpose 110

2.1 Primary objective 110
2.2 Secondary objectives 110

III. Study design 111

3.1. Study design 111
3.2 Follow-up schedule 113
3.3 Study timeline 113

IV. Study endpoints 114

4.1 Primary efficacy endpoint 114
4.2 Secondary efficacy endpoints 114
4.3 Safety endpoint 114

V. Participant selection 115

5.1 Inclusion Criteria 115
5.2 Exclusion Criteria 115

VI. Treatments 117

6.1 Treatment arms 117
6.2 Dose regiment 118

VII. Study conduction 118

7.1 Point-of-care genetic testing of CYP2C19 genotype 118
7.2 Randomization of the CYP2C19 intermediate/poor metabolizers 119
7.3 Blinding system and emergency unblinding procedure of the CYP2C19 intermediate/poor motabolizers 120
7.4 Study drug handling 121
7.5 Concurrent treatment 123
7.6 Temporary/permanent discontinuation of the study drug(s) or loss of follow-up 125
7.7 Consequence of withdrawal 127

IX. Data collection 128

X. Study procedures 128

10.1 Screening and inclusion 128

10.2 Day of randomization (Day 1) 129
10.3 Record of hospital discharge visit 130
10.4 Day 21±2 visit 130
10.5 Day 90±7 visit 131
10.6 12 months ± 15 days visit 131
10.7 Possible event visit 132
10.8 Schedule of activities and assessments 133
XI. Study risk pre-assessment and risk management 134
11.1 Monitoring of adverse events 135
11.2 Definitions of adverse events 135
11.2.1 Adverse event (AE) 135
11.2.2 Serious adverse event (SAE) 135
11.3 Recording of adverse events 136
11.4 Causal relationship between adverse events and study drugs: 136
11.5 Safety 138
XII. Ethical standards 140
12.1 Ethical standards 140
12.2 Law and regulations 140
12.3 Informed consent 140
12.4 Institutional review board/institutional ethics committee (IRB/IEC) 141
XIII. Statistical analysis 142
13.1 Sample size estimation 142
13.2 Data collection and entry 143
13.3 Statistical considerations 144
13.4 Interim analysis 147
XIV. Confidentiality and publication of research findings 147
XV. Study Organization 148
15.1 Constitution 148
15.2 Site training and certification 150
XVI. Study monitoring and quality ~~assurance~~ control 151
16.1 Responsibilities of the investigator(s) 151
16.2 Study monitoring 152
XVII. Data retention 152
XVIII. Data Security Monitoring 153
XIX. References 154
Appendix Table 1. Definitions of stroke events and vascular events 158

Appendix Table 2. Modified Rankin Scale 162

Appendix Table 3. National institutes of health stroke scale 164

Appendix Table 5. Abnormal clinical laboratory indicators 172

Appendix Table 6. Global Utilization of Streptokinase and Tissue Plasminogen Activator for Occluded Coronary Arteries (GUSTO) bleeding definition 173

Abstract of research protocol

**Brief Title**	**Clopidogrel With Aspirin in High-risk Patients With Acute Non-disabling Cerebrovascular Events II**
**Official Title**	**A multicenter, double-blind, placebo-controlled, randomized clinical trial comparing the efficacy and safety of ticagrelor/aspirin and clopidogrel/aspirin in preventing stroke in patients with high-risk non-disabling cerebrovascular events who carry the *CYP2C19* loss-of-function allele**
**Study objective**	**Primary objective：**<br>To assess the effects of ticagrelor plus aspirin versus clopidogrel plus aspirin on reducing the 3-month risk of any new stroke (ischemic or hemorrhagic, primary outcome) when initiated within 24 hours of symptom onset in *CYP2C19* LOF alleles carriers with TIA or minor ischemic stroke.
**Study settings**	Multicenter, double-blind, placebo-controlled, randomized clinical trial
**Sample size estimation**	The minimum necessary sample size in the trial is ~~established~~ determined by the requirement to detect the smallest expected, clinically meaningful treatment difference comparing the treatment with placebo. Based on the genetic sub-analysis of the CHANCE study, we presume that the 90-day risk of stroke recurrence in *CYP2C19* loss-of-function allele carriers is about 9.4%, and 6.7% for noncarriers. With the point-of-care identification of the *CYP2C19* loss-of-function allele carriers to assess a proper pharmacogenetic approach for patients with high-risk TIA or MIS, we assumed a 25% relative risk reduction with alteration from clopidogrel to ticagrelor (90-day risk of stroke recurrence: 7.1%). Considering the potential impact of the interim analyses on the probability of type I error, we adjusted the statistical significance to a 2-sided α of 0.048. With a sample size of 6,396 patients, we will have 90% power to detect a relative risk reduction (Ticagrelor: loading dose: 180mg, 90mg bid on day 2-90 vs. Clopidogrel: loading dose: 300mg, 75mg qd on day 2-90) of 25% and 5% dropouts (medication nonadherence). Assuming 58.8% prevalence of *CYP2C19* loss-of-

	function allele carriers in ~~a~~ Chinese population, we projected screening 10,878 patients would be necessary.
Participants	**Inclusion Criteria**<br>1. 40 years old and above；<br>2. Acute cerebral ischemic event due to：<br>• Acute non-disabling ischemic stroke (NIHSS≤3 at the time of randomization)or，<br>• TIA with moderate-to-high risk of stroke (ABCD^2 score ≥ 4 at the time of randomization)；<br>3. Can be treated with study drugs within 24 hours of symptoms onset*(*Symptom onset is defined by the "last seen normal" principle)；<br>4. *CYP2C19* loss-of-function allele carriers；<br>**5.** Informed consent signed.
	**Exclusion Criteria**<br>1. Malformation, tumor, abscess or other major non-ischemic brain disease (e.g., multiple sclerosis) on baseline head CT or MRI.<br>2. Isolated or pure sensory symptoms (e.g., numbness), isolated visual changes, or isolated dizziness/vertigo without evidence of acute infarction on baseline head CT or MRI.<br>3. Iatrogenic causes (angioplasty or surgery) of minor stroke or TIA.<br>4. Preceding moderate or severe dependency (modified Rankin scale [mRS] score 3-5).<br>5. Contraindications to clopidogrel, ticagrelor or aspirin<br>• Known allergy<br>• Severe renal (creatinine exceeding 1.5 times of the upper limit of normal range) or hepatic (ALT or AST > twice the upper limit of normal range) insufficiency<br>• Severe cardiac failure (NYHA level: III to IV)<br>• History of hemostatic disorder or systemic bleeding<br>• History of thrombocytopenia or neutropenia<br>• History of drug-induced hematologic disorder or hepatic dysfunction<br>• Low white blood cell (<2×10^9/L) or platelet count (<100×10^9/L)

	6. Hematocrit (HCT) <30%<br>7. Clear indication for anticoagulation (presumed cardiac source of embolus, e.g., atrial fibrillation, prosthetic cardiac valves known or suspected endocarditis)<br>8. History of intracranial hemorrhage or amyloid angiopathy<br>9. History of aneurysm (including intracranial aneurysm and peripheral aneurysm)<br>10. History of asthma or COPD (chronic obstructive pulmonary disease)<br>11. High-risk for bradyarrhythmia (first-degree or second-degree AV block caused by sinus node disease, and brady-arrhythmic syncope without pacemaker)<br>12. History of hyperuricemia nephropathy<br>13. Anticipated requirement for long-term (>7 days) non-steroidal anti-inflammatory drugs (NSAIDs)<br>14. Planned or likely revascularization (any angioplasty or vascular surgery) within the next 3 months<br>15. Scheduled for surgery or interventional treatment requiring study drug cessation<br>16. Severe non-cardiovascular comorbidity with life expectancy < 3 months<br>17. Inability to understand and/or follow research procedures due to mental, cognitive, or emotional disorders<br>18. Dual antiplatelet treatment (or more than two antiplatelet agents) in 72 hours before randomization<br>19. Current treatment (last dose given within 10 days before randomization) with heparin therapy or oral anti coagulation<br>20. Intravenous thrombolytic therapy (such as intravenous rtPA) or mechanical thrombectomy within 24 hours prior to randomization<br>21. Gastrointestinal bleed within 3 months or major surgery within 30 days<br>22. Diagnosis or suspicious diagnosis of acute coronary syndrome<br>23. Participation in another clinical study with an experimental product during the last 30 days<br>24. Currently receiving an experimental drug or device

	25. Pregnant, currently trying to become pregnant, or of child-bearing potential and not using birth control
**Treatment Allocation**	Patients meeting the inclusion criteria and signing the informed consent will be randomized, and the first dose of study medication will be given within 24 hours of symptom onset. Patients will be randomized into 2 groups:<br>● **Ticagrelor and aspirin group**<br>Day1:Ticagrelor 180mg; placebo of clopidogrel 300mg; aspirin 75-300mg (open label)<br>Day2nd-21st: Ticagrelor 90mg bid/day; placebo of clopidogrel 75mg; aspirin 75mg (open label)<br>Day 22nd-3 months:Ticagrelor 90mg bid/day; placebo of clopidogrel 75mg<br>● **Clopidogrel and aspirin group**<br>Day 1: Clopidogrel 300mg; placebo of ticagrelor 180mg; aspirin 75-300mg (open label)<br>Day2nd-21st: Clopidogrel 75mg/day; placebo of ticagrelor 90mg bid/day; aspirin 75mg (open label)<br>Day 22nd-3 months:Clopidogrel 75mg; placebo of ticagrelor 90mg bid/day
**Efficacy and safety measures**	**Primary Outcome Measure:**<br>Any new stroke events (ischemic stroke or hemorrhagic stroke) within 3 months<br>**Secondary Outcome Measures:**<br>• Any new stroke events (ischemic stroke or hemorrhagic stroke) within 30 days and 1 year;<br>• New clinical vascular events (ischemic stroke/ hemorrhagic stroke/ TIA/ myocardial infarction/vascular death) within 3 months and 1 year；<br>• New ischemic stroke within 3 months and 1 year；<br>• Disabling stroke (Modified Rankin Scale score, mRS>1) at 3 months and 1 year；<br>• Incidence and severity of recurrent stroke and TIA during follow-up to 3 months and 1-year (Severity is measured using a six-level ordered

	categorical scale that incorporates the mRS: fatal stroke/severe non-fatal stroke [mRS 4 or 5]/moderate stroke [mRS 2 or 3]/mild stroke [mRS 0 or 1]/TIA/no stroke-TIA);<br>• Neurological impairment at 3 months (NIHSS increased≥4 from baseline);<br>• Quality of Life (EuroQol EQ-5D scale) at 3 months and at 1 year.
	**Safety Outcome Measure**<br>**1) Primary Safety Outcome Measure:**<br>Moderate and severe bleeding events according to the GUSTO criteria at 3-month<br>**2) Secondary Primary Safety Outcome Measures：**<br>• Incidence of severe bleedings or moderate bleedings (GUSTO definition) at 1 year；<br>• All bleeding events (severe/moderate bleeding and intracranial hemorrhage) at 3-month and 1-year；<br>• Total mortality at 3-month and 1-year；<br>• Adverse events/Severe adverse events reported by investigators at 3-month and 1-year.
**Statistical considerations**	All statistics will be 2-sided with $P < 0.05$ considered significant.<br>1. Primary null hypothesis: in patients with TIA or minor ischemic stroke carried *CYP2C19* LOF allele treated with aspirin 75 mg/d, there is no difference in 90-day risk of stroke (ischemic or hemorrhagic) in those treated with a 3-month regimen of ticagrelor initiated with a loading dose of 180 mg followed by 90 mg bid compared with a 3-month regimen of clopidogrel initiated with a loading dose of 300 mg followed by 75 mg/d when therapy is initiated within 24 hours of symptom onset.<br>2. Data set of statistical analysis: intention to treat (ITT)<br>3. Statistical analysis：<br>**3.1 Balance of baseline characteristics（comparison between groups）**<br>1）Continuous variables will be compared using *t* test or Wilcoxon rank sum test；<br>2）Categorical variables will be compared using a $\chi 2$ test, Fisher exact test, or Wilcoxon rank sum test as appropriate.

	**3.2 Effectiveness analysis**<br>1）Primary efficacy outcome：Kaplan-Meier estimates of the cumulative risk of new stroke will be reported for the maximum 3-month follow-up. The hazard ratio with 95%CI for the treatment comparison will be derived using a Cox's proportional hazards model. The log-rank test will be used to evaluate the statistical significance of the treatment effect.<br>2）Secondary efficacy outcome：<br>① Kaplan-Meier estimates of the cumulative risk of stroke or combined vascular events will be reported during the 3-month treatment period. The hazard ratio with 95%CI for the treatment comparison will be derived using a Cox's proportional hazards model. The log-rank test will be used to evaluate the statistical significance of the treatment effect；<br>② Continuous variables ~~were~~ will be compared using t test or Wilcoxon rank sum test；<br>③ Categorical variables will be compared using a $\chi 2$ test, Fisher exact test, or Wilcoxon rank sum test as appropriate；<br>④ Comparison of neurologic impairment and EQ-5D-5L scales will be performed using non-parametric analysis<br>⑤ Extreme values will be thoroughly checked and corrected before analysis, sensitivity tests will be prespecified.<br>**3.3 Safety analysis: Rate of safety end points and adverse events** will be **compared.**<br>Subgroup analyses will be based on the carrier status of the *CYP2C19* LOF allele.
**Follow-up schedule**	Study visits will take place on day of randomization, ~~at~~ day21±2, and at day 90±7 via face-to-face interview. Study visit at month 12 (±15 day) will take place via telephone interview for clinical events.

ABRREVIATIONS

Abbreviations	Interpretation of the meaning
ACEI	Angiotensin converting enzyme inhibitors
ACS	Acute coronary syndrome
AE	Adverse event
AHA	American Heart Association
ALP	Alkaline phosphatase
ANTARCTIC	Platelet function monitoring to adjust antiplatelet therapy in elderly patients stented for an acute coronary syndrome
ALT	Alanine aminotransferase
ADC	Apparent diffusion coefficient
ARCTIC	Double Randomization of a Monitoring Adjusted Antiplatelet Treatment Versus a Common Antiplatelet Treatment for DES Implantation, and Interruption Versus Continuation of Double Antiplatelet Therapy
ARR	Absolute risk reduction
ARU	Aspirin reaction unit
ASA	Aspirin, Acetylsalicylic acid
AST	Aspartate aminotransferase
CABG	Coronary artery bypass graft
CHANCE	Clopidogrel with Aspirin in High-risk patients with Acute Non-disabling Cerebrovascular Events
CHANCE-2	Clopidogrel with Aspirin in High-risk patients with Acute Non-disabling Cerebrovascular Events II
COPD	Chronic obstructive pulmonary disease
Cox1	Cyclooxygenase 1
Cox2	Cyclooxygenase 1
CRA	Clinical research associate
CRF	Case report form

CRO	Contract Research Organization
CT	Computed Tomography
CYP2C19	Cytochrome P450 2C19
CYP3A	Cytochrome P450 3A
DAE	Discontinuation of Investigational Product due to Adverse Event
DICOM	Digital imaging and communications in medicine
DSMB	Data and Safety Monitoring Board
DWI	Diffusion weighted imaging
EDC	Electronic Data Capture System
ELEVATE-TIMI 56	Escalating Clopidogrel by Involving a Genetic Strategy - Thrombolysis In Myocardial Infarction 56
EM	extensive metabolizer
EQ-5D-5L	EuroQol five dimensions questionnaire
FAS	Full analysis set
FLAIR	Fluid attenuated inversion recovery
GCP	Good clinical practice
GPIIb/IIIa	Glycoprotein IIb/IIIa
GRAVITAS	Gauging Responsiveness with A VerifyNow assay-Impact on Thrombosis And Safety
GRE-T2*	Gradient recalled echo-T2*
GUSTO	Global Use of Strategies to Open Occluded Coronary Arteries
Holter	24-hour dynamic electrocardiogram
HR	Hazard ratio
IEC	Independent Ethics Committee
IM	intermediate metabolizer

IRB	Institutional Review Board
ITT	Intention-to-treat
LOCF	Last observation carried forward
LOF	Loss of functional
MRA	Magnetic resonance angiography
MRI	magnetic resonance imaging
mRS	Modified Rankin Scale
NCRC-ND	National Clinical Research Center of Neurological Diseases
NIHSS	National Institute of Health Stroke Score
NYHA	New York Heart Association
PCI	Percutaneous coronary intervention
PCR	Polymerase chain reaction
PLATO	The Study of Platelet Inhibition and Patient Outcomes Trial
PM	Poor metabolizer
POCT	Point-of-care Testing
POPular	Cost-effectiveness of Genotype Guided Treatment With Antiplatelet Drugs in STEMI Patients: Optimization of Treatment
PPI	Proton-pump inhibitor
RAPID GENE	Point-of-care genetic testing for personalisation of antiplatelet treatment
PHARMCLO	Pharmacogenetics of Clopidogrel in Acute Coronary Syndromes
rt-PA	Recombinant Human Tissue Plasminogen Activator
PRINCE	Platelet Reactivity in Acute Non-disabling Cerebrovascular Events
PPS	Per Protocol Set
SAE	Severe adverse event

SAP	Statistical analysis plan
SOCRATES	Acute Stroke or Transient Ischaemic Attack Treated with Aspirin or Ticagrelor and Patient Outcomes
SS	Safety set
SSRIs	Selective Serotonin Reuptake Inhibitor
TAILOR-PCI	Tailored Antiplatelet Therapy Following PCI
UM	ultrarapid metabolizer

I. Background

1. Combined therapy with clopidogrel and aspirin could effectively reduce the recurrent risk of non-disabling ischemic cerebrovascular events and has been recommended by recent guidelines

Non-disabling ischemic cerebrovascular events (NICE), including minor ischemic stroke (MIS) and transient ischemic attack (TIA), accounted for 65% of ischemic cerebrovascular events.[1] Early risk of recurrence after NICE was up to 10% to 20%, but these patients could be neglected for their minor or rapidly improving neurologic deficits. These events warn us that in a short window period, we have an opportunity for urgent prevention.[2-5] According to the CHANCE (Clopidogrel in high-risk patients with acute non-disabling cerebrovascular events) study, combined therapy with clopidogrel and aspirin was associated with a 32% relative risk reduction in terms of stroke recurrence within 90 days. (Figure 1) [6] Findings of the CHANCE study provided important evidence for the treatment recommendations of Chinese and international guidelines.[7, 8]

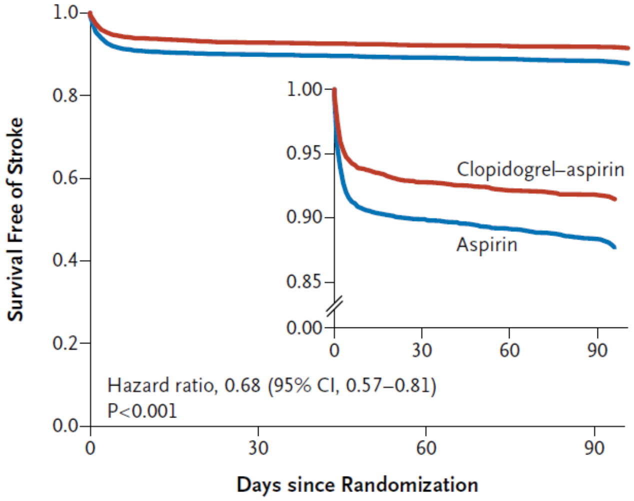

No. at Risk				
Aspirin	2586	2307	2287	1906
Clopidogrel–aspirin	2584	2376	2361	1989

Figure 1： Cumulative Probability of Survival Free of Stroke (Ischemic or Hemorrhagic Stroke) by treatment. Red line for Clopidogrel plus Aspirin, and blue line for Aspirin alone. ***N Engl J Med.*** 2013; 369(1):11-19

2. Carrier status of the *CYP2C19* loss-of-function allele was associated with different response to antiplatelet treatment, especially in Asian patients

In the pharmacogenetic sub-study of the CHANCE trial, the *CYP2C19* LOF carrier genotypes were associated with less protection from subsequent stroke and composite vascular events for patients with NICE in China. Noncarrier of the *CYP2C19* LOF alleles could have an additional 17% benefits from combined therapy of clopidogrel and aspirin. But the use of clopidogrel plus aspirin did not reduced recurrent stroke or composite vascular events in carriers of the *CYP2C19* LOF alleles (Figure 2).[9] In a meta-analysis of 15 studies, carriers of the *CYP2C19* LOF alleles were at increased risk of stroke recurrence (risk ratio, RR:1.92) (Figure 3).[10] These findings may justify genetic testing when clopidogrel is otherwise considered the preferred treatment modality, especially in Asian patient populations with a high prevalence of *CYP2C19* LOF allele. As the variation rate of *CYP2C19* gene in Chinese is as high as 58.8%, its impact on the efficacy of clopidogrel is far greater than that in western populations. Therefore, screening suitable populations based on genotyping and accurately formulating effective antiplatelet treatment is a major clinical problem that needs to be resolved urgently.

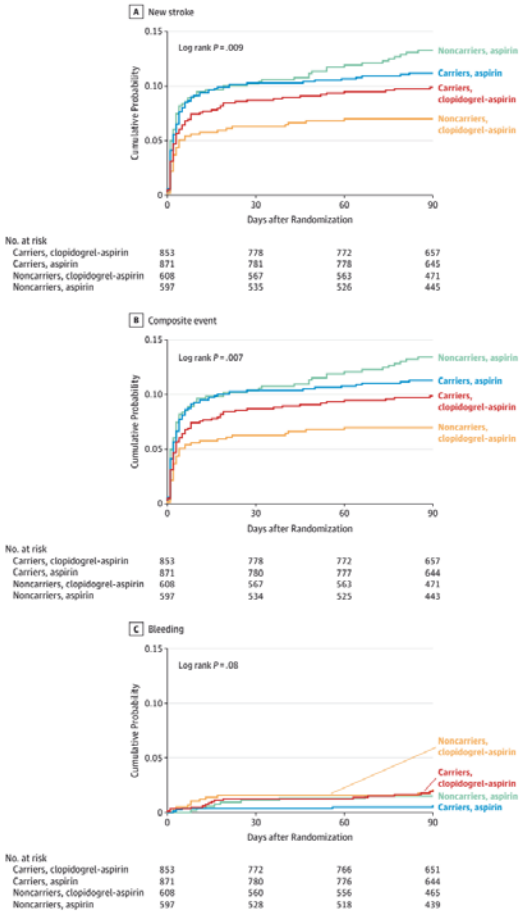

Figure 2: Cumulative Probability of Stroke, Composite Event, and Bleeding According to CYP2C19 Loss-of-Function Allele Carrier Status. JAMA, 2016,316 (1):70-78

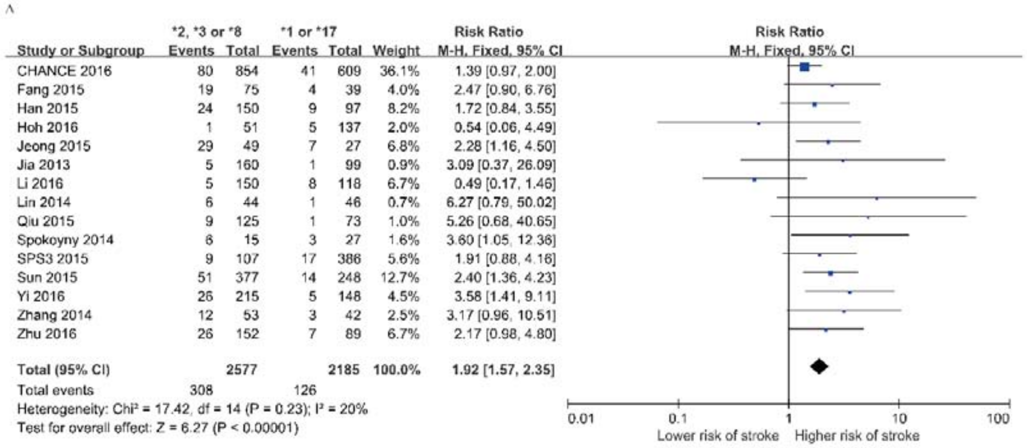

Figure 3: Risk of Stroke for Acute Ischemic Stroke or Transient Ischemic Attack Patients with Any Copy of CYP2C19 *2, *3 or *8 to wild-type (*1) or *17. Circulation.2017; 135 (1):21-33.

3. Genotyping-guided antiplatelet therapy has become a new focus in international research in the era of precision medicine

How to identify the most suitable patients and personalize the choose of antiplatelet agent is extremely challenging. Many researchers have tried to guide antiplatelet therapy based on the results of in vitro platelet reactivity tests, but the results are not satisfactory. A clinical trial in France found that vasodilator-stimulated phosphoprotein (VASP)-guided loading dose clopidogrel could lower the risk of stent thrombosis at 30 days.[11] But platelet function test-guided clopidogrel use was not associated with reduced cardiovascular or cerebrovascular events after acute coronary syndrome (ACS) according to the GRAVITAS (Gauging Responsiveness with A VerifyNow assay—Impact on Thrombosis And Safety) study[12], the ARCTIC(The Assessment by a Double Randomization of a Conventional Antiplatelet Strategy versus a Monitoring-guided Strategy for Drug-Eluting Stent Implantation and of Treatment Interruption versus Continuation One Year after Stenting) study[13], and the ANTARCTIC (Tailored Antiplatelet Therapy Versus Recommended Dose of Prasugrel) study[14]. According to the CREATIVE (Clopidogrel response evaluation and antiplatelet intervention in high thrombotic risk PCI patients) trial, in patients with low responsiveness to clopidogrel, as measured by thromboelastography, the intensified antiplatelet strategies (triple antiplatelet therapy) with adjunctive use of cilostazol significantly improved the clinical outcomes after percutaneous coronary intervention (PCI).[15] However, triple antiplatelet therapy increases the risk of bleeding in stroke patients, so this strategy could not be extrapolated to stroke patients. The application prospects of guiding drug therapy based on platelet reactivity in ischemic stroke are not

optimistic. Researchers have turned their attention to the effectiveness and feasibility of tailoring antiplatelet therapy based on genotyping.

With the rapid development of point-of-care genetic testing technology, genotyping guides antiplatelet therapy trials to be continuously optimized. According to the ELEVATE-TIMI 56 (Escalating clopidogrel by involving a genetic strategy - thrombolysis in myocardial infarction 56) study[16] and the RAPID GENE study[17], rapid genetic test coupled with subsequent personalized treatment reduces the on-treatment platelet reactivity compared to traditional antiplatelet therapy among *CYP2C19* LOF carriers after PCI. In a Chinese study randomized 623 patients after PCI, combined therapy of clopidogrel and aspirin in intermediate metabolizers and triple antiplatelets in poor metabolizers could effectively reduce the 6-month risk of MI, stroke and vascular death.[18] The ongoing TAILOR-PCI and POPular studies will also provide more clinical evidence for genotyping to guide antiplatelet therapy in cardiovascular patients. The PHARMCLO study showed that high-risk acute coronary syndrome patients benefit more from the application of precision therapy, and rapid point-of-care genetic testing (POCT) has a promising prospect for precision therapy. Although the trial was terminated early, it can still prove that precision therapy improves prognosis. Up to now, there is still a lack of similar research in the field of ischemic cerebrovascular disease. For carriers of *CYP2C19* loss-of-function alleles, whether increasing the dose of clopidogrel or switching to a new P2Y12 receptor antagonist is better remains unclear. Relevant research evidence is urgently needed.

4. Intensive antiplatelet therapy is expected to become a new option for patients with non-disabling ischemic cerebrovascular disease

Results of the ELEVATE-TIMI 56 trial indicated that elevated dose of clopidogrel intensified the platelet inhibition but did not reduce the risk of cardiac ischemic events. Moreover, for *CYP2C19*2* homozygotes, doses as high as 300 mg daily did not result in comparable degrees of platelet inhibition.[16] Ticagrelor is a reversibly binding, potent, oral adenosine diphosphate (ADP) P2Y12 receptor blocker.[19] The metabolism of ticagrelor does not involve CYP450 enzyme, thus not influenced by carrier status of *CYP2C19* LOF allele.[20]

According to the PLATO (platelet inhibition and patient outcomes) study, ticagrelor was superior to clopidogrel in reducing the rate of the composite efficacy endpoint of Cardiovascular (CV) death, myocardial infarction (MI), and stroke after ACS events within 12 months (9.8% vs.11.7%, HR 0.84, 95%CI 0.77-0.92, P< 0.001).[21] And among patients with a history of stroke or TIA, the reduction of the total mortality at one year with ticagrelor vs. clopidogrel was consistent with the overall trial results and with a trend of even greater reduction.[22] In the genetic sub analysis of the PLATO trial, ticagrelor is a more efficacious treatment for ACS than clopidogrel, irrespective of *CYP2C19* polymorphisms.[23] Ticagrelor, when compared with clopidogrel, was associated with similar total major bleeding[24, 25] but with increased non-coronary artery bypass bridging (CABG) and non-procedure-related major bleeding. This increased risk of spontaneous hemorrhage was seen primarily after a more than 30 days of dual antiplatelet treatment. And the increased risk of spontaneous hemorrhage could be partly attributed to higher dose of aspirin. Therefore, the treatment regimen of the PLATO trial is not suitable for stroke patients.

The SOCRATES trial is a superiority research comparing the efficacy of ticagrelor and aspirin in patients with TIA or MIS. Although the expected results were not

achieved[24], it provides sufficient evidence that ticagrelor is superior to aspirin in ~~reduce~~ reducing subsequent risk of major vascular events in acute-phase high-risk patients.[25] Noticably, in the subgroup analysis of Asian patients in the SOCRATES trial, there was a trend toward a lower hazard ratio in reducing risk of the primary end point of stroke, MI, or death in the ticagrelor group (ticagrelor: 9.6% vs. aspirin: 11.6%，HR 0.81, 95% CI 0.67 ~ 0.99，$P = 0.04$) [26]On the other hand, the study also confirmed the safety of ticagrelor in patients with ischemic stroke. Whether in the overall population or in the Asian population, the risk of bleeding in ticagrelor group is similar to that in aspirin group (P = 0.45 in the overall population and in the Asian population). The SOCRATES and PLATO indicated that in patients with non-disabling ischemic cerebrovascular disease with a high risk of recurrence, especially for Asian patients, the combination of ticagrelor and aspirin antiplatelet therapy may be a more effective treatment option.

The Prince trial (Platelet Reactivity in Acute Non-disabling Cerebrovascular Events，NCT02506140), a proof-of-concept randomized clinical trial, intended to test the difference of efficacy and safety between ticagrelor plus aspirin and clopidogrel and aspirin in reducing 90-day stroke recurrence after TIA or MIS when treatment initiated within 24 hours after symptom onset (Figure 4), particularly in carriers of the *CYP2C19* LOF allele. Patients with TIA or MIS who were treated with ticagrelor plus aspirin had a lower proportion of high platelet reactivity than those who were treated with clopidogrel plus aspirin (Figure 5). There was a trend toward lower risk of stroke recurrence (Figure 6). No difference was seen in the rates of major hemorrhagic events between the two groups[27].

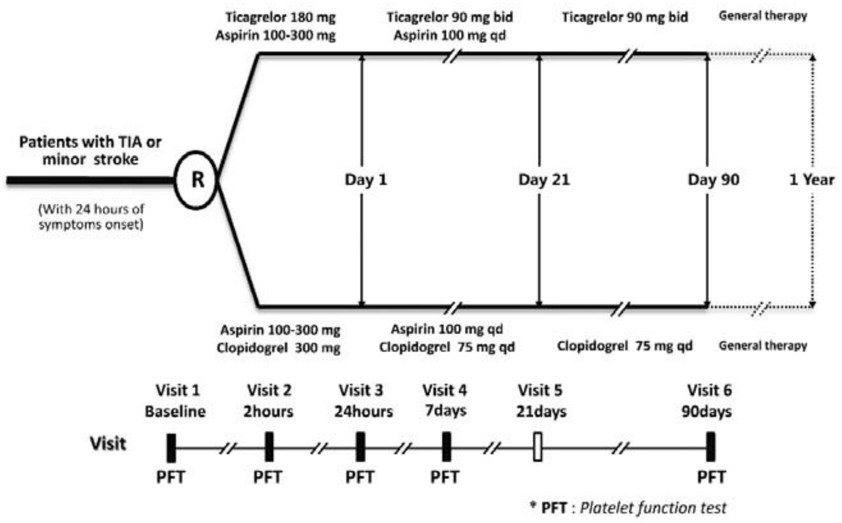

Figure 4： Flowchart of the PRINCE study. Platelet Reactivity in Acute Non-disabling Cerebrovascular Events (PRINCE) is designed as a prospective, multi-center, randomized, open-label, active-controlled, and blind-endpoint, phase II b trial. (NCT02506140).

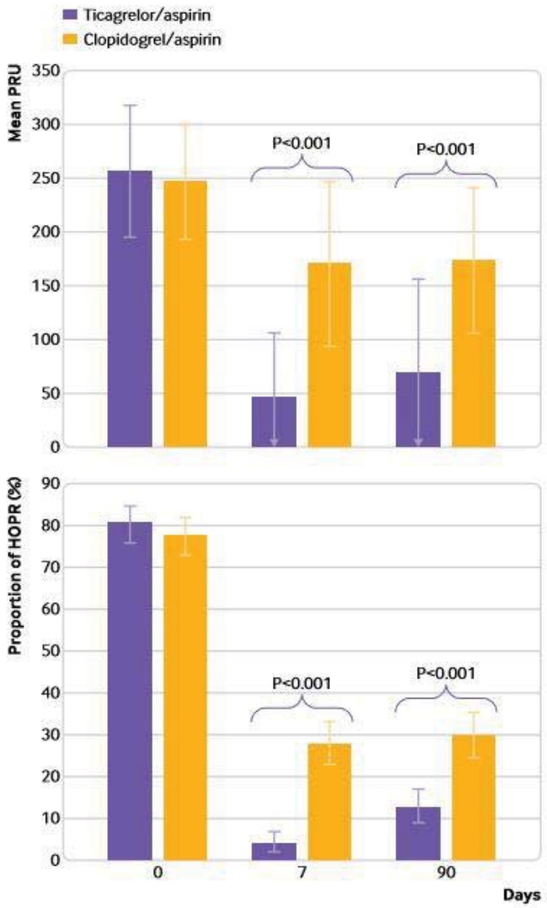

Figure 5： Platelet reactivity in trial groups at baseline and follow-up, showing P2Y12 reaction units (PRU; mean (standard deviation)) and proportion (%; 95% confidence intervals) of patients with high platelet reactivity (HOPR; PRU >208 as measured by the VerifyNow P2Y12 assay). A total of 333, 306, and 280 patients in the ticagrelor/aspirin group and 336, 321, and 290 patients in the clopidogrel/aspirin group were included in the 0, 7, and 90 day analyses, respectively

Outcome / Phenotype	Ticagrelor/aspirin No with event/total No (%)	Clopidogrel/aspirin No with event/total No (%)	Hazard ratio or risk ratio (95% CI)	P value	P for interaction
**HOPR at 90 days**					
Poor	4/38 (10.5)	14/33 (42.4)	0.23 (0.07 to 0.55)	0.004	0.42
Intermediate	12/113 (10.6)	41/124 (33.1)	0.34 (0.18 to 0.58)	<0.001	
Extensive	16/117 (13.7)	25/119 (21.0)	0.59 (0.33 to 1.03)	0.07	
Ultra	0/1 (0.0)	2/5 (40.0)	NA		
Unknown	1/6 (16.7)	2/4 (50.0)	0.33 (0.02 to 2.44)	0.29	
Total	33/275 (12.0)	84/285 (29.5)	0.40 (0.27 to 0.56)	<0.001	
**Stroke**					
Poor	4/39 (10.3)	5/41 (12.2)	0.82 (0.22 to 3.05)	0.77	0.98
Intermediate	11/138 (8.0)	16/145 (11.0)	0.71 (0.33 to 1.54)	0.39	
Extensive	4/136 (2.9)	8/133 (6.0)	0.48 (0.14 to 1.58)	0.23	
Ultra	0/1 (0.0)	0/6 (0.0)	NA		
Unknown	0/7 (0.0)	1/4 (25.0)	NA		
Total	19/321 (5.9)	30/329 (9.1)	0.64 (0.36 to 1.13)	0.13	
**Composite events**					
Poor	4/39 (10.3)	6/41 (14.6)	0.68 (0.19 to 2.39)	0.54	0.99
Intermediate	12/138 (8.7)	17/145 (11.7)	0.73 (0.35 to 1.54)	0.60	
Extensive	4/136 (2.9)	8/133 (6.0)	0.48 (0.14 to 1.58)	0.23	
Ultra	0/1 (0.0)	0/6 (0.0)	NA		
Unknown	0/7 (0.0)	1/4 (25.0)	NA		
Total	20/321 (6.2)	32/329 (9.7)	0.63 (0.36 to 1.10)	0.10	
**Major or minor events**					
Poor	3/39 (7.7)	2/41 (4.9)	1.61 (0.27 to 9.65)	0.60	0.97
Intermediate	3/138 (2.2)	6/145 (4.1)	0.53 (0.13 to 2.11)	0.37	
Extensive	6/136 (4.4)	4/133 (3.0)	1.45 (0.41 to 5.15)	0.56	
Ultra	0/1 (0.0)	0/6 (0.0)	NA		
Unknown	0/7 (0.0)	0/4 (0.0)	NA		
Total	12/321 (3.7)	12/329 (3.6)	1.03 (0.46 to 2.29)	0.94	

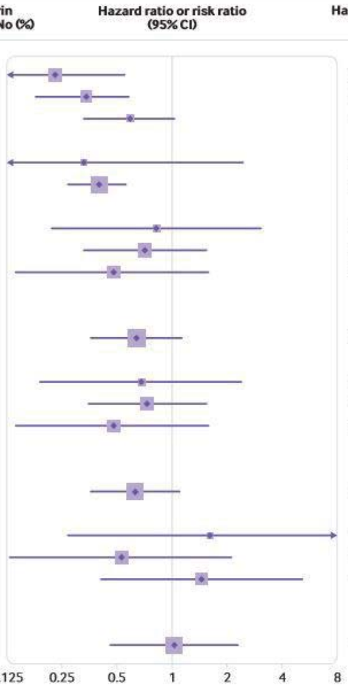

Figure 6：Effect of ticagrelor/aspirin versus clopidogrel/aspirin on high platelet reactivity and clinical outcome in PRINCE trial participants at 90 days, stratified by metaboliser status. A total of 321 patients in the ticagrelor/aspirin group and 329 patients in the clopidogrel/aspirin group were included in the genetic analysis. Patients with two *2 or *3 alleles (*2/*2, *2/*3, or *3/*3) were classified as having a poor metaboliser phenotype, those with one *2 or *3 allele (*1/*2 or *1/*3) were classified as having an intermediate metaboliser phenotype, those without a *2, *3, or *17 allele (*1/*1) were classified as having an extensive metaboliser phenotype, and those with a single *17 allele (*1/*17) and *17 homozygotes were classified as having an ultra-metaboliser phenotype. HOPR=P2Y12 reaction units of more than 208, as measured the VerifyNow P2Y12 assay; composite event=a new clinical vascular event, including stroke, transient ischaemic attack, myocardial infarction, or death from cardiovascular causes; NA=not applicable

5. New hypothesis and new strategy

Based on previous evidence, we hypothesize that pharmacogenetic test-guided antiplatelet treatment is superior to current guideline-recommended strategy. Therefore, we plan to carry out this multi-center, double-blind, double-simulation, randomized controlled clinical study to compare the effects of ticagrelor plus aspirin versus clopidogrel plus aspirin on reducing the 3-month risk of any stroke (ischemic or hemorrhagic, primary outcome) when initiated within 24 hours of symptom onset in *CYP2C19* LOF alleles carriers with TIA or minor ischemic stroke (MIS) with the guide of point-of-care *CYP2C19* genotyping. And we aim to provide diagnosis and treatment evidence for individualized antiplatelet therapy.

II. Study purpose

2.1 Primary objective

To assess the effects of ticagrelor plus aspirin versus clopidogrel plus aspirin on reducing the 3-month risk of any new stroke (ischemic or hemorrhagic, primary outcome) when initiated within 24 hours of symptom onset in *CYP2C19* LOF alleles carriers with TIA or minor ischemic stroke.

2.2 Secondary objectives

2.2.1 To assess the different rate of composite vascular outcome: composite of any stroke, TIA, myocardial infarction, and vascular death at 3-month and at one-year;

2.2.2 To assess the effects of ticagrelor plus aspirin regimen versus clopidogrel plus aspirin regimen on the incidence of: stroke within 30 days, neurological impairment at 3 months (NIHSS increased≥4 from baseline), ischemic stroke, TIA, MI, vascular death, disabling stroke (mRS: 2-6) at 3-month and at one-year;

2.2.3 To assess the incidence and severity of recurrent stroke and TIA during follow-up to 3 months and 1-year (Severity is measured using a six-level ordered categorical scale

that incorporates the mRS: fatal stroke/severe non-fatal stroke [mRS 4 or 5]/moderate stroke [mRS 2 or 3]/mild stroke [mRS 0 or 1]/TIA/no stroke-TIA);

2.2.4 To compare the safety of the two treatment regimens in terms of:

- Severe or moderate bleeding（GUSTO definition）
- Bleeding events
- Total mortality
- Adverse events/ Severe adverse events

2.2.5 To evaluate Quality of Life (EuroQol EQ-5D scale) among survivors

2.2.6 Subgroup analyses: The primary efficacy endpoint will also be analyzed stratified by age (<65 vs. ≥65 years), gender (men vs. women), Body Mass Index (BMI), index event type (TIA vs. minor stroke), time from index event to randomization, etiology subtype, diabetes mellitus, hypertension, type of LOF allele, previous ischemic stroke or TIA, prior antiplatelet therapy, prior statin therapy, and prior smoking status. Symptomatic intracranial and extracranial artery stenosis will also be evaluated in subgroup analyses.

III. Study design

3.1. Study design

This is a multicenter, double-blind, placebo-controlled, randomized clinical trial, which is designed to assess the effects of ticagrelor plus aspirin versus clopidogrel plus aspirin on reducing the 3-month risk of any stroke (both ischemic and hemorrhagic, primary outcome) when initiated within 24 hours of symptom onset in *CYP2C19* LOF alleles carriers with TIA or minor ischemic stroke.

3.1.1 Ticagrelor plus aspirin group**:**

- Day 1: two tablets of ticagrelor 90 mg, four tablets of placebo clopidogrel 75mg, and open label ASA (75mg-300mg)
- From D2 to D21±2 days: one tablet of ticagrelor 90mg twice per day, one tablet of placebo clopidogrel 75mg per day, and open label ASA (75mg) per day
- From D22 to D90±7 days: one tablet of ticagrelor 90mg twice per day and one tablet of placebo clopidogrel (75mg) per day

The first administration should be within 24 hours of the onset of symptoms.

3.1.2 Clopidogrel plus aspirin group:

- Day 1: four tablets of clopidogrel 75mg, two tablets of placebo ticagrelor 90 mg, and open label ASA (75mg-300mg)
- From D2 to D21±2 days: one tablet of clopidogrel 75mg per day, one tablet of placebo ticagrelor 90mg twice per day, and open label ASA (75mg) per day
- From D22 to D90±7 days: one tablet of clopidogrel 75mg per day, and one tablet of placebo ticagrelor (90mg) twice per day

The first administration should be within 24 hours of the onset of symptoms.

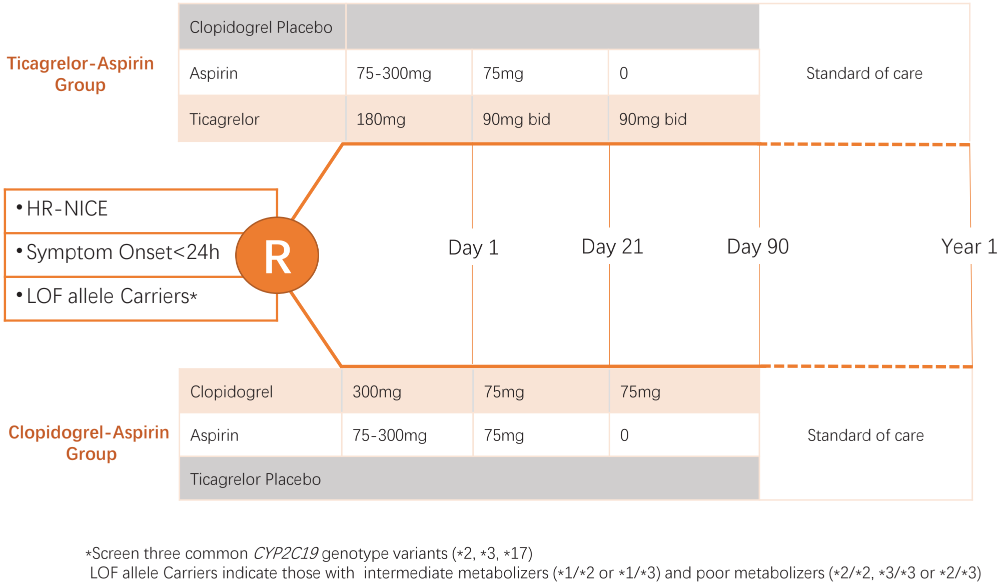

Figure 7. Study design

3.2 Follow-up schedule

Study visits will take place on ~~the~~ day of randomization, ~~at~~ day21±2, and ~~at~~ day 90±7 via face-to-face interview. Final diagnosis, etiologic subtype and relevant evaluation or treatment will be recorded at hospital discharge visit. After the day 90±7 ~~days~~ visit, a long-term follow-up will be scheduled. If a patient experiences a potential clinical neurological event, including a clinical deterioration that could be possibly related to ischemia, or new transient or persistent neurological symptoms, an adjudication packet will be produced by the site within 72 hours.

3.3 Study timeline

A 4-year study plan has been created. The timeline and tasks of the CHANCE-2 trial are shown in the Table below.

**Progress**	**Time interval**
Completion of research design, ethical review, research registration, recruitment of clinical sites	8 months (2018.10-2019.06)

Recruiment and enrollment	24 months (2019.06-2021.06)
Interim analysis	2020.12
Completion of 3-month follow-up*	3 months (2021.06-2021.09)
Data cleansing, analysis and publication	9 months (2021.09-2022.06)
**Total duration**	**44 months**

* Based on the 3-month follow-up, long-term follow-up will not be taking into account

IV. Study endpoints

4.1 Primary efficacy endpoint

Any new stroke events (ischemic stroke or hemorrhagic stroke) within 3 months

4.2 Secondary efficacy endpoints

- Any new stroke events (ischemic stroke or hemorrhagic stroke) within 30 days and 1 year；
- New clinical vascular events (ischemic stroke/ hemorrhagic stroke/ TIA/ myocardial infarction/vascular death) within 3 months and 1 year；
- New ischemic stroke events within 3 months and 1 year；
- Disabling stroke (Modified Rankin Scale score, mRS>1) at 3 months and 1 year；
- Incidence and severity of recurrent stroke and TIA during follow-up to 3 months and 1-year (Severity is measured using a six-level ordered categorical scale that incorporates the mRS: fatal stroke/severe non-fatal stroke [mRS 4 or 5]/moderate stroke [mRS 2 or 3]/mild stroke [mRS 0 or 1)]/TIA/no stroke-TIA);
- Neurological impairment at 3 months (NIHSS increased≥4 from baseline);
- Quality of Life (EuroQol EQ-5D scale) at 3 months and at 1 year.

4.3 Safety endpoint

1) Primary safety endpoint

Incidence of severe bleedings or moderate bleedings (GUSTO definition) at 3 months

2) Secondary safety endpoints

- Incidence of severe bleedings or moderate bleedings (GUSTO definition) at 1 year;
- Bleeding events (severe or moderate bleedings or intracranial hemorrhage) at 3 months and 1 year;
- Mortality at 3 months and 1 year;
- AEs/SAEs reported by the investigators at 3 months and 1 year.

V. Participant selection

During the course of the trial, about 6,396 *CYP2C19* LOF allele(s) carriers with TIA or minor ischemic stroke will be enrolled. Before enrolling patients, all collaborating sites need to be approved by local Institutional Review Boards (IRBs), which ~~will~~ have access to all study documentation and educational materials.

5.1 Inclusion Criteria

1. 40 years old and above；
2. Acute cerebral ischemic event due to：

- Acute non-disabling ischemic stroke (NIHSS≤3 at the time of randomization) or，
- TIA with moderate-to-high risk of stroke recurrence ($ABCD^2$ score ≥ 4 at the time of randomization)；

3. Can be treated with study drugs within 24 hours of symptoms onset*(*Symptom onset is defined by the "last see normal" principle)；
4. *CYP2C19* loss-of-function allele carriers；
5. Informed consent signed.

5.2 Exclusion Criteria

1. Malformation, tumor, abscess or other major non-ischemic brain disease (e.g., multiple sclerosis) on baseline head CT or MRI.
2. Isolated or pure sensory symptoms (e.g., numbness), isolated visual changes, or isolated dizziness/vertigo without evidence of acute infarction on baseline head CT or MRI.
3. Iatrogenic causes (angioplasty or surgery) of minor stroke or TIA.
4. Preceding moderate or severe dependency (modified Rankin scale [mRS] score 3-5).
5. Contraindications to clopidogrel, ticagrelor or aspirin
   - Known allergy
   - Severe renal (creatinine exceeding 1.5 times of the upper limit of normal range) or hepatic (ALT or AST exceeding twice the upper limit of normal range) insufficiency
   - Severe cardiac failure (NYHA level: III to IV)
   - History of Hemostatic disorder or systemic bleeding
   - History of thrombocytopenia or neutropenia
   - History of drug-induced hematologic disorder or hepatic dysfunction
   - Low white blood cell ($<2\times10^9$/L) or platelet count ($<100\times10^9$/L)
6. Hematocrit (HCT) <30%
7. Clear indication for anticoagulation (presumed cardiac source of embolus, e.g., atrial fibrillation, prosthetic cardiac valves known or suspected endocarditis)
8. History of intracranial hemorrhage or amyloid angiopathy
9. History of aneurysm (intracranial aneurysm and peripheral aneurysm)
10. History of asthma or COPD (chronic obstructive pulmonary disease)
11. High-risk for bradyarrhythmia (first-degree or second-degree AV block caused by sinus node disease, and brady-arrhythmic syncope without pacemaker)
12. History of hyperuricemia nephropathy
13. Anticipated requirement for long-term (>7 days) non-steroidal anti-inflammatory

drugs (NSAIDs)

14. Planned or likely revascularization (any angioplasty or vascular surgery) within the next 3 months

15. Scheduled for surgery or interventional treatment requiring study drug cessation

16. Severe non-cardiovascular comorbidity with life expectancy < 3 months

17. Inability to understand and/or follow research procedures due to mental, cognitive, or emotional disorders

18. Dual antiplatelet treatment (or more than two antiplatelet agents) in 72 hours before randomization

19. Current treatment (last dose given within 10 days before randomization) with heparin therapy or oral anti coagulation

20. Intravenous thrombolytic therapy (such as intravenous rtPA) or mechanical thrombectomy within 24 hours prior to randomization

21. Gastrointestinal bleed within 3 months or major surgery within 30 days

22. Diagnosis or suspicious diagnosis of acute coronary syndrome

23. Participation in another clinical study with an experimental product during the last 30 days

24. Currently receiving an experimental drug or device

25. Pregnant, currently trying to become pregnant, or of child-bearing potential and not using birth control

VI. Treatments

6.1 Treatment arms

***CYP2C19* intermediate or poor metabolizers (double blind)**

Ticagrelor plus asipirin	Day 1	two tablets of ticagrelor 90 mg, four tablets of placebo clopidogrel 75mg, and open label ASA (75mg – 300mg)
	From D2 to D21±2 days	one tablet of ticagrelor 90mg twice per day, one tablet of placebo clopidogrel 75mg per day, and open label ASA (75mg) per day
	From D22 to D90±7	one tablet of ticagrelor 90mg twice per day and one tablet

	days	of placebo clopidogrel (75mg) per day
Clopidogrel plus aspirin	Day 1	four tablets of clopidogrel 75mg, two tablets of placebo ticagrelor 90 mg, and open label ASA (75mg – 300mg)
	From D2 to D21±2 days	one tablet of clopidogrel 75mg per day, one tablet of placebo ticagrelor 90mg twice per day, and open label ASA (75mg) per day
	From D22 to D90±7 days	one tablet of clopidogrel 75mg per day, and one tablet of placebo ticagrelor (90mg) twice per day

6.2 Dose regiment

Patients will be randomly assigned to ticagrelor plus aspirin or clopidogrel plus aspirin according to the ratio of 1:1 within 24 h of the onset of symptoms of *CYP2C19* LOF allele(s) carriers with high-risk TIAs, defined as an $ABCD^2$ score ≥4, or minor ischemic stroke, with a National Institutes of Health Stroke Scale (NIHSS) score ≤3. The study is conducted in a double-blind manner. Placebos for clopidogrel or ticagrelor will be identical to the active drugs in appearance and taste. Minor side effects are unusual with the medication, so it is not anticipated that either subjects or clinicians will be able to differentiate the placebos from the active drugs.

- **Ticagrelor plus Aspirin Group**

  Ticagrelor of loading dosing of 180mg followed by 90mg bid for 3 months plus aspirin of loading dose of 75-300mg followed by 75mg daily for 21 days

- **Clopidogrel plus Aspirin Group**

  Clopidogrel of loading dosing of 300mg followed by 75mg daily for 3 months plus aspirin loading dose of 75-300mg followed by 75mg daily for 21 days

The study drugs should be initiated as soon as possible within 24 hours of symptoms onset.

VII. Study conduction

7.1 Point-of-care genetic testing of *CYP2C19* genotype

The *CYP2C19* gene contains four common variant star (*) alleles denoted as *1,

*2, *3, and *17. *CYP2C19*1* is the wild-type allele. *CYP2C19*2* allele (dbSNP rs4244285) is the 681G>A variant on exon 5 of *CYP2C19* gene, which is the most common loss-of-function allele. *CYP2C19*3* allele (dbSNP rs4986893), another LOF allele, is the 636G>A variant on exon 4. Carriers of *CYP2C19*2* or **3* have decreased activity of CYP2C19 enzyme, and the concentration of active ~~activated~~ metabolite in vivo is about one-third lower than that of wild type allele carriers. Carrying LOF allele will weaken the antiplatelet effect of clopidogrel. *CYP2C19*17* allele (dbSNP rs12248560) is a -806C>T variant on the 5' flanking region of the gene, this variation generates a specific binding site for nucleoprotein, binding of the nucloprotein to this site significantly increase the level of CYP2C19 gene transcription. Elevated amount of *CYP2C19* enzyme results in increased concentration of effective metabolite in vivo. Genotyping of the CYP2C19*2,*3, and*17 alleles for all enrolled patients with results reported within 1 hour can quickly provide information for the investigators to determine the patient's *CYP2C19* gene metabolizer status： ① Extensive metabolizer, EM): *1/*1, without *2, *3, and *17; ② Intermediate metabolizer, IM: with one *2 or *3 allele (*1/*2 or *1/*3); ③ Poor metabolizer, PM: with at least two *2 or *3 alleles (*2/*2, *2/*3, or *3/*3); ④ Ultrarapid metabolizer, UM: carrying at least one *17 allele (*1/*17 or *17/*17), which is extremely rare in Chinese population.

In this study a novel point-of-care genetic test platform will be used to identify carriers of the *CYP2C19* LOF alleles. The *CYP2C19* Genotyping is implemented by the GMEX® Point-of-Care Genotyping system, which includes a portable DNA analyzer, genotyping reagents, and a buccal sample collection kit. Please refer to the "Instruction Manual for Point-of-care Genotyping Testing" for specific testing methods.

7.2 Randomization of the *CYP2C19* intermediate/poor metabolizers

The list of randomization code ~~list~~ will be generated centrally by a Contract

Research Organization (CRO).

The patient kits will be packaged in accordance with this randomization code list. During the treatment period, the patient will receive study medication according to the corresponding group, either the ticagrelor plus ASA group or the clopidogrel plus ASA group, using a randomization ratio of 1:1.

The treatment number will be allocated using a centralized treatment allocation system on D1 (baseline visit). Subjects will be randomly assigned into groups, from small to large, to obtain the treatment number. According to this number, the investigator will give the first box of the corresponding package to the patient. Based on the rogress of each center, the goal of enrollment can be adjusted, but it needs to be confirmed by the data management commitee.

7.3 Blinding system and emergency unblinding procedure of the CYP2C19 intermediate/poor motabolizers

7.3.1 Description of blinding methods

This randomized double-blind study is primarily designed to compare a ticagrelor /ASA combination followed by ticagrelor alone regimen versus a clopidogrel/ASA combination followed by clopidogrel alone regimen.

The two types of ticagrelor tablets developed (90 mg active ticagrelor and placebo ticagrelor) are indistinguishable (identical in size, shape, color, appearance, taste), so are the two types of clopidogrel tablets developed (75 mg active clopidogrel and placebo clopidogrel)

No locally used biological test that could potentially unblind the treatment is planned in this study. Investigators won't be able to access the randomization (treatment) code unless in some special circumstances, such as occurrence of ~~a~~ serious adverse events, knowledge of the medication condition is considered necessary for treating the subjects.

7.3.2 Emergency unblinding procedure during the study

In case of an Adverse Event, the code may be broken only under such exceptional circumstances as knowledge of the Investigational Product is essential for treating the

patient. If possible, please contact the Monitoring Team before breaking the code. If the physician in charge believes unblinding is needed, he/she must call the monitoring committee and the sponsor immediately. All the calls will be documented by the CRO. If the blind is broken, the Investigator needs to document the date, time and reason for breaking the blind. Study drugs will be stopped afterwards.

7.4 Study drug handling

7.4.1 Supply and storage

The sponsor will supply all investigational products used in the study. All these drugs will be stored in a secure place, under the responsibility of the Investigator or other authorized individuals, and under the conditions described on the labeling.

7.4.2 Packaging and labeling

Each patient (the CYP2C19 intermediate/poor motabolizers) will be assigned a kit according to the randomization number: Each patient kit consists of a public box and two patient boxes. In accordance to the stage of the study, the contents of the patient boxs are as follows:

Box 1 (D1 to D 21±2 days): 1 wallet of open label aspirin, 46 ticagrelor 90mg tablets, 26 clopidogrel 75mg tablets;

Box 2 (D21±2 days to D 90±7 days): 158 ticagrelor 90mg tablets, 79 clopidogrel 75mg tablets.

Specifically, for Group1 Ticagrelor plus asipirin group

Box 1: 1 wallet of open label aspirin, 46 ticagrelor 90mg tablets, 26 placebo clopidogrel 75mg tablets;

Box 2: 158 ticagrelor 90mg tablets, 79 placebo clopidogrel 75mg tablets.

For Group2 Clopidogrel plus aspirin group

Box 1: 1 wallet of open label aspirin, 26 clopidogrel 75mg tablets, 46 placebo ticagrelor 90mg tablets;

Box 2: 79 clopidogrel 75mg tablets, 158 placebo ticagrelor 90mg tablets.

Label of box:

- Abbreviation of the study: CHANCE-2

- Symbol of the study
- Randomization number
- Box 1 (D1 to D 21±2 days), Box 2 (D21±2 days to D 90±7 days)
- Names of drugs
- Form, specification, route of administration and dosage of the study drug
- Indications
- Storage conditions
- Period of validity
- Please take the medicine as prescribed by the doctor, and please bring back the unused medicine, which will be counted at the next visit
- Keep away from children to prevent them from taking it by mistake
- For clinical research use only
- Please swallow carefully to avoid suffocation

7.4.3 Responsibilities of drug handling

The Investigator, the Hospital Pharmacist, or other personnel, who are allowed to store and dispense the Investigational Products, will be responsible for a secure storage of the products as specified by the Sponsor and in accordance with the applicable regulatory requirements.

All Products shall be dispensed in accordance with the Investigator's prescription. It is the Investigator's responsibility to ensure that ~~an~~ accurate records of product dispensing and return are kept.

Any quality problems noticed during the receipt or use of the Products (defects in appearance, labels, expiration date, etc.) should be promptly reported to the Sponsor, who will initiate a complaint procedure.

Under no circumstances will the Investigator supply the Products to a third party, allow~~s~~ the Products to be used other than as directed by this Clinical Trial Protocol, or dispose of the Products in any other manner.

7.4.4 Compliance to treatment and accountability

The administration of all medication (including study medication and concomitant

medication) should be recorded in the appropriate sections of the CRF. At Day 1 (randomization), patients will receive enough study medication to cover up to 21 days visit. At 21 days visit, patients will receive enough study medication to cover up to 90 days visit. Patients will be asked to return all unused study medications and empty packages at 21 days visit and 90 days visit. Pill counts should be done at individual patient level and recorded in both the CRF and a dispensing log by the study site personnel.

The study personnel will account for all study medication dispensed to and returned from the patient to evaluate the study compliance.

Information about the time of discontinuation and restart of study drugs should be recorded.

7.4.5 Return and destruction of study drugs

The investigator (or pharmacist) should have a detailed record of drug return, and the investigator and the monitoring team should jointly review and confirm the record.

The Investigators should not destruct any unused or partially used study drugs unless authorized in writing by the principal investigator.

If the study drug has quality defects, the investigator will adopt a recall procedure. In order to recall the study drug and eliminate potential hazards, the investigator is responsible for meeting any requirements put forward by the principal investigator.

7.5 Concurrent treatment

During the study period, any medication, surgery, and vascular intervention should be recorded in the case report form.

7.5.1 Prohibited concomitant treatments

1) Antiplatelet agents: Dipyridamole, Cilostazol, Ticlopidine, Prasugrel, GPIIb/IIIa receptor antagonist, Ozagrel, etc.

2) All anticoagulants

3) Thrombolytic drugs: rt-PA, urokinase and streptokinase, etc. Patients who received intravenous or arterial thrombolysis 24 hours before enrollment should be excluded. If the patient receives intravenous or intraarterial thrombolysis after enrollment, the study

treatment must be discontinued for more than 24 hours before restart.

4) Batroxobin, defibrase, snake venom preparation, lumbrokinase, etc.

5) Non-steroidal anti-inflammatory drugs (Cox1 and Cox2 inhibitors). The use of non-steroidal anti-inflammatory drugs (Cox1 and Cox2 inhibitors) within 7 days after randomization is a violation of the protocol. If absolutely necessary, non-steroidal anti-inflammatory drugs can be used 24 hours after discontiuation of the study drugs, the time to start the use must be no earlier than 8 days after randomization, and the period of the use must be less than 5 days.

6) Potent CYP3A inhibitors: ketoconazole, nefazodone, ritonavir, saquinavir, atazanavir, nelfinavir, itraconazole, voriconazole, clarithromycin, telithromycin (but ~~Does~~ not include erythromycin or azithromycin)。

7) Potent CYP3A inducers: rifampicin, dexamethasone, phenytoin, carbamazepine, oxcarbazepine, phenobarbital.

8) Potent *CYP2C19* inhibitors: omeprazole, esomeprazole; fluvoxamine, fluoxetine, moclobemide, fluconazole, chlorbenzylpyridine, ciprofloxacin, cimetidine, chlorine Mycin.

9) Potent P-glycoprotein (P-gp) inhibitors: verapamil, quinidine, cyclosporine.

10) P-glycoprotein substrate: digoxin. If digoxin is absolutely necessary, the dose of digoxin should be reduced as appropriate, and the blood concentration of digoxin should be closely monitored.

7.5.2 Permitted concomitant treatments

Any drugs other than those listed above are permitted. Patients with underlying diseases (hypertension, diabetes, coronary heart disease, epilepsy, etc.) can use diuretics, beta blockers, ACEIs, ARBs, calcium antagonists, lipid-lowering medications, coronary vasodilators, anti-diabetic medication (including Insulin), and anti-epileptics, etc. Information about diagnosis, medication, dose and administration should be collected and recorded in the CRF.

1) After randomization, patients who develop an indication (ACS or Percutaneous coronary intervention (PCI)) for dual antiplatelet therapy must stop the study therapy

and be treated with standardized care. Detailed information about the adjustment of the treatment should be recorded in the CRF.

2) H2 receptor blockers are permitted except cimetidin.

3) Proton pump inhibitors (PPIs): If necessary, PPIS can be used except omeprazole and esomeprazole. Rabeprazole is recommended. Other PPIs can also be used, such as dexlansoprazole, lansoprazole and pantoprazole.

4) Statin: for simvastatin and lovastatin, daily dose should be ≤40 mg; for other statin, any dose is permitted.

5) Drugs that can induce bradycardia: such as beta blockers, calcium antagonists, etc., when combined with ticagrelor, heart rate should be closely monitored.

6) Serotonin reuptake inhibitors (SSRIs): such as paroxetine, sertraline, and citalopram, may increase the risk of bleeding. If ~~the~~ concomitant use of SSRIs is inevitable, bleeding events should be carefully monitored.

7.5.3 Surgery and invasive procedure

During the three months period after randomization, if intervention (surgery or invasive procedure including vascular surgery, coronary artery angioplasty/ angiography , internal carotid/external carotid artery angioplasty/angiography) with high risk of bleeding is absolutely necessary, the study drugs should be stopped 5 days before intervention. After surgery, when the investigator believes that the risk of bleeding is low, the study drugs can be used again. For other surgery or invasive procedure, whether to discontinue the study drug(s) is up to the investigator to judge.

7.6 Temporary/permanent discontinuation of the study drug(s) or loss of follow-up

The study drug(s) should be continued whenever possible. If the study drug(s) are stopped, it should be determined whether they can be stopped temporarily; permanent study drug(s) discontinuation should be a last resort. Any study drug discontinuation should be fully documented in the CRF. Discontinuation of study medication does not mean discontinuation of follow-up or termination of study participation. In any case, the patient should stay in the study as long as possible.

7.6.1 Temporary treatment discontinuation of the study drug(s)

If the study drugs must be temporarily discontinued, they should be re-started as soon as possible:

Including the following conditions:

1) Severe thrombocytopenia (platelet count <50,000/uL). Patients may restart study

medication once the severe thrombocytopenia resolves;

2) Severe bleeding (GUSTO definition);
3) Need of treatment with prohibited concomitant medications, see Section 7.5;
4) Surgery or procedures associated with major haemorrhage, see Section 7.5.3; For other surgery or other invasive procedures, study medication may be continued or interrupted temporarily at the discretion of the investigator.

Once the Investigator has considered that the role of the Investigational Product(s) in the occurrence of the event concerned is unlikely and there are no other contraindications to prevent its(their) continued use in the study. Treatment with the Product can be re-initiated under close and appropriate clinical/and or laboratory monitoring.

7.6.2 Permanent treatment discontinuation of the study drug(s)

1) Patient decision. The patient is at any time free to discontinue treatment, without prejudice to further treatment

2) Investigator’s decision, including but not limited to these examples:

① Pregnancy or desire to become pregnant;

② Incorrectly enrolled patient, violation of the inclusion/exclusion criteria would put the patient at undue risk;

③ Severe non-compliance to study protocol;

④ Adverse events that lead the investigators to believe continued treatment may put the patient at undue risk;

⑤ Clear indication for anticoagulation;

⑥ After a temporary withdrawal, the investigator decides that the patient should not restart the study drugs again.

7.6.3 Handling of patients after definitive treatment discontinuation

Patients who permanently discontinued the study medication should be given conventional therapy.

A patient who decided to discontinue the study medication will always be asked why they want to discontinue and whether there are adverse events. Any adverse events need to be followed up; and the patient should return all study drugs.

All patients withdrawn prior to the last visit should have a complete end-of-study visit at three months or up to recovery or stabilization of an AE, which ever comes last.

All withdrawals should be confirmed, documented and recorded by the investigator on the appropriate CRF pages.

7.6.4 Loss to follow-up

The investigator should make every effort to contact the patient, find out the reason for his loss of follow-up, and determine his health status. For patients identified as lost to follow-up, the CRF must be completed up to their last visit.

7.7 Consequence of withdrawal

Patients who have withdrawn no longer participate in the study. Their patient number and treatment must not be re-used.

The investigator will call the monitoring team to notify the treatment discontinuation and/or the patient's withdrawal.

VIII. Biological sample collection

The collected blood samples should be sent to the relevant laboratory. The serum, plasma, and white blood cells in the blood samples should be separately processed (see the Biological Sample Collection and Processing Manual for details), and then transported via cold-chain transportation to Beijing Tiantan Hospital for centralized storage and data analysis. The time of blood samples collection should be recorded in detail on the blood sample information page.

IX. Data collection

Referring to the procedure manual and data collection guidelines, investigators should guarantee the input of CRF is precise, complete and timely, and answer the queries in time. Brain MRI which includes: T1+T2+DWI+FLAIR+ADC+GRE-T2*/SWI+MRA, will be collected as DICOM format. Vascular evaluation, transthoracic echocardiography, 24-hour Holter and laboratory results will be collected in photocopies of the reports or in DICOM format.

X. Study procedures

Investigator(s) should keep a record, the Eligibility CRF, of subjects who entered prestudy screening. The sub-center number must be indicated. The screening table will be used to analyze and determine whether the enrolled patients in different study sites are representative.

10.1 Screening and inclusion

- All participants need to sign a written informed consent at beginning
- Medical history, medication history and physical examination;
- $ABCD^2$ score for TIA patients and neurological evaluation (Modified Rankin Scale and NIHSS) will be performed for screening subjects;
- Emergency laboratory examinations will be performed: emergency blood routine, emergency renal function, emergency liver function (transaminase), emergency coagulation, random blood glucose, etc.;
- An electrocardiogram (ECG) will be performed (12-lead) to rule out atrial fibrillation, sick sinus syndrome, second or third degree atrioventricular block;
- A head CT or MRI scan will be required to rule out hemorrhage, vascular malformation, tumor, or abscess；

(Since ECG and brain imaging are routinely recommended for all patients with ischemic stroke and TIA, this study will not cover these costs)

- Women of childbearing age need to take a urine pregnancy test to exclude pregnancy;
- Orally inform patients of complete research-related information, and give them

corresponding written materials;

- Patients who meet the inclusion criteria but not the exclusion criteria need to undergo rapid *CYP2C19* genotyping. Subjects with *CYP2C19* intermediate metabolism;
- Subjects will be given randomization numbers.

10.2 Day of randomization (Day 1)

10.2.1 Baseline evaluation

- Baseline demographic characteristics;
- Symptoms of the index event, clinical course, past medical history, medications, cigarette and alcohol use, family history;
- Measurement of height, weight, waistline, physical examination, NIHSS at screening, $ABCD^2$, prestroke Rankin Score examination findings.

10.2.2 Collection of biological sample

- The blood samples should be collected from all subjects on the day they are admitted to the group and before they take the study drugs, and must be processed in accordance with the requirements of the operation manual Serum, plasma, and white blood cells should be packed separately and transported to the clinical resources and biological sample bank of the China National Clinical Research Center for Neurological Diseases via cold-chain transportation.

10.2.3 Study drug handling

- Public box includes open-label aspirin and patient box 1 (Day 1 to Day 21±2) corresponding to the treatment number.
- The study drugs will be started as soon as possible after randomization (within **one hour** after completing the point-of-care *CYP2C19* genotyping)
- The investigator will instruct the patient to take the first dose of the study drugs. The total dose on D1 includes any ASA treatment taken by the patient himself, and ASA given to the patient on the same day in the ambulance, emergency unit or after admission.

10.2.4 Schedule of auxilliary tests

- Schedule brain MRI (Includeing: T1+T2+DWI+FLAIR+ADC+GRE-T2*/SWI+MRA, should be completed within 72 hours after randomization) and

24h holter

- Schedule vascular evaluation (should be completed before discharge)

10.3 Record of hospital discharge visit

- Information will be collected on drug accountability;
- Information will be collected on concomitant medications;
- Information will be collected on new stroke or vascular events since the last visit;
- Information will be collected on adverse events or bleeding events since the last visit;
- Confirm that the brain imaging examination and vascular assessment have been completed;
- Raw data of auxiliary tests will be collected (photocopy of reports or imaging data in DICOM format);
- Record the final diagnosis and etiologic subtype according to the TOAST classification of the qualifying event;
- Record the discharge diagnosis of the subject;
- A neurological evaluation will be performed (Modified Rankin Scale and NIHSS);
- An appointment will be scheduled for the next study visit (21±2 days after randomization, face-to-face interview)

Inform the patients not to discard the pill box, empty medicine board and all wrapping paper, and bring them back to the hospital at the next follow-up visit

10.4 Day 21±2 visit

- A physical examination will be performed, including measurement of weight (kg) and vital signs (supine systolic and diastolic blood pressure, heart rate) and neurological examination;
- A neurological evaluation will be performed (Modified Rankin Scale and NIHSS);
- Treatment Boxes (public box of open-label aspirin and patient box 1) with unused study medication from completed study period (with Day 1 and Day 2 to Day 21±2 boxes) will be collected for drug accountability and assessment of treatment compliance;

Attention: Carefully count the ~~number of~~ unused pills and record the numbers

- Treatment Box 2 will be dispensed to the patient and he will be instructed how to take the study drugs until the next visit;

Inform the patients not to discard the pill box, empty medicine board and all wrapping paper, and bring them back to the hospital at the next follow-up visit

- Information will be collected on drug accountability and compliance;
- Information will be collected on **concomitant medications**;
- Information will be collected on new stroke or vascular events since the last visit;
- Information will be collected on adverse events or bleeding events since the last visit;
- An appointment will be scheduled for the next study visit (90±7 days after randomization, face-to-face interview)

10.5 Day 90±7 visit

- A physical examination will be performed, including measurement of weight (kg) and vital signs (supine systolic and diastolic blood pressure, heart rate) and neurological examination;
- A neurological evaluation will be performed (Modified Rankin Scale and NIHSS);
- Treatment Boxes (treatment box 2) with unused study medication will be collected for drug accountability and assessment of treatment compliance;

Attention: Carefully count the unused pills and record the numbers

- Information will be collected on drug accountability and compliance;
- Information will be collected on **concomitant medications**;
- Information will be collected on new stroke or vascular events since the last visit;
- Information will be collected on adverse events or bleeding events since the last visit;
- Quality of life scale (EQ-5D-5L) will be performed;
- Fill in the research summary;
- The subject and the investigator jointly decide which antiplatelet drugs should be used after the study;
- An appointment will be ~~made~~ scheduled for the next long-term study visit (12 months ± 15 days after randomization, telephone interview)

10.6 12 months ± 15 days visit

- A neurological evaluation will be performed (Modified Rankin Scale and NIHSS);
- Information will be collected on concomitant medications;
- Information will be collected on new stroke or vascular events since the last visit;
- Information will be collected on adverse events or bleeding events since the last visit;
- Quality of life scale (EQ-5D-5L) will be performed;
- Fill in the research summary.

10.7 Possible event visit

If a patient experiences a potential clinical neurological event, including a clinical deterioration that could be possibly related to ischemia, or new transient or persistent neurological symptoms, an adjudication packet will be produced by the site within 72 hours. This will include the following:

- A physical examination will be performed, including measurement of weight (kg) and vital signs (supine systolic and diastolic blood pressure, heart rate) and neurological examination;
- A neurological evaluation will be performed (Modified Rankin Scale and NIHSS);
- Brain CT or MRI (including T1 + T2 + DWI + FlAIR + ADC + GRE-T2*); imaging data in DICOM format will be transferred to the core imaging evaluation lab. Brain CT is recommended for hemorrhagic stroke and MRI is recommended for ischemic stroke;
- If a patient experiences a new potential cardiac event, a cardiac evaluation (including ECG, myocardial enzymology and TnI) will be performed as clinically indicated. Information supporting a possible myocardial infarction will be collected in an adjudication packet and transmitted to Beijing Tiantan Hospital for further adjudication within 72 hours;
- Information will be collected on concomitant medications and adverse events since the last visit

10.8 Schedule of activities and assessments

Measurements	Screening	Treatment period					
		Randomization Day 1	Discharge	21 days visit D 21±2 days	90 days visit D 90±7 days	Final visit M 12±15 days	Event visit
Demographic characteristics		√					
ABCD^2 score (TIA only)	√						
NIHSS (MIS only)	√		√	√	√	√	√
mRS	√		√	√	√	√	√
Physical examination		√	√	√	√	√	√
Focused medical history		√					
Current medications prior to randomisation		√					
Brain CT/MRI	√						
Laboratory tests	√[1]	√[2]	√[3]				
ECG or holter		√					√
Inclusion/Exclusion	√						
Informed consent signed	√						
Point-of-care genotyping	√						
Dispense and return of study medication		√		√	√		
Standardized brain MRI		√[4]					√
Etiologic diagnosis			√[5]				√
EQ-5D-5L					√	√	√
AE/SAEs			√	√	√	√	√
Compliance/drug accountability			√	√	√		
Concomitant medication			√	√	√	√	√

The cost of screening and baseline auxiliary examination will not be included in the budget of this study.

1. Laboratory tests during screening must be completed: emergency blood routine, emergency liver function (serum transaminase), emergency renal function (serum creatinine), emergency coagulation, and urine HCG test for women of childbearing age. The laboratory test results after the onset time can be used as the screening test results.

2. The blood samples collected on the day of enrollment before taking the study drug should be processed in accordance with the investigator's manual and transported to the clinical resources and biological sample bank of the China National Clinical Research Center for Neurological Diseases.

3. Laboratory examinations during the treatment period should be completed within 7 days after enrollment (including a full set of biochemistry (including liver function, blood lipids, fasting blood glucose, renal function, uric acid, lactate dehydrogenase, creatine kinase, blood electrolytes), glycosylated hemoglobin, homologous Cystine, urine routine, etc.). The laboratory test results after the onset time can be used as the screening test results.

4. Include: T1+T2+DWI+FLAIR+ADC+GRE- T2*/SWI+MRA, completed within 3 days after randomization, collected as DICOM format.

5.All enrolled subjects should undergo vascular assessment within 7 days after enrollment. And investigators need to perform stroke etiology classification according to the TOAST classification.

XI. Study risk pre-assessment and risk management

When subjects take the study drugs, they will be exposed to the risks brought by these drugs, as follows:

1) Side effects of aspirin: bleeding, hematoma, epistaxis, urogenital bleeding, gum bleeding, anemia, hemolysis, blood in urine, indigestion, gastrointestinal and abdominal pain, peptic ulcer and bleeding, kidney injury and acute renal failure, transient transaminase elevation, drug allergy, asthma, urticaria, angioedema or shock, etc.

2) Side effects of ticagrelor: hyperuricemia, hemorrhagic stroke, dyspnea, gastrointestinal bleeding, skin bleeding, urethral bleeding, bleeding at the vascular puncture site, etc.

3) Side effects of clopidogrel: gastrointestinal bleeding, neutropenia, abdominal pain, loss of appetite, gastritis, constipation, skin rash, etc.

Researchers will conduct close observation and follow-up of patients to evaluate clinical safety, as follows：

- Physical examinations on day 1, 21±2, and 90±7 days, including supine blood pressure, heart rate and neurological examination;
- Collect information on adverse events and bleeding events at each visit after the baseline visit;
- When clinical endpoint events or suspected clinical endpoint events happen, a

follow-up visit should be conducted in time according to Chapter 10.7. Study site should submit data of the event for interpretation and adjudication within 72 hours of the event;

- This study does not require other special laboratory tests. However, if the abnormal laboratory test results are related to the study drug, recording, reporting and handling of adverse events shall be carried out in accordance with the following requirements.

If adverse events occur during the study period, investigators will take appropriate treatment according to the study protocol.

11.1 Monitoring of adverse events

All events, whether reported by subjects or investigators, or detected by physical examination or laboratory results, will be managed and collected in compliance with all applicable regulations and will be included in the final Clinical Study Report (CSR).

11.2 Definitions of adverse events

11.2.1 Adverse event (AE)

Adverse Events, regardless of their relationship to Investigational Product, as long as they occur from the first visit planned in the Clinical Trial Protocol/signature of the informed consent (i.e., occurring during the washout period) to the last visit planned in the protocol, are adverse medical events or deterioration of qualifying event. AEs include symptoms (ie, nausea, chest pain), signs (ie, tachycardia, liver enlargement) and abnormal laboratory results (ie, laboratory or ECsG abnormalities). AEs can be classified as serious AEs and non-serious AEs.

11.2.2 Serious adverse event (SAE)

~~A~~ Serious adverse event ~~is~~ refers to any untoward medical occurrence that at any

dose:

- Results in death, or
- Is life-threatening, or

  Note: The term "life-threatening" in the definition of "serious" refers to an event in which the patient is at risk of death at the time of the event; it does not refer to an event which hypothetically might have caused death if it was more severe.

- Requires inpatient hospitalization or prolongation of existing hospitalization, or
- Results in persistent or significant disability/incapacity, or
- Is a congenital anomaly/birth defect, or
- Is a medically important event

11.3 Recording of adverse events

Only some of the non-serious AEs (ie, bleeding events, dyspnea, asthma, renal impairment/increased blood creatinine, bradycardia, increased liver function test indicators, gout/increased uric acid, pneumonia, gynecomastia, abnormal uterine bleeding, malignant tumors) and discontinuations of the study medication due to adverse events (DAEs) will be collected from time of randomization throughout the treatment/follow-up periods to the Study Closure Visit. Other non-serious AEs are up to the investigator to decide whether to collect.

SAE: All SAEs will be collected and recorded.

11.4 Causal relationship between adverse events and study drugs：

Attribution of ：①Definite；②Probably；③Possibly；④Unlikely；⑤Not related；⑥Not accessible. Definitions are as follows：

① **Definite：** Causal relationship is certain (i.e., the temporal relationship between treatment exposure and the adverse event onset/course/resolution is clinically

reasonable; other causes have been eliminated; and the event must be definitive pharmacologically or phenomenologically). The adverse reaction is alleviated or disappeared after the reduction of drug dose or discontinuation of the drug. The reaction reappears when the drug is re-initiated.

② **Probably：** High degree of certainty for causal relationship (i.e., the temporal relationship between treatment exposure and the adverse event onset/course/resolution is clinically reasonable and other causes have been eliminated or are unlikely). The adverse reaction is alleviated or disappeared after the reduction of drug dose or discontinuation of the drug. The subject's clinical status or other reasons could cause this reaction.。

③ **Possibly：** Causal relationship is uncertain (i.e., the temporal relationship between treatment exposure and the adverse event onset/course/resolution is clinically reasonable or unknown; and while other potential causes may or may not exist, a causal relationship to the study treatment does not appear probable). The adverse reaction is alleviated or disappeared after the reduction of drug dose or discontinuation of the drug. The subject's clinical status or other reasons could possibly cause this reaction.

④ **Unlikely：** Not reasonably related, although a causal relationship cannot be ruled out (i.e., while the temporal relationship between treatment exposure and the adverse event onset/course does not preclude causality, there is a clear alternate cause that is more likely to have caused the adverse event than the study treatment). The subject's clinical status or other reasons could possibly cause this reaction.

⑤ **Not related：** No possible relationship (i.e., the temporal relationship between treatment exposure and the adverse event onset/course is unreasonable or

incompatible; or a causal relationship to study treatment is implausible). The subject's clinical status or other reasons could possibly cause this reaction. After clinical symptoms or other causes resolved, the reaction is alleviated or disappeared.

⑥ Not accessible

Total rate of AE will be calculated by summarizing the incidence of adverse drug events in each case report form ①+②+③.

11.5 Safety

11.5.1Adverse events

All Adverse Events will be recorded on the corresponding page(s) in the Case Report Form. Whenever possible, symptoms should be grouped as a single syndrome or diagnosis. The Investigator should specify the date of onset, intensity, action taken with respect to Investigational Product, corrective treatment/therapy given, outcome and his/her opinion about whether it is possible that the Adverse Event is caused by the Investigational Product.

Laboratory, vital signs or ECG abnormalities will be recorded as Adverse Events only if they are medically relevant: symptomatic, requiring corrective treatment, leading to discontinuation and/or fulfilling a seriousness criterion.

11.5.2 Serious adverse event

For serious adverse events, the investigator must immediately take corresponding measures:

Immediately notify the representative of the Monitoring Team, send the signed and dated corresponding pages ~~of~~ in the case report form to the representative of the Monitoring Team, and attach ~~a~~ photocopies of all examinations carried out and the examination dates. For laboratory results, include the laboratory normal ranges. The

contact information (name, address and fax number) of the representative is on the Clinical Trial Protocol. These measures should be completed no later than **24 hours** after SAE.

Care should be taken to ensure that the patient's identity is protected and the patient's identifiers in the Clinical Trial are properly noted on all copies of source documents provided to the Sponsor.

11.5.3 Follow-up and risk management

The Investigator should take all appropriate measures to ensure the safety of the patients.

Screening of subjects should strictly follow the inclusion and exclusion criteria of the study. If an adverse event occurs during the study period, medication will be temporarily discontinued or withdrawn. Relevant evaluations will be performed, including blood routine examination, coagulation, creatinine, hepatic function, renal function, arterial blood gas analysis, ultrasound and computer tomography. Targeted treatment and necessary consultation should be carried out in a timely manner. When dealing with severe adverse events, it is important to make sure patients' airway is clear, respiration, blood pressure and heart rate is steady. Treatment in intensive care unit is recommended when necessary.

Notably, the investigator should follow up the outcome of any Adverse Events (clinical signs, laboratory values or other, etc.) until the patient's condition returns to normal or stabilizes. The follow-up will continue even if the patient withdraws from the clinical trial, and the patient will be interviewed by telephone or face-to-face at the scheduled visit time. The monitoring team may request additional visits and

investigations.

XII. Ethical standards

12.1 Ethical standards

This Clinical Trial will be conducted in accordance with the principles laid down by the 18th World Medical Assembly (Helsinki, 1964) and all applicable amendments laid down by the World Medical Assemblies and the ICH guidelines for Good Clinical Practice. Prior to initiating the study, each site will obtain Institutional review board (IRB) or institutional ethics committee (IEC) approval for the protocol, informed consent forms and materials used to recruit subjects. Before each subject is enrolled, the investigator is responsible for fully and comprehensively introducing the purpose, procedures and possible risks of the study to the subject or his/her agent, signing a written informed consent form, and informing the subject~~s~~ that he has the right to withdraw from this study at any time. The informed consent should be kept as a clinical study document for future reference. The personal privacy and data confidentiality of subjects will be protected during the study process.

12.2 Law and regulations

This Clinical Trial will be conducted in compliance with all international laws and regulations, and Chinese laws and regulations, as well as any applicable guidelines.

12.3 Informed consent

The Investigator/sub-investigator should fully inform the patient of all pertinent aspects of the Clinical Trial, including ~~the~~ written information approved/preferred by the Ethics Committee (IRB/IEC). The Informed Consent Form used by the Investigator for obtaining the patient's informed consent must be reviewed and

approved by the Sponsor and then submitted to the Ethics Committee (IRB/IEC) for approval/favorable opinion.

All participants should be informed to the fullest extent possible about the study, in language and terms they are able to understand. Prior to a patient's participation in the Clinical Trial, nformed Consent Form should be signed and dated by the patient or ~~by~~ the patient's legal representative and ~~by~~ the person who conduct~~ed~~ the informed consent discussion. A copy of the signed and dated Informed Consent Form will be provided to the patient.

12.4 Institutional review board/institutional ethics committee (IRB/IEC)

The Investigator or the Sponsor must submit this Clinical Trial Protocol to the appropriate Ethics Committee (IRB/IEC), and the Ethics Committee is required to forward to the Sponsor a copy of the written approval/favorable opinion signed and dated by the Chairman with Ethics Committee (IRB/IEC) composition. The Clinical Trial (study number, Clinical Trial Protocol title and version number), the documents reviewed (Clinical Trial Protocol, Informed Consent Form, Investigator's Brochure, Investigator's CV, etc.), the list of voting members along with their qualification and the date of the review should be clearly stated on the written (IRB/IEC) approval/favorable opinion.

Investigational Product will not be released at the study site and the Clinical Trial will not start until a copy of signed and dated approval/favorable opinion has been received by the Sponsor.

During the Clinical Trial, any amendment or modification to the Clinical Trial Protocol should be submitted to the Ethics Committee (IRB/IEC). It should also be informed of any event likely to affect the safety of patients or the continued conduct

of the Clinical Trial, in particular any change in safety. All updates to the Investigator's Brochure will be sent to the Ethics Committee (IRB/IEC). If requested, annual progress report, as well as final summary of the Clinical Trial's outcome at the end of the Clinical Trial, will also be sent to the Ethics Committee (IRB/IEC).

XIII. Statistical analysis

The primary null hypothesis of this study is that in patients with TIA or MIS carried *CYP2C19* LOF allele treated with aspirin 75 mg/d, there is no difference in 90-day risk of new stroke (ischemic or hemorrhagic) in those treated with a 3-month regimen of ticagrelor compared with a 3-month regimen of clopidogrel when therapy is initiated within 24 hours of symptom onset.

13.1 Sample size estimation

Primary null hypothesis: in patients with TIA or minor ischemic stroke carried *CYP2C19* LOF allele treated with aspirin 75 mg/d, there is no difference in 90-day risk of stroke (ischemic or hemorrhagic) in those treated with a 3-month regimen of ticagrelor initiated with a loading dose of 180 mg followed by 90 mg bid compared with a 3-month regimen of clopidogrel initiated with a loading dose of 300 mg followed by 75 mg/d when therapy is initiated within 24 hours of symptom onset.

The minimum necessary sample size in the trial is determined by the requirement to detect the smallest expected, clinically meaningful treatment difference comparing the treatment with placebo. Based on the genetic sub-analysis of the CHANCE study, we presume that the 90-day risk of stroke recurrence in *CYP2C19* loss-of-function allele carriers is about 9.4%, and 6.7% for noncarriers. With the point-of-care identification of the *CYP2C19* loss-of-function allele carriers to assess a proper pharmacogenetic approach for patients with high-risk TIA or MIS, we assumed a 25%

relative risk reduction with alteration from clopidogrel to ticagrelor (90-day risk of stroke recurrence: 7.1%). Considering the potential impact of the interim analyses on the probability of type I error, we adjusted the statistical significance to a 2-sided α of 0.048. With a sample size of 6,396 patients, we will have 90% power to detect a relative risk reduction (Ticagrelor: loading dose: 180mg, 90mg bid on day 2-90 vs. Clopidogrel: loading dose: 300mg, 75mg qd on day 2-90) of 25% and 5% dropouts (medication nonadherence). Assuming 58.8% prevalence of *CYP2C19* LOF allele carriers in ~~a~~ Chinese population, we projected screening 10,878 patients would be necessary.

13.2 Data collection and entry

Paper-based case report form (CRF) and electronic data capture (EDC) system will be used for data collection and input. All the content required by the protocol in the system must be filled, the unfilled content should be explained, and the reason needs to be marked in the EDC system.

13.2.1 Paper-based CRF filled out by investigator

Site investigators should use black or blue-black recording pens to fill out the paper-based CRF neatly and clearly to ensure that the data is clear and readable. If the paper-based CRF information needs to be modified, it should not be altered or overwritten. The correct information should be written next to the original information, signed and dated by the person who modified it. The clinical research monitor (CRA) will review the completeness and accuracy of the case report form and guide the investigator to make necessary corrections and supplements.

13.2.2 Data entry to the EDC system by CRC

After the paper-based CRF is completed, the research coordinator (CRC) will input the content of the paper CRF into the EDC system.

13.2.3 Submission to the EDC system after the approval of investigator

The paper-based CRF ~~is~~ will be submitted after the investigator has approves it. After the data is submitted, all data revisions and feedback are carried out through the EDC system. If the EDC system has submitted a form that needs to be modified, contact the CRA of this center. After the CRA opens the form, the investigator can guide the CRC to modify the data in EDC system.

13.2.4 Data monitoring and query by CRA via EDC

13.2.5 Data exportation from the EDC system

After the data from the EDC system is exported to the database, it will be proofread by the data administrator. Obvious errors will be corrected by the data administrator. Other errors or missing values will be filled in the data query form, and the query form will be sent to the participating center for solutions through email, express, telephone and WeChat .

The participating centers are responsible for correcting the data in the EDC system after verifying the original data and related information. Site investigators must answer these queries by verifying or modifying relevant information or data.

13.3 Statistical considerations

This section is an overview of the statistical considerations. It provides the general specifications for the analysis of the data to be collected and presented in the Clinical Study Report. A final SAP will be issued prior to database lockdown and before code breaking. The SAP will define all “pre-specified, planned analyses.”

13.3.1 Analysis sets

Full analysis set (FAS): FAS is the set for efficacy evaluation. All efficacy variables will be analyzed using the FAS. According to the ITT principle, the FAS includes

patients who are enrolled, randomized and have the record of at least one-day treatment of study drugs. Subjects with missing outcome data will be censored at the last follow-up assessment time (end of study or last visit preceding loss to follow up).

Per Protocol set (PPS): PPS includes subjects who completed study treatment without serious violation against protocol. Definition of serious violation against protocol will be confirmed at data review. Generally, it may include the following situations (but not limited to these situations): failure to meet the main inclusion criteria, concomitant treatment that seriously interferes with the evaluation of the efficacy of study drugs after randomization, poor compliance, and follow-up beyond the time window. PPS is the secondary analysis population for efficacy evaluation, ~~but~~ if its results are inconsistent with the full analysis set, detailed analysis of the inconsistent results is required.

Safety analysis set (SS): SS includes all patients who received at least 1 time of study drugs and safety assessment. Throughout the safety results sections, erroneously treated patients (eg, those are randomized to ticagrelor and aspirin group but are actually given clopidogrel and aspirin) will be accounted for in the actual treatment group.

13.3.2 Statistical considerations

1. Balance of baseline characteristics（comparison among groups）

1) Continuous variables will be compared using t test or Wilcoxon rank sum test;

2) Categorical variables will be compared using a $\chi 2$ test, Fisher exact test, or Wilcoxon rank sum test as appropriate.

2、Efficacy analysis

1) Primary efficacy endpoint：

Kaplan-Meier estimates of the cumulative risk of new stroke within 3 months will be reported. The hazard ratio with 95%CI for the treatment comparison will be derived using a Cox's proportional hazards model. The log-rank test will be used to evaluate the statistical significance of the treatment effect.

2) Secondary efficacy endpoint:

① Kaplan-Meier estimates of the cumulative risk of stroke or combined vascular events within 3 months will be reported. The hazard ratio with 95%CI for the treatment comparison will be derived using a Cox's proportional hazards model. The log-rank test will be used to evaluate the statistical significance of the treatment effect;

② ORs and 95%CIs will be compared using $\chi 2$ test or logistic analysis;

③ Continuous variables will be compared using t-test or Wilcoxon sum rank test.

④ Categorical variables will be compared using a $\chi 2$ test, Fisher exact test, or Wilcoxon rank sum test as appropriate;

⑤ Comparison of neurologic deficits scales and EQ-5D-5L scales will be performed using non-parametric analysis;

⑥ Extreme values will be thoroughly checked and corrected before analysis, sensitivity tests will be prespecified;

All statistics will be 2-sided with $P < 0.05$ considered significant.

3) Subgroup analyses:

The primary efficacy endpoint will also be analyzed stratified by age (<65 vs. ≥ 65 years), gender (men vs. women), Body Mass Index (BMI), index event type (TIA vs. minor stroke), time from index event to randomization, etiology subtype, diabetes mellitus, hypertension, type of LOF allele, previous ischemic stroke or TIA, prior

antiplatelet therapy, prior statin therapy, and prior smoking status. Symptomatic intracranial and extracranial artery stenosis will also be evaluated in subgroup analyses.

3. Safety analysis

All patients who receive at least 1 time study drugs and safety assessment will be included in the safety analysis set. The safety evaluation data include adverse events observed during the trial and changes in laboratory data before and after treatment. Adverse events will be summarized using descriptive statistics. And the differences in the primary safety endpoints, secondary safety endpoints and the incidence of various adverse reactions will be compared between the two treatment arms. In addition to comparing the mean of laboratory indicators before and after treatment, we will also list the specific conditions of ~~those been~~ normal before treatment but ~~became~~ abnormal after treatment.

13.4 Interim analysis

Interim analysis will focus on patient recruitment, baseline comparability of treatment arms, sample size assumptions with regard to event rates, loss to follow-up, adverse effects data, and effect of treatment on the primary endpoints. We will plan one interim analysis when 60% of total patients ~~had~~ have undergone randomization and completed follow-up. The sample size will be inflated to account for one interim analyses of the primary efficacy outcome with the use of an O'Brien–Fleming spending function, and a p value of less than 0.008 will be considered significant. In the final analysis, a p value of less than 0.048 will be considered statistically significant. Trial investigators will be blinded to the results of interim analysis.

XIV. Confidentiality and publication of research findings

The principle investigator has complete intellectual property rights. The entire research process and data analysis process will strictly protect the subjects' information. Publication of the results of this trial will be governed by the policies and procedures developed by the Executive Committee. The trial results will be published as soon as possible after database lockdown. This trial will produce detailed data on treatment effects, medical care, and outcomes in a cohort of subjects with TIA or MIS. CHANCE-2 biostatisticians will be consulted to ensure that it is impossible to uniquely identify any participant. Diskettes with the data in comma-delimited text format, along with a data dictionary in a text file, will be sent to interested parties.

XV. Study Organization

15.1 Constitution

- **Principal investigator**

Yongjun Wang, Beijing Tiantan Hospital, Capital Medical University, Beijing, China

- **The steering committee members of CHANCE-2 study**

✓ The steering committee will provide scientific and strategic direction for the trial and will have overall responsibility for its design, execution, and publication.

✓ The steering committee will also be responsible for ensuring that study execution and management are of the highest quality.

✓ It will approve the protocol and the operational guidelines of the trial prior to its commencement.

✓ The steering committee will meet regularly by teleconference or face-to-face meetings to discuss and report the progress of the study.

✓ The composition of the steering committee and its responsibilities are described in

a charter which will be finalized before the start of the trial.

- **Executive committee**

The executive committee is responsible for reviewing the status of the trial and available blinded data and will take appropriate actions regarding the conduct of the study. ~~A~~ Face-to-face Executive Committee meetings will be organized to make major decisions. The composition of the Executive Committee and its responsibilities are described in a charter which will be finalized before the start of the trial.

- **Data safety and monitoring board (DSMB)**

The DSMB will meet regularly and monitor the study progress to ensure that the study meets the highest standards of ethics and patient safety. It is composed of Academic Members, including an independent statistician, who does not otherwise participate in the trial. A DSMB charter including membership, role and responsibilities will be approved by both the DSMB and the Executive Committee before the start of the trial.

Written recommendations and their rationale will be provided to the Chairs of the Steering Committee immediately after each DSMB meeting.

- **Adjudication committee**

Clinical outcome events (stroke, MI, death, overt bleedings) will be reviewed by independent experts (neurologists, cardiologists). An adjudication committee charter including membership, role and responsibilities will be approved before the start of the trial by the Adjudication Committee and the Executive Committee.

Neuroimaging associated with clinical events will be read locally and reports will be included in adjudication packets. The adjudication committee may request actual images from sites or from the core lab in special instances.

15.2 Site training and certification

Executive committee will provide~~d~~ training to their participating sites in Good Clinical Practice Guidelines and in some outcome assessments (e.g., *CYP2C19* genotyping, NIHSS, mRS). Prior to initiation of patient enrollment, Site Investigators and Coordinators must complete all training programs and get their certificates.

The training programs that need to be completed are as follows:

- Study procedures
- Primer on the diagnosis of ischemic stroke or TIA
- Use of the ABCD^2 score
- CHANCE-2 eligibility
- Modified Rankin Scale
- NIHSS
- TOAST etiology subtyping
- *CYP2C19* genotyping
- Clinical outcome events adjudication
- GUSTO bleeding criteria
- EQ-5D-5L
- Collecting blood sample
- Collecting DICOM imaging data

Successful completion of the training program is a must before a site is certified to enroll patients. The conference call will be held intermittently, and PI and key staff will be available to answer questions. Qualification certificates can be obtained in the training centers.

A detailed Manual of Procedures will serve as the primary document describing all

study related procedures. It will serve as a guide to train clinical center personnel and will be updated periodically throughout the study on the CHANCE website, as needed. A system composed of members of executive committee and clinical research associate will be implemented for the clinical centers to ask any procedural questions by phone, fax, or e-mail. The CHANCE-2 executive committee and monitoring committee will formulate answers in consultation with the Steering Committee and will periodically distribute to the participating centers a set of frequently asked questions (FAQ) and answers.

The members of executive committee will manage and conduct site visits to ensure the integrity and validity of the data on the Case Report Forms. During the trial period, each site should be visited at least once. If there are data quality problems or recruitment problems, it should be visited as needed.

XVI. Study monitoring and quality ~~assurance~~ control

16.1 Responsibilities of the investigator(s)

The Investigator(s) should conduct the Clinical Trial in accordance with the Clinical Trial Protocol, ICH guidelines for Good Clinical Practice and the applicable regulatory requirements.

The Investigator is required to ensure compliance with all procedures required by the Clinical Trial Protocol and with all study procedures provided by the Sponsor (including security rules). The Investigator should provide reliable data and all information requested by the Protocol (with the help of the Case Report Form [CRF], Discrepancy Resolution Form [DRF] or other appropriate instruments) in an accurate and legible manner and ensure direct access to source documents (such as drug overdose or pregnancy) by Sponsor representatives.

The Investigator may appoint other individuals as Sub-Investigators, as he thinks appropriate. All Sub-Investigators shall be appointed and listed in a timely manner, and will be supervised by the Investigator. The Investigator will provide them a copy of the Clinical Trial Protocol and all necessary information. The Sponsor is responsible for taking all reasonable steps to ensure the proper conduct of the Clinical Trial Protocol as regards ethics, Clinical Trial Protocol compliance, and integrity and validity of the data on the Case Report Forms.

16.2 Study monitoring

The main responsibility of the monitoring team is to help researchers and sponsors to ensure that all aspects of clinical trials are ethical, scientific, professional, and standardized. According to the ICH guidelines for Good Clinical Practice (GCP), the Monitoring Team must check the Case Report Form entries according to the source documents, except for the pre-identified.

The monitoring team will regularly contact each center through site visits, mails or phone calls, and will send inspectors to evaluate the research progress, adherence of the investigators and patients to the research protocol and to solve urgent problems. During these inspection visits, the inspector will work together with the site-investigators. The main aspects of inspection and monitoring are as follows (not exclusive): patient’s informed consent, patient recruitment and follow-up, documentation and reporting of serious adverse events, study drug supply, adherence to treatment of participants, study drug counting, concomitant treatments and data quality.

XVII. Data retention

The double reviewed case report form and imaging data will be sent to the trial-

designated data management center by clinical research associates (CRAs). The person in charge of the data management center will check and sign the receipt form. The case report form will be kept by the research center after data entry is completed.

XVIII. Data Security Monitoring

The data safety monitoring board (DSMB) established to monitor the safety of participants, protect participants and ensure the integrity of the study. All adverse events should be recorded, handled and tracked until they are properly resolved or stabilized. Any serious adverse events and unexpected events should be reported in a timely manner to the ethics committee in accordance with the relevant provisions, the competent department, the sponsor and the pharmaceutical supervisory and administrative departments. The major researchers should regularly review all adverse events and set up meetings to assess the risks and benefits of the study if necessary. For risks greater than the minimum, separate data examiner will be arranged to monitor the data of the study; for high risks, independent data safety monitoring committee will be appointed to collect safety data, evaluate the effectiveness of data monitoring, and decide whether to make new proposal.

During the clinical trial, the data of the subjects should be collected anonymously in the case report form. The subjects are identified only by the subject number and the abbreviation of the initials. Due to safety reason and administrative instructions, when the subject's identity is leaked, researchers shall share the responsibility of confidentiality. In the informed consent form, the patient allows authorized bidders, ethics committee, and the authority to refer directly to the relevant original data on the case report (such as the patient's medical file case, booking records, the original laboratory records, etc.).The above personnel shall comply with occupational confidentiality rules and must keep all patient's identity and medical information confidential.

XIX. References

1. von Weitzel-Mudersbach P, Andersen G, Hundborg HH, Johnsen SP. Transient ischemic attack and minor stroke are the most common manifestations of acute cerebrovascular disease: A prospective, population-based study--the aarhus tia study. *Neuroepidemiology*. 2013;40:50-55
2. Johnston SC, Gress DR, Browner WS, Sidney S. Short-term prognosis after emergency department diagnosis of tia. *Jama*. 2000;284:2901-2906
3. Rothwell PM, Buchan A, Johnston SC. Recent advances in management of transient ischaemic attacks and minor ischaemic strokes. *The Lancet. Neurology*. 2006;5:323-331
4. Hill MD, Yiannakoulias N, Jeerakathil T, Tu JV, Svenson LW, Schopflocher DP. The high risk of stroke immediately after transient ischemic attack: A population-based study. *Neurology*. 2004;62:2015-2020
5. Ois A, Gomis M, Rodríguez-Campello A, Cuadrado-Godia E, Jiménez-Conde J, Pont-Sunyer C, et al. Factors associated with a high risk of recurrence in patients with transient ischemic attack or minor stroke. *Stroke*. 2008;39:1717-1721
6. Wang Y, Wang Y, Zhao X, Liu L, Wang D, Wang C, et al. Clopidogrel with aspirin in acute minor stroke or transient ischemic attack. *The New England journal of medicine*. 2013;369:11-19
7. Kernan WN, Ovbiagele B, Black HR, Bravata DM, Chimowitz MI, Ezekowitz MD, et al. Guidelines for the prevention of stroke in patients with stroke and transient ischemic attack: A guideline for healthcare professionals from the american heart association/american stroke association. *Stroke; a journal of cerebral circulation*. 2014;45:2160-2236
8. Wang Y, Zhang S, Zhang L, Dong Q, Cui L, Pu C, et al. Chinese guidelines for the secondary prevention of ischemic stroke and transient ischemic attack 2014. *Chin J Neurol*. 2014;48:258-273
9. Wang Y, Zhao X, Lin J, Li H, Johnston SC, Lin Y, et al. Association between cyp2c19 loss-of-function allele status and efficacy of clopidogrel for risk reduction among patients with minor stroke or transient ischemic attack. *JAMA : the journal of the American Medical Association*. 2016;316:70-78

10. Pan Y, Chen W, Xu Y, Yi X, Han Y, Yang Q, et al. Genetic polymorphisms and clopidogrel efficacy for acute ischemic stroke or transient ischemic attack: A systematic review and meta-analysis. *Circulation*. 2017;135:21-33

11. Bonello L, Camoin-Jau L, Armero S, Com O, Arques S, Burignat-Bonello C, et al. Tailored clopidogrel loading dose according to platelet reactivity monitoring to prevent acute and subacute stent thrombosis. *The American journal of cardiology*. 2009;103:5-10

12. Price MJ, Berger PB, Teirstein PS, Tanguay JF, Angiolillo DJ, Spriggs D, et al. Standard- vs high-dose clopidogrel based on platelet function testing after percutaneous coronary intervention: The gravitas randomized trial. *JAMA : the journal of the American Medical Association*. 2011;305:1097-1105

13. Collet JP, Cuisset T, Range G, Cayla G, Elhadad S, Pouillot C, et al. Bedside monitoring to adjust antiplatelet therapy for coronary stenting. *The New England journal of medicine*. 2012;367:2100-2109

14. Cayla G, Cuisset T, Silvain J, Leclercq F, Manzo-Silberman S, Saint-Etienne C, et al. Platelet function monitoring to adjust antiplatelet therapy in elderly patients stented for an acute coronary syndrome (antarctic): An open-label, blinded-endpoint, randomised controlled superiority trial. *Lancet*. 2016

15. Tang YD, Wang W, Yang M, Zhang K, Chen J, Qiao S, et al. Randomized comparisons of double-dose clopidogrel or adjunctive cilostazol versus standard dual antiplatelet in patients with high posttreatment platelet reactivity: Results of the creative trial. *Circulation*. 2018;137:2231-2245

16. Mega JL, Hochholzer W, Frelinger AL, 3rd, Kluk MJ, Angiolillo DJ, Kereiakes DJ, et al. Dosing clopidogrel based on cyp2c19 genotype and the effect on platelet reactivity in patients with stable cardiovascular disease. *Jama*. 2011;306:2221-2228

17. Roberts JD, Wells GA, Le May MR, Labinaz M, Glover C, Froeschl M, et al. Point-of-care genetic testing for personalisation of antiplatelet treatment (rapid gene): A prospective, randomised, proof-of-concept trial. *Lancet (London, England)*. 2012;379:1705-1711

18. Xie X, Ma YT, Yang YN, Li XM, Zheng YY, Ma X, et al. Personalized antiplatelet therapy according to cyp2c19 genotype after percutaneous coronary intervention: A randomized control trial. *International journal of cardiology*. 2013;168:3736-3740

19. Gurbel PA, Bliden KP, Butler K, Tantry US, Gesheff T, Wei C, et al. Randomized double-blind assessment of the onset and offset of the antiplatelet effects of ticagrelor versus clopidogrel in patients with stable coronary artery disease: The onset/offset study. *Circulation*. 2009;120:2577-2585

20. Tantry US, Bliden KP, Wei C, Storey RF, Armstrong M, Butler K, et al. First analysis of the relation between cyp2c19 genotype and pharmacodynamics in patients treated with ticagrelor versus clopidogrel: The onset/offset and respond genotype studies. *Circulation. Cardiovascular genetics*. 2010;3:556-566

21. Wallentin L, Becker RC, Budaj A, Cannon CP, Emanuelsson H, Held C, et al. Ticagrelor versus clopidogrel in patients with acute coronary syndromes. *The New England journal of medicine*. 2009;361:1045-1057

22. James SK, Storey RF, Khurmi NS, Husted S, Keltai M, Mahaffey KW, et al. Ticagrelor versus clopidogrel in patients with acute coronary syndromes and a history of stroke or transient ischemic attack. *Circulation*. 2012;125:2914-2921

23. Wallentin L, James S, Storey RF, Armstrong M, Barratt BJ, Horrow J, et al. Effect of cyp2c19 and abcb1 single nucleotide polymorphisms on outcomes of treatment with ticagrelor versus clopidogrel for acute coronary syndromes: A genetic substudy of the plato trial. *Lancet*. 2010;376:1320-1328

24. Johnston SC, Amarenco P, Albers GW, Denison H, Easton JD, Evans SR, et al. Ticagrelor versus aspirin in acute stroke or transient ischemic attack. *N Engl J Med*. 2016;375:35-43

25. Amarenco P, Albers GW, Denison H, Easton JD, Evans SR, Held P, et al. Efficacy and safety of ticagrelor versus aspirin in acute stroke or transient ischaemic attack of atherosclerotic origin: A subgroup analysis of socrates, a randomised, double-blind, controlled trial. *Lancet Neurol*. 2017

26. Wang Y, Minematsu K, Wong KS, Amarenco P, Albers GW, Denison H, et al. Ticagrelor in acute stroke or transient ischemic attack in asian patients: From the socrates trial (acute stroke or transient ischemic attack treated with aspirin or ticagrelor and patient outcomes). *Stroke; a journal of cerebral circulation*. 2017;48:167-173

27. Wang Y, Chen W, Lin Y, Meng X, Chen G, Wang Z, et al. Ticagrelor plus aspirin versus clopidogrel plus aspirin for platelet reactivity in patients with minor stroke or transient ischaemic attack: Open label, blinded endpoint, randomised controlled phase ii trial. *BMJ (Clinical research ed.)*. 2019;365:l2211

Appendix Table 1. Definitions of stroke events and vascular events

**Stroke**	Acute symptoms and signs of neurologic defect caused by sudden abnormality of the blood supply. Damage of focal or whole brain, spinal or retinal vascular damage, which is related to cerebral circulation disorder.
**Ischemic stroke**	Definitions: (1) Symptoms or imaging evidence of acute newly onset focal neurologic deficit last for more than 24 hours after excluding other non-ischemic reasons, such as brain infection, head trauma, brain tumor, epilepsy, severe metabolic diseases, degeneration diseases or adverse effect of medications; or (2) Acute brain or retinal ischemic event with focal symptoms or signs lasts for less than 24 hours after excluding other causes with imaging evidence of new infarction; or (3) Progression of original vascular ischemic stroke (NIHSS increased≥4 from baseline score after excluding hemorrhagic transformation or symptomatic intracerebral hemorrhage after cerebral infarction) lasts over 24 hours with new ischemic lesion on brain MRI or CT. Which would be classified by TOAST etiology standard.
**Transient ischemic attack**	A brief episode of neurological dysfunction caused by focal brain or retinal ischemia, with clinical symptoms typically lasting less than 24 hours, and without evidence of acute infarction, after excluding other non-ischemic reasons, such as brain infection, head trauma, brain tumor, epilepsy, severe metabolic diseases, degeneration diseases or adverse effect of medications.
**Hemorrhagic stroke**	Hemorrhagic stroke was defined as focal or whole brain or spine damage caused by non-traumatic bleeding into the brain parenchyma, intraventricular or subarachnoid.
**Hemorrhagic transformation after**	Any kind of non-traumatic extravascular bleeding in the area of brain tissue of existing acute/subacute infarction. Which could lead to relevant neurologic

**infarction**	symptoms(symptomatic) or not (asymptomatic).<br>1. To determine a symptomatic hemorrhagic transformation, the following two conditions must be met，（1）Image evidence of extravascular bleeding in the area of infarction on brain CT or MRI; (2) Clinical symptoms are relevant to hemorrhagic transformation. Hemorrhagic transformation can partly explain patients′ neurological symptoms, for example: (a) The area and location of infarction can′t explain the symptoms; (b) Clinically deterioration means NIHSS increased ≥ 4 from original ischemic event or causing death directly from hemorrhagic transformation; (c) a large space-occupying hematoma develops secondary to hemorrhagic transformation.<br>2. To determine an asymptomatic hemorrhagic transformation, the following two conditions must be met, (1) Image evidence of extravascular bleeding in the area of infarction on brain CT or MRI; (2) No symptoms are caused by hemorrhagic transformation or an neurologic deterioration that lead to a less than 4 increase of NIHSS score.
**Myocardial infarction**	Third universal definition of myocardial infarction（Thygesen 2012）<br>The term acute myocardial infarction (MI) should be used when there is evidence of myocardial necrosis in a clinical setting consistent with acute myocardial<br>ischemia. Under these conditions any one of the following criteria meets the diagnosis for MI:<br>1、 Detection of a rise and/or fall of cardiac biomarker values [preferably cardiac troponin (cTn)] with at least one value above the 99th percentile upper reference<br>limit (URL) and with at least one of the following:<br>(1) Symptoms of ischemia.<br>(2) New or presumed new significant ST-segment–T wave (ST–T) changes or new left bundle branch block (LBBB).

	(3) Development of pathological Q waves in the ECG.<br>(4) Imaging evidence of new loss of viable myocardium or new regional wall motion abnormality<br>(5) Identification of an intracoronary thrombus by angiography or autopsy.<br>2、Cardiac death with symptoms suggestive of myocardial ischemia and presumed new ischemic ECG changes or new LBBB, but death occurred before cardiac<br>biomarkers were obtained, or before cardiac biomarker values would be increased.<br>3、Percutaneous coronary intervention (PCI) related MI is arbitrarily defined by elevation of cTn values (>5×99th percentile URL) in patients with normal baseline<br>values (≤99th percentile URL) or a rise of cTn values >20% if the baseline values are elevated and are stable or falling. In addition, either (1) symptoms<br>suggestive of myocardial ischemia or (2) new ischemic ECG changes or (3) angiographic findings consistent with a procedural complication or (4) imaging<br>demonstration of new loss of viable myocardium or new regional wall motion abnormality are required。<br>4、Stent thrombosis associated with MI when detected by coronary angiography or autopsy in the setting of myocardial ischemia and with a rise and/or fall of cardiac<br>biomarker values with at least one value above the 99th percentile URL.<br>5、Coronary artery bypass grafting (CABG) related MI is arbitrarily defined by elevation of cardiac biomarker values (>10×99th percentile URL) in patients with<br>normal baseline cTn values (≤99th percentile URL). In addition, either<br>(1) new pathological Q waves or new LBBB, or<br>(2) angiographic documented new graft or new native coronary artery occlusion, or

	(3) imaging evidence of new loss of viable myocardium or new regional wall motion abnormality.
**Vascular death**	Vascular death include death due to stroke, cardiac sudden death, death caused by acute myocardial infarction, death caused by heart failure, death caused by pulmonary embolism, death caused by cardial/cerebral interventions or operations (not caused by myocardial infarction) and death caused by other cardiovascular diseases. (Arrhythmia irrelevant to cardiac sudden death, rupture of aortic aneurysm or peripheral artery disease).<br>Unexplained death happened within 30 days after stroke, myocardial infarction or cardiovascular/cerebral vascular operation will be considered as stroke, myocardial infarction and accidental death caused by operation separately.

Appendix Table 2. Modified Rankin Scale

The modified Rankin scale is used to measure the results of patients' functional recovery after stroke. Bold typeface shows the formal definition of each level. The italics give further guidance in order to reduce the possible errors between different observers, but there is no requirement for the structure of the interview. Please note that only symptoms that have occurred since the stroke are considered. If the patient can walk with the help of some assistive devices without outside help, it is considered to be able to walk independently.

If the two levels seem to be equally applicable to the patient, and further questions are unlikely to make an absolutely correct choice, the more severe level should be selected.

0- No symptoms at all

There may be mild symptoms. But no noticeable new functional limitations or new symptoms after stroke event.

1- No significant disability despite symptoms; able to carry out all usual duties and activities

No significant disability: symptoms present but no other limitations. Question: Does the person have difficulty reading or writing, difficulty speaking or finding the right word, problems with balance or coordination, visual problems, numbness (face, arms, legs, hands, feet), loss of movement (face, arms, legs, hands, feet), difficulty with swallowing, or other symptom resulting from stroke?

2- Slight disability; unable to carry out all previous activities but able to look after own affairs without assistance

Slight disability; limitations in participation in usual social roles, but independent for ADL. Questions: Has there been a change in the person’s ability to work or look after others if these were roles before stroke? Has there been a change in the person′ s ability to participate in previous social and leisure activities? Has the person had problems with relationships or become isolated?

3- Moderate disability; requiring some help, but able to walk without assistance

Moderate disability; need for assistance with some instrumental ADL but not basic ADL. Question: Is assistance essential for preparing a simple meal, doing household chores, looking after money, shopping, or traveling locally?

4- Moderately severe disability; unable to walk without assistance, and unable to attend to own bodily needs without assistance

Moderately severe disability: need for assistance with some basic ADL, but not requiring constant care. Question: Is

assistance essential for eating, using the toilet, daily hygiene, or walking?

5- Severe disability: bedridden, incontinent, and requiring constant nursing care and attention

Severe disability: someone needs to be available at all times; care may be provided by either a trained or an untrained caregiver. Question: Does the person require constant care?

Appendix Table 3. National institutes of health stroke scale

Administer stroke scale items in the order listed. Record performance in each category after each subscale exam. Do not go back and change scores. Follow directions provided for each exam technique. Scores should reflect what the patient does, not what the clinician thinks the patient can do. The clinician should record answers while administering the exam and work quickly. Except where indicated, the patient should not be coached (i.e., repeated requests to patient to make a special effort).

	Instructions	Scale Definition	Score
**1a**	Level of consciousness: The investigator must choose a response if a full evaluation is prevented by such obstacles as an endotracheal tube, language barrier, orotracheal trauma/bandages. A 3 is scored only if the patient makes no movement (other than reflexive posturing) in response to noxious stimulation	0 = Alert; keenly responsive.<br>1 = Not alert; but arousable by minor stimulation to obey, answer, or respond.<br>2 = Not alert; requires repeated stimulation to attend or is obtunded and requires strong or painful stimulation to make movements (not stereotyped). 3 = Responds only with reflex motor or autonomic effects or totally unresponsive, flaccid, and areflexic.	_____
**1b**	LOC Questions: The patient is asked the month and his/her age. The answer must be correct - there is no partial credit for being close. Aphasic and stuporous patients who do not comprehend the questions will score 2. Patients unable to speak because of endotracheal intubation, orotracheal trauma, severe dysarthria from any cause, language barrier, or any other problem not secondary to aphasia are given a 1. It is important that only the initial answer be graded and that the examiner not "help" the patient with verbal or non-verbal cues.	0 = Answers both questions correctly.<br>1 = Answers one question correctly.<br>2 = Answers neither question correctly.	_____
**1c**	LOC Commands: The patient is asked to open and close the eyes and then to grip and release the non-paretic hand. Substitute another one step command if the hands cannot be used. Credit is given if an unequivocal attempt is made but not completed due to weakness. If the patient does not respond to command, the task should be demonstrated to him or her (pantomime), and the result scored (i.e., follows none, one or two commands). Patients with trauma, amputation, or other physical impediments should be given suitable one-step commands. Only the first attempt is scored.	0 = Performs both tasks correctly.<br>1 = Performs one task correctly.<br>2 = Performs neither task correctly.	_____
**2**	Best Gaze: Only horizontal eye movements will be tested. Voluntary or reflexive (oculocephalic) eye movements will be scored, but caloric testing is not done. If the patient has a conjugate deviation of the eyes that can be overcome by voluntary or reflexive activity, the score will be 1. If a patient has an isolated peripheral nerve paresis (CN III, IV or VI), score a 1. Gaze is testable in all aphasic	0 = Normal.<br>1 = Partial gaze palsy; gaze is abnormal in one or both eyes, but forced deviation or total gaze paresis is not present.<br>2 = Forced deviation, or total gaze paresis not overcome by the oculocephalic maneuver.	_____

	Instructions	Scale Definition	Score
	patients. Patients with ocular trauma, bandages, pre-existing blindness, or other disorder of visual acuity or fields should be tested with reflexive movements, and a choice made by the investigator. Establishing eye contact and then moving about the patient from side to side will occasionally clarify the presence of a partial gaze palsy.		
**3**	Visual: Visual fields (upper and lower quadrants) are tested by confrontation, using finger counting or visual threat, as appropriate. Patients may be encouraged, but if they look at the side of the moving fingers appropriately, this can be scored as normal. If there is unilateral blindness or enucleation, visual fields in the remaining eye are scored. Score 1 only if a clear-cut asymmetry, including quadrantanopia, is found. If patient is blind from any cause, score 3. Double simultaneous stimulation is performed at this point. If there is extinction, patient receives a 1, and the results are used to respond to item 11.	0 = No visual loss.<br>1 = Partial hemianopia.<br>2 = Complete hemianopia.<br>3 = Bilateral hemianopia (blind including cortical blindness).	_____
**4**	Facial Palsy: Ask – or use pantomime to encourage – the patient to show teeth or raise eyebrows and close eyes. Score symmetry of grimace in response to noxious stimuli in the poorly responsive or non-comprehending patient. If facial trauma/bandages, orotracheal tube, tape or other physical barriers obscure the face, these should be removed to the extent possible.	0 = Normal symmetrical movements.<br>1 = Minor paralysis (flattened nasolabial fold, asymmetry on smiling).<br>2 = Partial paralysis (total or near-total paralysis of lower face).<br>3 = Complete paralysis of one or both sides (absence of facial movement in the upper and lower face).	_____
**5**	Motor Arm: The limb is placed in the appropriate position: extend the arms (palms down) 90 degrees (if sitting) or 45 degrees (if supine). Drift is scored if the arm falls before 10 seconds. The aphasic patient is encouraged using urgency in the voice and pantomime, but not noxious stimulation. Each limb is tested in turn, beginning with the non-paretic arm. Only in the case of amputation or joint fusion at the shoulder, the examiner should record the score as untestable (UN), and clearly write the explanation for this choice.	0 = No drift; limb holds 90 (or 45) degrees for full 10 seconds.<br>1 = Drift; limb holds 90 (or 45) degrees, but drifts down before full 10 seconds; does not hit bed or other support.<br>2 = Some effort against gravity; limb cannot get to or maintain (if cued) 90 (or 45) degrees, drifts down to bed, but has some effort against gravity.<br>3 = No effort against gravity; limb falls.<br>4 = No movement.<br>UN = Amputation or joint fusion, explain: _____	5a Left Arm<br><br>5b Right Arm
**6**	Motor Leg: The limb is placed in the appropriate position: hold the leg at 30 degrees (always tested supine). Drift is scored if the leg falls before 5 seconds. The aphasic patient is encouraged using urgency in the voice and pantomime, but not noxious stimulation. Each limb is tested in turn, beginning with the non-paretic leg. Only in the case of	0 = No drift; leg holds 30-degree<br><br>position for full 5 second<br><br>1 = Drift; leg falls by the end of the 5-	6a Left Leg<br><br>6b Right Leg

	Instructions	Scale Definition	Score
	amputation or joint fusion at the hip, the examiner should record the score as untestable (UN), and clearly write the explanation for this choice.	second period but does not hit bed.<br>2 = Some effort against gravity; leg falls to bed by 5 seconds but has some effort against gravity.<br>3 = No effort against gravity; leg falls to bed immediately.<br>4 = No movement.<br>UN = Amputation or joint fusion, explain: ________________	
**7**	Limb Ataxia: This item is aimed at finding evidence of a unilateral cerebellar lesion. Test with eyes open. In case of visual defect, ensure testing is done in intact visual field. The finger-nose-finger and heel-shin tests are performed on both sides, and ataxia is scored only if present out of proportion to weakness. Ataxia is absent in the patient who cannot understand or is paralyzed. Only in the case of amputation or joint fusion, the examiner should record the score as untestable (UN), and clearly write the explanation for this choice. In case of blindness, test by having the patient touch nose from extended arm position.	0 = Absent.<br>1 = Present in one limb.<br>2 = Present in two limbs.<br>UN = Amputation or joint fusion, explain: ________________	_____
**8**	Sensory: Sensation or grimace to pinprick when tested, or withdrawal from noxious stimulus in the obtunded or aphasic patient. Only sensory loss attributed to stroke is scored as abnormal and the examiner should test as many body areas (arms [not hands], legs, trunk, face) as needed to accurately check for hemisensory loss. A score of 2, "severe or total sensory loss," should only be given when a severe or total loss of sensation can be clearly demonstrated.<br>Stuporous and aphasic patients will, therefore, probably score 1 or 0. The patient with brainstem stroke who has bilateral loss of sensation is scored 2. If the patient does not respond and is quadriplegic, score 2. Patients in a coma (item 1a=3) are automatically given a 2 on this item	0 = Normal; no sensory loss.<br>1 = Mild-to-moderate sensory loss; patient feels pinprick is less sharp or is dull on the affected side; or there is a loss of superficial pain with pinprick, but patient is aware of being touched.<br>2 = Severe to total sensory loss; patient is not aware of being touched in the face, arm, and leg.	_____
**9**	Best Language: A great deal of information about comprehension will be obtained during the preceding sections of the examination. For this scale item, the patient is asked to describe what is happening in the	0 = No aphasia; normal.<br>1 = Mild-to-moderate aphasia; some obvious loss of fluency or facility of comprehension, without significant	____

	Instructions	Scale Definition	Score
	attached picture, to name the items on the attached naming sheet and to read from the attached list of sentences.<br>Comprehension is judged from responses here, as well as to all of the commands in the preceding general neurological exam. If visual loss interferes with the tests, ask the patient to identify objects placed in the hand, repeat, and produce speech. The intubated patient should be asked to write. The patient in a coma (item 1a=3) will automatically score 3 on this item. The examiner must choose a score for the patient with stupor or limited cooperation, but a score of 3 should be used only if the patient is mute and follows no one-step commands.	limitation on ideas expressed or form of expression. Reduction of speech and/or comprehension, however, makes conversation about provided materials difficult or impossible. For example, in conversation about provided materials, examiner can identify picture or naming card content from patient's response.<br>2 = Severe aphasia; all communication is through fragmentary expression; great need for inference, questioning, and guessing by the listener. Range of information that can be exchanged is limited; listener carries burden of communication. Examiner cannot identify materials provided from patient response.<br>3 = Mute, global aphasia; no usable speech or auditory comprehension.	
**10**	Dysarthria: If patient is thought to be normal, an adequate sample of speech must be obtained by asking patient to read or repeat words from the attached list. If the patient has severe aphasia, the clarity of articulation of spontaneous speech can be rated. Only if the patient is intubated or has other physical barriers to producing speech, the examiner should record the score as untestable (UN), and clearly write an explanation for this choice. Do not tell the patient why he or she is being tested	0 = Normal.<br>1 = Mild-to-moderate dysarthria; patient slurs at least some words and, at worst, can be understood with some difficulty.<br>2 = Severe dysarthria; patient's speech is so slurred as to be unintelligible in the absence of or out of proportion to any dysphasia, or is mute/anarthric.<br>UN = Intubated or other physical barrier, explain:___	____
**11**	Extinction and Inattention (formerly Neglect): Sufficient information to identify neglect may be obtained during the prior testing. If the patient has a severe visual loss preventing visual double simultaneous stimulation, and the cutaneous stimuli are normal, the score is normal. If the patient has aphasia but does appear to attend to both sides, the score is normal. The presence of visual spatial neglect or anosagnosia may also be taken as evidence of abnormality. Since the abnormality is scored only if present, the item is never untestable.	0 = No abnormality.<br>1 = Visual, tactile, auditory, spatial, or personal inattention or extinction to bilateral simultaneous stimulation in one of the sensory modalities.<br>2 = Profound hemi-inattention or extinction to more than one modality; does not recognize own hand or orients to only one side of space.	____
**Total Score**			____

Appendix：1. Pictures for item 9-10

Picture 1

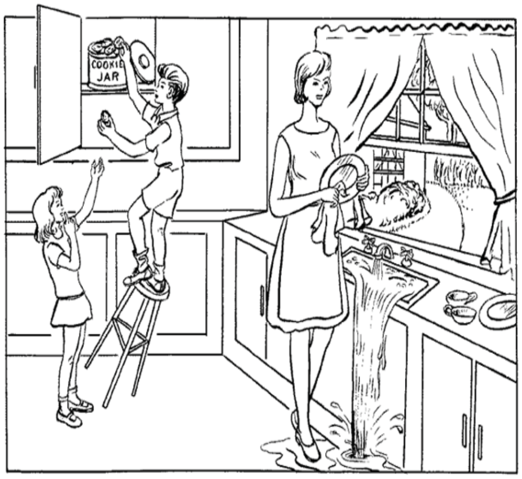

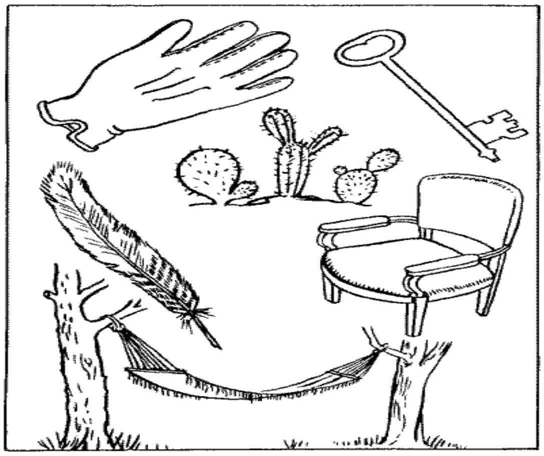

Picture 2

Picture 3

Picture 4

**Please read the following scentences：**
**You know how.**
**Down to earth**
**I got home from work**
**Near the table in the dining room.**
**They heard him speak on the radio last night.**

**Please read the following scentences：**
**You know how.**
**Down to earth**
**I got home from work**
**Near the table in the dining room.**
**They heard him speak on the radio last night.**

Appendix：2. How to evaluate the NIH stroke scale for coma patients?

In patients with a score of 1a item less than 3, each category of the score should be evaluated. A 3 is scored only if the patient makes no movement (other than reflexive posturing) in response to noxious stimulation (sternum friction or orbit pressing). The other categories should be scored as the followings:

- **1b- LOC Questions：2**
- **1c- LOC Commands：2**
- **2-Best gaze：1 = Partial gaze palsy; gaze is abnormal in one or both eyes but forced deviation or total gaze paresis is not present. 2 = Forced deviation, or total gaze paresis not overcome by the oculocephalic maneuver.**
- **3-Visual：Using visual threat to evaluate.**
- **4-Facial palsy：3**
- **5、6-Motor arm/leg：4 for each limb**
- **7- Limb Ataxia：This category can be scored only when the patient has limb ataxia. If the finger-nose-finger and heel-shin tests could not be tested due to limb weakness, then score 0.**
- **8-Sensory：2**
- **9-Best language：3**
- **10-Dysathria：2**
- **11- Extinction and Inattention： Coma means losing all kinds of cognitive abilities, then score 2.**

3、How to calculate the total score of NIH stroke score?

The following items will not be calculated into the total score:

- **The fifth, sixth item: UN = Amputation or joint fusion**

  The seventh item: - Limb ataxia presents in which limb.

Appendix Table 4. EQ-5D-5Lindex

By placing a tick in one box in each group below, please indicate which statements best describe your own health state today.

**Mobility**	
I have no problems in walking about	☐
I have slight problems in walking about	☐
I have moderate problems in walking about	☐
I have severe problems in walking about	☐
I am unable to walk about	☐
**Self-care**	
I have no problems with washing or dressing myself	☐
I have slight problems with washing or dressing myself	☐
I have moderate problems with washing or dressing myself	☐
I have severe problems with washing or dressing myself	☐
I am unable to wash or dress myself	☐
**Usual activities (e.g. work, study, housework, family or leisure activities)**	
I have no problems doing my usual activities	☐
I have slight problems doing my usual activities	☐
I have moderate problems doing my usual activities	☐
I have severe problems doing my usual activities	☐
I am unable to do my usual activities	☐
**Pain/discomfort**	
I have no pain or discomfort	☐
I have slight pain or discomfort	☐
I have moderate pain or discomfort	☐
I have severe pain or discomfort	☐
I have extreme pain or discomfort	☐
**Anxiety/depression**	
I am not anxious or depressed	☐
I am slightly anxious or depressed	☐
I am moderately anxious or depressed	☐
I am severely anxious or depressed	☐
I am extremely anxious or depressed	☐

Illustrations

（1）Please choose only one level under each dimension.

（2）Please fill in the form by participants. The interviewer should avoid obvious guiding questions

and should not chose from his/her own subjective judgement after listening to the participant's narration. The interviewer should not express his/her guiding views to the participant.

To help people say how good or bad a health state is, we have drawn a scale (rather like a thermometer) on which the best state you can imagine is marked 100 and the worst state you can imagine is marked 0.

We would like you to indicate on this scale how good or bad your own health is today, in your opinion. Please do this by drawing a line from the box below to whichever point on the scale indicates how good or bad your health state is today.

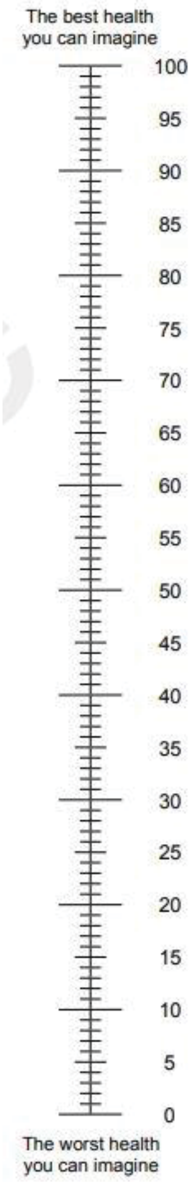

How good or bad your own health is today

Questionnaire completed by:

○1Patients independently

○2Patients with help of others

○3Agent (the patient' s family members)

Appendix Table 5. Abnormal clinical laboratory indicators

Hemorrhagic tendency	The prothrombin time was 1.5 times longer than that of the normal control group or platelet < 10×10^9/L.
Moderate/severe anemia	Hemoglobin (Hb)<90g/L
Hepatic insufficiency	Aminotransaminase exceeded twice the upper limit of standard level
Renal insufficiency	Serum creatinine > 1.5mg/dl or creatinine clearance rate < 50ml/min

Appendix Table 6. Global Utilization of Streptokinase and Tissue Plasminogen Activator for Occluded Coronary Arteries (GUSTO) bleeding definition

Severe bleeding	Including life-threatening bleeding, primary intracranial hemorrhage, post-traumatic symptomatic intracranial hemorrhage that resulted in substantial hemodynamic compromise requiring treatment (blood transfusion, liquid transition, vasoconstrictor medication or surgical intervention)
Moderate bleeding	Moderate bleeding was defined by the need for transfusion, but does not reach the standard as severe bleeding; including reduction of absolute value of hemoglobin or red blood cells, causing severe disability or intraocular hemorrhage with severe visual field defect, etc.
Minor bleeding	Minor bleeding referred to other bleeding, not requiring transfusion or causing hemodynamic compromise. Including bleeding at the puncture site or hematoma, etc.

Summary of protocol amendments

APPENDIX I:

Protocol changes version 1.0 (Jan 09, 2019) to 1.1 (April 28, 2019).

Protocol Version 1.0	Protocol Version 1.1
	Official Title： A multicenter, double-blind, placebo-controlled, randomized clinical trial comparing the efficacy and safety of ticagrelor/aspirin and clopidogrel/aspirin in preventing stroke of patients with high-risk non-disabling cerebrovascular events who carry the CYP2C19 loss-of-function allele
Multicenter, prospective, double-blind, placebo-controlled, randomized clinical trial	Multicenter, double-blind, randomized clinical trial
**Exclusion Criteria**<br>1. Malformation, tumor, abscess or other major non-ischemic brain disease (e.g., multiple sclerosis) on baseline head CT or MRI.<br>2. Isolated or pure sensory symptoms (e.g., numbness), isolated visual	**Exclusion Criteria**<br>1. Malformation, tumor, abscess or other major non-ischemic brain disease (e.g., multiple sclerosis) on baseline head CT or MRI.<br>2. Isolated or pure sensory symptoms (e.g., numbness), isolated visual

changes, or isolated dizziness/vertigo without evidence of acute infarction on baseline head CT or MRI.	changes, or isolated dizziness/vertigo without evidence of acute infarction on baseline head CT or MRI.
3. Preceding moderate or severe dependency (modified Rankin scale [mRS] score 3-5).	3. Preceding moderate or severe dependency (modified Rankin scale [mRS] score 3-5).
4. Contraindication to clopidogrel, ticagrelor or aspirin:	4. Contraindication to clopidogrel, ticagrelor or aspirin:
1) Known allergy	1) Known allergy
2) Severe renal (creatinine exceeding 1.5 times of the upper limit of normal range) or hepatic (ALT or AST > twice the upper limit of normal range) insufficiency	2) Severe renal (creatinine exceeding 1.5 times of the upper limit of normal range) or hepatic (ALT or AST > twice the upper limit of normal range) insufficiency
3) Severe cardiac failure (NYHA level: III to IV)	3) Severe cardiac failure (NYHA level: III to IV)
4) History of hemostatic disorder or systemic bleeding	5) History of hemostatic disorder or systemic bleeding
5) History of thrombocytopenia or neutropenia	5) History of thrombocytopenia or neutropenia
6)History of drug-induced hematologic disorder or hepatic dysfunction	6)History of drug-induced hematologic disorder or hepatic dysfunction
7) Low white blood cell ($<2\times10^9$/L) or platelet count ($<100\times10^9$/L)	7) Low white blood cell ($<2\times10^9$/L) or platelet count ($<100\times10^9$/L)
5..Clear indication for anticoagulation (presumed cardiac source of embolus, e.g., atrial fibrillation, prosthetic cardiac valves known or suspected endocarditis)	5.Hematocrit (HCT) <30%
6. Clopidogrel or ticagrelor has been used continuously for ≥5 days before	6.Clear indication for anticoagulation (presumed cardiac source of embolus, e.g., atrial fibrillation, prosthetic cardiac valves known or suspected endocarditis)

enrollment.<br>7. Used heparin or oral anticoagulant drugs within 10 days before enrollment.<br>8. Undergone intravenous or arterial thrombolysis and mechanical thrombectomy within 24 hours before enrollment.<br>9 History of intracranial hemorrhage or amyloid angiopathy.<br>10. History of aneurysm (including intracranial aneurysm and peripheral aneurysm).<br>11. Diagnosis or suspicious diagnosis of acute coronary syndrome<br>12. History of asthma or COPD (chronic obstructive pulmonary disease).<br>13. High-risk for bradyarrhythmia (first-degree or second-degree AV block caused by sinus node disease, and brady-arrhythmic syncope without pacemaker).<br>14. History of hyperuricemia nephropathy.<br>15. Anticipated requirement for long-term (>7 days) non-steroidal anti-inflammatory drugs (NSAIDs).<br>16. History of gastrointestinal bleeding within 3 months before enrollment or major surgery within 30 days.	7.Iatrogenic causes (angioplasty or surgery) of minor stroke or TIA.<br>8.Anticipated requirement for long-term (>7 days) non-steroidal anti-inflammatory drugs (NSAIDs).<br>9. History of intracranial hemorrhage or amyloid angiopathy.<br>10. History of aneurysm (including intracranial aneurysm and peripheral aneurysm).<br>11. Diagnosis or suspicious diagnosis of acute coronary syndrome<br>12. History of asthma or COPD (chronic obstructive pulmonary disease).<br>13. High-risk for bradyarrhythmia (first-degree or second-degree AV block caused by sinus node disease, and brady-arrhythmic syncope without pacemaker).<br>14. History of hyperuricemia nephropathy （in the case of a purine diet, the serum uric acid >420umol/L (7.0mg/dl) for men and menopausal women on two different days on a non-same day , >357umol/L (6.0mg/dl)) for premenopausal women）<br>15.Severe non-cardiovascular comorbidity with life expectancy < 3 months.<br>16.Inability to understand and/or follow research procedures due to mental,

17. Iatrogenic causes (angioplasty or surgery) of minor stroke or TIA.<br>18. Planned or likely revascularization (any angioplasty or vascular surgery) within the next 3 months.<br>19. Scheduled for surgery or interventional treatment requiring study drug cessation.<br>20. Severe non-cardiovascular comorbidity with life expectancy < 3 months.<br>21. Women of childbearing age who have not taken effective contraceptive measures and have a positive pregnancy test record, as well as women who are pregnant or breastfeeding.<br>22. Currently receiving an experimental drug or device.<br>23. Participation in another clinical study with an experimental product during the last 30 days.<br>24. Inability to understand and/or follow research procedures due to mental, cognitive, or emotional disorders.<br>25. Hematocrit (HCT) <30%.	cognitive, or emotional disorders.<br>17.Clopidogrel or ticagrelor has been used continuously for ≥5 days before enrollment.<br>18.Used heparin or oral anticoagulant drugs within 10 days before enrollment.<br>19.Undergone intravenous or arterial thrombolysis and mechanical thrombectomy within 24 hours before enrollment.<br>20. History of gastrointestinal bleeding within 3 months before enrollment or major surgery within 30 days.<br>21. Planned or likely revascularization (any angioplasty or vascular surgery) within the next 3 months.<br>22. Scheduled for surgery or interventional treatment requiring study drug cessation.<br>23. Women of childbearing age who have not taken effective contraceptive measures and have a positive pregnancy test record, as well as women who are pregnant or breastfeeding.<br>24. Currently receiving an experimental drug or device.<br>25. Participation in another clinical study with an experimental product during

	the last 30 days.

APPENDIX II:

Protocol changes version 1.1 (April 28, 2019) to 1.2 (July 17, 2019).

**Protocol Version 1.1**	**Protocol Version 1.2**
**Inclusion Criteria**<br>1. 40 years or older than 40 years and less than 80 years；<br>2. Acute cerebral ischemic event due to：<br>● Acute non-disabling ischemic stroke (NIHSS≤3 at the time of randomization)or，<br>● TIA with moderate-to-high risk of stroke (ABCD2 score ≥ 4 at the time of randomization) or responsible vessel stenosis more than 50%;	**Inclusion Criteria**<br>1. 40 years old and above；<br>2. Acute cerebral ischemic event due to：<br>● Acute non-disabling ischemic stroke (NIHSS≤3 at the time of randomization)or，<br>● TIA with moderate-to-high risk of stroke (ABCD2 score ≥ 4 at the time of randomization) ;
**Exclusion Criteria**<br>1. Malformation, tumor, abscess or other major non-ischemic brain disease	**Exclusion Criteria**<br>1. Malformation, tumor, abscess or other major non-ischemic brain disease

(e.g., multiple sclerosis) on baseline head CT or MRI.<br>2. Isolated or pure sensory symptoms (e.g., numbness), isolated visual changes, or isolated dizziness/vertigo without evidence of acute infarction on baseline head CT or MRI.<br>3. Preceding moderate or severe dependency (modified Rankin scale [mRS] score 3-5).<br>4. Contraindication to clopidogrel, ticagrelor or aspirin:<br>1) Known allergy<br>2) Severe renal (creatinine exceeding 1.5 times of the upper limit of normal range) or hepatic (ALT or AST > twice the upper limit of normal range) insufficiency<br>3) Severe cardiac failure (NYHA level: III to IV)<br>6) History of hemostatic disorder or systemic bleeding<br>5) History of thrombocytopenia or neutropenia<br>6)History of drug-induced hematologic disorder or hepatic dysfunction<br>7) Low white blood cell (<2×10^9/L) or platelet count (<100×10^9/L)<br>5.Hematocrit (HCT) <30%	(e.g., multiple sclerosis) on baseline head CT or MRI.<br>2. Isolated or pure sensory symptoms (e.g., numbness), isolated visual changes, or isolated dizziness/vertigo without evidence of acute infarction on baseline head CT or MRI.<br>3. Iatrogenic causes (angioplasty or surgery) of minor stroke or TIA.<br>4. Preceding moderate or severe dependency (modified Rankin scale [mRS] score 3-5).<br>[Pre-onset mRS score [Note: According to the evaluation of the patient′ s pre-onset medical history, patients with mRS>2 cannot be included in the study].<br>5. Contraindication to clopidogrel, ticagrelor or aspirin:<br>• Known allergy<br>• Severe renal (creatinine exceeding 1.5 times of the upper limit of normal range) or hepatic (ALT or AST > twice the upper limit of normal range) insufficiency<br>• Severe cardiac failure (NYHA level: III to IV)<br>• History of hemostatic disorder or systemic bleeding<br>• History of thrombocytopenia or neutropenia

6.Clear indication for anticoagulation (presumed cardiac source of embolus, e.g., atrial fibrillation, prosthetic cardiac valves known or suspected endocarditis)<br>7.Iatrogenic causes (angioplasty or surgery) of minor stroke or TIA.<br>8.Anticipated requirement for long-term (>7 days) non-steroidal anti-inflammatory drugs (NSAIDs).<br>9. History of intracranial hemorrhage or amyloid angiopathy.<br>10. History of aneurysm (including intracranial aneurysm and peripheral aneurysm).<br>11. Diagnosis or suspicious diagnosis of acute coronary syndrome<br>12. History of asthma or COPD (chronic obstructive pulmonary disease).<br>13. High-risk for bradyarrhythmia (first-degree or second-degree AV block caused by sinus node disease, and brady-arrhythmic syncope without pacemaker).<br>14. History of hyperuricemia nephropathy （in the case of a purine diet, the serum uric acid >420umol/L (7.0mg/dl) for men and menopausal women on two different days on a non-same day , >357umol/L (6.0mg/dl)) for premenopausal women）	• History of drug-induced hematologic disorder or hepatic dysfunction<br>• Low white blood cell (<2×10^9/L) or platelet count (<100×10^9/L)<br>6.Hematocrit (HCT) <30%<br>7.Clear indication for anticoagulation (presumed cardiac source of embolus, e.g., atrial fibrillation, prosthetic cardiac valves known or suspected endocarditis)<br>8. History of intracranial hemorrhage or amyloid angiopathy.<br>9. History of aneurysm (including intracranial aneurysm and peripheral aneurysm).<br>10. History of asthma or COPD (chronic obstructive pulmonary disease).<br>11. High-risk for bradyarrhythmia (first-degree or second-degree AV block caused by sinus node disease, and brady-arrhythmic syncope without pacemaker).<br>12. History of hyperuricemia nephropathy （in the case of a purine diet, the serum uric acid >420umol/L (7.0mg/dl) for men and menopausal women on two different days on a non-same day , >357umol/L (6.0mg/dl)) for premenopausal women）<br>13.Anticipated requirement for long-term (>7 days) non-steroidal anti-

15.Severe non-cardiovascular comorbidity with life expectancy < 3 months.<br>16.Inability to understand and/or follow research procedures due to mental, cognitive, or emotional disorders.<br>17.Clopidogrel or ticagrelor has been used continuously for ≥5 days before enrollment.<br>18.Used heparin or oral anticoagulant drugs within 10 days before enrollment.<br>19.Undergone intravenous or arterial thrombolysis and mechanical thrombectomy within 24 hours before enrollment.<br>20. History of gastrointestinal bleeding within 3 months before enrollment or major surgery within 30 days.<br>21. Planned or likely revascularization (any angioplasty or vascular surgery) within the next 3 months.<br>22. Scheduled for surgery or interventional treatment requiring study drug cessation.<br>23. Women of childbearing age who have not taken effective contraceptive measures and have a positive pregnancy test record, as well as women who are pregnant or breastfeeding.	inflammatory drugs (NSAIDs).<br>14.Planned or likely revascularization (any angioplasty or vascular surgery) within the next 3 months。<br>15.Scheduled for surgery or interventional treatment requiring study drug cessation。<br>16.Severe non-cardiovascular comorbidity with life expectancy < 3 months.<br>17.Inability to understand and/or follow research procedures due to mental, cognitive, or emotional disorders.<br>18.Clopidogrel or ticagrelor has been used in 72 hours before randomization。.<br>19.Used heparin or oral anticoagulant drugs within 10 days before enrollment.<br>20.Undergone intravenous or arterial thrombolysis and mechanical thrombectomy within 24 hours before enrollment.<br>21. History of gastrointestinal bleeding within 3 months before enrollment or major surgery within 30 days.<br>22.Diagnosis or suspicious diagnosis of acute coronary syndrome.<br>23.Participation in another clinical study with an experimental product during the last 30 days.

24. Currently receiving an experimental drug or device.<br>25. Participation in another clinical study with an experimental product during the last 30 days.	24. Currently receiving an experimental drug or device.<br>25.Women of childbearing age who have not taken effective contraceptive measures and have a negative pregnancy test record, as well as women who are pregnant or breastfeeding.
Platelet reactivity at different visits during 90 days. (a) P2Y12 reaction units; (b) proportion of patients with high platelet reactivity.	Platelet reactivity in trial groups at baseline and follow-up, showing P2Y12 reaction units (PRU; mean (standard deviation)) and proportion (%; 95% confidence intervals) of patients with high platelet reactivity (HOPR; PRU >208 as measured by the VerifyNow P2Y12 assay). A total of 333, 306, and 280 patients in the ticagrelor/aspirin group and 336, 321, and 290 patients in the clopidogrel/aspirin group were included in the 0, 7, and 90 day analyses, respectively
Effect of ticagrelor/aspirin as compared with clopidogrel/aspirin on 90-day high platelet reactivity and clinical outcome stratified by metabolizer status.	v Effect of ticagrelor/aspirin versus clopidogrel/aspirin on high platelet reactivity and clinical outcome in PRINCE trial participants at 90 days, stratified by metaboliser status. A total of 321 patients in the ticagrelor/aspirin group and 329 patients in the clopidogrel/aspirin group were included in the genetic analysis. Patients with two *2 or *3 alleles (*2/*2, *2/*3, or *3/*3) were classified as having a poor metaboliser phenotype, those with one *2 or *3 allele (*1/*2 or

	*1/*3) were classified as having an intermediate metaboliser phenotype, those without a *2, *3, or *17 allele (*1/*1) were classified as having an extensive metaboliser phenotype, and those with a single *17 allele (*1/*17) and *17 homozygotes were classified as having an ultra-metaboliser phenotype. HOPR=P2Y12 reaction units of more than 208, as measured the VerifyNow P2Y12 assay; composite event=a new clinical vascular event, including stroke, transient ischaemic attack, myocardial infarction, or death from cardiovascular causes; NA=not applicable

APPENDIX III:

Protocol changes version 1.2 (July 17, 2019) to 1.3 (Sep 10, 2019).

**Protocol Version 1.2**	**Protocol Version 1.3**
Study visits will be performed on the day of randomization, at 14 days/discharge, at day21±2, and at day 90±7 via face-to-face interview. Study visit at month 12 (±15 day) will be performed via telephone interview for clinical events.	Study visits will be performed on the day of randomization, at day21±2, and at day 90±7 via face-to-face interview. Study visit at month 12 (±15 day) will be performed via telephone interview for clinical events.

APPENDIX IV:

Protocol changes version 1.3 (Sep 10, 2019) to 1.4 (Dec 09, 2020).

Protocol Version 1.3	Protocol Version 1.4
**Secondary Outcome Measures:**<br>• Any new stroke events (ischemic stroke or hemorrhagic stroke) within 1 year；<br>• New clinical vascular events (ischemic stroke/ hemorrhagic stroke/ TIA/ myocardial infarction/vascular death) within 3-month and within1-year；<br>• New ischemic stroke events within 3-month and 1 year；<br>• Modified Rankin Scale score changes (continuous) and dichotomized at percentage with score 0-2 vs. 3-6 at 3-month and at 1-year；<br>• Neurological impairment (National Institutes of Health Stroke Scale, NIHSS change at 3-month compared to baseline) at 3-month；<br>• Quality of Life (EuroQol EQ-5D scale) at 3-month and at 1-year.	**Secondary Outcome Measures:**<br>• Any new stroke events (ischemic stroke or hemorrhagic stroke) within 30 days and 1 year;<br>• New clinical vascular events (ischemic stroke/ hemorrhagic stroke/ TIA/ myocardial infarction/vascular death) within 3 months and 1 year；<br>• New ischemic stroke within 3 months and 1 year；<br>• Disabling stroke (Modified Rankin Scale score, mRS>1) at 3 months and 1 year；<br>• Incidence and severity of recurrent stroke and TIA during follow-up to 3 months and 1-year (Severity is measured using a six-level ordered categorical scale that incorporates the mRS: fatal stroke/severe non-fatal stroke [mRS 4 or 5]/moderate stroke [mRS 2 or 3]/mild stroke [mRS 0 or 1]/TIA/no stroke-TIA);

	• Neurological impairment at 3 months (NIHSS increased≥4 from baseline); • Quality of Life (EuroQol EQ-5D scale) at 3 months and at 1 year.
Subgroup analyses:<br>The primary efficacy endpoint will also be analyzed stratified by gender (men vs. women), age (<65 vs. ≥65 years), by etiology subtype based on the TOAST (Trial of ORG 10172 in Acute Stroke Treatment) subtype system, by diabetes (yes vs. no) and by type of LOF allele (intermediate metabolizers vs. poor metabolizers), index event (TIA or MIS), and location of symptomatic artery stenosis (intracranial/extracranial).	Subgroup analyses:<br>The primary efficacy endpoint will also be analyzed stratified by age (<65 vs. ≥ 65 years), gender (men vs. women), Body Mass Index (BMI), index event type (TIA vs. minor stroke), time from index event to randomization, etiology subtype, diabetes mellitus, hypertension, type of LOF allele, previous ischemic stroke or TIA, prior antiplatelet therapy, prior statin therapy, and prior smoking status. Symptomatic intracranial and extracranial artery stenosis will also be evaluated in subgroup

Clopidogrel with Aspirin in High-risk patients with Acute Non-disabling Cerebrovascular Events II (CHANCE-2)

Statistical Analysis Plan

Principal Investigator

Yongjun Wang, MD

Beijing Tiantan Hospital, Capital Medical University, Beijing, China

Prepared by

Yuesong Pan, PhD

Aoming Jin, PhD

Hao Li, PhD

Hongyi Yan, MD

Mengxing Wang, MD

Beijing Tiantan Hospital, Capital Medical University, Beijing, China

Version 1.0

January 9, 2019

Table of Contents

1. Introduction 188
2. Study Objective 188
3. Study Endpoint(s) 189
Primary Efficacy Endpoints: 189
Secondary Efficacy Endpoint: 189
Primary Safety Endpoint 190
Secondary Safety Endpoint 190
4. Statistical Hypotheses 190
5. Design 191
6. Sample size estimates 193
7. Analysis populations 194
Full Analysis Set (FAS)： 194
Per Protocol Set (PPS) 194
Safety Set (SS) 195
8. Treatment comparisons 195
9. General considerations for data analyses 195
Multicenter Studies 195
Examination of Subgroups 196
Multiple Comparisons and Multiplicity 196
10. Data handling conventions 196
Premature Withdrawal and Missing Data 196
Event Rates 197
Time to Event Analysis 197
11. Study Population 197
Disposition of Subjects 197
Protocol Deviations 198
Demographic and Baseline Characteristics 198
12. Efficacy Analyses 199
Primary Efficacy Analysis 199
Secondary Efficacy Analyses 200
13. Safety Analyses 202
14. References 205

1. Introduction

This statistical analysis plan (SAP) documents the planned statistical analyses for the CHANCE-2 study and is based on the protocol, together with any subsequent amendments.
This SAP is intended for the use of project team members and should be read in conjunction with the aforementioned protocol.

2. Study Objective

The primary objective of the study is to assess the effects of ticagrelor plus aspirin versus clopidogrel plus aspirin on reducing the 3-month risk of any stroke (ischemic or hemorrhagic, primary outcome) when initiated within 24 hours of symptom onset in *CYP2Y19* LOF alleles carriers with TIA or minor ischemic stroke.

The secondary objectives of the study are:

1) To assess the different rate of composite vascular outcome: composite of any stroke, TIA, myocardial infarction, and vascular death at 3-month and at one-year;

2) To assess separately the effects of ticagrelor plus aspirin regimen versus clopidogrel plus aspirin regimen on the occurrence of: ischemic stroke, hemorrhagic stroke, TIA, MI, vascular death, death of all-cause, disability (mRS: 2-6) at 3-month and at one-year;

3) To compare the safety of the two treatment regimens in terms of:

- Severe or moderate bleeding (GUSTO definition)
- Bleeding events
- Total mortality
- Intracranial hemorrhage (symptomatic and asymptomatic)
- Adverse events/ Severe adverse events

4) Subgroup analyses: Efficacy endpoint will also be analyzed stratified by gender (male vs. female), age (<65 vs. ≥65 years), by etiology subtype (main subgroups type of ischemic stroke), by diabetes (yes vs. no) and by type of LOF allele (intermediate metabolizers vs. weak metabolizers), target event (TIA or mild stroke), and responsible blood vessels (intracranial/extracranial arterial stenosis).

5) To evaluate neurological impairment (change in NIHSS scores), and Quality of Life (EuroQol EQ-5D scale) among survivors.

6) To evaluate the health economics indicators of the two treatment regimens.

7) Consistency analysis and performance evaluation of different rapid genotyping instruments.

3. Study Endpoint(s)

Primary Efficacy Endpoints:

Any new stroke events (ischemic stroke or hemorrhagic stroke) within 3 months.

Secondary Efficacy Endpoint:

1) New clinical vascular events (any stroke/ TIA/ myocardial infarction/ vascular death) within 3 months； At the same time, each new vascular event were evaluated independently;

2) New ischemic stroke within 3 months;

3) The percentage change of the Modified Rankin Scale (mRS) score between 0-2 and 3-6 points group follow-up to 3 months;

4) Continuous changes in NIHSS scores at 3 months;

5) Changes in EQ-5D-5L scale at 3 months;

6) Any new stroke events (ischemic stroke or hemorrhagic stroke) within 1 year；

7) New clinical vascular events (any stroke/ TIA/ myocardial infarction/ vascular death); At the same time, each new vascular event were evaluated independently;

8) New ischemic stroke within 1 year;

9) The percentage change of the mRS score between 0-2 points group and 3-6 points group follow-up to 1-year;

10) Continuous changes in NIHSS scores at 1 year;

11) Changes in EQ-5D-5L scale at 1 year.

Primary Safety Endpoint

Incidence of severe bleedings or moderate bleedings (GUSTO definition) at 3 months.

Secondary Safety Endpoint

1) Severe or moderate bleedings (GUSTO definition) at 1 year;

2) Bleeding events (severe or moderate bleedings or intracranial hemorrhage) at 3 months and 1 year;

3) Mortality at 3 months and 1 year;

4) Incidence of symptomatic and asymptomatic intracranial hemorrhagic events at 3 months and 1 year;

5) AEs/SAEs reported by the investigators.

4. Statistical Hypotheses

The primary endpoint for this study is new stroke event (ischemic stroke or hemorrhagic stroke) during the 3-month treatment period. The null hypothesis of no difference in this rate between the two treatment groups will be tested using a two-sided test at the 5% level of significance.

H_0: $\lambda_1/\lambda_2=1$

H_1: $\lambda_1/\lambda_2\neq1$

Where λ_1 is the rate of new stroke over the 3-month treatment period in the group treated with ticagrelor plus aspirin regimen and λ_2 is the same endpoint in the group treated with clopidogrel plus aspirin regimen.

5. Design

The CHANCE-2 study is a multicenter, prospective, double-blind, placebo-controlled, randomized clinical trial. Patients with acute nondisabling cerebrovascular events, including acute minor stroke and transient ischemic attack (TIA), are at high risk of recurrent stroke and cardiovascular events. The objective of the CHANCE-2 study is to assess the effects of ticagrelor plus aspirin versus clopidogrel plus aspirin on reducing the 3-month risk of any stroke (both ischemic and hemorrhagic, primary outcome) when initiated within 24 hours of symptom onset in *CYP2C19* LOF alleles carriers with TIA or minor ischemic stroke (MIS). Eligibility criteria for the trial participants include 40 years of age or older than 40 years and less than 80 years, *CYP2C19* LOF alleles carriers with high-risk TIAs, defined as an ABCD2 score ≥4, or minor ischemic stroke, with a National Institutes of Health Stroke Scale (NIHSS) score ≤3, and can be treated with study drug within 24 h after symptoms onset. Patients with diagnosis of hemorrhage or other pathology, contraindication to ticagrelor, clopidogrel or ASA, clear indication for anticoagulation, or intravenous thrombolytic therapy (such as intravenous rtPA) or mechanical thrombectomy will be excluded. In this study a novel point-of-care genetic test platform was used to identify carriers of the *CYP2C19* LOF alleles including poor metabolizers with at least two *2 or *3 alleles (*2/*2, *2/*3, or *3/*3) or intermediate metabolizers with one *2 or *3 allele (*1/*2 or *1/*3). All eligible patients will be randomized to receive either the ticagrelor plus aspirin group or the clopidogrel plus aspirin group. Patients in the ticagrelor plus aspirin group will receive ticagrelor of loading dosing of 180mg followed by 90mg bid for 3 months plus aspirin of loading dose of 75-300mg followed by 75mg daily for 21 days. Patients in clopidogrel plus aspirin group will receive clopidogrel of loading dosing of 300mg followed by 75mg daily for 3 months plus aspirin loading dose of 75-300mg followed by 75mg daily for 21 days. The study drug should be initiated as soon as possible within 24 hours of symptoms onset. The primary efficacy outcome is any new stroke (ischemic or hemorrhage) within 3 months. Secondary outcomes include following event within a 3 months and 1 year timeframe: (1) Composite major cardiovascular events including stroke, TIA, myocardial infarction, and cardiovascular deaths; (2) Ischemic stroke; (3) The percentage change of the mRS

score between 0-2 points group and 3-6 points group; (4) Continuous changes in NIHSS scores; (5) Changes in Quality of Life (EuroQol EQ-5D) scale. The primary safety outcome was a severe or moderate bleeding event at 3 months, according to the Global Utilization of Streptokinase and Tissue Plasminogen Activator for Occluded Coronary Arteries (GUSTO) definition. The trial was approved by the ethics committee of the participating hospitals. The study was registered with ClinicalTrials.gov (NCT04078737).

Planned Analyses

The analyses that are detailed in this SAP will be performed only when the database has been locked, all protocol violators identified, and treatment allocations have been unblinded. Membership of the Full Analysis and Per Protocol populations will be determined using the rules set out in this SAP. At a date to be agreed within the project team, a data look will be performed. This will involve production of all data displays on a subset of the data using dummy treatment codes. These are produced purely as an aide to the pre-programming of the study and no unblinding will occur.

Interim Analyses

Data and Safety Monitoring Board (DSMB) is in place to ensure the safety of subjects in the study. An independent statistician will prepare summary statistics on enrollment, subject status in the study, baseline characteristics, and safety data, including summary tables of coded SAEs and these will be examined by the DSMB. These tables will be provided to the DSMB at regular intervals. If the tables give rise to safety concerns for any treatment, the DSMB may recommend that the trial should be modified or stopped prematurely. The Steering Committee will, in conjunction with the sponsor, decide whether to act on this recommendation.

The interim analysis is based on the stopping boundaries calculated by the Lan-DeMets method with an O'Brien-Fleming type alpha spending function. One interim and one final analysis are planned to be conducted. We planned one interim analysis when 60% of total patients had undergone randomization and completed follow-up. Let p0 and p1 be the proportion of primary outcome (new stroke) for the control and intervention group respectively. Let d =p0-p1 and d^ be the observed difference. The following table lists the stopping boundaries for the interim analysis.

Table. Interim analysis for testing H_0: d=0 vs Ha: d≠0

Stage	Expected sample size*	Boundary value	Rule	Type I error achieved
1	3838	0.035	If |d^|>0.035, reject H_0 and stop; otherwise continue to the next stage	0.008
2	6396	0.023	If |d^|>0.023, reject H_0 and stop; otherwise stop, accept H_0	0.048

Note: * indicates the sample size is adjusted for 5% of loss of follow-up over 3 months

6. Sample size estimates

The study hypothesized that there is no difference in 90-day risk of stroke (ischemic or hemorrhagic) in those treated with a 3-month regimen of ticagrelor initiated with a loading dose of 180 mg followed by 90 mg bid compared with a 3-month regimen of clopidogrel initiated with a loading dose of 300 mg followed by 75 mg/d when therapy is initiated within 24 hours of symptom onset in patients with TIA or minor ischemic stroke carried *CYP2C19* LOF allele treated with aspirin 75 mg/d.

The minimum necessary sample size in the trial is established by the requirement to detect the smallest expected, clinically meaningful treatment difference comparing the treatment with placebo. Based on the genetic sub-analysis of the CHANCE study, we presume that the 90-day risk of stroke recurrence in *CYP2C19* loss-of-function allele carriers is about 9.4%, and 6.7% for noncarriers. With the point-of-care identification of the *CYP2C19* loss-of-function allele carriers to assess a proper pharmacogenetic approach for patients with high-risk TIA or MIS, we assumed a 25% relative risk reduction with alteration from clopidogrel to ticagrelor (90-day risk of stroke recurrence: 7.1%). Considering the potential impact of the interim analyses on the probability of type I error, we adjusted the statistical significance to a 2-sided α of 0.048. With a sample size of 6,396 patients,

we will have 90% power to detect a relative risk reduction (Ticagrelor: loading dose: 180mg, 90mg bid on day 2-90 vs. Clopidogrel: loading dose: 300mg, 75mg qd on day 2-90) of 25% and 5% dropouts (medication nonadherence). Assuming 58.8% prevalence of CYP2C19 loss-of-function allele carriers in a Chinese population, we projected screening 10,878 patients would be necessary.

7. Analysis populations

Full Analysis Set (FAS):

According to the basic principle of intention-to-treat (ITT), all patients who have been enrolled, randomized and had the record of at least one-day treatment of study drugs will be included. Subjects missing outcome data will be censored at the last follow-up assessment time (end of study or last visit preceding loss to follow up). This population will be the primary population for analyses of efficacy.

Per Protocol Set (PPS)

Per Protocol Set (PPS) is a subset of FAS. All patients with finishing the treatment or without violating the trail program seriously are included in PPS. The exact definition of a serious violation will be finalized at the time of data review and may generally include (but is not limited to) the following criteria: non-compliance with the primary inclusion criteria, concomitant interference treatment after enrollment, poor compliance, and exceed the time window of follow-up seriously and so on. A partial protocol violator will be included in the Per Protocol Population up to the time of their violation. For the Per Protocol Population, participants will be analyzed according to the treatment received, providing the same treatment was taken for the duration of the study. If study medication was changed then the participant will be considered a partial protocol violator (from the point of change onwards).

Safety Set (SS)

All patients who received at least 1-time of study drug according to the study protocol and safety assessment available will be included in the safety population. This population will be used for safety analyses.

8. Treatment comparisons

The treatment comparison of interest in this study is to assess the antiplatelet effects of ticagrelor plus aspirin versus clopidogrel plus aspirin in Chinese patients in *CYP2C19* LOF alleles carriers with TIA or minor stroke.

9. General considerations for data analyses

All programming will be performed using SAS Version 9.4. All analysis output will use the following treatment group naming conventions and treatment order:

Ticagrelor plus aspirin group: Ticagrelor of loading dosing of 180mg followed by 90mg bid for 3 months plus aspirin of loading dose of 75-300mg followed by 75mg daily for 21 days.

Clopidogrel plus aspirin group: Clopidogrel of loading dosing of 300mg followed by 75mg daily for 3 months plus aspirin loading dose of 75-300mg followed by 75mg daily for 21 days.

All statistics were two sided with a $P<0.05$ considered significant.

Multicenter Studies

As stated in the protocol, centers with less than 20 subjects will be pooled with larger centers within the same geographic region so that centers are of a reasonable size for the purpose of the statistical analyses. This process will be performed and finalized before the treatment codes are unblinded.

In multicenter randomized controlled clinical study, there were some difference effect in different center due to different baseline, clinical practice or other factor, therefore, central effect analysis was required. Stratified analysis was used to exclude the mixed effect of results caused by

center effect: each center was served as a stratum, calculating the HR by Cox proportional hazards model.

Examination of Subgroups

The rate of new stroke (ischemic or hemorrhagic) at 90 days will be presented for each level of the covariates listed below. The extent to which the treatment effect varies across levels of each subgroup will be assessed through interaction tests.

Multiple Comparisons and Multiplicity

A single primary efficacy variable has been defined for this study, with all other efficacy variables identified as secondary or other. Similarly, only one treatment comparison is of interest in the study and therefore there are no requirements to adjust for multiple comparisons or multiple endpoints within this study.

10. Data handling conventions

Premature Withdrawal and Missing Data

If any subject withdraws prematurely from the study (prior to the final visit D90±7 days assessment), they are required to complete the withdrawal visit in the CRF. The reasons for withdrawal will be presented in a summary table. For the purposes of summaries and analysis of clinic visit data, this visit will be assigned to the next scheduled clinic visit for that subject, regardless of whether the date falls within the next visit window.

Subjects who withdraw before the end of the study, but who do provide at least one post-baseline measure for a particular endpoint, will be included in the analysis.

Subjects who do not attend any visits after randomization will be excluded from analysis of any endpoint, as no post−baseline data will be available.

Event Rates

The number of people and person-time of events should be recorded in detail and showing the event rate in 90 days of each treatment group in summary statement.

The event rate for each treatment group will be calculated as: the sum of number of event for all the patients / the sum of number of treatment periods for all the patients.

Time to Event Analysis

Differences between treatments in the risk of new stroke (ischemic or hemorrhagic) event and combined vascular events during maximum 90–day follow–up were assessed using standard Kaplan–Meier time–to–event approaches. The time to the first event was used in the model when there were multiple events of the same type. Patients were considered censored at the time of study termination or death if there were no events occurred during the study.

11.Study Population

Disposition of Subjects

The number of subjects in each analysis population will be presented, subjects to be excluded from the Per Protocol population will be listed, and the total number of subjects attending each clinic visit will also be summarized by treatment group.

The number of subjects randomized, completed and prematurely withdrawn from the study will be presented for each treatment group. The primary reasons for withdrawal both prior to and post randomization will also be presented.

A data display listing and summary of deviations from the inclusion/exclusion criteria will be presented for all subjects who were either entered or randomized into the trial.

Protocol Deviations

Subject data will be examined for evidence of protocol violators in order to assess how well the protocol was followed. Inclusion and exclusion criteria are detailed in the study protocol.

Subjects who commit protocol violations will be included in the FAS Population but excluded from the Per Protocol Population. These protocol violations will be shown in a listing. Subjects can either be full or partial protocol violators. A full protocol violator is completely excluded from the Per Protocol Population. A partial protocol violator has only some data excluded. For subjects who violated the protocol during the treatment period due to unpermitted changes in the medication or prohibited concurrent medication, the analysis will only use data recorded prior to the violation. For all violations which reference the treatment period, the treatment start date will be used as the reference date.

A listing of all possible protocol violators will be produced for clinical review. The final list of subjects who are protocol violators and are therefore excluded from the Per−Protocol population will be agreed by the study team.

Demographic and Baseline Characteristics

The following demographic information will be listed and summarized for subjects in each treatment group: age, sex, body mass index (BMI). This will also be done for stroke and TIA history, medical history, smoking history, alcohol use, symptoms of the index event, pretreatment Rankin Score and NIHSS score.

Vital signs including supine systolic blood pressure, diastolic blood pressure, and heart rate will also be listed and summarized in each treatment group.

The continuous data followed normal distribution will be presented as mean and standard deviation, and the continuous data followed skewness distribution will be presented as median and

interquartile range; categorical data will be presented as n(%). T-test or Wilcoxon rank sum test will be used for comparison between two continuous data, and Chi-squared tests, Fisher exact test, or Wilcoxon rank sum test will be used for comparison between two categorical data.

12. Efficacy Analyses

Primary Efficacy Analysis

The primary endpoint is the rate of new stroke (both ischemic and hemorrhagic) reported during the 3 months treatment period. FAS will be the primary population for efficacy analyses. PPS will be used as secondary population for the efficacy analyses. If the results in the PPS population are inconsistent with the FAS population, detailed analysis of the inconsistent results is required.

Main Model

The time to first new stroke (both ischemic and hemorrhagic) reported during the 3 months treatment period for the FAS Population will be summarized by treatment group using Kaplan-Meier estimates. The hazard ratio for the treatment comparison will be derived using a Cox proportional hazards model, including the pooled study center as a random effect. The hazards ratios with 95% CI will be reported. This will also be presented graphically on a Kaplan-Meier curve. The log-rank test will be used to evaluate the statistical significance of the treatment effect.

Interactions with Subgroups

Summary tables will be produced for the predefined subgroups and interactions between treatment and these subgroups will be investigated, using a Cox proportional hazards model. A separate model will be used for each interaction to determine its significance. This will also be presented graphically on a forest plot.

The predefined subgroups including:

- Gender (male vs. female)
- Different age category (<65 years vs. ≥65 years)
- Etiological stroke subtype (main subgroups type of ischemic stroke)
- History of diabetes (yes vs. no)
- CYP2C19 genetic variants (intermediate metabolizers vs. weak metabolizers)
- Target event (TIA vs. mild stroke)
- Responsible blood vessels (Intracranial artery stenosis vs. extracranial arterial stenosis)

Secondary Efficacy Analyses

Rate of new clinical vascular events (any stroke, TIA, myocardial infarction, or vascular death) and the individually event within 3 months

The rate of new clinical vascular events occurring within 3 months will be analyzed using a Cox proportional hazard model similar to that in the previous section, with the pooled study center as a random effect. A combined vascular event is defined as any of the 4 following events: stroke, TIA, myocardial infarction, or vascular death. This will also be presented graphically on a Kaplan-Meier curve. Each of the 4 vascular events will also be analyzed using the same method and the event rate for each event will be calculated for each treatment group. The hazard ratios with 95% CIs will be reported. The log-rank test will be used to evaluate the statistical significance of the treatment effect.

Rate of new ischemic stroke within 3 months

The rate of ischemic stroke occurring within 3 months will be analyzed using a Cox proportional hazard model, with the pooled study center as a random effect. This will also be presented graphically on a Kaplan-Meier curve. The hazard ratio with 95% CI will be reported. The log-rank test will be used to evaluate the statistical significance of the treatment effect.

Modified Rankin Scale score dichotomized at percentage with score 0-2 vs 3-6 at 3 months follow up

Logistic regression will be performed to compare proportion of Modified Rankin Scale score 0-2 vs 3-6 at 3 months follow-up between the two treatment groups. The odds ratio with 95% CI will be reported.

Neurological impairment at 3 months follow-up

Changes of NIHSS scores between the end of study and baseline will be summarized for the two treatment groups among the survivors during the study. The treatment difference will be tested using student t−test or Wilcoxon rank sum test as appropriate.

Quality of life at 3 months follow-up

Health related quality of life will be measured using EuroQol EQ−5D scale among the survivors. Treatment differences will be tested using student t−test or Wilcoxon rank sum test as appropriate.

Rate of any new strokes (ischemic stroke or hemorrhagic stroke) within 1 year

The rate of new stroke (both ischemic and hemorrhagic) reported within 1 year will be analyzed using a Cox proportional hazard model, with the pooled study center as a random effect. This will also be presented graphically on a Kaplan-Meier curve. The hazard ratio with 95% CI will be reported. The log-rank test will be used to evaluate the statistical significance of the treatment effect.

Rate of new clinical vascular events (any stroke, TIA, myocardial infarction, or vascular death) and the individually event within 1 year

The rate of new clinical vascular events occurring within 1 year will be analyzed using a Cox proportional hazard model, with the pooled study center as a random effect. A combined vascular event is defined as any of the 4 following events: stroke, TIA, myocardial infarction, or vascular death. This will also be presented graphically on a Kaplan-Meier curve. Each of the 4 vascular events will also be analyzed using the same method and the event rate for each event will be calculated for each treatment group.

The hazard ratios with 95% CIs will be reported. The log-rank test will be used to evaluate the statistical significance of the treatment effect.

Rate of new ischemic stroke within 1 year

The rate of ischemic stroke occurring within 1 year will be analyzed using a Cox proportional hazard model, with the pooled study center as a random effect. This will also be presented graphically on a Kaplan-Meier curve. The hazard ratio with 95% CI will be reported. The log-rank test will be used to evaluate the statistical significance of the treatment effect.

Modified Rankin Scale score dichotomized at percentage with score 0-2 vs 3-6 at 1-year follow up

Logistic regression will be performed to compare proportion of Modified Rankin Scale score 0-2 vs 3-6 at 1-year follow-up between the two treatment groups. The odds ratio with 95% CI will be reported.

Neurological impairment at 1-year follow-up

Changes of NIHSS scores between the end of study and baseline will be summarized for the two treatment groups among the survivors during the study. The treatment difference will be tested using student t−test or Wilcoxon rank sum test as appropriate.

Quality of life at 1-year follow-up

Health related quality of life will be measured using EuroQol EQ−5D scale among the survivors. Treatment differences will be tested using student t−test or Wilcoxon rank sum test as appropriate.

13. Safety Analyses

All analyses of safety data will be carried out using the safety set (SS) population.

Extent of Exposure

The extent of exposure will be calculated as the number of days between start of treatment and end of treatment (i.e. treatment stop date – treatment start date + 1), and

categorized into months: <1, 1−2, 2−3 months. Exposure will then be summarized by treatment group.

Bleeding Events

Severe or moderate bleeding (GUSTO definition) within 3 months;

Severe or moderate bleeding (GUSTO definition) at 1 year;

Any bleeding events (severe or moderate hemorrhage, intracranial hemorrhage) within 3 months and 1 year;

Symptomatic and asymptomatic intracranial hemorrhagic events within 3 months and 1 year;

For most bleeding events, the Cox proportional hazards model will be used to compare the hazard ratio (HR) between the two treatments, or Poisson regression or negative binomial regression which are more appropriate for the analysis of rare event.

Adverse Events

Adverse events (AEs) will be coded using the MedDRA coding dictionary (Version 6.0 or a later release) and grouped by system organ class (as detailed in the study protocol). Separate data display listings and summaries will be presented for adverse events that start prior to first dose of study medication (pre−treatment), whilst on study medication (during treatment) and after the last dose of study medication (post−treatment).

Within each treatment group, the number and percentage of subjects experiencing an AE will be summarized by system organ class and preferred term and Fisher's Exact test will be used to compare the number of each grouped AE event between treatment groups. In addition, a separate summary will be provided for AEs experienced by more than 5% of subjects in either of the treatment groups.

Deaths and Serious Adverse Events

Summary tables and data displays will be provided for serious adverse events (as detailed in the study protocol). In addition, all deaths and serious AE's will be documented in a case narrative format in the clinical study report.

The number of deaths occurring over the treatment period will be summarized and Fisher's Exact test will be used to compare the number of deaths between treatment groups.

14. References

1. Nagashima Z, Tsukahara K, Morita S, et al. Platelet reactivity in the early and late phases of acute coronary syndromes according to cytochrome p450 2c19 phenotypes. J Cardiol 2013; 62: 158–164.
2. Wang Y, Wang Y, Zhao X, et al. Clopidogrel with aspirin in acute minor stroke or transient ischemic attack. N Engl J Med 2013;369: 11-19.
3. Rabin R, Charro Fd. EQ-SD: a measure of health status from the EuroQol Group. Annals of Medicine 2001;33: 337-343.
4. Andersen P, Gill, RD. Cox's regression model for counting processes: a large sample study. Annals of Statistics 1982;10: 1100-1120.

Clopidogrel with Aspirin in High-risk patients with Acute Non-disabling Cerebrovascular Events II (CHANCE-2)

Statistical Analysis Plan

Principal Investigator

Yongjun Wang, MD

Beijing Tiantan Hospital, Capital Medical University, Beijing, China

Prepared by

Yuesong Pan, PhD

Aoming Jin, PhD

Hao Li, PhD

Hongyi Yan, MD

Mengxing Wang, MD

Beijing Tiantan Hospital, Capital Medical University, Beijing, China

Version 1.3

April 20, 2021

Table of Contents

1. Introduction....208
2. Study Objective....208
3. Study Endpoint(s)....209
Primary Efficacy Endpoints:....209
Secondary Efficacy Endpoint:....209
Primary Safety Endpoint....210
Secondary Safety Endpoint....210
4. Statistical Hypotheses....210
5. Design....211
6. Sample size estimates....213
7. Analysis populations....215
Full Analysis Set (FAS)：....215
Per Protocol Set (PPS)....215
Safety Set (SS)....215
8. Treatment comparisons....216
9. General considerations for data analyses....216
Multicenter Studies....216
Examination of Subgroups....217
Multiple Comparisons and Multiplicity....217
10. Data handling conventions....217
Premature Withdrawal and Missing Data....217
Event Rates....218
Time to Event Analysis....218
11. Study Population....218
Disposition of Subjects....218
Protocol Deviations....219
Demographic and Baseline Characteristics....219
12. Efficacy Analyses....220
Primary Efficacy Analysis....220
Secondary Efficacy Analyses....221
13. Safety Analyses....224
14. References....226

1. Introduction

This statistical analysis plan (SAP) documents the planned statistical analyses for the CHANCE-2 study and is based on the protocol, together with any subsequent amendments.

This SAP is intended for the use of project team members and should be read in conjunction with the aforementioned protocol.

2. Study Objective

The primary objective of the study is to assess the effects of ticagrelor plus aspirin versus clopidogrel plus aspirin on reducing the 3-month risk of any new stroke (ischemic or hemorrhagic, primary outcome) when initiated within 24 hours of symptom onset in *CYP2Y19* LOF alleles carriers with TIA or minor ischemic stroke.

The secondary objectives of the study are:

1) To assess the different rate of composite vascular outcome: composite of any stroke, TIA, myocardial infarction, and vascular death at 3-month and at one-year;

2) To assess the effects of ticagrelor plus aspirin regimen versus clopidogrel plus aspirin regimen on the incidence of: stroke within 30 days, neurological impairment at 3 months (NIHSS increased ≥4 from baseline), ischemic stroke, TIA, MI, vascular death, disabling stroke (mRS: 2-6) at 3-month and at one-year;

3) To assess the incidence and severity of recurrent stroke and TIA during follow-up to 3 months and 1-year (Severity is measured using a six-level ordered categorical scale that incorporates the mRS: fatal stroke/severe non-fatal stroke [mRS 4 or 5]/moderate stroke [mRS 2 or 3]/mild stroke [mRS 0 or 1]/TIA/no stroke-TIA);

4) To compare the safety of the two treatment regimens in terms of:

- Severe or moderate bleeding (GUSTO definition)
- Bleeding events
- Total mortality
- Adverse events/ Severe adverse events

5) To evaluate Quality of Life (EuroQol EQ-5D scale) among survivors;

6) Subgroup analyses: The primary efficacy endpoint will also be analyzed stratified by age (<65 vs. ≥65 years), gender (men vs. women), Body Mass Index (BMI), index event (TIA vs. Minor stroke), time from index event to randomization, etiology subtype, diabetes mellitus, hypertension, type of LOF allele, previous ischemic stroke or TIA, prior antiplatelet therapy, prior statin therapy, prior smoking status, and symptomatic intracranial and extracranial artery stenosis will be evaluated in subgroup analyses.

3. Study Endpoint(s)

Primary Efficacy Endpoints:

Any new stroke events (ischemic stroke or hemorrhagic stroke) within 3 months.

Secondary Efficacy Endpoint:

1) Any new stroke events (ischemic stroke or hemorrhagic stroke) within 30 days and 1 year;

2) New clinical vascular events (ischemic stroke/ hemorrhagic stroke/ TIA/ MI/ vascular death) within the 3 months and 1 year;

3) New ischemic stroke within 3 months and 1 year;

4) Disabling stroke (Modified Rankin Scale score, mRS>1) at 3 months and 1 year;

5) Incidence and severity of recurrent stroke and TIA during follow-up to 3 months and

1-year (Severity is measured using a six-level ordered categorical scale that incorporates the mRS: fatal stroke/severe non-fatal stroke [mRS 4 or 5]/moderate stroke [mRS 2 or 3]/mild stroke [mRS 0 or 1)]/TIA/no stroke-TIA);

6) Neurological impairment at 3 months (NIHSS increased ≥4 from baseline);

7) Quality of Life (EuroQol EQ-5D scale) at 3 months and at 1 year.

Primary Safety Endpoint

Incidence of severe bleedings or moderate bleedings (GUSTO definition) at 3 months.

Secondary Safety Endpoint

1) Incidence of severe bleedings or moderate bleedings (GUSTO definition) at 1 year;

2) Bleeding events (severe or moderate bleedings or intracranial hemorrhage) at 3 months and 1 year;

3) Mortality within 3 months and 1 year;

4) AEs/SAEs reported by the investigators at 3 months and 1 year.

4. Statistical Hypotheses

The primary endpoint for this study is new stroke event (ischemic stroke or hemorrhagic stroke) within the 3-month treatment period. In this study of patients with TIA or MIS carried CYP2C19 LOF allele treated with aspirin 75 mg/d, the null hypothesis of no difference in 90-day risk of new stroke between the two treatment groups will be tested using a two-sided test at the 5% level of significance.

H_0: $\lambda_1/\lambda_2=1$

H_1: $\lambda_1/\lambda_2\neq1$

Where λ_1 is the rate of new stroke over the 3-month treatment period in the group treated with ticagrelor plus aspirin regimen and λ_2 is the same endpoint in the group

treated with clopidogrel plus aspirin regimen.

5. Design

The CHANCE-2 study is a multicenter, double-blind, placebo-controlled, randomized clinical trial. Patients with acute nondisabling cerebrovascular events, including acute minor stroke and transient ischemic attack (TIA), are at high risk of recurrent stroke and cardiovascular events. The objective of the CHANCE-2 study is to assess the effects of ticagrelor plus aspirin versus clopidogrel plus aspirin on reducing the 3-month risk of any stroke (both ischemic and hemorrhagic, primary outcome) when initiated within 24 hours of symptom onset in *CYP2C19* LOF alleles carriers with TIA or minor stroke. Eligibility criteria for the trial participants include 40 years of age or older, *CYP2C19* LOF alleles carriers with high-risk TIAs, defined as an ABCD2 score ≥4, or acute non-disabling ischemic stroke, with a National Institutes of Health Stroke Scale (NIHSS) score ≤3, and can be treated with study drug within 24 h after symptoms onset. Patients with diagnosis of hemorrhage or other pathology, contraindication to ticagrelor, clopidogrel or ASA, clear indication for anticoagulation, or intravenous thrombolytic therapy (such as intravenous rtPA) or mechanical thrombectomy will be excluded. In this study a novel point-of-care genetic test platform was used to identify carriers of the *CYP2C19* LOF alleles including poor metabolizers with at least two *2 or *3 alleles (*2/*2, *2/*3, or *3/*3) or intermediate metabolizers with one *2 or *3 allele (*1/*2 or *1/*3). All eligible patients will be randomized to receive either the ticagrelor plus aspirin group or the clopidogrel plus aspirin group. Patients in the ticagrelor plus aspirin group will receive ticagrelor of loading dosing of 180mg followed by 90mg bid for 3 months plus aspirin of loading dose of 75-300mg followed by 75mg daily for 21 days. Patients in clopidogrel plus aspirin group will receive clopidogrel of loading dosing of 300mg followed by 75mg daily for 3 months plus aspirin loading dose of 75-300mg followed by 75mg daily for 21 days. The study drug should be initiated as soon as

possible within 24 hours of symptoms onset. The primary efficacy outcome is any new stroke (ischemic or hemorrhage) within 3 months. Secondary outcomes include any new stroke within 30 days and 1 year, following event within a 3 months and 1 year timeframe: (1) new clinical vascular events including ischemic stroke, hemorrhagic stroke, TIA, myocardial infarction, and vascular deaths; (2) Ischemic stroke; (3) Disabling stroke (Modified Rankin Scale score, mRS>1); (4) Incidence and severity of recurrent stroke and TIA; (5) Quality of Life (EuroQol EQ-5D scale; and Neurological impairment at 3 months (NIHSS increased ≥4 from baseline). The primary safety outcome was incidence of severe bleedings or moderate bleedings event at 3 months, according to the Global Utilization of Streptokinase and Tissue Plasminogen Activator for Occluded Coronary Arteries (GUSTO) definition. The trial was approved by the ethics committee of the participating hospitals. The study was registered with ClinicalTrials.gov (NCT04078737).

Planned Analyses

The analyses that are detailed in this SAP will be performed only when the database has been locked, all protocol violators identified, and treatment allocations have been unblinded. Membership of the Full Analysis and Per Protocol populations will be determined using the rules set out in this SAP. At a date to be agreed within the project team, a data look will be performed. This will involve production of all data displays on a subset of the data using dummy treatment codes. These are produced purely as an aide to the pre-programming of the study and no unblinding will occur.

Interim Analyses

Data and Safety Monitoring Board (DSMB) is in place to ensure the safety of subjects in the study. An independent statistician will prepare summary statistics on enrollment, subject status in the study, baseline characteristics, and safety data, including summary tables of coded SAEs and these will be examined by the DSMB. These tables will be provided to the DSMB at regular intervals. If the tables give rise to safety concerns for

any treatment, the DSMB may recommend that the trial should be modified or stopped prematurely. The Steering Committee will, in conjunction with the sponsor, decide whether to act on this recommendation.

The interim analysis is based on the stopping boundaries calculated by the Lan-DeMets method with an O'Brien-Fleming type alpha spending function. One interim and one final analysis are planned to be conducted. We planned one interim analysis when 60% of total patients had undergone randomization and completed follow-up. Let p0 and p1 be the proportion of primary outcome (new stroke) for the control and intervention group respectively. Let d =p0-p1 and d^ be the observed difference. The following table lists the stopping boundaries for the interim analysis.

Table. Interim analysis for testing H_0: d=0 vs Ha: d≠0

Stage	Expected sample size*	Boundary value	Rule	Type I error achieved
1	3838	0.035	If |d^|>0.035, reject H_0 and stop; otherwise continue to the next stage	0.008
2	6396	0.023	If |d^|>0.023, reject H_0 and stop; otherwise stop, accept H_0	0.048

Note: * indicates the sample size is adjusted for 5% of loss of follow-up over 3 months

6. Sample size estimates

The study hypothesized that there is no difference in 90-day risk of stroke (ischemic or hemorrhagic) in those treated with a 3-month regimen of ticagrelor initiated with a loading dose of 180 mg followed by 90 mg bid compared with a 3-month regimen of clopidogrel initiated with a loading dose of 300 mg followed by 75 mg/d when therapy is initiated within 24 hours of symptom onset in patients with TIA or minor ischemic

stroke carried *CYP2C19* LOF allele treated with aspirin 75 mg/d.

The minimum necessary sample size in the trial is established by the requirement to detect the smallest expected, clinically meaningful treatment difference comparing the treatment with placebo. Based on the genetic sub-analysis of the CHANCE study, we presume that the 90-day risk of stroke recurrence in CYP2C19 loss-of-function allele carriers is about 9.4%, and 6.7% for noncarriers. With the point-of-care identification of the CYP2C19 loss-of-function allele carriers to assess a proper pharmacogenetic approach for patients with high-risk TIA or MIS, we assumed a 25% relative risk reduction with alteration from clopidogrel to ticagrelor (90-day risk of stroke recurrence: 7.1%). Considering the potential impact of the interim analyses on the probability of type I error, we adjusted the statistical significance to a 2-sided α of 0.048. With a sample size of 6,396 patients, we will have 90% power to detect a relative risk reduction (Ticagrelor: loading dose: 180mg, 90mg bid on day 2-90 vs. Clopidogrel: loading dose: 300mg, 75mg qd on day 2-90) of 25% and 5% dropouts (medication nonadherence). Assuming 58.8% prevalence of CYP2C19 loss-of-function allele carriers in a Chinese population, we projected screening 10,878 patients would be necessary. Based on the results of interim analysis, the DSMB will recommend whether the sample size need to be re-calculated.

According to the DSMB meeting on April 7, 2021, the DSMB lookup the overall event rates of the trial and suggested not to unblind the treatment assignment in the interim analysis. Therefore, comparison of efficacy and safety outcome between two treatment groups were not performed in the interim analysis, and sample size was not re-calculated. The type I error level of the statistical significance will be set to a 2-sided α of 0.05 in the final analysis.

7. Analysis populations

Full Analysis Set (FAS):

According to the basic principle of intention-to-treat (ITT), all patients who were enrolled, randomized and had the record of at least one-day treatment of study drugs will be included. Subjects missing outcome data will be censored at the last follow-up assessment time (end of study or last visit preceding loss to follow up). This population will be the primary population for analyses of efficacy.

Per Protocol Set (PPS)

Per Protocol Set (PPS) is a subset of FAS. All patients with finishing the treatment without violating the trail program seriously are included in PPS. The exact definition of a serious violation will be finalized at the time of data review and may generally include (but is not limited to) the following criteria: failure to meet the main inclusion criteria, concomitant treatment that seriously interferes with the evaluation of the efficacy of study drugs after randomization, poor compliance, and exceed the time window of follow-up seriously and so on. A partial protocol violator will be included in the Per Protocol Population up to the time of their violation. For the Per Protocol Population, participants will be analyzed according to the treatment received, providing the same treatment was taken for the duration of the study. If study medication was changed then the participant will be considered a partial protocol violator (from the point of change onwards).

Safety Set (SS)

All patients who received at least 1-time of study drug according to the study protocol and safety assessment available will be included in the safety population. Throughout

the safety results sections, erroneously treated patients (eg, those randomized to ticagrelor and aspirin group but actually given clopidogrel and aspirin) will be accounted for in the actual treatment group. This population will be used for safety analyses.

8. Treatment comparisons

The treatment comparison of interest in this study is to assess the antiplatelet effects of ticagrelor plus aspirin versus clopidogrel plus aspirin in Chinese patients in *CYP2C19* LOF alleles carriers with TIA or minor stroke.

9. General considerations for data analyses

All programming will be performed using SAS Version 9.4. All analysis output will use the following treatment group naming conventions and treatment order:

Ticagrelor plus aspirin group: Ticagrelor of loading dosing of 180mg followed by 90mg bid for 3 months plus aspirin of loading dose of 75-300mg followed by 75mg daily for 21 days.

Clopidogrel plus aspirin group: Clopidogrel of loading dosing of 300mg followed by 75mg daily for 3 months plus aspirin loading dose of 75-300mg followed by 75mg daily for 21 days.

All statistics were two sided with a $P<0.05$ considered significant.

Multicenter Studies

As stated in the protocol, centers with less than 20 subjects will be pooled with larger centers within the same geographic region so that centers are of a reasonable size for the purpose of the statistical analyses. This process will be performed and finalized before the treatment codes are unblinded.

In multicenter randomized controlled clinical study, there were some difference effect in different center due to different baseline, clinical practice or other factor, therefore, central effect analysis was required. Stratified analysis was used to exclude the mixed effect of results caused by center effect: each center was served as a stratum, calculating the HR by Cox proportional hazards model.

Examination of Subgroups

The rate of new stroke (ischemic or hemorrhagic) at 90 days will be presented for each level of the covariates listed below. The extent to which the treatment effect varies across levels of each subgroup will be assessed through interaction tests.

Multiple Comparisons and Multiplicity

A single primary efficacy variable has been defined for this study, with all other efficacy variables identified as secondary or other. Similarly, only one treatment comparison is of interest in the study and therefore there are no requirements to adjust for multiple comparisons or multiple endpoints within this study.

10. Data handling conventions

Premature Withdrawal and Missing Data

If any subject withdraws prematurely from the study (prior to the final visit D90±7 days assessment), they are required to complete the withdrawal visit in the CRF. The reasons for withdrawal will be presented in a summary table. For the purposes of summaries and analysis of clinic visit data, this visit will be assigned to the next scheduled clinic visit for that subject, regardless of whether the date falls within the next visit window. Subjects who withdraw before the end of the study, but who do provide at least one

post-baseline measure for a particular endpoint, will be included in the analysis. Subjects who do not attend any visits after randomization will be excluded from analysis of any endpoint, as no post−baseline data will be available.

Event Rates

The number of people and person-time of events should be recorded in detail and showing the event rate in 90 days of each treatment group in summary statement.
The event rate for each treatment group will be calculated as: the sum of number of event for all the patients / the sum of number of treatment periods for all the patients.

Time to Event Analysis

Differences between treatments in the risk of new stroke (ischemic or hemorrhagic) event and clinical vascular events during maximum 90−day follow−up were assessed using standard Kaplan−Meier time−to−event approaches. The time to the first event was used in the model when there were multiple events of the same type. Patients were considered censored at the time of study termination or death if there were no events occurred during the study.

11.Study Population

Disposition of Subjects

The number of subjects in each analysis population will be presented, subjects to be excluded from the Per Protocol population will be listed, and the total number of subjects attending each clinic visit will also be summarized by treatment group.

The number of subjects randomized, completed and prematurely withdrawn from the study will be presented for each treatment group. The primary reasons for

withdrawal both prior to and post randomization will also be presented.

A data display listing and summary of deviations from the inclusion/exclusion criteria will be presented for all subjects who were either entered or randomized into the trial.

Protocol Deviations

Subject data will be examined for evidence of protocol violators in order to assess how well the protocol was followed. Inclusion and exclusion criteria are detailed in the study protocol.

Subjects who commit protocol violations will be included in the FAS Population but excluded from the Per Protocol Population. These protocol violations will be shown in a listing. Subjects can either be full or partial protocol violators. A full protocol violator is completely excluded from the Per Protocol Population. A partial protocol violator has only some data excluded. For subjects who violated the protocol during the treatment period due to unpermitted changes in the medication or prohibited concurrent medication, the analysis will only use data recorded prior to the violation. For all violations which reference the treatment period, the treatment start date will be used as the reference date.

A listing of all possible protocol violators will be produced for clinical review. The final list of subjects who are protocol violators and are therefore excluded from the Per−Protocol population will be agreed by the study team.

Demographic and Baseline Characteristics

The following demographic information will be listed and summarized for subjects in each treatment group: age, sex, body mass index (BMI). This will also be done for

stroke and TIA history, medical history, smoking history, alcohol use, symptoms of the index event, pretreatment Rankin Score and NIHSS score.

Vital signs including supine systolic blood pressure, diastolic blood pressure, and heart rate will also be listed and summarized in each treatment group.

The continuous data followed normal distribution will be presented as mean and standard deviation, and the continuous data followed skewness distribution will be presented as median and interquartile range; categorical data will be presented as n(%). T-test or Wilcoxon rank sum test will be used for comparison between two continuous data, and Chi-squared tests or Fisher exact test will be used for comparison between two categorical data.

12. Efficacy Analyses

Primary Efficacy Analysis

The primary endpoint is the rate of new stroke (both ischemic and hemorrhagic) reported during the 3 months treatment period. FAS will be the primary population for efficacy analyses. PPS will be used as secondary population for the efficacy analyses. If the results in the PPS population are inconsistent with the FAS population, detailed analysis of the inconsistent results is required.

Main Model

The time to first new stroke (ischemic stroke or hemorrhagic stroke) reported during the 3 months treatment period for the FAS Population will be summarized by treatment group using Kaplan-Meier estimates. The hazard ratio for the treatment comparison will be derived using a Cox proportional hazards model, including the pooled study center as a random effect. The hazards ratios with 95% CI will be reported. This will also be presented graphically on a Kaplan-Meier curve. The log-rank test will be used

to evaluate the statistical significance of the treatment effect.

Interactions with Subgroups

Summary tables will be produced for the predefined subgroups and interactions between treatment and these subgroups will be investigated, using a Cox proportional hazards model. A separate model will be used for each interaction to determine its significance. This will also be presented graphically on a forest plot.

The predefined subgroups including:

- Age (<65 years vs. ≥65 years)
- Gender (men vs. women)
- BMI
- Index event (TIA vs. minor ischemic stroke)
- Time from index event to randomization
- Etiological stroke subtype based on the TOAST Classification
- Diabetes mellitus
- Hypertension
- Type of *CYP2Y19* LOF alleles (intermediate metabolizers vs. poor metabolizers)
- Previous ischemic stroke or TIA
- Prior antiplatelet therapy
- Prior statin therapy
- Prior smoking status
- Location of symptomatic artery stenosis (Intracranial artery stenosis vs. extracranial artery stenosis)

Secondary Efficacy Analyses

Rate of new stroke events within 30 days

The rate of stroke occurring within 30 days will be analyzed using a Cox proportional

hazard model, with the pooled study center as a random effect. This will also be presented graphically on a Kaplan-Meier curve. The hazard ratio with 95% CI will be reported. The log-rank test will be used to evaluate the statistical significance of the treatment effect.

Rate of new clinical vascular events (ischemic stroke, hemorrhagic stroke, TIA, myocardial infarction, or vascular death) within 3 months

The rate of new clinical vascular events occurring within 3 months will be analyzed using a Cox proportional hazard model similar to that in the previous section, with the pooled study center as a random effect. A combined vascular event is defined as any of the 5 following events: ischemic stroke, hemorrhagic stroke, TIA, myocardial infarction, or vascular death. This will also be presented graphically on a Kaplan-Meier curve. The hazard ratios with 95% CIs will be reported. The log-rank test will be used to evaluate the statistical significance of the treatment effect.

Rate of new ischemic stroke within 3 months

The rate of ischemic stroke occurring within 3 months will be analyzed using a Cox proportional hazard model, with the pooled study center as a random effect. This will also be presented graphically on a Kaplan-Meier curve. The hazard ratio with 95% CI will be reported. The log-rank test will be used to evaluate the statistical significance of the treatment effect.

Proportion of disabling stroke at 3 months

The proportion of disabling stroke at 3 months will be analyzed using a logistic regression model. The disabling stroke is defined as Modified Rankin Scale score (mRS) >1. The odds ratio with 95% CI will be reported.

Incidence and severity of recurrent stroke and TIA during follow-up to 3 months

Differences in proportion of severity of recurrent stroke and TIA during follow-up to 3 months between the two treatment groups will be tested by Wilcoxon rank sum test. Severity is measured using a six-level ordered categorical scale that incorporates the

mRS: fatal stroke/severe non-fatal stroke [mRS 4 or 5]/moderate stroke [mRS 2 or 3]/mild stroke [mRS 0 or 1)]/TIA/no stroke-TIA. The test statistics with p value will be reported.

Proportion of neurological impairment at 3 months

The proportion of neurological impairment will be analyzed using a logistic regression model. The neurological impairment is defined as NIHSS increased ≥4 from baseline. The odds ratio with 95% CI will be reported.

Quality of life at 3 months follow-up

Health related quality of life will be measured using EuroQol EQ−5D scale among the survivors. Treatment differences will be tested using student t−test or Wilcoxon rank sum test as appropriate.

Rate of any new strokes (ischemic stroke or hemorrhagic stroke) within 1 year

The rate of new stroke (both ischemic and hemorrhagic) reported within 1 year will be analyzed using a Cox proportional hazard model, with the pooled study center as a random effect. This will also be presented graphically on a Kaplan-Meier curve. The hazard ratio with 95% CI will be reported. The log-rank test will be used to evaluate the statistical significance of the treatment effect.

Rate of new clinical vascular events (ischemic stroke, hemorrhagic stroke, TIA, myocardial infarction, or vascular death) and the individually event within 1 year

The rate of new clinical vascular events occurring within 1 year will be analyzed using a Cox proportional hazard model, with the pooled study center as a random effect. A combined vascular event is defined as any of the 5 following events: ischemic stroke, hemorrhagic stroke, TIA, myocardial infarction, or vascular death. This will also be presented graphically on a Kaplan-Meier curve. The hazard ratios with 95% CIs will be reported. The log-rank test will be used to evaluate the statistical significance of the treatment effect.

Rate of new ischemic stroke within 1 year

The rate of ischemic stroke occurring within 1 year will be analyzed using a Cox proportional hazard model, with the pooled study center as a random effect. This will also be presented graphically on a Kaplan-Meier curve. The hazard ratio with 95% CI will be reported. The log-rank test will be used to evaluate the statistical significance of the treatment effect.

Proportion of disabling stroke at 1 year

The proportion of disabling stroke at 1 year will be analyzed using a logistic regression model. The disabling stroke is defined as Modified Rankin Scale score (mRS) >1. The odds ratio with 95% CI will be reported.

Incidence and severity of recurrent stroke and TIA during follow-up to 1 year

Differences in proportion of severity of recurrent stroke and TIA during follow-up to 1 year between the two treatment groups will be tested by Wilcoxon rank sum test. Severity is measured using a six-level ordered categorical scale that incorporates the mRS: fatal stroke/severe non-fatal stroke [mRS 4 or 5]/moderate stroke [mRS 2 or 3]/mild stroke [mRS 0 or 1)]/TIA/no stroke-TIA. The test statistics with p value will be reported.

Quality of life at 1-year follow-up

Health related quality of life will be measured using EuroQol EQ−5D scale among the survivors. Treatment differences will be tested using student t−test or Wilcoxon rank sum test as appropriate.

13. Safety Analyses

All analyses of safety data will be carried out using the safety set (SS) population.

Extent of Exposure

The extent of exposure will be calculated as the number of days between start of treatment and end of treatment (i.e. treatment stop date – treatment start date + 1), and categorized into months: <1, 1−2, 2−3 months. Exposure will then be summarized by

treatment group.

Bleeding Events

Severe or moderate bleedings (GUSTO definition) at 3 months;

Severe or moderate bleedings (GUSTO definition) at 1 year;

Any bleeding events (severe or moderate bleedings or intracranial hemorrhage) at 3 months and 1 year;

For most bleeding events, the Cox proportional hazards model will be used to compare the hazard ratio (HR) between the two treatments, or Poisson regression or negative binomial regression which are more appropriate for the analysis of rare event.

Adverse Events

Adverse events (AEs) will be coded using the MedDRA coding dictionary (Version 6.0 or a later release) and grouped by system organ class (as detailed in the study protocol). Separate data display listings and summaries will be presented for adverse events that start prior to first dose of study medication (pre−treatment), whilst on study medication (during treatment) and after the last dose of study medication (post−treatment).

Within each treatment group, the number and percentage of subjects experiencing an AE will be summarized by system organ class and preferred term and Fisher's Exact test will be used to compare the number of each grouped AE event between treatment groups. In addition, a separate summary will be provided for AEs experienced by more than 5% of subjects in either of the treatment groups.

Deaths and Serious Adverse Events

Summary tables and data displays will be provided for serious adverse events (as detailed in the study protocol). In addition, all deaths and serious AE's will be documented in a case narrative format in the clinical study report.

The number of deaths occurring over the treatment period will be summarized and Fisher's Exact test will be used to compare the number of deaths between treatment groups.

14. References

1. Nagashima Z, Tsukahara K, Morita S, et al. Platelet reactivity in the early and late phases of acute coronary syndromes according to cytochrome p450 2c19 phenotypes. J Cardiol 2013; 62: 158–164.
2. Wang Y, Wang Y, Zhao X, et al. Clopidogrel with aspirin in acute minor stroke or transient ischemic attack. N Engl J Med 2013;369: 11-19.
3. Rabin R, Charro Fd. EQ-SD: a measure of health status from the EuroQol Group. Annals of Medicine 2001;33: 337-343.
4. Andersen P, Gill, RD. Cox's regression model for counting processes: a large sample study. Annals of Statistics 1982;10: 1100-1120.

Summary of amendments of statistical analysis plan

APPENDIX I:

Statistical analysis plan changes version 1.0 (Jan 09, 2019) to 1.1 (Sep 10, 2019)

SAP version 1.0	Changes in SAP version 1.1
2. Study Objective<br>7) Consistency analysis and performance evaluation of different rapid genotyping instruments.	Delete this objective
3. Study Endpoint(s)<br>Secondary Efficacy Endpoint:<br>1) New clinical vascular events (any stroke/ TIA/ myocardial infarction/ vascular death) within 3 months; At the same time, each new vascular event were evaluated independently;<br>7) New clinical vascular events (any stroke/ TIA/ myocardial infarction/ vascular death); At the same time, each new vascular event were evaluated independently;<br>10) Continuous changes in NIHSS scores at 1 year;	3. Study Endpoint(s)<br>Secondary Efficacy Endpoint:<br>2) New clinical vascular events (ischemic stroke/ hemorrhagic stroke/ TIA/ MI/ vascular death) within 3 months;<br>7) New clinical vascular events (ischemic stroke/ hemorrhagic stroke/ TIA/ MI/ vascular death) within 1-year;
5. Design<br>Eligibility criteria for the trial participants include 40 years of age or older than 40 years and less than 80 years,	5. Design<br>Eligibility criteria for the trial participants include 40 years of age or older,

	6. Sample size estimates<br>Based on the results of interim analysis, the DSMB will recommend whether the sample size need to be re-calculated.
12. Efficacy Analyses<br>**Rate of new clinical vascular events (any stroke, TIA, myocardial infarction, or vascular death) and the individually event within 3 months**<br>The rate of new clinical vascular events occurring within 3 months will be analyzed using a Cox proportional hazard model similar to that in the previous section, with the pooled study center as a random effect. A combined vascular event is defined as any of the 4 following events: stroke, TIA, myocardial infarction, or vascular death. This will also be presented graphically on a Kaplan-Meier curve. Each of the 4 vascular events will also be analyzed using the same method and the event rate for each event will be calculated for each treatment group. The hazard ratios with 95% CIs will be reported. The log-rank test will be used to evaluate the statistical significance of the treatment effect.	12. Efficacy Analyses<br>**Rate of new clinical vascular events (ischemic stroke, hemorrhagic stroke, TIA, myocardial infarction, or vascular death) within 3 months**<br>The rate of new clinical vascular events occurring within 3 months will be analyzed using a Cox proportional hazard model similar to that in the previous section, with the pooled study center as a random effect. A combined vascular event is defined as any of the 5 following events: ischemic stroke, hemorrhagic stroke, TIA, myocardial infarction, or vascular death. This will also be presented graphically on a Kaplan-Meier curve. The hazard ratios with 95% CIs will be reported. The log-rank test will be used to evaluate the statistical significance of the treatment effect.

**Rate of new clinical vascular events (any stroke, TIA, myocardial infarction, or vascular death) and the individually event within 1 year**<br>The rate of new clinical vascular events occurring within 1 year will be analyzed using a Cox proportional hazard model, with the pooled study center as a random effect. A combined vascular event is defined as any of the 4 following events: stroke, TIA, myocardial infarction, or vascular death. This will also be presented graphically on a Kaplan-Meier curve. Each of the 4 vascular events will also be analyzed using the same method and the event rate for each event will be calculated for each treatment group. The hazard ratios with 95% CIs will be reported. The log-rank test will be used to evaluate the statistical significance of the treatment effect.	**Rate of new clinical vascular events (ischemic stroke, hemorrhagic stroke, TIA, myocardial infarction, or vascular death) and the individually event within 1 year**<br>The rate of new clinical vascular events occurring within 1 year will be analyzed using a Cox proportional hazard model, with the pooled study center as a random effect. A combined vascular event is defined as any of the 5 following events: ischemic stroke, hemorrhagic stroke, TIA, myocardial infarction, or vascular death. This will also be presented graphically on a Kaplan-Meier curve. The hazard ratios with 95% CIs will be reported. The log-rank test will be used to evaluate the statistical significance of the treatment effect.

APPENDIX II:

Statistical analysis plan changes version 1.1 (Sep 10, 2019) to 1.2 (Dec 09, 2020)

SAP version 1.1	Changes in SAP version 1.2
2. Study Objective<br>2) To assess separately the effects of ticagrelor plus aspirin regimen versus	2. Study Objective<br>2) To assess the effects of ticagrelor plus aspirin regimen versus clopidogrel plus

clopidogrel plus aspirin regimen on the occurrence of: ischemic stroke, hemorrhagic stroke, TIA, MI, vascular death, death of all-cause, disability (mRS: 2-6) at 3-month and at one-year;	aspirin regimen on the incidence of: stroke within 30 days, neurological impairment at 3 months (NIHSS increased ≥4 from baseline), ischemic stroke, TIA, MI, vascular death, disabling stroke (mRS: 2-6) at 3-month and at one-year;
	3) To assess the incidence and severity of recurrent stroke and TIA during follow-up to 3 months and 1-year (Severity is measured using a six-level ordered categorical scale that incorporates the mRS: fatal stroke/severe non-fatal stroke [mRS 4 or 5]/moderate stroke [mRS 2 or 3]/mild stroke [mRS 0 or 1]/TIA/no stroke-TIA);
4) Subgroup analyses: Efficacy endpoint will also be analyzed stratified by gender (men vs. women), age (<65 vs. ≥65 years), by etiology subtype based on the TOAST (Trial of ORG 10172 in Acute Stroke Treatment) subtype system, by diabetes (yes vs. no) and by type of LOF allele (intermediate metabolizers vs. poor metabolizers), index event (TIA or MIS), and location of symptomatic artery stenosis (intracranial/extracranial).	6) Subgroup analyses: The primary efficacy endpoint will also be analyzed stratified by age (<65 vs. ≥65 years), gender (men vs. women), Body Mass Index (BMI), index event (TIA vs. Minor stroke), time from index event to randomization, etiology subtype, diabetes mellitus, hypertension, type of LOF allele, previous ischemic stroke or TIA, prior antiplatelet therapy, prior statin therapy, prior smoking status, and symptomatic intracranial and extracranial artery stenosis will be

	evaluated in subgroup analyses.
6) To evaluate the health economics indicators of the two treatment regimens.	Delete the objective.
3. Study Endpoint(s)<br>Secondary Efficacy Endpoint:<br>1) Any new stroke events (ischemic stroke or hemorrhagic stroke) within 1 year;<br>4) Modified Rankin Scale score dichotomized at percentage with score 0-2 vs. 3-6 at 3 months follow-up;<br>9) Modified Rankin Scale score dichotomized at percentage with score 0-2 vs. 3-6 at 1-year follow-up;	3. Study Endpoint(s)<br>Secondary Efficacy Endpoint:<br>1) Any new stroke events (ischemic stroke or hemorrhagic stroke) within 30 days and 1 year;<br>4) Disabling stroke (Modified Rankin Scale score, mRS>1) at 3 months and 1 year;<br>5) Incidence and severity of recurrent stroke and TIA during follow-up to 3 months and 1-year (Severity is measured using a six-level ordered categorical scale that incorporates the mRS: fatal stroke/severe non-fatal stroke [mRS 4 or 5]/moderate stroke [mRS 2 or 3]/mild stroke [mRS 0 or 1)]/TIA/no stroke-TIA);
5. Design<br>Secondary outcomes include following event within a 3 months and 1 year timeframe: (1) Composite major cardiovascular events including stroke, TIA, myocardial infarction, and cardiovascular deaths; (2) Ischemic stroke; (3) Modified Rankin Scale score	5. Design<br>Secondary outcomes include any new stroke within 30 days and 1 year, following event within a 3 months and 1 year timeframe: (1) new clinical vascular events including ischemic stroke, hemorrhagic stroke, TIA, myocardial infarction, and vascular deaths; (2)

dichotomized at percentage with score 0-2 vs. 3-6; (4) Neurological impairment (changes in NIHSS scores at 3 month follow-up); (5) Quality of Life (EuroQol EQ-5D scale.	Ischemic stroke; (3) Disabling stroke (Modified Rankin Scale score, mRS>1); (4) Incidence and severity of recurrent stroke and TIA; (5) Quality of Life (EuroQol EQ-5D scale; and Neurological impairment at 3 months (NIHSS increased ≥4 from baseline).
7. Analysis populations<br>According to the basic principle of intention-to-treat (ITT), all patients who were enrolled, randomized to groups and had the record of at least one-day treatment of study drugs will be included. This population will be the primary population for analyses of efficacy.	7. Analysis populations<br>According to the basic principle of intention-to-treat (ITT), all patients who were enrolled, randomized and had the record of at least one-day treatment of study drugs will be included. Subjects missing outcome data will be censored at the last follow-up assessment time (end of study or last visit preceding loss to follow up). This population will be the primary population for analyses of efficacy.
	**Safety Set (SS)**<br>Throughout the safety results sections, erroneously treated patients (eg, those randomized to ticagrelor and aspirin group but actually given clopidogrel and aspirin) will be accounted for in the actual treatment group.

12. Efficacy Analyses<br>**Interactions with Subgroups**<br>The predefined subgroups including:<br>● Gender (man vs. women)<br>● Different age category (<65 years vs. ≥65 years)<br>● Etiological stroke subtype based on the TOAST Classification<br>● History of diabetes (yes vs. no)<br>● CYP2C19 genetic variants (intermediate metabolizers vs. poor metabolizers)<br>● Index event (TIA vs. minor ischemic stroke)<br>● Location of symptomatic artery stenosis (Intracranial artery stenosis vs. extracranial artery stenosis)	12. Efficacy Analyses<br>**Interactions with Subgroups**<br>The predefined subgroups including:<br>● Age (<65 years vs. ≥65 years)<br>● Gender (men vs. women)<br>● BMI<br>● Index event (TIA vs. minor ischemic stroke)<br>● Time from index event to randomization<br>● Etiological stroke subtype based on the TOAST Classification<br>● Diabetes mellitus<br>● Hypertension<br>● Type of CYP2Y19 LOF alleles (intermediate metabolizers vs. poor metabolizers)<br>● Previous ischemic stroke or TIA<br>● Prior antiplatelet therapy<br>● Prior statin therapy<br>● Prior smoking status<br>● Location of symptomatic artery stenosis (Intracranial artery stenosis vs. extracranial artery stenosis)
	**Rate of new stroke events within 30 days**<br>The rate of stroke occurring within 30 days will be analyzed using a Cox proportional hazard model, with the

	pooled study center as a random effect. This will also be presented graphically on a Kaplan-Meier curve. The hazard ratio with 95% CI will be reported. The log-rank test will be used to evaluate the statistical significance of the treatment effect.
**Modified Rankin Scale score dichotomized at percentage with score 0-2 vs 3-6 at 3 months follow up**<br>Logistic regression will be performed to compare proportion of Modified Rankin Scale score 0-2 vs 3-6 at 3 months follow-up between the two treatment groups. The odds ratio with 95% CI will be reported.<br>**Neurological impairment at 3 months follow-up**<br>Changes of NIHSS scores between the end of study and baseline will be summarized for the two treatment groups among the survivors during the study. The treatment difference will be tested using student t-test or Wilcoxon rank sum test as appropriate.	**Proportion of disabling stroke at 3 months**<br>The proportion of disabling stroke at 3 months will be analyzed using a logistic regression model. The disabling stroke is defined as Modified Rankin Scale score (mRS) >1. The odds ratio with 95% CI will be reported.<br>**Incidence and severity of recurrent stroke and TIA during follow-up to 3 months**<br>Differences in proportion of severity of recurrent stroke and TIA during follow-up to 3 months between the two treatment groups will be tested by Wilcoxon rank sum test. Severity is measured using a six-level ordered categorical scale that incorporates the mRS: fatal stroke/severe non-fatal stroke [mRS 4 or 5]/moderate stroke [mRS 2 or 3]/mild stroke [mRS 0 or 1)]/TIA/no stroke-TIA. The test statistics with p

	value will be reported.<br>**Proportion of neurological impairment at 3 months**<br>The proportion of neurological impairment will be analyzed using a logistic regression model. The neurological impairment is defined as NIHSS increased ≥4 from baseline. The odds ratio with 95% CI will be reported.
**Modified Rankin Scale score dichotomized at percentage with score 0-2 vs 3-6 at 1-year follow up**<br>Logistic regression will be performed to compare proportion of Modified Rankin Scale score 0-2 vs 3-6 at 1-year follow-up between the two treatment groups. The odds ratio with 95% CI will be reported.	**Proportion of disabling stroke at 1 year**<br>The proportion of disabling stroke at 1 year will be analyzed using a logistic regression model. The disabling stroke is defined as Modified Rankin Scale score (mRS) >1. The odds ratio with 95% CI will be reported.<br>**Incidence and severity of recurrent stroke and TIA during follow-up to 1 year**<br>Differences in proportion of severity of recurrent stroke and TIA during follow-up to 1 year between the two treatment groups will be tested by Wilcoxon rank sum test. Severity is measured using a six-level ordered categorical scale that incorporates the mRS: fatal stroke/severe non-fatal stroke [mRS 4 or 5]/moderate stroke [mRS 2 or 3]/mild stroke [mRS 0

	or 1)]/TIA/no stroke-TIA. The test statistics with p value will be reported.

APPENDIX III:

Statistical analysis plan changes version 1.2 (Dec 09, 2020) to 1.3 (April 20, 2021)

SAP version 1.2	Changes in SAP version 1.3
	6. Sample size estimates<br>According to the DSMB meeting on April 7, 2021, the DSMB lookup the overall event rates of the trial and suggested not to unblind the treatment assignment in the interim analysis. Therefore, comparison of efficacy and safety outcome between two treatment groups were not performed in the interim analysis, and sample size was not re-calculated. The type I error level of the statistical significance will be set to a 2-sided α of 0.05 in the final analysis.
