# Supplementary material for: Association of CYP2C19 Loss-of-Function Metabolizer Status With Stroke Risk Among Chinese Patients Treated With Ticagrelor-Aspirin vs Clopidogrel-Aspirin: A Prespecified Secondary Analysis of a Randomized Clinical Trial
Source: JAMA Netw Open. 2023 Jun 6;6(6):e2317037. doi: 10.1001/jamanetworkopen.2023.17037 (PMC10245195; doi:10.1001/jamanetworkopen.2023.17037)
Supplement: Supplement 2. — eTable 1. Baseline Characteristics of the Patients Classified by CYP2C19 Genotypes eTable 2. Primary Efficacy and Safety Outcomes by Genotypes eTable 3. Association of Intermediate in Patients Receiving Ticagrelor-Aspirin or Clopidogrel-Aspirin eFigure 1. The Flowchart of the Study eFigure 2. Cumulative Probability of Stroke According to Loss-of-Function Allele Carrier Status [file jamanetwopen-e2317037-s002.pdf]

Supplementary Online Content

Xie X, Johnston SC, Wang A, et al. Association of *CYP2C19* loss-of-function metabolizer status with stroke risk among Chinese patients treated with ticagrelor-aspirin vs clopidogrel-aspirin: a prespecified secondary analysis of a randomized clinical trial. *JAMA Netw Open.* 2023;6(6):e2317037. doi:10.1001/jamanetworkopen.2023.17037

eTable 1. Baseline Characteristics of the Patients Classified by *CYP2C19* Genotypes
eTable 2. Primary Efficacy and Safety Outcomes by Genotypes
eTable 3. Association of Intermediate in Patients Receiving Ticagrelor-Aspirin or Clopidogrel-Aspirin
eFigure 1. The Flowchart of the Study
eFigure 2. Cumulative Probability of Stroke According to Loss-of-Function Allele Carrier Status

This supplementary material has been provided by the authors to give readers additional information about their work.

© 2023 Xie X et al. *JAMA Network Open.*

eTable 1. Baseline Characteristics of the Patients Classified by *CYP2C19* Genotypes

Characteristic	Intermediate metabolizers						Poor metabolizers					
	***1/*2**		***1/*3**		***2/*17；*3/*17**		***2/*2**		***3/*3**		***2/*3**	
	**T+A**	**C+A**	**T+A**	**C+A**	**T+A**	**C+A**	**T+A**	**C+A**	**T+A**	**C+A**	**T+A**	**C+A**
Median age (IQR) -yr	65.28(57.14-71.73)	64.69(56.94-71.04)	64.74(56-70.81)	64.26(56.72-71.38)	62.47(56.6-71.25)	63.32(56.01-69.22)	64.34(56.72-71.89)	65.11(56.58-71.63)	64.28(57.2-75.13)	63.98(57.85-68.52)	64.35(57.53-70.84)	64.07(57.47-70.51)
Female sex - no. (%)	720 (34.53)	712 (34.02)	122 (35.06)	111 (30.08)	13 (24.53)	13 (24.53)	172 (33.14)	175 (34.18)	8 (27.59)	9 (39.13)	55 (32.16)	60 (38.22)
Han ethnicity - no. (%)	2052 (98.42)	2051 (97.99)	336 (96.55)	356 (96.48)	52 (98.11)	51 (96.23)	509 (98.07)	504 (98.44)	27 (93.10)	20 (86.96)	168 (98.25)	156 (99.36)
Median body Mass Index (IQR)* - kg/m2	24.49(22.6-26.62)	24.34(22.5-26.42)	24.83(23.14-27.04)	24.31(22.49-26.47)	24.93(23.12-26.67)	24.49(22.49-26.67)	24.39(22.49-26.67)	24.24(22.76-26.35)	24.62(22.86-27.04)	25.39(23.44-26.95)	24.49(22.66-26.3)	24.22(22.04-26.57)
Median blood pressure (IQR) - mm Hg												
Systolic	148(136-161)	148(136-161.5)	149(135.75-164)	148(132-160)	149(130-163)	145(136-161)	149(136-164)	149(136-162)	152(142-173)	151(140-163)	147(134-160)	149(139-160)
Diastolic	86(80-95)	86(80-95)	88(80-94)	86(80-95)	87(80-94)	87(80-92)	88(79-95)	87(80-95)	90(80-93)	88(75-94)	86(80-95)	86(78-94)
Medical history - no. (%)												
Hypertension	1537 (73.72)	1572 (75.11)	253 (72.70)	257 (69.65)	33 (62.26)	36 (67.92)	387 (74.57)	375 (73.24)	20 (68.97)	17 (73.91)	126 (73.68)	117 (74.52)
Diabetes mellitus	666 (31.94)	646 (30.86)	115 (33.05)	131 (35.50)	18 (33.96)	12 (22.64)	164 (31.60)	150 (29.30)	14 (48.28)	11 (47.83)	56 (32.75)	59 (37.58)
Dyslipidemia	212 (10.17)	192 (9.17)	38 (10.92)	31 (8.40)	4 (7.55)	2 (3.77)	49 (9.44)	44 (8.59)	5 (17.24)	2 (8.70)	15 (8.77)	20 (12.74)
Previous ischemic stroke	404 (19.38)	452 (21.60)	82 (23.56)	76 (20.60)	17 (32.08)	7 (13.21)	123 (23.70)	114 (22.27)	8 (27.59)	3 (13.04)	35 (20.47)	29 (18.47)
Previous TIA	28 (1.34)	28 (1.34)	7 (2.01)	4 (1.08)	1 (1.89)	2 (3.77)	7 (1.35)	5 (0.98)	0 (0.00)	1 (4.35)	3 (1.75)	2 (1.27)
Myocardial infarction	41 (1.97)	32 (1.53)	4 (1.15)	4 (1.08)	2 (3.77)	0 (0.00)	6 (1.16)	5 (0.98)			1 (0.58)	1 (0.64)
Angina	54 (2.59)	65 (3.11)	8 (2.30)	9 (2.44)	1 (1.89)	2 (3.77)	16 (3.08)	14 (2.73)	1 (3.45)	0 (0.00)	5 (2.92)	2 (1.27)

© 2023 Xie X et al. *JAMA Network Open.*

Characteristic	Intermediate metabolizers						Poor metabolizers					
	*1/*2		*1/*3		*2/*17；*3/*17		*2/*2		*3/*3		*2/*3	
	T+A	C+A	T+A	C+A	T+A	C+A	T+A	C+A	T+A	C+A	T+A	C+A
Peripheral vascular disease	4 (0.19)	3 (0.14)	1 (0.29)	1 (0.27)			0 (0.00)	3 (0.59)			1 (0.58)	0 (0.00)
Median time to randomization (IQR) - hr	13.67(8.95-20.33)	14(8.82-20.52)	13.42(9.21-20)	14.4(9-20.5)	14.57(9.18-19.2)	14.75(9.88-19.85)	13.43(8.67-20.5)	14.98(9.1-21.33)	12(8.58-16.17)	17.83(10.3-22.5)	14(8.67-21.02)	14.78(9.02-20.45)
Time to randomization - no. (%)												
<12 hr	848 (40.67)	891 (42.57)	148 (42.53)	139 (37.67)	21 (39.62)	18 (33.96)	226 (43.55)	186 (36.33)	14 (48.28)	7 (30.43)	71 (41.52)	57 (36.31)
≥12 hr	1237 (59.33)	1202 (57.43)	200 (57.47)	230 (62.33)	32 (60.38)	35 (66.04)	293 (56.45)	326 (63.67)	15 (51.72)	16 (69.57)	100 (58.48)	100 (63.69)
Qualifying event - no. (%)												
Ischemic stroke	1675 (80.34)	1697 (81.08)	287 (82.47)	300 (81.30)	40 (75.47)	43 (81.13)	420 (80.92)	400 (78.13)	24 (82.76)	19 (82.61)	131 (76.61)	122 (77.71)
TIA	410 (19.66)	396 (18.92)	61 (17.53)	69 (18.70)	13 (24.53)	10 (18.87)	99 (19.08)	112 (21.88)	5 (17.24)	4 (17.39)	40 (23.39)	35 (22.29)
Median NIHSS score in patients with qualifying ischemic stroke † (IQR)	2(1-3)	2(1-3)	2(1-3)	2(1-2)	2(1-3)	2(1-2)	2(1-3)	2(1-3)	2(1-3)	2(2-3)	2(1-3)	2(1-3)
Median ABCD2 score in patients with qualifying TIA ‡ (IQR)	5(4-5)	4.5(4-5)	5(4-5)	4(4-5)	4(4-5)	5(4-6)	4(4-5)	5(4-5)	6(5-6)	4(4-4)	4(4-5)	4(4-5)
Previous antiplatelet therapy§ - no. (%)	241 (11.56)	254 (12.14)	40 (11.49)	37 (10.03)	12 (22.64)	4 (7.55)	72 (13.87)	53 (10.35)	3 (10.34)	2 (8.70)	17 (9.94)	13 (8.28)

© 2023 Xie X et al. *JAMA Network Open.*

Characteristic	Intermediate metabolizers						Poor metabolizers					
	*1/*2		*1/*3		*2/*17; *3/*17		*2/*2		*3/*3		*2/*3	
	T+A	C+A	T+A	C+A	T+A	C+A	T+A	C+A	T+A	C+A	T+A	C+A
Previous lipid-lowering therapy§ - no. (%)	170 (8.15)	161 (7.69)	28 (8.05)	26 (7.05)	4 (7.55)	2 (3.77)	40 (7.71)	38 (7.42)	3 (10.34)	2 (8.70)	13 (7.60)	12 (7.64)

T+A denotes Ticagrelor–Aspirin; C+A, Clopidogrel-Aspirin TIA and IQR, interquartile range.

* The body-mass index is the weight in kilograms divided by the square of the height in meters.

†Medication after onset to before randomization

‡ Data are only for the patients who had a TIA. The ABCD2 assesses the risk of stroke on the basis of age, blood pressure, clinical features, duration of TIA, and presence or absence of diabetes, with scores ranging from 0 to 7 and higher scores indicating greater short-term risk.

© 2023 Xie X et al. *JAMA Network Open.*

eTable 2. Primary Efficacy and Safety Outcomes by Genotypes

Outcomes	Ticagrelor- Aspirin, No（%）	Clopidogrel-Aspirin, No（%）	Hazard Ratios (95% CI)	P Value
***1/*2**				
Stroke within 3 months	116 (5.56)	159 (7.60)	0.71(0.56-0.91)	0.0060
Severe or moderate bleeding at 3months	6 (0.29)	9 (0.43)	0.67(0.24-1.89)	0.4506
***1/*3**				
Stroke within 3 months	32 (9.20)	26 (7.05)	1.52(0.83-2.77)	0.1757
Severe or moderate bleeding at 3 months *	1 (0.29)	0 (0.00)	NA	NA
***2/*17; *3/*17**				
Stroke within 3 months	2 (3.77)	6 (11.32)	0.31(0.03-3.17)	0.3266
Severe or moderate bleeding at 3 months	0 (0.00)	1 (1.89)	NA	NA
***2/*2**				
Stroke within 3 months	30 (5.78)	38 (7.42)	0.81(0.49-1.35)	0.4220
Severe or moderate bleeding at 3 months *	2 (0.39)	0 (0.00)	NA	0.9978
***3/*3**				
Stroke within 3 months	1 (3.45)	1 (4.35)	NA	NA
Severe or moderate bleeding at 3 months	0/29 (0)	0/23 (0)	NA	NA
***2/*3**				
Stroke within 3 months	10 (5.85)	13 (8.28)	1.28(0.48-3.44)	0.6231
Severe or moderate bleeding at 3 months *	0 (0.00)	1 (0.64)	NA	1.0000

* Severe or moderate bleeding at 3 months GUSTO definition.

© 2023 Xie X et al. *JAMA Network Open.*

eTable 3. Association of Intermediate in Patients Receiving Ticagrelor-Aspirin or Clopidogrel-Aspirin

Outcomes	Ticagrelor–Aspirin, No. (%)		Hazard Ratio (95% CI)	P	Clopidogrel–Aspirin, No. (%)		Hazard Ratio (95% CI)	P
	IM (N=2486)	PM (N=2515)			IM (N=719)	PM (N=692)		
**Primary outcome**								
Stroke	150 (6.03)	41 (5.70)	1.00(0.70-1.42)	0.977	191 (7.59)	52 (7.51)	1.01(0.73-1.38)	0.977
**Secondary outcome**								
Stroke within 30 days	125 (5.03)	31 (4.31)	0.89(0.60-1.33)	0.570	157 (6.24)	48 (6.94)	1.14(0.81-1.59)	0.449
Composite vascular events†	174 (7.00)	55 (7.65)	1.12(0.82-1.53)	0.465	232 (9.22)	61 (8.82)	0.95(0.71-1.28)	0.750
Ischemic stroke	148 (5.95)	41 (5.70)	1.01(0.71-1.44)	0.945	187 (7.44)	51 (7.37)	1.01(0.73-1.39)	0.959
Disabling stroke‡	74 (2.98)	23 (3.20)	1.17(0.72-1.88)	0.530	70 (2.78)	22 (3.18)	1.18(0.72-1.94)	0.507
**Primary safety outcome**								
Severe or moderate bleeding¶	7 (0.28)	2 (0.28)	0.87(0.18-4.22)	0.860	10 (0.40)	1 (0.14)	0.31(0.04-2.46)	0.270
Fatal bleeding	2 (0.08)	1 (0.14)	1.79(0.16-0.39)	0.640	3 (0.12)	0 (0.00)		
Intracranial hemorrhage	3 (0.12)	0 (0.00)			5 (0.20)	1 (0.14)	0.70(0.08-5.97)	0.741
**Secondary safety outcome**								
Any bleeding	134 (5.39)	36 (5.01)	0.90(0.61-1.31)	0.574	66 (2.62)	14 (2.02)	0.76(0.41-1.39)	0.370
Mild bleeding ¶	127 (5.11)	34 (4.73)	0.90(0.61-1.33)	0.585	56 (2.23)	13 (1.88)	0.85(0.45-1.61)	0.618
Mortality	5 (0.20)	4 (0.56)	3.40(0.84-13.80)	0.087	15 (0.60)	3 (0.43)	0.81(0.23-2.87)	0.747

© 2023 Xie X et al. *JAMA Network Open.*

**Outcomes**	**Ticagrelor–Aspirin, No. (%)**		**Hazard Ratio (95% CI)**	**P**	**Clopidogrel–Aspirin, No. (%)**		**Hazard Ratio (95% CI)**	**P**
	**IM (N=2486)**	**PM (N=2515)**			**IM (N=719)**	**PM (N=692)**		
Adverse event	419 (16.85)	121 (16.83)	0.91(0.74-1.12)	0.376	339 (13.48)	88 (12.72)	0.92(0.72-1.17)	0.472
Serious adverse event	60 (2.41)	18 (2.50)	1.07(0.62-1.85)	0.808	63 (2.50)	21 (3.03)	1.33(0.80-2.22)	0.278

© 2023 Xie X et al. *JAMA Network Open.*

eFigure 1. The Flowchart of the Study

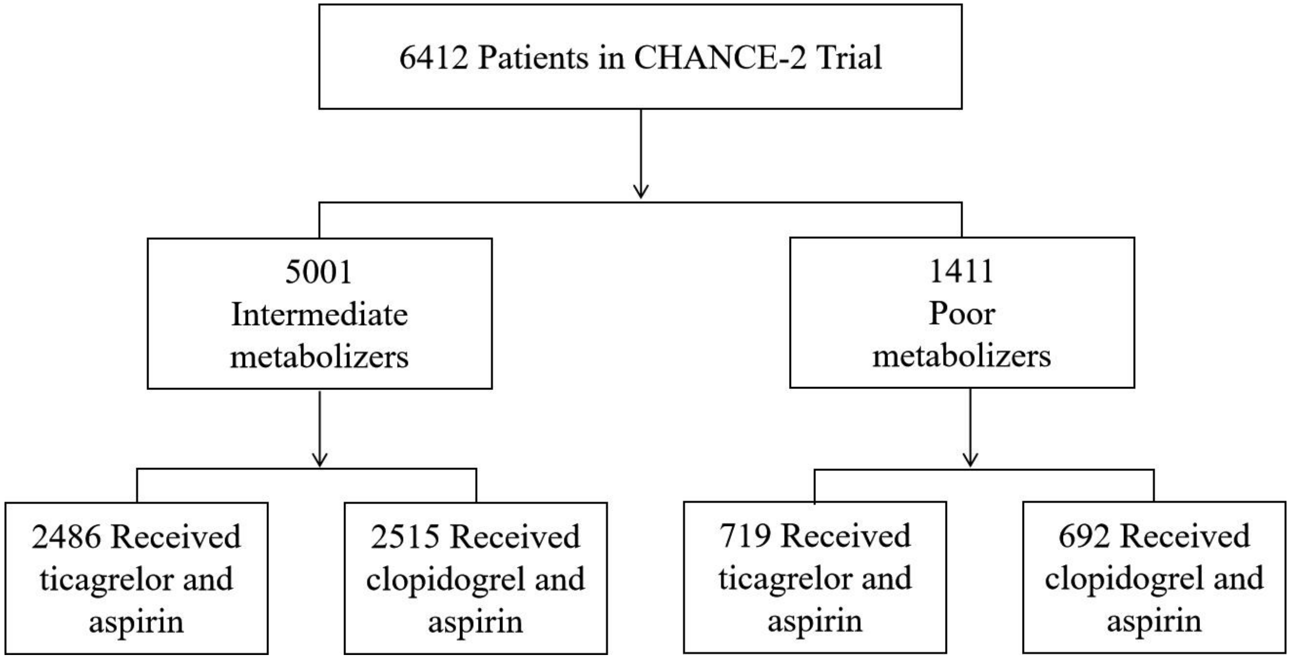

© 2023 Xie X et al. *JAMA Network Open*.

eFigure 2. Cumulative Probability of Stroke According to Loss-of-Function Allele Carrier Status

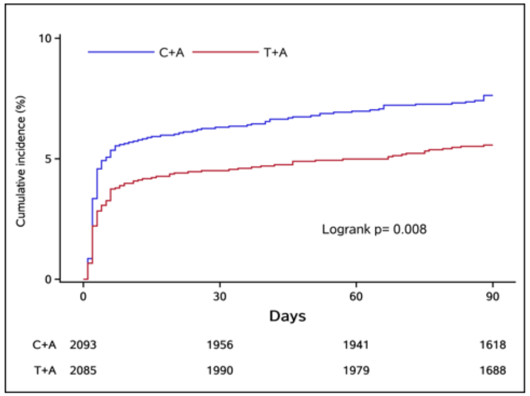

***1/*2**

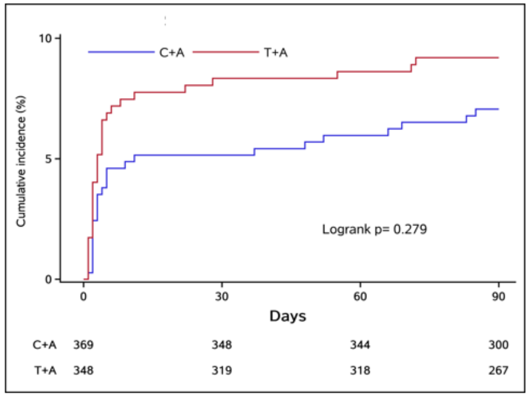

***1/*3**

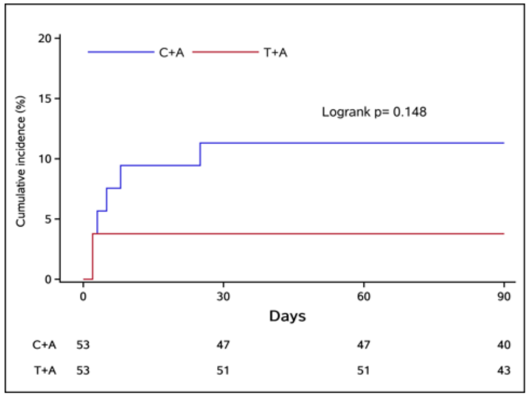

***2/*17; *3/*17**

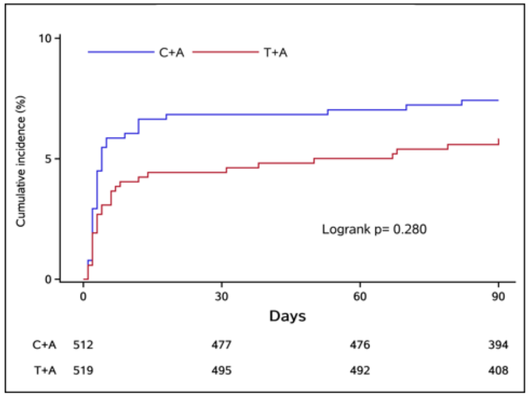

***2/*2**

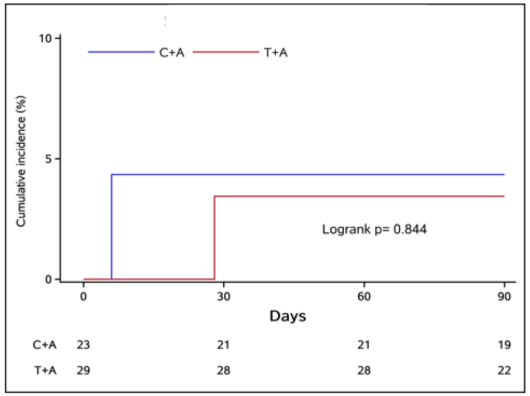

***3/*3**

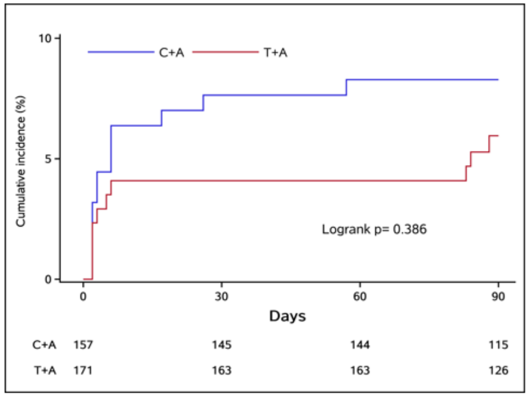

***2/*3**

© 2023 Xie X et al. *JAMA Network Open.*
