# Supplementary material for: Association of CYP2C19 Loss-of-Function Metabolizer Status With Stroke Risk Among Chinese Patients Treated With Ticagrelor-Aspirin vs Clopidogrel-Aspirin: A Prespecified Secondary Analysis of a Randomized Clinical Trial
Source: JAMA Netw Open. 2023 Jun 6;6(6):e2317037. doi: 10.1001/jamanetworkopen.2023.17037 (PMC10245195; doi:10.1001/jamanetworkopen.2023.17037)
Supplement: Supplement 3. — Data Sharing Statement [file jamanetwopen-e2317037-s003.pdf]

# Data Sharing Statement

Xie. Association of CYP2C19 Loss-of-Function Metabolizer Status With Stroke Risk Among Chinese Patients Treated With Ticagrelor-Aspirin vs Clopidogrel-Aspirin. *JAMA Netw Open*. Published June 06, 2023. doi:10.1001/jamanetworkopen.2023.17037

**Data**
**Data available:** No
